# Lip and Oral Cancer, Caries and Other Oral Conditions: Estimates From the 2021 Global Burden of Disease Study and Projections up to 2050

**DOI:** 10.1111/jre.13421

**Published:** 2025-06-18

**Authors:** Silas Alves‐Costa, Mario Romandini, Gustavo G. Nascimento

**Affiliations:** ^1^ Graduate Program in Dentistry Federal University of Maranhão São Luís Brazil; ^2^ Perio‐Implant Innovation Center Institute for Integrated Oral, Craniofacial and Sensory Research—National Clinical Research Center of Stomatology, Ninth People's Hospital, Shanghai Jiao Tong University School of Medicine Shanghai China; ^3^ National Dental Research Institute Singapore National Dental Centre Singapore Singapore; ^4^ Oral Health Academic Clinical Programme Duke‐NUS Medical School Singapore

**Keywords:** caries, demography, epidemiologic factors, epidemiology, forecasting, global disease burden, global health, morbidity, oral cancer, oral conditions

## Abstract

**Aim:**

To (i) assess the prevalence, incidence, and burden of lip and oral cavity cancer, untreated caries, and “other oral conditions” (a group that includes temporomandibular disorders, malocclusion, and dental trauma, among others) in 2021; and (ii) forecast their estimates in 2050. Aggregate estimates for overall oral conditions (comprising caries, periodontitis, and edentulism, but excluding cancer) were also evaluated.

**Methods:**

Prevalence, incidence, Years Lived with Disability (YLDs), Years of Life Lost (YLLs), Disability‐Adjusted Life Years (DALYs), and deaths were reported for lip and oral cavity cancer. Untreated caries and “other oral conditions” were described as prevalence and YLDs, with incidence included only for caries. Aggregate estimates for overall oral conditions encompassed prevalence, incidence, and YLDs. Data were gathered globally, covering 204 countries, seven super‐regions, and 21 regions from the Global Burden of Disease (GBD) study in 2021, with projections up to 2050 using mixed‐effects models.

**Results:**

In 2021, over 1.54 million (95% UI 1.44; 1.63) people worldwide were affected by lip and oral cavity cancer, with a global age‐standardized prevalence of 0.02%. The burden included 1.28 YLDs per 100 000 (0.97; 1.59), 65.86 YLLs per 100 000 (59.53; 71.00), 67.71 DALYs per 100 000 (61.32; 73.17), and 2.42 deaths per 100 000 (2.21; 2.60). By 2050, prevalence and incidence are projected to increase by +68.7% and + 82.6%, respectively. By that time, lip and oral cavity cancer is expected to rank 119th in terms of YLDs. In 2021, 7.55% (6.29–8.78) of children had untreated caries in deciduous teeth, 27.54% (23.98–32.02) of the adult population had caries in permanent teeth, and 1.86% (1.78–1.93) were affected by “other oral conditions”, with 45.91% (42.26–49.78) of the global population experiencing at least one oral condition. By 2050, the prevalence of deciduous caries is projected to decrease by −15.80%, while permanent caries is predicted to rise by +23.77%, and “other oral conditions” will increase by +22.28%. Overall oral conditions (including periodontitis and edentulism, but excluding cancer) are projected to affect 46.17% (42.52–49.95) of the global population in 2050, ranking as the 10th leading cause of YLDs, surpassing conditions such as stroke and Alzheimer's disease.

**Conclusion:**

Lip and oral cavity cancer, along with oral conditions aggregated (caries, periodontitis, edentulism, “other oral conditions”), continued to pose significant public health challenges in 2021, with the number of affected individuals expected to increase substantially in the coming decades, largely driven by rising estimates of edentulism and severe periodontitis.

## Graphical abstract

Oral conditions and oral cancers affect billions globally. By 2050, there is expected to be a substantial increase in the prevalence, incidence, and burden of lip and oral cavity cancer and oral conditions (including caries, periodontitis and edentulism), emphasizing the urgent need for targeted public health interventions.

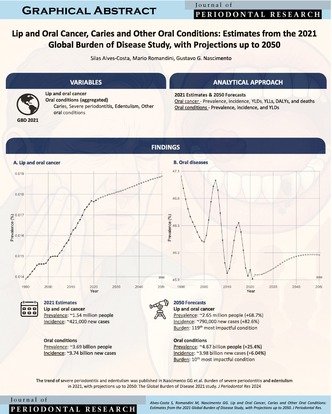

## 1 Introduction

Oral health inequalities remain a critical global challenge, disproportionately affecting vulnerable populations and widening disparities in care access. The financial burden of oral conditions has surged over the past decade, with global costs rising from an estimated US$442 billion in 2010 [1] to US$710 billion in 2019, encompassing both direct dental expenses and indirect productivity losses [2]. Stark disparities persist, with per capita dental spending in high‐income nations reaching up to 500 times that of low‐income regions [2]. In response, the World Health Organization (WHO) has reinforced the urgency of universal oral health coverage, underscored by the 2024 Bangkok Declaration, which calls for equitable access to essential oral healthcare by 2030 [3].

Accurately assessing the evolving burden of oral conditions is critical for global healthcare planning, particularly amid population growth in low‐income regions and aging trends in high‐income nations [4]. Given the interconnected nature of oral conditions [5, 6, 7, 8], where periodontitis, caries, and oral cancers share risk factors and systemic health implications, examining these conditions collectively aligns with the growing recognition of oral health as a unified, multisystem challenge.

Building on recent work by Nascimento et al. [9], which detailed the burden of periodontitis and edentulism in 2021 along with projections up to 2050, this analysis expands the lens to include lip and oral cavity cancer and a broader category of aggregated oral conditions, comprising untreated dental caries (in both permanent and deciduous teeth), periodontitis, edentulism, and “other oral conditions” (such as temporomandibular disorders, malocclusion, and dental trauma). Specifically, we aimed to (i) assess the prevalence, incidence, and burden of these conditions in 2021 and (ii) project their global, regional, and national burden by 2050, thereby providing a data‐driven basis for future health planning and policy‐making.

## 2 Methods

This manuscript adheres to the Guidelines for Accurate and Transparent Health Estimates Reporting (GATHER) statement [10].

### 2.1 Global Burden of Disease 2021

This study draws data from the Global Burden of Disease (GBD) 2021 study, curated by the Institute for Health Metrics and Evaluation (IHME) at the University of Washington. The GBD study integrates data from systematic reviews, opportunistic searches, national collaborators, and the World Health Organization, with an iterative data collection process that is continuously updated to incorporate new sources. Comprehensive details on data acquisition across GBD iterations, including GBD 2021, are available in the GBD capstone manuscript [11].

**FIGURE 1:**
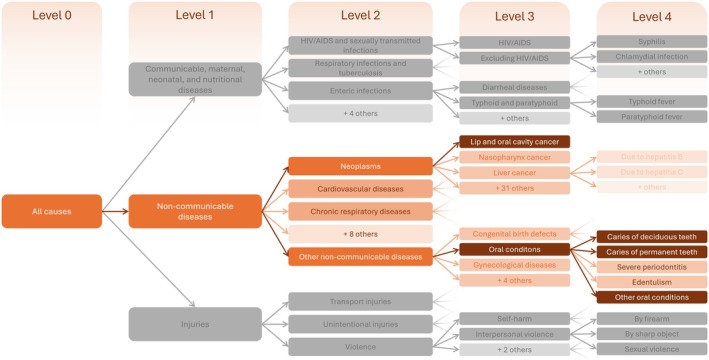
Schematic representation of the levels of the GBD 2021 hierarchy of causes.

The GBD 2021 study categorizes the burden of 371 diseases and injuries into a hierarchical system with five levels (Figure 1), described in detail elsewhere [12]. In this structure, Level 0 represents the overall disease burden, while each subsequent level (from Level 1 to Level 4) provides increasing detail, with Level 4 consisting of the most specific, fully disaggregated conditions. Detailed information on data sources, processing methods, and modeling strategies, including the meta‐regression Bayesian regularized trimmed (MR‐BRT) approach used for data crosswalking, can be found in the GBD capstone publication [11].

### 2.2 Case Definitions

Oral conditions and lip/oral cavity cancer were classified by GBD as distinct Level 3 causes (Figure 1).

#### 2.2.1 Lip and Oral Cavity Cancer (Level 3)

In the GBD 2021, this level represented malignant neoplasms of the lips and oral cavity, corresponding to ICD‐10 codes C00–C08. This condition was included in the neoplasms group (Level 2) and was not sub‐classified at Level 4 (Figure 1).

#### 2.2.2 Oral Conditions (Level 3)

Oral conditions were categorized within the non‐communicable diseases group (Level 2) and further classified into the following Level 4 conditions: untreated caries of deciduous and permanent teeth, severe periodontitis, edentulism, and “other oral conditions” (Figure 1). The burden of severe periodontitis and edentulism in 2021, along with specific projections to 2050, is reported elsewhere [9].

##### 2.2.2.1 Caries

Untreated caries in deciduous and permanent teeth were defined by the presence of a distinct cavity, undermined enamel, a softened floor or wall, a temporary filling, a filled tooth with active decay, or a tooth extracted due to caries, corresponding to ICD‐9 code 521.0 and ICD‐10 codes K02.3–K02.9. Cosmetic defects, enamel stains, non‐cavitated fissures, fluorosis, and abrasion lesions were excluded.

##### 2.2.2.2 Other Oral Conditions

“Other oral conditions” refer to a heterogeneous group of oral health conditions not classified under untreated dental caries (permanent or deciduous), periodontitis, edentulism, or severe tooth loss [11]. This category includes temporomandibular disorders, malocclusion, and dental trauma [13]. The definition was based on required procedures and reasons for dental visits recorded in the U.S. Medical Expenditure Panel Survey (MEPS), a nationally representative survey conducted annually from 1996 to 2011 by the U.S. Agency for Healthcare Research and Quality [11, 13].

### 2.3 Data Presentation

Prevalence, incidence, Years Lived with Disability (YLDs), Years of Life Lost (YLLs), Disability‐Adjusted Life Years (DALYs), and deaths were reported for lip and oral cavity cancer (Level 3). For untreated dental caries in deciduous and permanent teeth (Level 4), estimates were provided for prevalence, incidence, and YLDs. For “other oral conditions” (Level 4), estimates included prevalence and YLDs. Additionally, an aggregated estimate for overall oral conditions (Level 3), encompassing untreated caries, severe periodontitis, edentulism, and “other oral conditions” (excluding lip and oral cavity cancer), was reported for prevalence, incidence, and YLDs.

Data were stratified into five‐year age groups. Lip and oral cavity cancer (Level 3) was analyzed from ages 15–19 through ≥ 95 years. Overall oral conditions (Level 3) and “other oral conditions” (Level 4) were assessed across all age groups, from < 5 years to ≥ 95 years. Untreated caries of deciduous teeth (Level 4) was examined in individuals < 5 to 10–14 years, while untreated caries of permanent teeth (Level 4) was assessed from ages 5–9 to ≥ 95 years. Both absolute numbers (in millions) and age‐standardized estimates (per 100 for prevalence and incidence; per 100 000 for YLDs, YLLs, and DALYs) were provided globally, for seven super‐regions, 21 regions, and 204 countries, according to the GBD hierarchy [12].

Years Lived with Disability (YLDs) and Years of Life Lost (YLLs) are key metrics for assessing disease burden. YLDs quantify the impact of non‐fatal conditions by multiplying disease prevalence by its disability weight, which ranges from 0 (perfect health) to 1 (equivalent to death) [11]. For lip and oral cavity cancer, disability weights varied by stage, from 0.049 [0.031–0.072] in controlled phases to 0.540 [0.377–0.687] in terminal phases with medication. Untreated caries in deciduous and permanent teeth had a disability weight of 0.010 (0.005–0.019), while “other oral conditions” were classified as mild (0.006 [0.002–0.012]) or severe (0.051 [0.032–0.074]) [14]. YLLs measure premature mortality by multiplying the number of deaths by years of life lost. Together, YLDs and YLLs form Disability‐Adjusted Life Years (DALYs), a comprehensive measure of disease burden encompassing both disability and early death. Since there is no mortality attributed to oral conditions, their DALYs equate to YLDs, whereas lip and oral cavity cancer contribute to DALYs through both YLDs and YLLs [11].

### 2.4 Forecasting up to 2050

Forecasting of prevalence, incidence, YLDs, YLLs, DALYs, and deaths for lip and oral cavity cancer, as well as prevalence, incidence, and YLDs for oral conditions up to 2050, was provided *ad hoc* for this manuscript by the 2021 GBD Forecasting Collaborators group. Detailed methods are described elsewhere [15]. In summary, prevalence and incidence were estimated using mixed‐effects models, with the Socio‐demographic Index (SDI) as the primary covariate. The SDI represents a composite measure incorporating lag‐distributed income per capita, average years of education, and total fertility under age 25 [15]. YLDs were derived from these prevalence estimates and the average disability weights from GBD. YLLs and DALYs for lip and oral cavity cancer were calculated by integrating mortality data [15]. Based on YLDs, lip and oral cavity cancer and overall oral conditions (aggregated) were ranked among Level 3 conditions, while disaggregated oral conditions (untreated caries, severe periodontitis, edentulism, and “other oral conditions”) were ranked within Level 4 conditions.

Total percentage change (TPC) and annual percentage change (APC) were reported to illustrate variations from 1990 to 2021 and projections from 2021 to 2050. Statistical analyses for TPC/APC calculations and figure generation were conducted using R (version 4.2.10) and RStudio (version 2024.12.0 + 467).

## 3 Results

### 3.1 Burden of Lip and Oral Cavity Cancer in 2021

**FIGURE 2:**
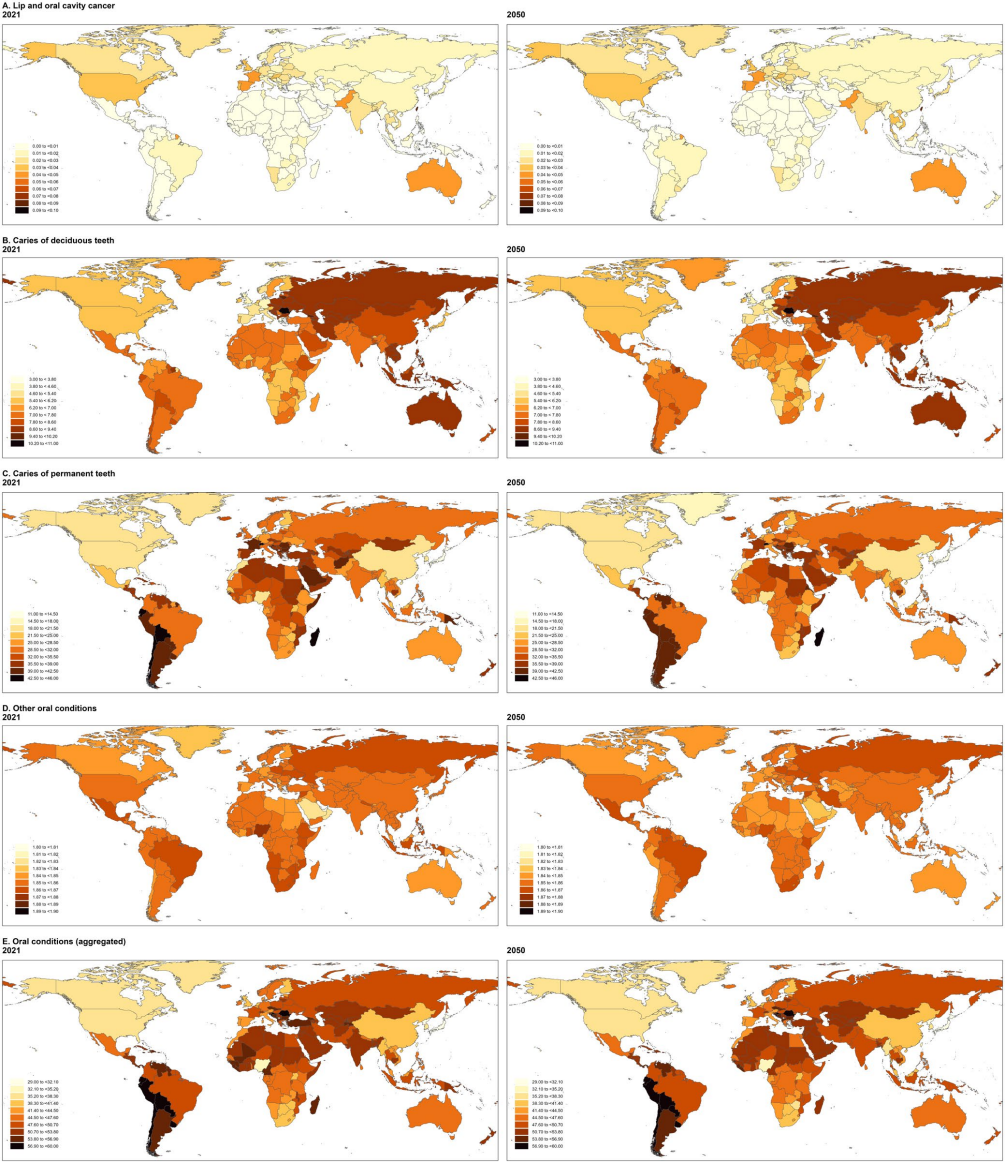
Age‐standardized lip and oral cavity cancer and oral conditions prevalence in 2021 and 2050.

In 2021, over 1.54 million (95% UI 1.44; 1.63) people worldwide were affected by lip and oral cavity cancer, resulting in a global age‐standardized prevalence of 0.02% (Table 1; Figure 2A). Additionally, nearly 0.42 million (0.39; 0.45) new cases were reported.

**TABLE 1:** Global prevalence, incidence and burden of lip and oral cavity cancer in 2021, with projections to 2050.

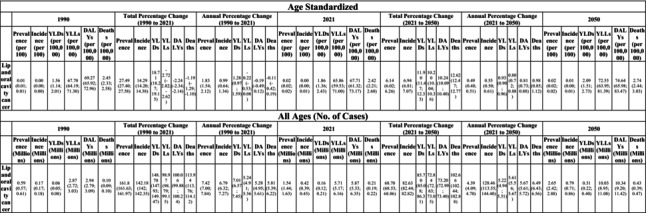

*Note:* Estimates are presented as age‐standardized and all ages values. Age‐standardized estimates are adjusted to a standard global population structure, allowing for comparison across countries and time periods, while all ages estimates reflect the total burden in the population, including all individuals with available data.

**FIGURE 3:**
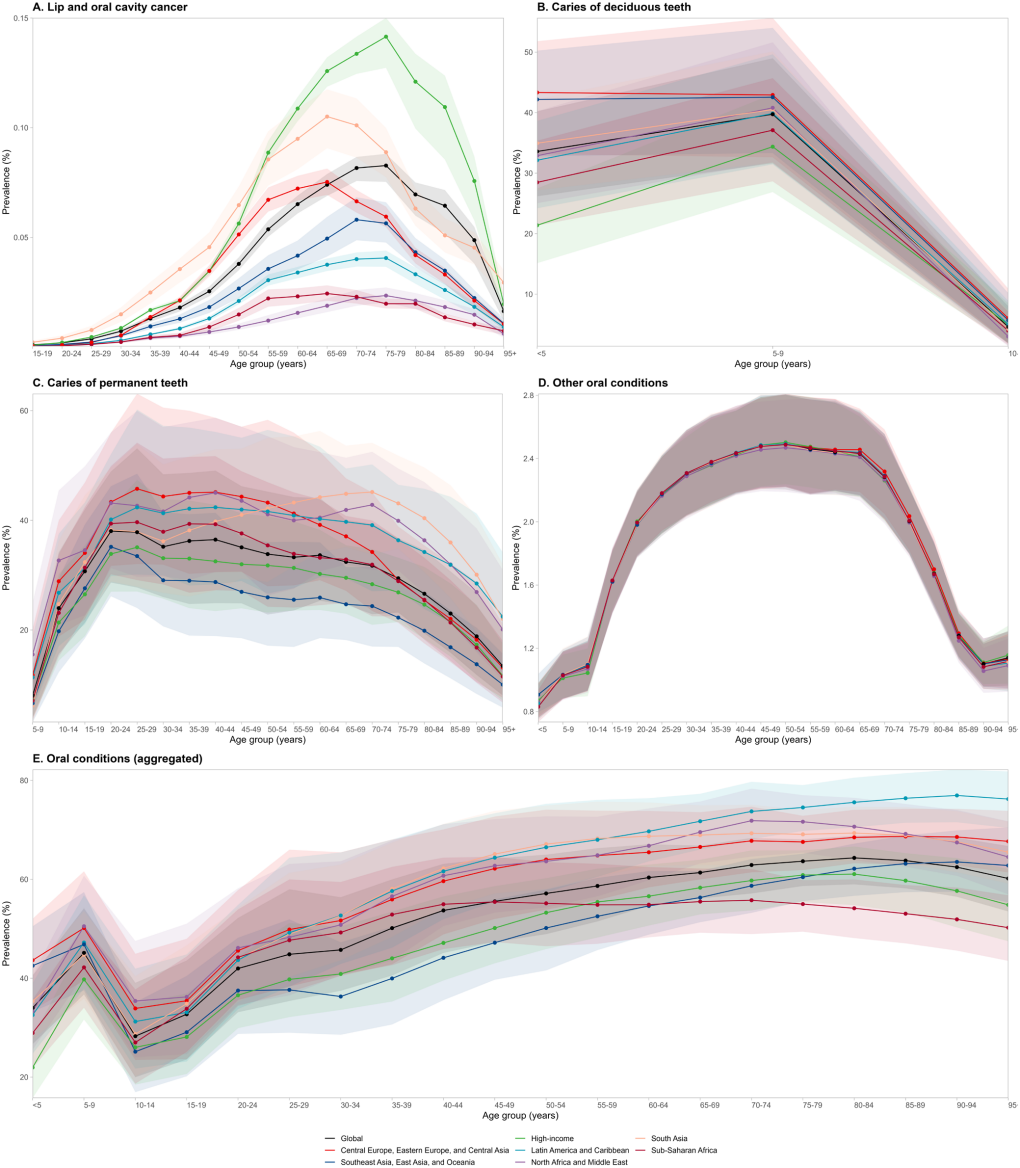
Prevalence of lip and oral cavity cancer (A), caries of deciduous teeth (B), caries of permanent teeth (C), other oral conditions (D), and aggregated oral conditions (E) by age and GBD super‐region in 2021.

Age‐standardized prevalence varied by super‐region, with the High‐income super‐region reporting the highest value at 0.03% (0.03; 0.03), while North Africa and the Middle East had the lowest, at near 0.00% (0.00; 0.01) (Table S1). Nationally, Palau and Taiwan had the highest age‐standardized prevalence, both at 0.09%, while São Tomé and Príncipe reported the lowest, near 0.00%. Regarding sex differences, the prevalence patterns were similar, though values were higher among men at all ages (Figure S1A). The highest prevalence was observed in individuals aged 55–84 years in the High‐income super‐region; beyond this age range, the prevalence declined (Figure 3A).

**FIGURE 4:**
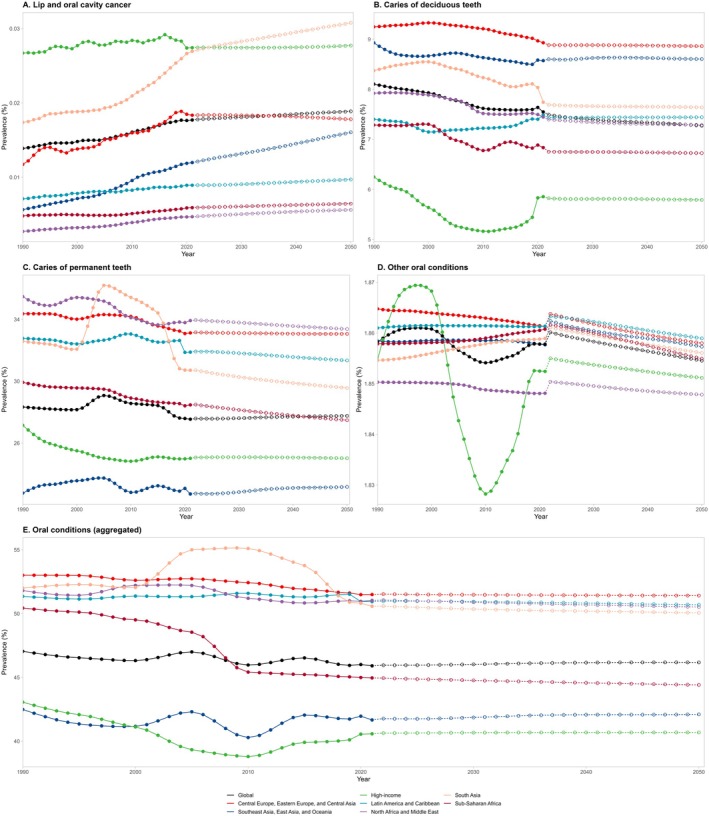
Prevalence of lip and oral cavity cancer (A), caries of deciduous teeth (B), caries of permanent teeth (C), other oral conditions (D) and aggregated oral conditions (E) by GBD super‐region from 1990 to 2050.

**FIGURE 5:**
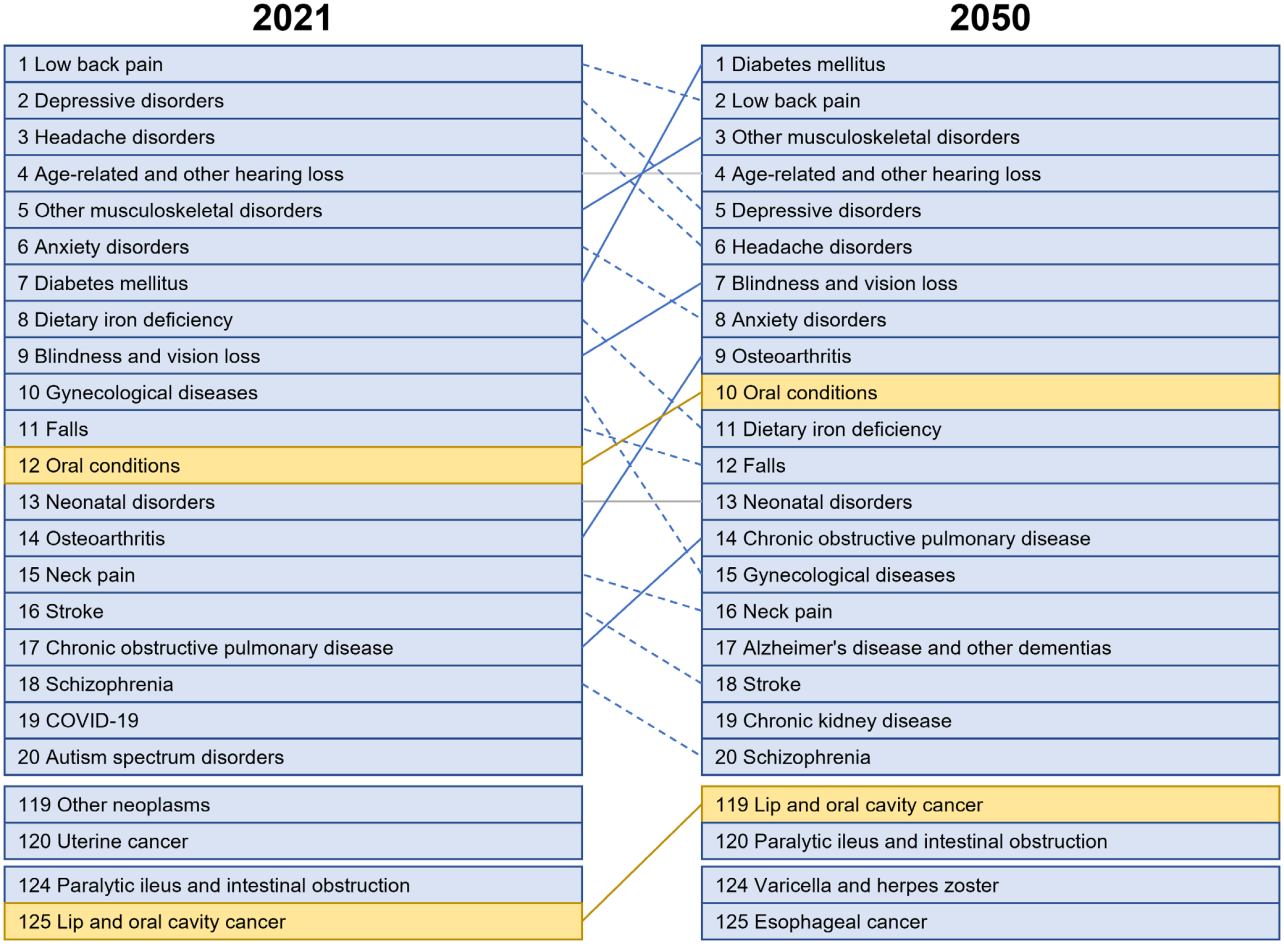
Level 3 leading diseases/conditions in terms of numbers of YLDs affecting humans worldwide in 2021 and 2050. Level 3 includes 175 cause categories, comprising 132 specific diseases and 43 clusters of more detailed Level 4 causes. This level provides a detailed yet aggregated view of disease burden across major conditions (see Figure 1).

The global age‐standardized YLD for lip and oral cavity cancer was 1.28 per 100 000 (0.97; 1.59), corresponding to 0.16 million (0.12; 0.21) YLDs in 2021. The global YLL estimate was 65.86 per 100 000 (59.53; 71.00), accounting for 5.71 million (5.17; 6.16) YLLs. Consequently, the global DALY reached 67.71 per 100 000 (61.32; 73.17), totaling 5.87 million (5.33; 6.35) DALYs. The mortality burden was substantial, with a global death estimate of 2.42 per 100 000 (2.21; 2.60), translating to 0.21 million (0.19; 0.22) deaths (Table 1). Among super‐regions, South Asia recorded the highest values for YLD, YLL, DALY, and deaths (Table S1; Figure 4A). According to the YLD age‐standardized values, lip and oral cavity cancer ranked globally as the 125th most burdensome Level 3 condition in 2021 (Figure 5).

### 3.2 Lip and Oral Cavity Cancer Over Time: 1990–2021, and Forecasts to 2050

Compared to 1990, the total number of prevalent and incident cases of lip and oral cavity cancer has risen substantially, with increases of +161.8% and +142.18%, respectively. However, the age‐standardized prevalence (+27.49%) and incidence (+14.29%) have exhibited more moderate growth (Table 1; Figure S2A).

By 2050, the number of people living with lip and oral cavity cancer is projected to exceed 2.65 million, reflecting an increase of +68.7% and an annual growth rate of +4.39% from 2021 (Table 1, Figure 4A). The incidence is also expected to rise to 0.79 million, representing an +82.63% increase compared to 2021. Meanwhile, the global age‐standardized prevalence (0.02% [0.02; 0.02]) and incidence (0.01% [0.00; 0.01]) are projected to rise by +6.14% and +6.94%, respectively.

Between 2021 and 2050, age‐standardized burden (per 100 000) is projected to increase globally. YLDs are expected to rise by +11.90%, while YLLs will see a +10.20% increase. The DALY burden is anticipated to grow by +10.24%, and deaths are projected to increase by 12.62%. By 2050, lip and oral cavity cancer is expected to rank as the 119th most impactful Level 3 disease/condition in terms of YLD, advancing six positions in the global ranking compared to 2021 (Figure 5).

### 3.3 Burden of Oral Conditions in 2021

#### 3.3.1 Caries in Deciduous Teeth in 2021

In 2021, an estimated 524.63 million (437.68; 611.23) people worldwide were affected by untreated caries in deciduous teeth, corresponding to an age‐standardized prevalence of 7.55% (6.29; 8.78) (Table 2; Figure 2B). The highest prevalence was observed in the Central Europe, Eastern Europe, and Central Asia super‐region, at 8.93% (7.44; 10.59), with Romania recording the highest national prevalence at 10.52% (8.86; 12.26). In contrast, the lowest prevalence was found in the High‐income super‐region, at 5.86% (4.75; 7.13), with Switzerland (3.11% [2.28; 4.37]) reporting the lowest at the country level (Table S2). Globally, the prevalence of untreated caries in deciduous teeth peaked at ages 5–9, affecting more than 40% of children in this age group (Figure 3B).

**TABLE 2:** Global prevalence, incidence and burden of oral conditions in 2021, with projections to 2050.

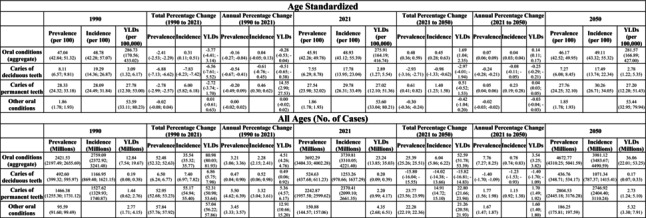

*Note:* Estimates are presented as age‐standardized and all ages values. Age‐standardized estimates are adjusted to a standard global population structure, allowing for comparison across countries and time periods, while all age estimates reflect the total burden in the population, including all individuals with available data.

Approximately 1.25 billion new cases (1253.26 million [978.66; 1637.29]) of untreated caries in deciduous teeth occurred globally, corresponding to an age‐standardized incidence of 17.78% (13.95; 23.04) (Figure S2B). The highest incidence was observed in the Central Europe, Eastern Europe, and Central Asia super‐region, at 20.62% (15.31; 29.35), with Romania again presenting the highest national age‐standardized incidence, at 21.72% (15.51; 31.14). The lowest incidence at the country level was recorded in Denmark (11.39% [6.91; 17.21]) (Table S2).

**FIGURE 6:**
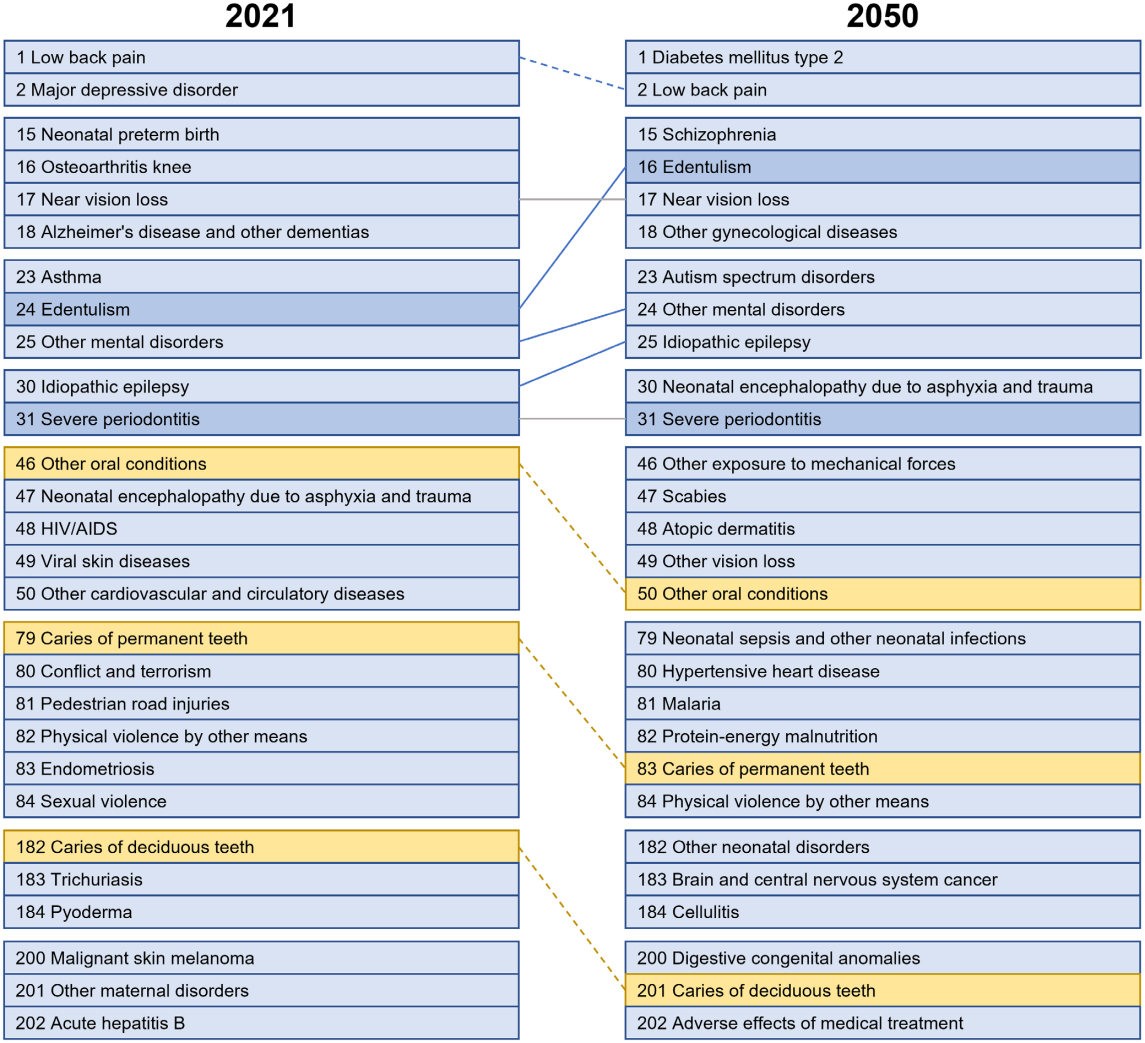
Level 4 leading diseases/conditions in terms of numbers of YLDs affecting humans worldwide in 2021 and 2050. Level 4 represents the most detailed classification of specific causes, nested within broader Level 3 categories. This level provides disaggregated clusters into 302 individual causes (see Figure 1).

Untreated caries in deciduous teeth contributed to 23.24 million YLDs (13.85; 35.03) globally, with an age‐standardized YLD of 2.89 per 100 000 individuals (1.27; 5.54) (Table 2; Figure S3B). Among Level 4 diseases and conditions, untreated caries in deciduous teeth were ranked as the 182nd most impactful condition worldwide (Figure 6).

#### 3.3.2 Caries in Permanent Teeth in 2021

Approximately 2.24 billion (2242.87 million [1957.58; 2599.62]) people worldwide were affected by untreated caries in permanent teeth, resulting in an age‐standardized prevalence of 27.54% (23.98; 32.02) (Table 2; Figure 2C) in 2021. The highest prevalence was observed in the North Africa and Middle East super‐region, at 33.92% (29.42; 39.16), with Madagascar reporting the highest national estimate (45.95% [40.30; 51.55]). In contrast, the lowest prevalence was found in the High‐income super‐region, at 25.01% (22.20; 28.65), with Japan recording the lowest national prevalence at 11.76% (10.21; 13.64) (Table S3). The global prevalence of untreated caries in permanent teeth peaked at 20 years—exceeding 38%, remained above 30% until age 74, and then declined. Notably, the prevalence exceeded 45% in the Central Europe, Eastern Europe, and Central Asia super‐region among individuals aged 25–29, and in the South Asia super‐region among those aged 70–74 (Figure 3C).

Regarding incidence, there were 2.37 billion (2370.41 million [2099.10; 2661.35]) new cases of untreated caries in permanent teeth, resulting in an age‐standardized incidence of 29.78% (26.31; 33.49) (Figure S2C). The highest incidence estimate was recorded in the Latin America and Caribbean super‐region (33.07% [28.97; 37.43]), with Cambodia (37.69% [32.67; 42.77]), Indonesia (36.83% [32.32; 41.42]), and Brazil (36.80% [32.04; 41.66]) having the highest national values. Conversely, the lowest incidence was observed in the Southeast Asia, East Asia, and Oceania super‐region (27.08% [23.53; 30.83]), with Taiwan (13.33% [10.26; 16.27]), the Democratic People's Republic of Korea (14.29% [10.98; 17.84]), and China (23.76% [20.40; 27.68]) standing out with the lowest values at the country level (Table S3).

Globally, untreated caries in permanent teeth contributed to 2.20 million YLDs (0.99; 4.17), with an age‐standardized YLD of 27.02 per 100 000 individuals (12.10; 51.36) (Table 2; Figure S3C). Among Level 4 diseases and conditions, this condition ranked 79th in terms of YLD estimates (Figure 6).

#### 3.3.3 Other Oral Conditions in 2021

In 2021, approximately 150.88 million (144.57; 157.06) people worldwide were affected by “other oral conditions”, with an age‐standardized prevalence of 1.86% (1.78; 1.93). The highest prevalence was observed in Nigeria and Russia, both with a prevalence of 1.87% (1.79; 1.96), while the lowest prevalence was found in the United Arab Emirates (1.80% [1.72; 1.87]) and Qatar (1.80% [1.73; 1.88]) (Table 2; Figure 2D; Table S4).

“Other oral conditions” accounted for 4.35 million YLDs (2.68; 6.51) globally, corresponding to an age‐standardized YLD of 53.60 per 100 000 (33.04; 80.21) (Table 2; Figure S3D). In terms of disease burden, this condition ranked 46th among the Level 4 ones (Figure 6).

#### 3.3.4 Overall Oral Conditions in 2021

In 2021, approximately 3.69 billion people worldwide were affected by at least one oral condition, including caries in deciduous or permanent teeth, severe periodontitis, edentulism, or “other oral conditions” (3692.29 million [3404.33; 4002.28]), resulting in an age‐standardized prevalence of 45.91% (42.26; 49.78) (Table 2; Figure 2E). Among super‐regions, Central Europe, Eastern Europe, and Central Asia exhibited the highest prevalence, at 51.50% (46.99; 56.01). At the national level, Bolivia (59.74% [54.75; 65.01]) reported the highest age‐standardized prevalence. Conversely, the lowest prevalence was observed in High‐income countries (40.58% [37.37; 43.90]), with Japan (29.14% [26.25; 31.76]) and Singapore (32.78% [29.18; 36.60]) having the lowest estimates at the country level (Table S5). Across all GBD super‐regions and both sexes, the age‐standardized prevalence of overall oral conditions increased with age, reaching approximately 80% of females over 80 years old in Southeast Asia, East Asia, and Oceania (Figure 3E; Figure S1E).

In addition, there were 3.74 billion new cases (3739.81 million [3310.05; 4221.40]), resulting in a global age‐standardized incidence of 48.93% (43.12; 55.39) (Table 2; Figure S2D). Indonesia (59.29% [51.45; 69.12]) and Brazil (58.98% [51.01; 69.46]) exhibited the highest incidence values, while the lowest were observed in Taiwan (32.41% [24.91; 42.31]) and the Democratic People's Republic of Korea (34.05% [27.12; 42.19]) (Table S5).

In terms of disease burden, overall oral conditions accounted for 23.24 million YLDs (13.85; 35.03) globally in 2021, with an age‐standardized YLD of 275.91 per 100 000 people (164.19; 416.74) (Table 2; Figure S3E). At the country level, Bolivia recorded the highest YLD, at 459.45 per 100 000 (288.53; 668.12), while Nigeria had the lowest, at 166.88 per 100 000 (100.90; 253.85) (Table S5). Among Level 3 conditions, overall oral conditions ranked as the 12th most impactful one worldwide in terms of disease burden (Figure 5).

### 3.4 Oral Conditions Over Time: 1990 to 2021, and Forecasts to 2050

#### 3.4.1 Caries in Deciduous Teeth Over Time

From 1990 to 2021, both the prevalence and incidence of untreated caries in deciduous teeth showed a significant decline. The total percentage change in prevalence decreased by −6.88%, with an annual reduction of −0.54%. Similarly, the incidence decreased by −7.83%, reflecting an annual decline of −0.61% (Table 2; Figure 4B; Figure S2B).

The global prevalence of untreated caries in deciduous teeth is projected to continue declining through 2050, reaching approximately 436.76 million (348.71; 534.17) cases, representing a reduction of −15.80% compared to 2021, with an annual decrease of −1.40%. The number of new cases is also expected to decline, with an estimated 1.07 billion (1071.34 million [787.37; 1415.41]) cases, reflecting a − 14.02% decrease from 2021, at an annual rate of −1.23% (Table S2).

Regarding YLDs due to untreated caries in deciduous teeth, a reduction of −15.82% is projected by 2050. The YLD is expected to be 2.78 per 100 000 people (1.22; 5.35) (Table 2; Figure S3B), with untreated caries in deciduous teeth ranking 201st among Level 4 diseases, a decrease of 19 positions compared to 2021.

#### 3.4.2 Caries in Permanent Teeth Over Time

Between 1990 and 2021, the prevalence of untreated caries in permanent teeth experienced a modest decline, with a total decrease of −2.78% and an annual change of −0.20%, remaining relatively stable over time. In contrast, the incidence increased by +6.00%, with an annual rise of +0.46% (Table 2, Figure 4C; Figure S2C).

By 2050, the global number of prevalent cases of untreated caries in permanent teeth is projected to reach approximately 2.8 billion (2804.53 million [2445.18; 3176.28]), reflecting a + 23.77% increase compared to 2021, with an annual growth rate of +1.77%. Similarly, the incidence is expected to reach 2.75 billion (2746.92 million [2404.40; 3110.24]), representing a + 14.91% increase from 2021, with an annual change of +1.15% (Table 2; Figure 4C; Figure S2C).

The YLD due to untreated caries in permanent teeth is anticipated to rise by +22.80% by 2050. The YLD is projected to be 27.20 per 100 000 (12.20; 51.43) (Table 2; Figure S3C). Untreated caries in permanent teeth is expected to rank 83rd among Level 3 diseases/conditions in 2050, a decrease of four positions compared to 2021 (Figure 6).

#### 3.4.3 Other Oral Conditions Over Time

The prevalence of “other oral conditions” remained stable between 1990 and 2021, with a negligible total percentage change of −0.02% and an annual variation of 0.00%. Similarly, the YLD per 100 000 people exhibited minimal change, with a total percentage shift of 0.01% and an annual variation, near 0.00% (Table 2; Figure 4D; Figure S3D).

By 2050, the number of individuals affected by “other oral conditions” is expected to reach approximately 186.25 million (175.81; 197.59), reflecting a + 22.28% increase compared to 2021, with an annual growth rate of +1.67% (Table S4). The YLD burden is also projected to rise, reaching an estimated 5.32 million (3.30; 7.91), representing a + 21.26% increase from 2021. The age‐standardized YLD is anticipated to reach 53.44 per 100 000 people (32.95; 79.94) by 2050 (Table 2; Figure S3D). Despite this increase, “other oral conditions” are expected to drop four positions in the Level 3 disease ranking, reaching the 50th position by 2050 (Figure 6).

#### 3.4.4 Overall Oral Conditions Over Time

There was a slight decrease in the global prevalence of oral conditions (−2.41%), while the incidence showed a modest increase (+0.31%) compared to 1990 (Table 2; Figure 4E; Figure S2D). The annual percentage change in prevalence was −0.16%, reflecting a slow but steady decline, while the incidence remained relatively stable, with an annual change of +0.04%.

Looking ahead to 2050, the global prevalence of oral conditions is projected to reach 4.47 billion (4672.77 million [4310.25; 5041.59]), representing an increase of over +25.39% compared to 2021, with an annual growth rate of +7.76% (Table 2; Figure 4E; Figure S2D). Similarly, the number of new cases is expected to rise to approximately 4 billion (3981.12 million [3483.67; 4490.59]), reflecting a +6.04% increase compared to 2021, with an annual rise of +0.78%. The countries with the highest age‐standardized incidence are projected to be the Republic of Korea (59.71 [51.58; 70.39]) and Brazil (59.01 [50.97; 69.56]) (Table 2; Table S5).

The number of YLDs due to oral conditions is anticipated to rise significantly by 2050, with a total increase of +52.59% compared to 2021. By then, the global YLD is expected to reach 281.57 per 100 000 people (Table 2; Figure S3E), positioning overall oral conditions as the 10th most burdensome condition among Level 3 diseases, rising two positions from 2021 (Figure 5), surpassing conditions such as stroke and Alzheimer's disease.

## 4 Discussion

Our findings provide compelling evidence of the substantial global burden of lip and oral cavity cancer and oral conditions. In 2021, lip and oral cavity cancer was more prevalent among men, particularly those aged 55 to 84 in high‐income regions, while overall oral conditions showed an increasing prevalence with age, affecting nearly 80% of women over 80 years old in Southeast Asia, East Asia, and Oceania. Over the past three decades, cases of lip and oral cavity cancer have more than doubled, and by 2050, they are projected to exceed 2.65 million, with new cases increasing by 82.63%. Although the prevalence of overall oral conditions slightly declined between 1990 and 2021, their total number is expected to rise to 4.47 billion by 2050, reflecting a 25.39% increase, while the YLD burden is projected to grow by 52.59%, largely driven by edentulism and periodontitis, positioning oral conditions among the top 10 Level 3 conditions in global burden.

The global burden of lip and oral cavity cancer and oral conditions may align with trends in non‐communicable diseases (NCDs) and shared behavioral and metabolic risk factors. While oral cancers are less prevalent, their projected rise to over 2.65 million cases by 2050 could reflect persistent tobacco and alcohol use, factors also implicated in cardiovascular and other chronic conditions [16]. Similarly, oral conditions, affecting nearly half the global population, appear to parallel NCD trajectories [17, 18], with added sugars [19], smoking [20], obesity [21], and hyperglycemia [22] potentially contributing to both oral and systemic chronic diseases. This pattern reinforces evidence suggesting a convergence between caries and periodontitis [5, 6, 7, 8], both of which may precede or signal cumulative risks for NCDs later in adult life. This trajectory may explain why these conditions currently rank as the 12th leading cause of YLDs, a measure of non‐fatal health loss, and are projected to become the 10th by 2050—driven predominantly by the persistent, prevalent, and disabling nature of edentulism and periodontitis—surpassing conditions such as stroke, Alzheimer's disease, and chronic obstructive pulmonary disease in terms of disability burden at the population level.

This study reveals the increasing burden of lip and oral cavity cancer, in line with previous findings [23]. In 2021, certain high‐burden regions exhibited the highest oral cancer prevalence and incidence, likely driven by behavioral risk factors such as chewing tobacco [24] and betel‐based product use [25], with South Asia projected to surpass high‐income countries in prevalence by 2050. Caution is warranted when interpreting these data, as estimates from smaller countries, like Palau, may rely on imputed values rather than country‐specific studies. Lip cancer, strongly associated with sun exposure, may be further exacerbated by climate change and ozone depletion in regions with insufficient atmospheric protection [26]. Additionally, the growing role of HPV in oropharyngeal cancer underscores the potential of HPV vaccination as an effective strategy to mitigate the cancer burden [27].

Andean Latin American countries show notably high oral condition prevalence and YLD values, potentially linked to socioeconomic barriers to care [28, 29], while diets high in added sugars [30] and uneven fluoridation policies may contribute to the caries burden [31]. Projections suggest a rising oral condition incidence in Brazil and the Republic of Korea by 2050, possibly influenced by demographic and lifestyle shifts [32, 33]—though Brazil's elevated edentulism rates [9] raise questions about healthcare access. Additionally, Romania and Madagascar report the highest prevalence of caries in deciduous and permanent teeth, respectively, with limited fluoride availability [34] and weaker regulation of taxing sugar‐sweetened beverages [35] possibly contributing to these trends. Despite experiencing a modest decline from 1990 to 2021, overall oral condition prevalence is projected to rise sharply by 2050, largely driven by increasing estimates of edentulism and severe periodontitis, as shown by Nascimento et al. [9], underscoring the urgency of strengthened prevention and control efforts.

Socioeconomic disparities critically shape oral health outcomes in low‐ and middle‐income countries, where limited access to nutritious diets, hygiene, and care amplifies the burden of oral conditions [36]. In many African, Latin American, and Asian countries with limited healthcare resources, dental caries and periodontitis remain highly prevalent, contributing substantially to years lived with disability. Paradoxically, some oral conditions are more prevalent in high‐income countries, likely due to better diagnostic capacity, lifestyle‐related risk factors, and persistent inequalities affecting marginalized urban populations [37]. Emerging trends such as increased e‐cigarette use, despite concerns about its potential harm to oral health [38], and declining alcohol consumption among younger populations [39] may also influence future patterns. Without targeted, context‐specific interventions, the burden of oral conditions, including oral cancer, is expected to rise, deepening global health disparities.

Interpreting these findings requires acknowledging several limitations. GBD estimates may be subject to selection bias, as disadvantaged populations are often underrepresented in studies [40]. Notably, data on “other oral conditions” are only available for the United States, derived from the Medical Expenditure Panel Survey [11]. This limited data availability may partly account for discrepancies observed in estimates for the High‐income super‐region. Additionally, forecasts relied solely on the sociodemographic index [15], excluding key predictors such as sugar consumption, smoking, and taxation policies. Lastly, the GBD disability weights likely underestimate the true burden of oral conditions by overlooking their impacts on phonation, social functioning, and systemic health [11, 14], suggesting that the actual burden may be even greater than estimated.

## 5 Conclusions

### 5.1 Implications for Clinical Practice

Our findings confirm that oral cancer and oral conditions remain a significant public health challenge, underscoring the urgent need to integrate oral health into broader noncommunicable disease prevention strategies. Oral conditions aggregated (caries, periodontitis, edentulism, “other oral conditions”), currently the 12th most burdensome health condition, are projected to rise to the 10th position globally, fueled by the rise in edentulism and periodontitis prevalence. This underscores the urgent need to expand targeted screening and early intervention programs. To effectively address these rising trends, clinicians should prioritize high‐risk populations and invest in evidence‐based preventive strategies.

### 5.2 Implications for Research

The escalating impact of oral conditions calls for further research to unravel the complex interplay between behavioral and metabolic shared risk factors in the progression of both oral and systemic conditions. This includes addressing current epidemiological gaps, particularly the limited availability of data in low‐income regions. Future studies should focus on refining intervention strategies, assessing the long‐term benefits of preventive care, and developing innovative approaches to reduce disparities in oral health outcomes.

### 5.3 Implications for Policy

Our findings expose a critical shortfall: despite decades of global health initiatives, progress in reducing the burden of lip and oral cavity cancer and oral conditions (caries, periodontitis, edentulism, “other oral conditions”) has been negligible. The 2021 data clearly demonstrate that, in spite of extensive international efforts, prevailing trends have scarcely shifted over the past three decades, while projections for 2050 are even more disquieting. This highlights the need for targeted interventions. Integrating oral health into NCD strategies, expanding screening for high‐risk groups, and engaging dental institutions in guideline development, policy advocacy, and professional training are essential. A coordinated effort is crucial to curb this growing burden.

## Author Contributions

Silas Alves‐Costa contributed to data analysis and manuscript drafting. Mario Romandini contributed to study conception, data acquisition and interpretation, and critically revised the manuscript. Gustavo G. Nascimento contributed to data interpretation and critically revised the manuscript.

## Conflicts of Interest

The authors declare no conflicts of interest.

## Supporting information


**Data S1.** Supporting Information.

## Data Availability

The data that support the findings of this study are available in Global Health Data Exchange at https://ghdx.healthdata.org/. These data were derived from the following resources available in the public domain: ‐ GBD Results, https://vizhub.healthdata.org/gbd‐results/‐ GBD Foresight, https://vizhub.healthdata.org/gbd‐foresight/.
