# Supplementary material for: Lip and Oral Cancer, Caries and Other Oral Conditions: Estimates From the 2021 Global Burden of Disease Study and Projections up to 2050
Source: J Periodontal Res. 2025 Jun 18;60(6):544–58. doi: 10.1111/jre.13421 (PMC12312818; doi:10.1111/jre.13421)
Supplement: Supplementary file 1 — Data S1. Supporting Information. [file JRE-60-544-s001.docx]

**Appendix**

**Appendix Table 1.** Prevalence, incidence and burden of lip and oral cavity cancer in 2021, with projections to 2050, in 21 GBD regions and 204 countries.

| **LIP AND ORAL CAVITY CANCER** | | | | | | | | | | | | | | | | | | | | | | | | | | | | | | | | | | | | |
| --- | --- | --- | --- | --- | --- | --- | --- | --- | --- | --- | --- | --- | --- | --- | --- | --- | --- | --- | --- | --- | --- | --- | --- | --- | --- | --- | --- | --- | --- | --- | --- | --- | --- | --- | --- | --- |
| **Location** | **Age-standardized** | | | | | | | | | | | | | | | | | | **All Ages (nº of cases)** | | | | | | | | | | | | | | | | | |
|  | **Prevalence (%)** | | | **Incidence (%)** | | | **YLDs (per 100,000)** | | | **YLLs (per 100,000)** | | | **DALYs (per 100,000)** | | | **Deaths (per 100,000)** | | | **Prevalence (Millions)** | | | **Incidence (Millions)** | | | **YLDs (Millions)** | | | **YLLs (Millions)** | | | **DALYs (Millions)** | | | **Deaths (Millions)** | | |
|  | **2021** | **2050** | **2021-2050 TPC (%)** | **2021** | **2050** | **2021-2050 TPC (%)** | **2021** | **2050** | **2021-2050 TPC (%)** | **2021** | **2050** | **2021-2050 TPC (%)** | **2021** | **2050** | **2021-2050 TPC (%)** | **2021** | **2050** | **2021-2050 TPC (%)** | **2021** | **2050** | **2021-2050 TPC (%)** | **2021** | **2050** | **2021-2050 TPC (%)** | **2021** | **2050** | **2021-2050 TPC (%)** | **2021** | **2050** | **2021-2050 TPC (%)** | **2021** | **2050** | **2021-2050 TPC (%)** | **2021** | **2050** | **2021-2050 TPC (%)** |
| **Global** | 0.02 (0.02; 0.02) | 0.02 (0.02; 0.02) | 6.14 (6.02; 6.26) | 0.00 (0.00; 0.01) | 0.01 (0.00; 0.01) | 6.94 (6.81; 7.07) | 1.86 (1.36; 2.43) | 2.09 (1.51; 2.73) | 11.90 (11.47; 12.33) | 65.86 (59.53; 71.00) | 72.55 (63.95; 81.39) | 10.20 (10.04; 10.36) | 67.71 (61.32; 73.17) | 74.64 (65.98; 83.47) | 10.24 (10.09; 10.40) | 2.42 (2.21; 2.60) | 2.74 (2.44; 3.03) | 12.62 (12.47; 12.77) | 1.54 (1.44; 1.63) | 2.65 (2.42; 2.88) | 68.70 (68.53; 68.86) | 0.42 (0.39; 0.45) | 0.79 (0.71; 0.86) | 82.63 (82.44; 82.82) | 0.16 (0.12; 0.21) | 0.31 (0.22; 0.40) | 85.70 (85.08; 86.31) | 5.71 (5.17; 6.16) | 10.03 (8.95; 11.08) | 72.84 (72.63; 73.05) | 5.87 (5.33; 6.35) | 10.34 (9.20; 11.42) | 73.20 (72.99; 73.40) | 0.21 (0.19; 0.22) | 0.43 (0.39; 0.47) | 102.66 (102.44; 102.88) |
| **Central Europe, Eastern Europe, and Central Asia** | 0.02 (0.02; 0.02) | 0.02 (0.02; 0.02) | -3.02 (-3.13; -2.90) | 0.01 (0.01; 0.01) | 0.01 (0.00; 0.01) | -5.73 (-5.85; -5.61) | 2.03 (1.49; 2.65) | 2.01 (1.43; 2.65) | -1.52 (-1.93; -1.10) | 73.46 (68.49; 78.36) | 66.69 (60.05; 74.08) | -8.97 (-9.09; -8.85) | 75.49 (70.14; 80.50) | 68.70 (61.87; 76.36) | -8.77 (-8.89; -8.65) | 2.57 (2.41; 2.74) | 2.47 (2.24; 2.73) | -4.23 (-4.34; -4.11) | 0.11 (0.10; 0.12) | 0.12 (0.11; 0.13) | 10.32 (10.19; 10.46) | 0.03 (0.03; 0.04) | 0.04 (0.04; 0.04) | 14.67 (14.52; 14.82) | 0.01 (0.01; 0.02) | 0.01 (0.01; 0.02) | 17.10 (16.66; 17.55) | 0.45 (0.42; 0.48) | 0.46 (0.42; 0.51) | 4.73 (4.59; 4.86) | 0.46 (0.43; 0.49) | 0.48 (0.44; 0.53) | 5.07 (4.93; 5.20) | 0.02 (0.02; 0.02) | 0.02 (0.02; 0.02) | 26.61 (26.46; 26.76) |
| **Central Asia** | 0.01 (0.01; 0.01) | 0.01 (0.01; 0.01) | 5.46 (5.23; 5.69) | 0.00 (0.00; 0.00) | 0.00 (0.00; 0.00) | 2.46 (2.24; 2.69) | 0.89 (0.63; 1.20) | 0.96 (0.66; 1.33) | 7.91 (7.42; 8.40) | 41.37 (36.05; 47.51) | 40.41 (33.49; 48.03) | -2.06 (-2.28; -1.84) | 42.26 (36.89; 48.31) | 41.37 (34.36; 49.02) | -1.85 (-2.06; -1.63) | 1.56 (1.36; 1.78) | 1.57 (1.31; 1.84) | 0.59 (0.38; 0.80) | 0.01 (0.01; 0.01) | 0.01 (0.01; 0.02) | 85.16 (84.79; 85.53) | 0.00 (0.00; 0.00) | 0.00 (0.00; 0.01) | 101.69 (101.28; 102.09) | 0.00 (0.00; 0.00) | 0.00 (0.00; 0.00) | 105.08 (104.30; 105.86) | 0.04 (0.03; 0.04) | 0.07 (0.05; 0.08) | 73.84 (73.47; 74.21) | 0.04 (0.03; 0.04) | 0.07 (0.06; 0.08) | 74.48 (74.11; 74.85) | 0.00 (0.00; 0.00) | 0.00 (0.00; 0.00) | 111.97 (111.58; 112.36) |
| **Armenia** | 0.01 (0.01; 0.01) | 0.01 (0.01; 0.01) | 9.33 (9.02; 9.64) | 0.00 (0.00; 0.00) | 0.00 (0.00; 0.00) | 4.00 (3.70; 4.30) | 0.73 (0.49; 0.98) | 0.81 (0.54; 1.11) | 9.80 (9.28; 10.32) | 28.43 (23.71; 33.75) | 28.04 (21.50; 35.10) | -0.72 (-1.03; -0.42) | 29.15 (24.16; 34.35) | 28.84 (22.29; 36.05) | -0.46 (-0.76; -0.16) | 1.14 (0.95; 1.34) | 1.14 (0.89; 1.42) | 0.43 (0.14; 0.73) | 0.00 (0.00; 0.00) | 0.00 (0.00; 0.00) | 35.64 (35.22; 36.05) | 0.00 (0.00; 0.00) | 0.00 (0.00; 0.00) | 44.31 (43.89; 44.74) | 0.00 (0.00; 0.00) | 0.00 (0.00; 0.00) | 46.99 (46.32; 47.67) | 0.00 (0.00; 0.00) | 0.00 (0.00; 0.00) | 25.05 (24.67; 25.42) | 0.00 (0.00; 0.00) | 0.00 (0.00; 0.00) | 25.61 (25.24; 25.98) | 0.00 (0.00; 0.00) | 0.00 (0.00; 0.00) | 52.90 (52.48; 53.33) |
| **Azerbaijan** | 0.00 (0.00; 0.00) | 0.00 (0.00; 0.01) | 23.40 (22.73; 24.08) | 0.00 (0.00; 0.00) | 0.00 (0.00; 0.00) | 17.77 (17.15; 18.40) | 0.40 (0.24; 0.64) | 0.51 (0.31; 0.83) | 25.86 (25.05; 26.66) | 18.17 (11.63; 27.54) | 20.08 (12.49; 30.15) | 10.54 (9.90; 11.18) | 18.57 (11.93; 27.90) | 20.60 (13.14; 30.63) | 10.88 (10.25; 11.50) | 0.70 (0.48; 1.03) | 0.80 (0.54; 1.16) | 13.43 (12.86; 14) | 0.00 (0.00; 0.00) | 0.00 (0.00; 0.00) | 116.28 (115.31; 117.26) | 0.00 (0.00; 0.00) | 0.00 (0.00; 0.00) | 135.29 (134.30; 136.28) | 0.00 (0.00; 0.00) | 0.00 (0.00; 0.00) | 142.57 (141.24; 143.90) | 0.00 (0.00; 0.00) | 0.00 (0.00; 0.01) | 96.34 (95.45; 97.24) | 0.00 (0.00; 0.00) | 0.00 (0.00; 0.01) | 97.31 (96.43; 98.19) | 0.00 (0.00; 0.00) | 0.00 (0.00; 0.00) | 144.43 (143.48; 145.39) |
| **Georgia** | 0.01 (0.01; 0.01) | 0.01 (0.01; 0.02) | 17.36 (17.09; 17.64) | 0.00 (0.00; 0.00) | 0.00 (0.00; 0.01) | 21.46 (21.17; 21.74) | 1.29 (0.90; 1.71) | 1.54 (1.07; 2.14) | 18.23 (17.71; 18.74) | 60.73 (53.06; 69.17) | 75.84 (61.38; 94.43) | 23.20 (22.90; 23.49) | 62.02 (53.83; 70.73) | 77.38 (62.77; 96.37) | 23.09 (22.80; 23.39) | 2.27 (1.98; 2.58) | 2.86 (2.37; 3.50) | 24.12 (23.85; 24.40) | 0.00 (0.00; 0.00) | 0.00 (0.00; 0.00) | 19.54 (19.19; 19.89) | 0.00 (0.00; 0.00) | 0.00 (0.00; 0.00) | 31.90 (31.53; 32.28) | 0.00 (0.00; 0.00) | 0.00 (0.00; 0.00) | 24.81 (24.23; 25.39) | 0.00 (0.00; 0.00) | 0.00 (0.00; 0.01) | 25.49 (25.13; 25.84) | 0.00 (0.00; 0.00) | 0.00 (0.00; 0.01) | 25.47 (25.12; 25.83) | 0.00 (0.00; 0.00) | 0.00 (0.00; 0.00) | 40.65 (40.28; 41.02) |
| **Kazakhstan** | 0.01 (0.01; 0.01) | 0.01 (0.01; 0.01) | 0.85 (0.56; 1.14) | 0.00 (0.00; 0.00) | 0.00 (0.00; 0.00) | -4.85 (-5.13; -4.56) | 1.25 (0.88; 1.79) | 1.28 (0.88; 1.84) | 2.18 (1.66; 2.71) | 52.76 (43.96; 63.67) | 45.51 (35.12; 57.37) | -13.49 (-13.77; -13.21) | 54.02 (44.68; 64.33) | 46.79 (36.28; 58.64) | -13.12 (-13.39; -12.85) | 1.98 (1.66; 2.37) | 1.81 (1.42; 2.25) | -8.62 (-8.89; -8.35) | 0.00 (0.00; 0.00) | 0.00 (0.00; 0.00) | 58.55 (58.11; 59) | 0.00 (0.00; 0.00) | 0.00 (0.00; 0.00) | 66.13 (65.68; 66.58) | 0.00 (0.00; 0.00) | 0.00 (0.00; 0.00) | 72.17 (71.40; 72.93) | 0.01 (0.01; 0.01) | 0.01 (0.01; 0.02) | 36.90 (36.51; 37.29) | 0.01 (0.01; 0.01) | 0.01 (0.01; 0.02) | 37.71 (37.32; 38.09) | 0.00 (0.00; 0.00) | 0.00 (0.00; 0.00) | 71.88 (71.44; 72.33) |
| **Kyrgyzstan** | 0.01 (0.01; 0.01) | 0.01 (0.01; 0.01) | 29.43 (29.02; 29.84) | 0.00 (0.00; 0.00) | 0.00 (0.00; 0.00) | 19.29 (18.91; 19.66) | 0.93 (0.65; 1.31) | 1.25 (0.87; 1.82) | 32.32 (31.71; 32.94) | 43.15 (35.05; 52.46) | 46.43 (35.79; 60.01) | 7.65 (7.31; 8) | 44.08 (36.46; 53.81) | 47.69 (36.94; 61.27) | 8.18 (7.85; 8.52) | 1.61 (1.32; 1.95) | 1.85 (1.44; 2.37) | 14.02 (13.67; 14.37) | 0.00 (0.00; 0.00) | 0.00 (0.00; 0.00) | 143.69 (142.91; 144.48) | 0.00 (0.00; 0.00) | 0.00 (0.00; 0.00) | 149.24 (148.43; 150.04) | 0.00 (0.00; 0.00) | 0.00 (0.00; 0.00) | 168.52 (167.35; 169.68) | 0.00 (0.00; 0.00) | 0.00 (0.00; 0.01) | 103.96 (103.30; 104.61) | 0.00 (0.00; 0.00) | 0.01 (0.00; 0.01) | 105.29 (104.64; 105.93) | 0.00 (0.00; 0.00) | 0.00 (0.00; 0.00) | 155.38 (154.63; 156.13) |
| **Mongolia** | 0.01 (0.00; 0.01) | 0.01 (0.01; 0.01) | 59.46 (58.80; 60.13) | 0.00 (0.00; 0.00) | 0.00 (0.00; 0.01) | 44.11 (43.49; 44.73) | 0.86 (0.54; 1.26) | 1.40 (0.87; 2.08) | 61.29 (60.48; 62.10) | 45.83 (31.82; 61.49) | 63.57 (43.09; 87.02) | 37.79 (37.22; 38.36) | 46.70 (33.00; 62.20) | 64.97 (44.34; 88.45) | 38.22 (37.66; 38.79) | 1.72 (1.18; 2.34) | 2.38 (1.59; 3.29) | 36.65 (36.06; 37.23) | 0.00 (0.00; 0.00) | 0.00 (0.00; 0.00) | 228.56 (227.33; 229.79) | 0.00 (0.00; 0.00) | 0.00 (0.00; 0.00) | 234.40 (233.12; 235.68) | 0.00 (0.00; 0.00) | 0.00 (0.00; 0.00) | 262.50 (260.85; 264.14) | 0.00 (0.00; 0.00) | 0.00 (0.00; 0.01) | 183.59 (182.54; 184.63) | 0.00 (0.00; 0.00) | 0.00 (0.00; 0.01) | 184.96 (183.92; 185.99) | 0.00 (0.00; 0.00) | 0.00 (0.00; 0.00) | 238.61 (237.37; 239.85) |
| **Tajikistan** | 0.00 (0.00; 0.00) | 0.00 (0.00; 0.00) | 4.08 (3.59; 4.58) | 0.00 (0.00; 0.00) | 0.00 (0.00; 0.00) | -1.70 (-2.16; -1.24) | 0.30 (0.19; 0.45) | 0.32 (0.20; 0.49) | 5.37 (4.72; 6.02) | 16.49 (11.66; 22.59) | 14.82 (9.82; 20.77) | -9.21 (-9.67; -8.74) | 16.78 (12.02; 22.70) | 15.13 (10.10; 21.13) | -8.94 (-9.40; -8.48) | 0.64 (0.47; 0.86) | 0.61 (0.42; 0.84) | -5.95 (-6.38; -5.51) | 0.00 (0.00; 0.00) | 0.00 (0.00; 0.00) | 138.73 (137.74; 139.71) | 0.00 (0.00; 0.00) | 0.00 (0.00; 0.00) | 162.60 (161.54; 163.65) | 0.00 (0.00; 0.00) | 0.00 (0.00; 0.00) | 170.79 (169.42; 172.15) | 0.00 (0.00; 0.00) | 0.00 (0.00; 0.00) | 116.05 (115.18; 116.91) | 0.00 (0.00; 0.00) | 0.00 (0.00; 0.00) | 116.97 (116.13; 117.82) | 0.00 (0.00; 0.00) | 0.00 (0.00; 0.00) | 170.98 (170.03; 171.92) |
| **Turkmenistan** | 0.01 (0.01; 0.01) | 0.01 (0.01; 0.02) | 15.30 (14.82; 15.78) | 0.00 (0.00; 0.00) | 0.00 (0.00; 0.01) | 8.81 (8.37; 9.26) | 1.23 (0.82; 1.81) | 1.45 (0.96; 2.13) | 16.83 (16.20; 17.45) | 64.66 (48.66; 86.01) | 65.11 (46.75; 90.06) | 0.80 (0.36; 1.24) | 65.88 (49.88; 88.57) | 66.56 (48.12; 91.69) | 1.10 (0.67; 1.53) | 2.31 (1.76; 3.05) | 2.42 (1.74; 3.33) | 4.33 (3.89; 4.76) | 0.00 (0.00; 0.00) | 0.00 (0.00; 0.00) | 100.62 (99.88; 101.36) | 0.00 (0.00; 0.00) | 0.00 (0.00; 0.00) | 111.11 (110.35; 111.87) | 0.00 (0.00; 0.00) | 0.00 (0.00; 0.00) | 118.65 (117.65; 119.64) | 0.00 (0.00; 0.00) | 0.01 (0.00; 0.01) | 77.83 (77.23; 78.43) | 0.00 (0.00; 0.00) | 0.01 (0.00; 0.01) | 78.57 (77.98; 79.16) | 0.00 (0.00; 0.00) | 0.00 (0.00; 0.00) | 115.36 (114.73; 115.99) |
| **Uzbekistan** | 0.01 (0.01; 0.01) | 0.01 (0.01; 0.01) | 7.35 (6.98; 7.72) | 0.00 (0.00; 0.00) | 0.00 (0.00; 0.00) | 6.16 (5.80; 6.53) | 0.84 (0.57; 1.18) | 0.92 (0.58; 1.33) | 8.81 (8.23; 9.38) | 42.34 (33.50; 52.66) | 42.94 (32.30; 55.61) | 1.63 (1.28; 1.98) | 43.19 (34.25; 53.50) | 43.86 (33.28; 56.80) | 1.77 (1.42; 2.11) | 1.57 (1.25; 1.96) | 1.65 (1.25; 2.12) | 4.45 (4.10; 4.80) | 0.00 (0.00; 0.00) | 0.00 (0.00; 0.01) | 99.63 (98.81; 100.45) | 0.00 (0.00; 0.00) | 0.00 (0.00; 0.00) | 128.36 (127.45; 129.26) | 0.00 (0.00; 0.00) | 0.00 (0.00; 0.00) | 125.19 (124.03; 126.36) | 0.01 (0.01; 0.02) | 0.03 (0.02; 0.04) | 95.17 (94.33; 96.01) | 0.01 (0.01; 0.02) | 0.03 (0.02; 0.04) | 95.74 (94.90; 96.57) | 0.00 (0.00; 0.00) | 0.00 (0.00; 0.00) | 142.09 (141.11; 143.07) |
| **Central Europe** | 0.02 (0.02; 0.02) | 0.02 (0.02; 0.03) | 7.15 (7.01; 7.29) | 0.01 (0.01; 0.01) | 0.01 (0.01; 0.01) | 4.02 (3.89; 4.15) | 2.23 (1.63; 2.94) | 2.44 (1.78; 3.23) | 8.91 (8.47; 9.35) | 80.40 (74.05; 86.75) | 78.54 (70.34; 88.74) | -2.11 (-2.26; -1.97) | 82.62 (76.00; 89.25) | 80.99 (72.27; 91.58) | -1.81 (-1.96; -1.67) | 2.90 (2.67; 3.12) | 2.99 (2.69; 3.34) | 2.82 (2.68; 2.96) | 0.04 (0.04; 0.05) | 0.05 (0.04; 0.05) | 10.35 (10.19; 10.50) | 0.01 (0.01; 0.01) | 0.01 (0.01; 0.01) | 15.56 (15.41; 15.72) | 0.00 (0.00; 0.01) | 0.01 (0.00; 0.01) | 17.24 (16.80; 17.68) | 0.15 (0.14; 0.17) | 0.15 (0.14; 0.17) | 0.91 (0.76; 1.05) | 0.16 (0.15; 0.17) | 0.16 (0.14; 0.17) | 1.37 (1.22; 1.51) | 0.01 (0.01; 0.01) | 0.01 (0.01; 0.01) | 23.10 (22.94; 23.25) |
| **Albania** | 0.01 (0.01; 0.01) | 0.02 (0.01; 0.02) | 46.97 (46.27; 47.66) | 0.00 (0.00; 0.00) | 0.00 (0.00; 0.01) | 37.90 (37.21; 38.59) | 1.08 (0.66; 1.65) | 1.63 (1.00; 2.53) | 48.19 (47.35; 49.03) | 35.98 (24.27; 51.36) | 46.33 (30.55; 67.78) | 27.16 (26.52; 27.81) | 37.05 (25.01; 52.85) | 47.96 (32.03; 70.16) | 27.78 (27.15; 28.41) | 1.47 (1.02; 2.06) | 1.96 (1.33; 2.78) | 30.86 (30.25; 31.46) | 0.00 (0.00; 0.00) | 0.00 (0.00; 0.00) | 75.06 (74.20; 75.92) | 0.00 (0.00; 0.00) | 0.00 (0.00; 0.00) | 85.75 (84.87; 86.63) | 0.00 (0.00; 0.00) | 0.00 (0.00; 0.00) | 89.45 (88.36; 90.55) | 0.00 (0.00; 0.00) | 0.00 (0.00; 0.00) | 57.08 (56.33; 57.83) | 0.00 (0.00; 0.00) | 0.00 (0.00; 0.00) | 58.07 (57.33; 58.80) | 0.00 (0.00; 0.00) | 0.00 (0.00; 0.00) | 94.90 (94.11; 95.69) |
| **Bosnia and Herzegovina** | 0.01 (0.01; 0.02) | 0.02 (0.01; 0.02) | 25.83 (25.38; 26.29) | 0.00 (0.00; 0.00) | 0.00 (0.00; 0.01) | 19.37 (18.94; 19.80) | 1.35 (0.88; 1.93) | 1.73 (1.09; 2.51) | 27.24 (26.59; 27.89) | 47.48 (35.29; 61.08) | 51.80 (36.00; 68.81) | 9.85 (9.41; 10.29) | 48.84 (37.10; 63.33) | 53.53 (37.87; 70.77) | 10.34 (9.91; 10.76) | 1.78 (1.35; 2.26) | 2.04 (1.46; 2.64) | 14.57 (14.15; 14.99) | 0.00 (0.00; 0.00) | 0.00 (0.00; 0.00) | 21.01 (20.56; 21.47) | 0.00 (0.00; 0.00) | 0.00 (0.00; 0.00) | 29.89 (29.41; 30.37) | 0.00 (0.00; 0.00) | 0.00 (0.00; 0.00) | 32.13 (31.45; 32.80) | 0.00 (0.00; 0.00) | 0.00 (0.00; 0.00) | 6.74 (6.33; 7.14) | 0.00 (0.00; 0.00) | 0.00 (0.00; 0.00) | 7.47 (7.07; 7.87) | 0.00 (0.00; 0.00) | 0.00 (0.00; 0.00) | 38.63 (38.18; 39.08) |
| **Bulgaria** | 0.03 (0.02; 0.03) | 0.03 (0.02; 0.04) | 8.87 (8.51; 9.23) | 0.01 (0.00; 0.01) | 0.01 (0.01; 0.01) | 9.35 (8.99; 9.70) | 2.57 (1.75; 3.67) | 2.86 (1.89; 4.19) | 10.14 (9.56; 10.71) | 66.83 (53.15; 82.50) | 71.45 (54.29; 91.22) | 6.91 (6.56; 7.25) | 69.40 (55.93; 84.46) | 74.31 (56.97; 94.81) | 7.03 (6.69; 7.37) | 2.32 (1.87; 2.84) | 2.59 (2.03; 3.24) | 11.31 (10.98; 11.63) | 0.00 (0.00; 0.00) | 0.00 (0.00; 0.00) | -12.53 (-12.88; -12.18) | 0.00 (0.00; 0.00) | 0.00 (0.00; 0.00) | -7.20 (-7.55; -6.85) | 0.00 (0.00; 0.00) | 0.00 (0.00; 0.00) | -8.35 (-8.88; -7.82) | 0.01 (0.01; 0.01) | 0.01 (0.00; 0.01) | -14.40 (-14.73; -14.07) | 0.01 (0.01; 0.01) | 0.01 (0.01; 0.01) | -14.16 (-14.49; -13.84) | 0.00 (0.00; 0.00) | 0.00 (0.00; 0.00) | -0.63 (-0.97; -0.29) |
| **Croatia** | 0.03 (0.03; 0.04) | 0.03 (0.03; 0.04) | 5.48 (5.22; 5.75) | 0.01 (0.01; 0.01) | 0.01 (0.01; 0.01) | 1.65 (1.39; 1.91) | 2.95 (2.11; 4.09) | 3.18 (2.22; 4.43) | 7.01 (6.51; 7.51) | 63.19 (53.32; 74.11) | 57.71 (47.08; 71.44) | -8.57 (-8.83; -8.31) | 66.14 (55.78; 77.44) | 60.89 (49.80; 75.06) | -7.87 (-8.12; -7.61) | 2.41 (2.06; 2.80) | 2.36 (1.95; 2.88) | -2.78 (-3.03; -2.53) | 0.00 (0.00; 0.00) | 0.00 (0.00; 0.00) | 3.72 (3.41; 4.04) | 0.00 (0.00; 0.00) | 0.00 (0.00; 0.00) | 6.82 (6.50; 7.14) | 0.00 (0.00; 0.00) | 0.00 (0.00; 0.00) | 9.42 (8.90; 9.95) | 0.00 (0.00; 0.01) | 0.00 (0.00; 0.01) | -11.56 (-11.85; -11.28) | 0.01 (0.00; 0.01) | 0.00 (0.00; 0.01) | -10.57 (-10.85; -10.29) | 0.00 (0.00; 0.00) | 0.00 (0.00; 0.00) | 10.04 (9.73; 10.35) |
| **Czechia** | 0.02 (0.02; 0.02) | 0.02 (0.02; 0.02) | 3.28 (3.01; 3.55) | 0.00 (0.00; 0.01) | 0.00 (0.00; 0.01) | -0.45 (-0.71; -0.18) | 1.83 (1.27; 2.51) | 1.91 (1.30; 2.74) | 4.47 (3.95; 4.99) | 61.93 (51.87; 73.90) | 57.44 (46.48; 74.24) | -6.89 (-7.17; -6.60) | 63.76 (53.76; 76.24) | 59.35 (48.47; 76.57) | -6.56 (-6.84; -6.28) | 2.35 (1.99; 2.76) | 2.29 (1.89; 2.85) | -2.65 (-2.91; -2.39) | 0.00 (0.00; 0.00) | 0.00 (0.00; 0.00) | 12.64 (12.32; 12.96) | 0.00 (0.00; 0.00) | 0.00 (0.00; 0.00) | 17.27 (16.95; 17.59) | 0.00 (0.00; 0.00) | 0.00 (0.00; 0.00) | 19.12 (18.57; 19.67) | 0.01 (0.01; 0.01) | 0.01 (0.01; 0.01) | 0.64 (0.34; 0.94) | 0.01 (0.01; 0.01) | 0.01 (0.01; 0.01) | 1.20 (0.91; 1.49) | 0.00 (0.00; 0.00) | 0.00 (0.00; 0.00) | 24.55 (24.23; 24.87) |
| **Hungary** | 0.04 (0.03; 0.04) | 0.04 (0.03; 0.04) | 0.76 (0.51; 1.00) | 0.01 (0.01; 0.01) | 0.01 (0.01; 0.01) | -1.45 (-1.69; -1.22) | 3.63 (2.53; 4.98) | 3.72 (2.59; 5.11) | 2.05 (1.56; 2.53) | 117.29 (98.02; 136.46) | 106.31 (83.16; 135.74) | -9.23 (-9.51; -8.94) | 120.92 (102.66; 141.04) | 110.03 (86.19; 139.86) | -8.89 (-9.16; -8.61) | 4.14 (3.48; 4.81) | 3.98 (3.22; 5.00) | -4.34 (-4.61; -4.07) | 0.01 (0.01; 0.01) | 0.01 (0.01; 0.01) | 1.81 (1.52; 2.09) | 0.00 (0.00; 0.00) | 0.00 (0.00; 0.00) | 4.50 (4.21; 4.79) | 0.00 (0.00; 0.00) | 0.00 (0.00; 0.00) | 5.83 (5.33; 6.32) | 0.02 (0.02; 0.02) | 0.02 (0.01; 0.02) | -7.31 (-7.63; -6.99) | 0.02 (0.02; 0.02) | 0.02 (0.01; 0.02) | -6.90 (-7.22; -6.59) | 0.00 (0.00; 0.00) | 0.00 (0.00; 0.00) | 8.64 (8.32; 8.96) |
| **Montenegro** | 0.02 (0.01; 0.02) | 0.02 (0.01; 0.02) | 3.28 (2.92; 3.64) | 0.00 (0.00; 0.01) | 0.00 (0.00; 0.01) | 1.27 (0.93; 1.62) | 1.89 (1.30; 2.70) | 1.98 (1.31; 2.90) | 4.41 (3.85; 4.98) | 60.30 (46.14; 75.60) | 57.18 (42.07; 74.55) | -4.60 (-4.97; -4.24) | 62.20 (47.60; 77.48) | 59.16 (43.73; 76.55) | -4.33 (-4.68; -3.97) | 2.22 (1.76; 2.73) | 2.21 (1.69; 2.78) | -0.46 (-0.79; -0.13) | 0.00 (0.00; 0.00) | 0.00 (0.00; 0.00) | 9.73 (9.30; 10.15) | 0.00 (0.00; 0.00) | 0.00 (0.00; 0.00) | 14.72 (14.31; 15.13) | 0.00 (0.00; 0.00) | 0.00 (0.00; 0.00) | 15.39 (14.76; 16.03) | 0.00 (0.00; 0.00) | 0.00 (0.00; 0.00) | 0.30 (-0.10; 0.71) | 0.00 (0.00; 0.00) | 0.00 (0.00; 0.00) | 0.78 (0.38; 1.18) | 0.00 (0.00; 0.00) | 0.00 (0.00; 0.00) | 18.92 (18.51; 19.33) |
| **North Macedonia** | 0.01 (0.01; 0.01) | 0.01 (0.01; 0.02) | 18.51 (18.09; 18.94) | 0.00 (0.00; 0.00) | 0.00 (0.00; 0.00) | 15.14 (14.75; 15.53) | 1.18 (0.82; 1.73) | 1.45 (0.98; 2.10) | 21.17 (20.56; 21.77) | 42.88 (33.14; 54.81) | 45.25 (34.13; 59.44) | 5.39 (5.00; 5.78) | 44.08 (34.09; 56.38) | 46.70 (35.45; 60.86) | 5.82 (5.44; 6.20) | 1.70 (1.36; 2.13) | 1.94 (1.52; 2.43) | 12.56 (12.21; 12.91) | 0.00 (0.00; 0.00) | 0.00 (0.00; 0.00) | 43.84 (43.24; 44.43) | 0.00 (0.00; 0.00) | 0.00 (0.00; 0.00) | 51.23 (50.63; 51.83) | 0.00 (0.00; 0.00) | 0.00 (0.00; 0.00) | 54.60 (53.82; 55.38) | 0.00 (0.00; 0.00) | 0.00 (0.00; 0.00) | 26.90 (26.39; 27.40) | 0.00 (0.00; 0.00) | 0.00 (0.00; 0.00) | 27.66 (27.16; 28.16) | 0.00 (0.00; 0.00) | 0.00 (0.00; 0.00) | 55.40 (54.85; 55.94) |
| **Poland** | 0.02 (0.01; 0.02) | 0.02 (0.02; 0.02) | 9.86 (9.65; 10.06) | 0.01 (0.00; 0.01) | 0.01 (0.00; 0.01) | 4.46 (4.28; 4.65) | 1.85 (1.33; 2.44) | 2.06 (1.44; 2.82) | 10.98 (10.50; 11.46) | 82.70 (75.12; 90.23) | 80.07 (65.89; 94.11) | -2.68 (-2.88; -2.49) | 84.54 (77.13; 92.25) | 82.14 (67.86; 96.25) | -2.38 (-2.58; -2.19) | 3.05 (2.77; 3.31) | 3.11 (2.62; 3.58) | 1.86 (1.68; 2.05) | 0.01 (0.01; 0.01) | 0.01 (0.01; 0.02) | 21.33 (21.06; 21.60) | 0.00 (0.00; 0.00) | 0.00 (0.00; 0.01) | 27.48 (27.21; 27.75) | 0.00 (0.00; 0.00) | 0.00 (0.00; 0.00) | 29.48 (28.95; 30) | 0.05 (0.05; 0.06) | 0.06 (0.05; 0.07) | 9.03 (8.78; 9.27) | 0.05 (0.05; 0.06) | 0.06 (0.05; 0.07) | 9.50 (9.26; 9.74) | 0.00 (0.00; 0.00) | 0.00 (0.00; 0.00) | 35.88 (35.61; 36.14) |
| **Romania** | 0.03 (0.02; 0.03) | 0.03 (0.02; 0.03) | 10.03 (9.75; 10.30) | 0.01 (0.01; 0.01) | 0.01 (0.01; 0.01) | 5.21 (4.95; 5.46) | 2.58 (1.80; 3.57) | 2.89 (1.99; 4.02) | 11.15 (10.63; 11.67) | 100.01 (83.74; 116.88) | 101.08 (78.18; 126.70) | 1.30 (1.00; 1.59) | 102.57 (87.47; 119.67) | 103.98 (81.07; 129.61) | 1.55 (1.25; 1.84) | 3.40 (2.87; 3.97) | 3.59 (2.86; 4.48) | 5.36 (5.07; 5.65) | 0.01 (0.01; 0.01) | 0.01 (0.01; 0.01) | -1.79 (-2.12; -1.46) | 0.00 (0.00; 0.00) | 0.00 (0.00; 0.00) | -0.09 (-0.42; 0.24) | 0.00 (0.00; 0.00) | 0.00 (0.00; 0.00) | 3.62 (3.11; 4.13) | 0.03 (0.03; 0.04) | 0.03 (0.02; 0.03) | -11.70 (-12.01; -11.39) | 0.03 (0.03; 0.04) | 0.03 (0.02; 0.04) | -11.30 (-11.60; -10.99) | 0.00 (0.00; 0.00) | 0.00 (0.00; 0.00) | 5.30 (4.97; 5.62) |
| **Serbia** | 0.02 (0.01; 0.02) | 0.02 (0.02; 0.03) | 23.60 (23.20; 23.99) | 0.00 (0.00; 0.01) | 0.01 (0.00; 0.01) | 18.06 (17.67; 18.44) | 1.91 (1.28; 2.72) | 2.43 (1.57; 3.58) | 25.79 (25.15; 26.44) | 60.27 (47.05; 75.11) | 66.17 (49.85; 84.16) | 8.81 (8.45; 9.17) | 62.16 (48.95; 77.12) | 68.60 (52.11; 87.10) | 9.33 (8.97; 9.69) | 2.26 (1.79; 2.81) | 2.64 (2.03; 3.37) | 14.77 (14.40; 15.14) | 0.00 (0.00; 0.00) | 0.00 (0.00; 0.00) | 30.54 (30.05; 31.03) | 0.00 (0.00; 0.00) | 0.00 (0.00; 0.00) | 29.44 (28.95; 29.93) | 0.00 (0.00; 0.00) | 0.00 (0.00; 0.00) | 36 (35.26; 36.74) | 0.01 (0.01; 0.01) | 0.01 (0.01; 0.01) | 15.21 (14.80; 15.61) | 0.01 (0.01; 0.01) | 0.01 (0.01; 0.01) | 15.87 (15.47; 16.27) | 0.00 (0.00; 0.00) | 0.00 (0.00; 0.00) | 29.65 (29.23; 30.06) |
| **Slovakia** | 0.03 (0.02; 0.04) | 0.03 (0.02; 0.04) | 2.46 (2.06; 2.86) | 0.01 (0.01; 0.01) | 0.01 (0.01; 0.01) | 1.86 (1.46; 2.25) | 2.91 (1.81; 4.11) | 3.03 (1.87; 4.32) | 3.79 (3.22; 4.37) | 104.87 (77.24; 136.00) | 101.73 (71.22; 142.64) | -3.20 (-3.64; -2.75) | 107.81 (79.12; 138.93) | 104.76 (74.01; 146.08) | -3.01 (-3.44; -2.58) | 3.63 (2.71; 4.68) | 3.76 (2.70; 5.16) | 2.68 (2.25; 3.12) | 0.00 (0.00; 0.00) | 0.00 (0.00; 0.00) | 17.67 (17.24; 18.11) | 0.00 (0.00; 0.00) | 0.00 (0.00; 0.00) | 25.27 (24.81; 25.72) | 0.00 (0.00; 0.00) | 0.00 (0.00; 0.00) | 23.88 (23.21; 24.55) | 0.01 (0.01; 0.01) | 0.01 (0.01; 0.01) | 7.24 (6.78; 7.70) | 0.01 (0.01; 0.01) | 0.01 (0.01; 0.01) | 7.71 (7.26; 8.16) | 0.00 (0.00; 0.00) | 0.00 (0.00; 0.00) | 31.98 (31.50; 32.47) |
| **Slovenia** | 0.03 (0.02; 0.03) | 0.02 (0.02; 0.03) | -9.41 (-9.68; -9.14) | 0.00 (0.00; 0.01) | 0.00 (0.00; 0.01) | -12.88 (-13.14; -12.61) | 2.27 (1.56; 3.25) | 2.09 (1.41; 3.06) | -7.75 (-8.27; -7.23) | 39.45 (33.00; 47.12) | 30.53 (24.39; 37.90) | -21.74 (-21.98; -21.50) | 41.73 (35.18; 49.96) | 32.62 (26.47; 40.45) | -20.97 (-21.21; -20.73) | 1.52 (1.28; 1.79) | 1.28 (1.05; 1.55) | -15.37 (-15.60; -15.14) | 0.00 (0.00; 0.00) | 0.00 (0.00; 0.00) | 3.67 (3.36; 3.98) | 0.00 (0.00; 0.00) | 0.00 (0.00; 0.00) | 9.71 (9.40; 10.03) | 0.00 (0.00; 0.00) | 0.00 (0.00; 0.00) | 11.74 (11.17; 12.32) | 0.00 (0.00; 0.00) | 0.00 (0.00; 0.00) | -5.73 (-6.01; -5.45) | 0.00 (0.00; 0.00) | 0.00 (0.00; 0.00) | -4.76 (-5.03; -4.49) | 0.00 (0.00; 0.00) | 0.00 (0.00; 0.00) | 25.46 (25.15; 25.78) |
| **Eastern Europe** | 0.02 (0.02; 0.02) | 0.02 (0.02; 0.02) | 0.36 (0.20; 0.53) | 0.01 (0.01; 0.01) | 0.01 (0.01; 0.01) | -2.66 (-2.83; -2.50) | 2.26 (1.65; 2.96) | 2.29 (1.64; 3.06) | 1.11 (0.68; 1.54) | 79.44 (71.48; 87.92) | 73.87 (62.88; 86.24) | -7.02 (-7.20; -6.84) | 81.71 (73.49; 90.43) | 76.16 (64.74; 88.94) | -6.79 (-6.97; -6.62) | 2.67 (2.42; 2.94) | 2.62 (2.28; 3.04) | -2.20 (-2.37; -2.03) | 0.06 (0.06; 0.07) | 0.06 (0.05; 0.07) | 2.02 (1.81; 2.23) | 0.02 (0.02; 0.02) | 0.02 (0.02; 0.03) | 5.51 (5.29; 5.73) | 0.01 (0.01; 0.01) | 0.01 (0.01; 0.01) | 7.48 (7.02; 7.94) | 0.25 (0.23; 0.28) | 0.25 (0.21; 0.29) | -3.32 (-3.53; -3.12) | 0.26 (0.24; 0.29) | 0.25 (0.22; 0.30) | -3.02 (-3.23; -2.82) | 0.01 (0.01; 0.01) | 0.01 (0.01; 0.01) | 16.52 (16.30; 16.74) |
| **Belarus** | 0.02 (0.02; 0.03) | 0.03 (0.02; 0.04) | 11.09 (10.72; 11.46) | 0.01 (0.01; 0.01) | 0.01 (0.01; 0.01) | 9.59 (9.22; 9.97) | 2.71 (1.86; 3.83) | 3.09 (2.04; 4.56) | 12.94 (12.35; 13.53) | 98.18 (75.62; 124.46) | 103.97 (74.85; 135.93) | 4.47 (4.09; 4.86) | 100.86 (78.51; 128.93) | 107.06 (77.69; 139.02) | 4.70 (4.32; 5.07) | 3.26 (2.55; 4.10) | 3.68 (2.74; 4.71) | 10.71 (10.34; 11.08) | 0.00 (0.00; 0.00) | 0.00 (0.00; 0.01) | 15.23 (14.80; 15.66) | 0.00 (0.00; 0.00) | 0.00 (0.00; 0.00) | 19.08 (18.64; 19.51) | 0.00 (0.00; 0.00) | 0.00 (0.00; 0.00) | 21.54 (20.90; 22.19) | 0.01 (0.01; 0.02) | 0.02 (0.01; 0.02) | 4.18 (3.80; 4.57) | 0.01 (0.01; 0.02) | 0.02 (0.01; 0.02) | 4.66 (4.28; 5.03) | 0.00 (0.00; 0.00) | 0.00 (0.00; 0.00) | 25.12 (24.74; 25.51) |
| **Estonia** | 0.03 (0.02; 0.03) | 0.03 (0.02; 0.04) | 1.81 (1.54; 2.08) | 0.01 (0.01; 0.01) | 0.01 (0.01; 0.01) | -1.46 (-1.72; -1.20) | 2.69 (1.87; 3.73) | 2.79 (1.90; 3.92) | 3.80 (3.29; 4.31) | 59.34 (49.25; 69.29) | 53.04 (41.37; 67.53) | -10.25 (-10.53; -9.98) | 62.03 (51.17; 72.38) | 55.84 (43.92; 70.05) | -9.64 (-9.91; -9.38) | 2.26 (1.89; 2.60) | 2.19 (1.74; 2.72) | -3.07 (-3.33; -2.80) | 0.00 (0.00; 0.00) | 0.00 (0.00; 0.00) | 14.44 (14.13; 14.76) | 0.00 (0.00; 0.00) | 0.00 (0.00; 0.00) | 17.65 (17.33; 17.96) | 0.00 (0.00; 0.00) | 0.00 (0.00; 0.00) | 20.40 (19.82; 20.98) | 0.00 (0.00; 0.00) | 0.00 (0.00; 0.00) | 4.16 (3.86; 4.47) | 0.00 (0.00; 0.00) | 0.00 (0.00; 0.00) | 4.90 (4.60; 5.20) | 0.00 (0.00; 0.00) | 0.00 (0.00; 0.00) | 26.50 (26.17; 26.83) |
| **Latvia** | 0.02 (0.02; 0.02) | 0.02 (0.02; 0.03) | 16.03 (15.77; 16.30) | 0.01 (0.00; 0.01) | 0.01 (0.01; 0.01) | 14.81 (14.53; 15.09) | 2.04 (1.42; 2.87) | 2.44 (1.69; 3.56) | 18.46 (17.89; 19.03) | 83.99 (68.96; 101.09) | 91.54 (72.39; 114.21) | 8.03 (7.72; 8.34) | 86.00 (71.24; 103.00) | 93.98 (74.66; 116.89) | 8.28 (7.98; 8.59) | 3.02 (2.49; 3.61) | 3.53 (2.85; 4.31) | 15.24 (14.94; 15.54) | 0.00 (0.00; 0.00) | 0.00 (0.00; 0.00) | 3.87 (3.59; 4.15) | 0.00 (0.00; 0.00) | 0.00 (0.00; 0.00) | 10.86 (10.57; 11.16) | 0.00 (0.00; 0.00) | 0.00 (0.00; 0.00) | 10.44 (9.90; 10.99) | 0.00 (0.00; 0.00) | 0.00 (0.00; 0.00) | -3.18 (-3.48; -2.88) | 0.00 (0.00; 0.00) | 0.00 (0.00; 0.00) | -2.84 (-3.13; -2.55) | 0.00 (0.00; 0.00) | 0.00 (0.00; 0.00) | 17.41 (17.08; 17.73) |
| **Lithuania** | 0.02 (0.02; 0.02) | 0.02 (0.02; 0.03) | 19.79 (19.51; 20.06) | 0.01 (0.01; 0.01) | 0.01 (0.01; 0.01) | 20.18 (19.87; 20.48) | 2.38 (1.67; 3.28) | 2.93 (1.99; 4.10) | 21.42 (20.86; 21.98) | 91.17 (76.64; 106.49) | 106.15 (82.02; 133.00) | 15.20 (14.88; 15.52) | 93.55 (78.43; 109.54) | 109.08 (85.11; 136.93) | 15.36 (15.04; 15.68) | 3.16 (2.67; 3.70) | 3.90 (3.07; 4.81) | 21.77 (21.45; 22.09) | 0.00 (0.00; 0.00) | 0.00 (0.00; 0.00) | 3.19 (2.91; 3.47) | 0.00 (0.00; 0.00) | 0.00 (0.00; 0.00) | 10.49 (10.18; 10.80) | 0.00 (0.00; 0.00) | 0.00 (0.00; 0.00) | 8.70 (8.18; 9.21) | 0.00 (0.00; 0.01) | 0.00 (0.00; 0.01) | -2.14 (-2.44; -1.85) | 0.00 (0.00; 0.01) | 0.00 (0.00; 0.01) | -1.86 (-2.15; -1.57) | 0.00 (0.00; 0.00) | 0.00 (0.00; 0.00) | 19.34 (19.02; 19.65) |
| **Republic of Moldova** | 0.01 (0.01; 0.02) | 0.02 (0.01; 0.02) | 11.26 (11; 11.52) | 0.00 (0.00; 0.01) | 0.01 (0.00; 0.01) | 10.17 (9.91; 10.43) | 1.64 (1.17; 2.25) | 1.86 (1.26; 2.62) | 12.61 (12.08; 13.13) | 73.80 (64.80; 83.49) | 79.41 (62.78; 100.42) | 7.36 (7.07; 7.65) | 75.43 (66.73; 85.19) | 81.27 (64.64; 102.47) | 7.47 (7.19; 7.76) | 2.56 (2.25; 2.88) | 2.83 (2.26; 3.55) | 10.10 (9.82; 10.38) | 0.00 (0.00; 0.00) | 0.00 (0.00; 0.00) | 10.63 (10.28; 10.97) | 0.00 (0.00; 0.00) | 0.00 (0.00; 0.00) | 18.16 (17.78; 18.54) | 0.00 (0.00; 0.00) | 0.00 (0.00; 0.00) | 18.09 (17.49; 18.68) | 0.00 (0.00; 0.00) | 0.00 (0.00; 0.01) | 4.76 (4.39; 5.13) | 0.00 (0.00; 0.00) | 0.00 (0.00; 0.01) | 5.05 (4.68; 5.42) | 0.00 (0.00; 0.00) | 0.00 (0.00; 0.00) | 25.45 (25.06; 25.84) |
| **Russian Federation** | 0.02 (0.02; 0.02) | 0.02 (0.02; 0.02) | -2.84 (-3.02; -2.66) | 0.01 (0.01; 0.01) | 0.01 (0.01; 0.01) | -6.43 (-6.61; -6.25) | 2.19 (1.58; 2.86) | 2.14 (1.52; 2.91) | -2.29 (-2.72; -1.85) | 76.29 (69.46; 83.03) | 68.28 (57.01; 81.83) | -10.20 (-10.39; -10.01) | 78.48 (71.30; 85.70) | 70.42 (58.77; 84.18) | -9.98 (-10.17; -9.79) | 2.59 (2.36; 2.81) | 2.45 (2.08; 2.90) | -5.48 (-5.67; -5.30) | 0.04 (0.04; 0.04) | 0.04 (0.03; 0.05) | 0.01 (-0.25; 0.26) | 0.01 (0.01; 0.02) | 0.01 (0.01; 0.02) | 4.05 (3.79; 4.32) | 0.00 (0.00; 0.01) | 0.01 (0.00; 0.01) | 6.02 (5.55; 6.50) | 0.17 (0.15; 0.18) | 0.16 (0.13; 0.20) | -2.33 (-2.60; -2.06) | 0.17 (0.16; 0.19) | 0.17 (0.14; 0.21) | -2.10 (-2.36; -1.83) | 0.01 (0.01; 0.01) | 0.01 (0.01; 0.01) | 18.57 (18.29; 18.85) |
| **Ukraine** | 0.02 (0.02; 0.03) | 0.02 (0.02; 0.03) | 5.76 (5.30; 6.22) | 0.01 (0.00; 0.01) | 0.01 (0.00; 0.01) | 3.95 (3.48; 4.41) | 2.42 (1.52; 3.58) | 2.59 (1.62; 3.84) | 6.57 (5.95; 7.19) | 85.45 (59.40; 116.61) | 85.15 (58.32; 118.93) | -0.86 (-1.34; -0.38) | 87.89 (60.86; 122.30) | 87.74 (60.53; 122.18) | -0.66 (-1.13; -0.19) | 2.77 (1.95; 3.74) | 2.90 (2.04; 3.94) | 3.58 (3.12; 4.04) | 0.02 (0.01; 0.02) | 0.02 (0.01; 0.02) | 2.94 (2.38; 3.50) | 0.00 (0.00; 0.01) | 0.00 (0.00; 0.01) | 4.97 (4.40; 5.55) | 0.00 (0.00; 0.00) | 0.00 (0.00; 0.00) | 6.95 (6.25; 7.66) | 0.06 (0.04; 0.08) | 0.05 (0.04; 0.08) | -8.85 (-9.34; -8.36) | 0.06 (0.04; 0.08) | 0.06 (0.04; 0.08) | -8.40 (-8.88; -7.92) | 0.00 (0.00; 0.00) | 0.00 (0.00; 0.00) | 7.05 (6.57; 7.54) |
| **High-income** | 0.03 (0.03; 0.03) | 0.03 (0.03; 0.03) | 0.98 (0.91; 1.05) | 0.01 (0.01; 0.01) | 0.01 (0.00; 0.01) | -2.08 (-2.16; -1.99) | 2.43 (1.81; 3.19) | 2.48 (1.84; 3.25) | 1.75 (1.34; 2.15) | 33.33 (31.72; 34.50) | 30.12 (27.98; 32.08) | -9.22 (-9.30; -9.15) | 35.76 (33.89; 37.16) | 32.60 (30.34; 34.77) | -8.47 (-8.55; -8.40) | 1.39 (1.28; 1.46) | 1.32 (1.19; 1.42) | -5.22 (-5.32; -5.11) | 0.51 (0.48; 0.53) | 0.63 (0.58; 0.68) | 22.10 (22; 22.20) | 0.11 (0.10; 0.11) | 0.14 (0.12; 0.15) | 28.58 (28.44; 28.71) | 0.05 (0.04; 0.06) | 0.06 (0.04; 0.08) | 28.08 (27.62; 28.53) | 0.64 (0.60; 0.67) | 0.74 (0.66; 0.80) | 15.52 (15.40; 15.65) | 0.69 (0.64; 0.72) | 0.80 (0.72; 0.87) | 16.39 (16.27; 16.51) | 0.03 (0.03; 0.03) | 0.04 (0.04; 0.05) | 41.16 (40.96; 41.35) |
| **Australasia** | 0.04 (0.04; 0.04) | 0.04 (0.03; 0.04) | -3.71 (-3.87; -3.55) | 0.01 (0.01; 0.01) | 0.01 (0.01; 0.01) | -7.20 (-7.36; -7.05) | 3.39 (2.39; 4.51) | 3.29 (2.31; 4.40) | -2.78 (-3.22; -2.34) | 30.78 (27.87; 33.84) | 26.45 (22.96; 30.28) | -13.11 (-13.26; -12.95) | 34.17 (31.05; 37.61) | 29.74 (25.74; 33.74) | -12.08 (-12.23; -11.93) | 1.33 (1.18; 1.46) | 1.21 (1.03; 1.37) | -8.25 (-8.42; -8.09) | 0.02 (0.02; 0.02) | 0.03 (0.02; 0.03) | 48.25 (48; 48.50) | 0.00 (0.00; 0.00) | 0.01 (0.00; 0.01) | 51.85 (51.60; 52.10) | 0.00 (0.00; 0.00) | 0.00 (0.00; 0.00) | 54.06 (53.47; 54.65) | 0.02 (0.01; 0.02) | 0.02 (0.02; 0.02) | 41.40 (41.17; 41.64) | 0.02 (0.02; 0.02) | 0.02 (0.02; 0.03) | 42.67 (42.44; 42.89) | 0.00 (0.00; 0.00) | 0.00 (0.00; 0.00) | 72.23 (71.93; 72.53) |
| **Australia** | 0.04 (0.04; 0.05) | 0.04 (0.04; 0.05) | -3.90 (-4.07; -3.72) | 0.01 (0.01; 0.01) | 0.01 (0.01; 0.01) | -7.53 (-7.70; -7.36) | 3.55 (2.49; 4.76) | 3.44 (2.40; 4.61) | -2.94 (-3.39; -2.50) | 31.09 (27.91; 34.51) | 26.71 (22.62; 31.14) | -13.13 (-13.30; -12.96) | 34.64 (31.22; 38.56) | 30.15 (25.75; 34.72) | -12.08 (-12.25; -11.91) | 1.34 (1.19; 1.49) | 1.23 (1.03; 1.42) | -8.23 (-8.41; -8.05) | 0.02 (0.02; 0.02) | 0.03 (0.02; 0.03) | 47.25 (46.98; 47.52) | 0.00 (0.00; 0.00) | 0.00 (0.00; 0.01) | 50.60 (50.33; 50.88) | 0.00 (0.00; 0.00) | 0.00 (0.00; 0.00) | 53.12 (52.53; 53.71) | 0.01 (0.01; 0.01) | 0.02 (0.02; 0.02) | 41.32 (41.06; 41.57) | 0.01 (0.01; 0.02) | 0.02 (0.02; 0.02) | 42.53 (42.28; 42.77) | 0.00 (0.00; 0.00) | 0.00 (0.00; 0.00) | 72.33 (71.99; 72.66) |
| **New Zealand** | 0.03 (0.03; 0.03) | 0.03 (0.03; 0.03) | -1.88 (-2.06; -1.70) | 0.01 (0.00; 0.01) | 0.01 (0.00; 0.01) | -4.53 (-4.70; -4.35) | 2.56 (1.80; 3.45) | 2.53 (1.74; 3.51) | -1.21 (-1.68; -0.73) | 29.21 (26.31; 32.33) | 25.14 (21.74; 28.99) | -13.05 (-13.21; -12.89) | 31.77 (28.56; 35.33) | 27.67 (24.28; 31.75) | -12.09 (-12.24; -11.93) | 1.25 (1.11; 1.40) | 1.14 (0.99; 1.31) | -8.39 (-8.56; -8.22) | 0.00 (0.00; 0.00) | 0.00 (0.00; 0.00) | 55.55 (55.26; 55.84) | 0.00 (0.00; 0.00) | 0.00 (0.00; 0.00) | 60.74 (60.44; 61.03) | 0.00 (0.00; 0.00) | 0.00 (0.00; 0.00) | 60.72 (60.07; 61.38) | 0.00 (0.00; 0.00) | 0.00 (0.00; 0.00) | 41.90 (41.63; 42.17) | 0.00 (0.00; 0.00) | 0.00 (0.00; 0.00) | 43.45 (43.19; 43.72) | 0.00 (0.00; 0.00) | 0.00 (0.00; 0.00) | 71.66 (71.32; 72) |
| **High-income Asia Pacific** | 0.02 (0.02; 0.02) | 0.02 (0.02; 0.02) | 7.96 (7.82; 8.10) | 0.00 (0.00; 0.00) | 0.00 (0.00; 0.00) | -0.93 (-1.07; -0.79) | 1.67 (1.22; 2.21) | 1.81 (1.27; 2.44) | 8.10 (7.64; 8.56) | 25.14 (23.09; 26.72) | 22.82 (20.02; 25.17) | -8.91 (-9.04; -8.78) | 26.82 (24.59; 28.51) | 24.63 (21.81; 27.24) | -7.84 (-7.97; -7.72) | 1.14 (0.99; 1.23) | 1.07 (0.91; 1.20) | -5.84 (-6.01; -5.68) | 0.06 (0.05; 0.07) | 0.07 (0.06; 0.08) | 16.85 (16.65; 17.06) | 0.02 (0.02; 0.02) | 0.02 (0.02; 0.03) | 24.88 (24.64; 25.11) | 0.01 (0.00; 0.01) | 0.01 (0.01; 0.01) | 26.74 (26.22; 27.25) | 0.10 (0.09; 0.11) | 0.12 (0.10; 0.14) | 16.22 (15.99; 16.45) | 0.11 (0.10; 0.12) | 0.13 (0.10; 0.15) | 16.86 (16.64; 17.08) | 0.01 (0.00; 0.01) | 0.01 (0.01; 0.01) | 45.44 (45.11; 45.78) |
| **Brunei Darussalam** | 0.01 (0.01; 0.02) | 0.01 (0.01; 0.02) | 5.91 (5.60; 6.21) | 0.00 (0.00; 0.01) | 0.00 (0.00; 0.01) | 4.48 (4.19; 4.78) | 1.53 (1.04; 2.19) | 1.68 (1.13; 2.41) | 8.87 (8.31; 9.43) | 56.74 (46.27; 67.90) | 55.83 (44.53; 68.71) | -1.19 (-1.47; -0.91) | 58.27 (47.83; 70.37) | 57.51 (46.11; 70.38) | -0.92 (-1.20; -0.65) | 2.31 (1.87; 2.77) | 2.48 (1.98; 3.07) | 6.60 (6.30; 6.90) | 0.00 (0.00; 0.00) | 0.00 (0.00; 0.00) | 93.12 (92.56; 93.68) | 0.00 (0.00; 0.00) | 0.00 (0.00; 0.00) | 146.52 (145.85; 147.19) | 0.00 (0.00; 0.00) | 0.00 (0.00; 0.00) | 131.83 (130.80; 132.85) | 0.00 (0.00; 0.00) | 0.00 (0.00; 0.00) | 106.21 (105.69; 106.73) | 0.00 (0.00; 0.00) | 0.00 (0.00; 0.00) | 106.89 (106.38; 107.39) | 0.00 (0.00; 0.00) | 0.00 (0.00; 0.00) | 194.65 (193.94; 195.36) |
| **Japan** | 0.02 (0.02; 0.02) | 0.02 (0.02; 0.02) | 11.88 (11.73; 12.02) | 0.00 (0.00; 0.01) | 0.00 (0.00; 0.01) | 3.16 (3.01; 3.31) | 1.86 (1.35; 2.45) | 2.10 (1.48; 2.78) | 12.02 (11.57; 12.48) | 27.51 (25.55; 28.64) | 25.88 (22.88; 28.68) | -5.91 (-6.03; -5.78) | 29.37 (27.28; 30.71) | 27.98 (24.86; 30.94) | -4.77 (-4.89; -4.64) | 1.23 (1.08; 1.31) | 1.21 (1.03; 1.36) | -1.66 (-1.83; -1.49) | 0.05 (0.04; 0.05) | 0.05 (0.05; 0.06) | 8.00 (7.80; 8.20) | 0.02 (0.01; 0.02) | 0.02 (0.01; 0.02) | 12.02 (11.78; 12.27) | 0.01 (0.00; 0.01) | 0.01 (0.00; 0.01) | 14.79 (14.31; 15.26) | 0.08 (0.07; 0.09) | 0.09 (0.07; 0.10) | 6.25 (6.02; 6.49) | 0.09 (0.08; 0.09) | 0.10 (0.08; 0.11) | 6.78 (6.56; 7.01) | 0.01 (0.00; 0.01) | 0.01 (0.00; 0.01) | 28.84 (28.50; 29.19) |
| **Republic of Korea** | 0.01 (0.01; 0.01) | 0.01 (0.01; 0.02) | 5.91 (5.57; 6.25) | 0.00 (0.00; 0.00) | 0.00 (0.00; 0.00) | -0.38 (-0.71; -0.05) | 1.20 (0.82; 1.70) | 1.30 (0.85; 1.88) | 7.76 (7.20; 8.32) | 18.83 (14.56; 22.81) | 16.81 (12.81; 21.11) | -9.81 (-10.13; -9.50) | 20.04 (15.51; 24.24) | 18.10 (14.09; 22.57) | -8.75 (-9.05; -8.45) | 0.82 (0.64; 0.99) | 0.79 (0.61; 0.98) | -3.37 (-3.68; -3.06) | 0.01 (0.01; 0.01) | 0.02 (0.01; 0.02) | 49.67 (49.24; 50.11) | 0.00 (0.00; 0.00) | 0.01 (0.00; 0.01) | 85.14 (84.64; 85.64) | 0.00 (0.00; 0.00) | 0.00 (0.00; 0.00) | 76.28 (75.46; 77.09) | 0.02 (0.01; 0.02) | 0.03 (0.02; 0.03) | 55.33 (54.90; 55.76) | 0.02 (0.01; 0.02) | 0.03 (0.02; 0.03) | 56.60 (56.18; 57.03) | 0.00 (0.00; 0.00) | 0.00 (0.00; 0.00) | 139.30 (138.66; 139.93) |
| **Singapore** | 0.01 (0.01; 0.01) | 0.01 (0.01; 0.01) | 3.02 (2.85; 3.19) | 0.00 (0.00; 0.00) | 0.00 (0.00; 0.00) | -1.80 (-1.96; -1.63) | 1.24 (0.89; 1.65) | 1.31 (0.91; 1.74) | 5.25 (4.79; 5.70) | 19.79 (18.03; 21.83) | 17.62 (15.38; 20.21) | -10.03 (-10.19; -9.87) | 21.03 (19.17; 23.08) | 18.93 (16.58; 21.62) | -9.12 (-9.27; -8.96) | 0.88 (0.79; 0.98) | 0.85 (0.73; 0.97) | -4.29 (-4.46; -4.12) | 0.00 (0.00; 0.00) | 0.00 (0.00; 0.00) | 99.82 (99.47; 100.17) | 0.00 (0.00; 0.00) | 0.00 (0.00; 0.00) | 135.70 (135.27; 136.13) | 0.00 (0.00; 0.00) | 0.00 (0.00; 0.00) | 127.89 (127.10; 128.67) | 0.00 (0.00; 0.00) | 0.00 (0.00; 0.00) | 102.50 (102.14; 102.86) | 0.00 (0.00; 0.00) | 0.00 (0.00; 0.00) | 104.02 (103.66; 104.37) | 0.00 (0.00; 0.00) | 0.00 (0.00; 0.00) | 195.53 (194.98; 196.09) |
| **High-income North America** | 0.03 (0.03; 0.03) | 0.03 (0.03; 0.04) | 1.61 (1.52; 1.70) | 0.01 (0.01; 0.01) | 0.01 (0.01; 0.01) | 2.29 (2.18; 2.39) | 2.78 (2.07; 3.67) | 2.86 (2.11; 3.74) | 2.29 (1.89; 2.70) | 31.45 (30.00; 32.55) | 30.47 (27.62; 33.33) | -3.31 (-3.41; -3.21) | 34.23 (32.63; 35.78) | 33.33 (30.12; 36.70) | -2.85 (-2.96; -2.74) | 1.34 (1.24; 1.40) | 1.36 (1.20; 1.49) | 0.84 (0.72; 0.96) | 0.20 (0.19; 0.21) | 0.25 (0.23; 0.28) | 26.49 (26.33; 26.64) | 0.04 (0.03; 0.04) | 0.05 (0.05; 0.06) | 37.71 (37.53; 37.89) | 0.02 (0.01; 0.02) | 0.02 (0.02; 0.03) | 31.46 (31; 31.93) | 0.19 (0.18; 0.20) | 0.24 (0.21; 0.27) | 25.23 (25.07; 25.40) | 0.21 (0.20; 0.22) | 0.27 (0.24; 0.30) | 25.75 (25.59; 25.91) | 0.01 (0.01; 0.01) | 0.01 (0.01; 0.02) | 52.45 (52.22; 52.69) |
| **Canada** | 0.03 (0.02; 0.03) | 0.03 (0.02; 0.03) | 3.45 (3.25; 3.65) | 0.00 (0.00; 0.01) | 0.00 (0.00; 0.01) | 0.05 (-0.14; 0.24) | 2.27 (1.63; 3.04) | 2.37 (1.69; 3.17) | 4.21 (3.76; 4.66) | 30.41 (26.83; 34.52) | 28.44 (24.18; 33.44) | -6.10 (-6.31; -5.90) | 32.67 (28.98; 36.93) | 30.80 (26.32; 35.73) | -5.39 (-5.58; -5.20) | 1.32 (1.16; 1.49) | 1.29 (1.10; 1.50) | -2.23 (-2.43; -2.03) | 0.02 (0.01; 0.02) | 0.02 (0.02; 0.03) | 42.12 (41.86; 42.39) | 0.00 (0.00; 0.00) | 0.01 (0.00; 0.01) | 51.70 (51.42; 51.97) | 0.00 (0.00; 0.00) | 0.00 (0.00; 0.00) | 49.68 (49.11; 50.25) | 0.02 (0.02; 0.02) | 0.03 (0.02; 0.03) | 39.16 (38.89; 39.43) | 0.02 (0.02; 0.02) | 0.03 (0.03; 0.04) | 39.90 (39.64; 40.15) | 0.00 (0.00; 0.00) | 0.00 (0.00; 0.00) | 74.41 (74.09; 74.74) |
| **Greenland** | 0.02 (0.02; 0.03) | 0.02 (0.02; 0.03) | 2.10 (1.78; 2.41) | 0.01 (0.00; 0.01) | 0.01 (0.00; 0.01) | -3.06 (-3.36; -2.76) | 2.26 (1.52; 3.13) | 2.32 (1.50; 3.31) | 2.76 (2.23; 3.30) | 73.64 (60.30; 90.80) | 66.48 (53.07; 81.54) | -8.55 (-8.83; -8.28) | 75.89 (63.29; 91.67) | 68.80 (55.01; 84.38) | -8.21 (-8.48; -7.94) | 3.05 (2.50; 3.75) | 2.85 (2.28; 3.53) | -6.04 (-6.32; -5.75) | 0.00 (0.00; 0.00) | 0.00 (0.00; 0.00) | 30.31 (29.91; 30.70) | 0.00 (0.00; 0.00) | 0.00 (0.00; 0.00) | 50.60 (50.17; 51.03) | 0.00 (0.00; 0.00) | 0.00 (0.00; 0.00) | 44.69 (44; 45.38) | 0.00 (0.00; 0.00) | 0.00 (0.00; 0.00) | 26.33 (25.98; 26.69) | 0.00 (0.00; 0.00) | 0.00 (0.00; 0.00) | 26.88 (26.53; 27.23) | 0.00 (0.00; 0.00) | 0.00 (0.00; 0.00) | 72.42 (71.98; 72.86) |
| **United States of America** | 0.03 (0.03; 0.03) | 0.03 (0.03; 0.04) | 1.67 (1.57; 1.76) | 0.01 (0.01; 0.01) | 0.01 (0.01; 0.01) | 2.72 (2.61; 2.84) | 2.84 (2.11; 3.75) | 2.93 (2.16; 3.83) | 2.29 (1.88; 2.70) | 31.57 (30.11; 32.66) | 30.73 (27.62; 33.79) | -2.93 (-3.04; -2.83) | 34.41 (32.80; 35.81) | 33.65 (30.24; 36.96) | -2.50 (-2.61; -2.39) | 1.34 (1.24; 1.40) | 1.37 (1.20; 1.52) | 1.22 (1.09; 1.35) | 0.18 (0.17; 0.19) | 0.23 (0.20; 0.26) | 25.06 (24.90; 25.22) | 0.03 (0.03; 0.03) | 0.05 (0.04; 0.05) | 36.32 (36.12; 36.51) | 0.02 (0.01; 0.02) | 0.02 (0.02; 0.03) | 29.71 (29.24; 30.18) | 0.17 (0.16; 0.18) | 0.21 (0.19; 0.24) | 23.61 (23.43; 23.78) | 0.19 (0.18; 0.20) | 0.23 (0.21; 0.26) | 24.12 (23.94; 24.29) | 0.01 (0.01; 0.01) | 0.01 (0.01; 0.01) | 49.75 (49.50; 49.99) |
| **Southern Latin America** | 0.01 (0.01; 0.01) | 0.01 (0.01; 0.01) | 11.37 (11.22; 11.53) | 0.00 (0.00; 0.00) | 0.00 (0.00; 0.00) | 4.85 (4.70; 5.00) | 1.02 (0.73; 1.38) | 1.16 (0.82; 1.54) | 12.75 (12.28; 13.22) | 30.74 (28.08; 33.61) | 29.49 (26.26; 33.45) | -3.23 (-3.38; -3.08) | 31.76 (28.82; 34.80) | 30.64 (27.44; 34.74) | -2.71 (-2.86; -2.56) | 1.23 (1.12; 1.35) | 1.23 (1.10; 1.39) | 0.13 (-0.01; 0.28) | 0.01 (0.01; 0.01) | 0.01 (0.01; 0.02) | 71.24 (70.94; 71.54) | 0.00 (0.00; 0.00) | 0.00 (0.00; 0.00) | 73.96 (73.67; 74.26) | 0.00 (0.00; 0.00) | 0.00 (0.00; 0.00) | 82.38 (81.69; 83.06) | 0.03 (0.02; 0.03) | 0.04 (0.03; 0.05) | 54.47 (54.19; 54.75) | 0.03 (0.02; 0.03) | 0.04 (0.04; 0.05) | 55.38 (55.11; 55.66) | 0.00 (0.00; 0.00) | 0.00 (0.00; 0.00) | 78.99 (78.69; 79.28) |
| **Argentina** | 0.01 (0.01; 0.01) | 0.01 (0.01; 0.01) | 11.96 (11.75; 12.16) | 0.00 (0.00; 0.00) | 0.00 (0.00; 0.00) | 6.06 (5.87; 6.25) | 1.06 (0.73; 1.43) | 1.21 (0.84; 1.65) | 13.40 (12.90; 13.90) | 34.37 (30.74; 38.29) | 33.39 (28.82; 38.85) | -2.18 (-2.37; -1.99) | 35.43 (31.57; 39.52) | 34.60 (29.96; 40.23) | -1.71 (-1.90; -1.53) | 1.37 (1.23; 1.52) | 1.40 (1.22; 1.61) | 2 (1.82; 2.18) | 0.01 (0.00; 0.01) | 0.01 (0.01; 0.01) | 73.21 (72.79; 73.62) | 0.00 (0.00; 0.00) | 0.00 (0.00; 0.00) | 73.96 (73.56; 74.36) | 0.00 (0.00; 0.00) | 0.00 (0.00; 0.00) | 82.68 (81.91; 83.44) | 0.02 (0.02; 0.02) | 0.03 (0.02; 0.04) | 56.05 (55.69; 56.41) | 0.02 (0.02; 0.02) | 0.03 (0.02; 0.04) | 56.86 (56.50; 57.23) | 0.00 (0.00; 0.00) | 0.00 (0.00; 0.00) | 76.13 (75.75; 76.51) |
| **Chile** | 0.01 (0.01; 0.01) | 0.01 (0.01; 0.01) | 14.25 (14.06; 14.43) | 0.00 (0.00; 0.00) | 0.00 (0.00; 0.00) | 7.01 (6.85; 7.18) | 0.78 (0.56; 1.06) | 0.90 (0.65; 1.24) | 15.89 (15.39; 16.40) | 18.13 (16.58; 19.89) | 17.55 (15.34; 20.09) | -2.50 (-2.66; -2.34) | 18.91 (17.32; 20.72) | 18.45 (16.16; 21.01) | -1.74 (-1.90; -1.58) | 0.74 (0.67; 0.81) | 0.75 (0.66; 0.86) | 1.99 (1.83; 2.16) | 0.00 (0.00; 0.00) | 0.00 (0.00; 0.00) | 82.91 (82.60; 83.23) | 0.00 (0.00; 0.00) | 0.00 (0.00; 0.00) | 94.62 (94.29; 94.94) | 0.00 (0.00; 0.00) | 0.00 (0.00; 0.00) | 101.54 (100.79; 102.29) | 0.00 (0.00; 0.00) | 0.01 (0.01; 0.01) | 67.65 (67.36; 67.95) | 0.00 (0.00; 0.01) | 0.01 (0.01; 0.01) | 69.06 (68.78; 69.34) | 0.00 (0.00; 0.00) | 0.00 (0.00; 0.00) | 116.26 (115.87; 116.64) |
| **Uruguay** | 0.02 (0.02; 0.02) | 0.02 (0.02; 0.02) | 9.43 (9.24; 9.61) | 0.00 (0.00; 0.01) | 0.01 (0.00; 0.01) | 4.57 (4.39; 4.74) | 1.90 (1.33; 2.56) | 2.11 (1.47; 2.87) | 10.81 (10.32; 11.30) | 56.35 (50.76; 62.20) | 54.24 (47.56; 62.36) | -3.10 (-3.27; -2.93) | 58.25 (52.69; 64.33) | 56.35 (49.61; 64.67) | -2.64 (-2.81; -2.47) | 2.22 (2.00; 2.44) | 2.26 (2.00; 2.57) | 1.63 (1.47; 1.79) | 0.00 (0.00; 0.00) | 0.00 (0.00; 0.00) | 34.04 (33.73; 34.34) | 0.00 (0.00; 0.00) | 0.00 (0.00; 0.00) | 34.49 (34.19; 34.79) | 0.00 (0.00; 0.00) | 0.00 (0.00; 0.00) | 39.94 (39.35; 40.54) | 0.00 (0.00; 0.00) | 0.00 (0.00; 0.00) | 21.59 (21.31; 21.87) | 0.00 (0.00; 0.00) | 0.00 (0.00; 0.00) | 22.20 (21.91; 22.49) | 0.00 (0.00; 0.00) | 0.00 (0.00; 0.00) | 37.16 (36.86; 37.46) |
| **Western Europe** | 0.03 (0.03; 0.03) | 0.03 (0.03; 0.03) | -1.38 (-1.47; -1.30) | 0.01 (0.01; 0.01) | 0.01 (0.00; 0.01) | -5.32 (-5.41; -5.23) | 2.62 (1.91; 3.44) | 2.60 (1.88; 3.40) | -0.50 (-0.91; -0.09) | 38.86 (36.71; 40.69) | 33.33 (30.89; 35.80) | -13.46 (-13.55; -13.38) | 41.47 (39.12; 43.47) | 35.93 (33.30; 38.44) | -12.64 (-12.72; -12.56) | 1.56 (1.44; 1.64) | 1.41 (1.29; 1.52) | -9.15 (-9.25; -9.05) | 0.23 (0.21; 0.24) | 0.27 (0.24; 0.29) | 15.71 (15.59; 15.84) | 0.05 (0.04; 0.05) | 0.06 (0.05; 0.06) | 18.79 (18.66; 18.93) | 0.02 (0.02; 0.03) | 0.03 (0.02; 0.03) | 21.34 (20.90; 21.78) | 0.31 (0.29; 0.32) | 0.32 (0.29; 0.35) | 4.56 (4.44; 4.67) | 0.33 (0.30; 0.34) | 0.35 (0.31; 0.37) | 5.63 (5.52; 5.75) | 0.01 (0.01; 0.02) | 0.02 (0.02; 0.02) | 27.79 (27.62; 27.95) |
| **Andorra** | 0.01 (0.01; 0.02) | 0.01 (0.01; 0.02) | -14.02 (-14.56; -13.48) | 0.00 (0.00; 0.00) | 0.00 (0.00; 0.00) | -13.69 (-14.22; -13.15) | 1.20 (0.70; 1.96) | 1.03 (0.59; 1.71) | -13.51 (-14.21; -12.81) | 20.19 (13.07; 29.55) | 17.00 (10.83; 25.45) | -15.35 (-15.90; -14.79) | 21.40 (13.64; 31.21) | 18.04 (11.56; 26.44) | -15.24 (-15.78; -14.71) | 0.74 (0.48; 1.08) | 0.65 (0.42; 0.96) | -12.28 (-12.82; -11.75) | 0.00 (0.00; 0.00) | 0.00 (0.00; 0.00) | 0.18 (-0.41; 0.76) | 0.00 (0.00; 0.00) | 0.00 (0.00; 0.00) | 18.15 (17.53; 18.77) | 0.00 (0.00; 0.00) | 0.00 (0.00; 0.00) | 10.93 (10.16; 11.71) | 0.00 (0.00; 0.00) | 0.00 (0.00; 0.00) | 1.37 (0.81; 1.93) | 0.00 (0.00; 0.00) | 0.00 (0.00; 0.00) | 1.91 (1.37; 2.45) | 0.00 (0.00; 0.00) | 0.00 (0.00; 0.00) | 44.48 (43.87; 45.09) |
| **Austria** | 0.02 (0.02; 0.02) | 0.02 (0.01; 0.02) | -7.58 (-7.75; -7.42) | 0.00 (0.00; 0.00) | 0.00 (0.00; 0.00) | -10.59 (-10.75; -10.42) | 1.74 (1.24; 2.39) | 1.62 (1.14; 2.25) | -6.29 (-6.75; -5.83) | 41.35 (37.34; 45.45) | 33.91 (28.87; 39.68) | -16.83 (-16.99; -16.66) | 43.09 (38.87; 47.22) | 35.53 (30.38; 41.48) | -16.40 (-16.56; -16.23) | 1.64 (1.47; 1.80) | 1.42 (1.22; 1.64) | -12.74 (-12.90; -12.57) | 0.00 (0.00; 0.00) | 0.00 (0.00; 0.00) | 17.73 (17.50; 17.97) | 0.00 (0.00; 0.00) | 0.00 (0.00; 0.00) | 24.25 (24.02; 24.49) | 0.00 (0.00; 0.00) | 0.00 (0.00; 0.00) | 25.33 (24.77; 25.88) | 0.01 (0.01; 0.01) | 0.01 (0.01; 0.01) | 8.80 (8.58; 9.01) | 0.01 (0.01; 0.01) | 0.01 (0.01; 0.01) | 9.48 (9.27; 9.69) | 0.00 (0.00; 0.00) | 0.00 (0.00; 0.00) | 34.22 (33.97; 34.47) |
| **Belgium** | 0.03 (0.03; 0.03) | 0.03 (0.02; 0.03) | -10.85 (-11; -10.69) | 0.01 (0.00; 0.01) | 0.00 (0.00; 0.01) | -13.03 (-13.18; -12.88) | 2.53 (1.81; 3.35) | 2.27 (1.61; 3.09) | -9.86 (-10.28; -9.44) | 44.50 (40.67; 48.70) | 36.48 (31.62; 41.86) | -17.17 (-17.32; -17.03) | 47.04 (43.22; 51.63) | 38.75 (33.71; 44.35) | -16.78 (-16.92; -16.63) | 1.70 (1.55; 1.85) | 1.46 (1.28; 1.66) | -13.82 (-13.97; -13.68) | 0.01 (0.00; 0.01) | 0.01 (0.01; 0.01) | 6.43 (6.22; 6.64) | 0.00 (0.00; 0.00) | 0.00 (0.00; 0.00) | 10.40 (10.18; 10.62) | 0.00 (0.00; 0.00) | 0.00 (0.00; 0.00) | 11.81 (11.31; 12.30) | 0.01 (0.01; 0.01) | 0.01 (0.01; 0.01) | 1.55 (1.34; 1.76) | 0.01 (0.01; 0.01) | 0.01 (0.01; 0.01) | 2.11 (1.91; 2.31) | 0.00 (0.00; 0.00) | 0.00 (0.00; 0.00) | 20.54 (20.31; 20.77) |
| **Cyprus** | 0.01 (0.01; 0.02) | 0.01 (0.01; 0.02) | 0.52 (0.15; 0.89) | 0.00 (0.00; 0.00) | 0.00 (0.00; 0.00) | -2.73 (-3.07; -2.39) | 1.33 (0.87; 1.83) | 1.35 (0.88; 1.89) | 1.59 (1.07; 2.12) | 22.42 (17.56; 28.32) | 20.17 (15.32; 25.64) | -9.66 (-9.99; -9.33) | 23.77 (18.40; 30.04) | 21.52 (16.67; 27.14) | -9.03 (-9.34; -8.72) | 0.97 (0.78; 1.21) | 0.92 (0.72; 1.14) | -5.95 (-6.26; -5.64) | 0.00 (0.00; 0.00) | 0.00 (0.00; 0.00) | 61.55 (61; 62.10) | 0.00 (0.00; 0.00) | 0.00 (0.00; 0.00) | 69.99 (69.46; 70.52) | 0.00 (0.00; 0.00) | 0.00 (0.00; 0.00) | 71.58 (70.82; 72.34) | 0.00 (0.00; 0.00) | 0.00 (0.00; 0.00) | 51.32 (50.84; 51.80) | 0.00 (0.00; 0.00) | 0.00 (0.00; 0.00) | 52.51 (52.04; 52.97) | 0.00 (0.00; 0.00) | 0.00 (0.00; 0.00) | 85.20 (84.68; 85.73) |
| **Denmark** | 0.02 (0.02; 0.02) | 0.02 (0.02; 0.02) | -12.41 (-12.54; -12.27) | 0.00 (0.00; 0.00) | 0.00 (0.00; 0.00) | -14.28 (-14.42; -14.15) | 2.08 (1.51; 2.79) | 1.83 (1.33; 2.51) | -11.35 (-11.77; -10.93) | 36.30 (33.92; 38.80) | 28.85 (25.77; 32.73) | -19.56 (-19.68; -19.44) | 38.39 (35.98; 40.91) | 30.68 (27.32; 34.81) | -19.11 (-19.23; -19) | 1.50 (1.38; 1.60) | 1.27 (1.13; 1.42) | -15.11 (-15.23; -14.98) | 0.00 (0.00; 0.00) | 0.00 (0.00; 0.00) | 3.46 (3.26; 3.66) | 0.00 (0.00; 0.00) | 0.00 (0.00; 0.00) | 7.85 (7.64; 8.05) | 0.00 (0.00; 0.00) | 0.00 (0.00; 0.00) | 8.28 (7.80; 8.76) | 0.00 (0.00; 0.00) | 0.00 (0.00; 0.00) | -1.49 (-1.68; -1.31) | 0.00 (0.00; 0.00) | 0.00 (0.00; 0.00) | -0.95 (-1.13; -0.76) | 0.00 (0.00; 0.00) | 0.00 (0.00; 0.00) | 16.73 (16.51; 16.96) |
| **Finland** | 0.02 (0.02; 0.02) | 0.02 (0.02; 0.02) | 1.50 (1.35; 1.66) | 0.00 (0.00; 0.00) | 0.00 (0.00; 0.00) | -1.37 (-1.52; -1.21) | 1.76 (1.29; 2.33) | 1.81 (1.30; 2.47) | 2.71 (2.27; 3.16) | 28.41 (26.63; 30.18) | 25.95 (22.90; 29.15) | -8.33 (-8.46; -8.20) | 30.17 (28.29; 32.05) | 27.76 (24.35; 31.18) | -7.69 (-7.82; -7.55) | 1.13 (1.03; 1.21) | 1.10 (0.95; 1.24) | -3.03 (-3.18; -2.88) | 0.00 (0.00; 0.00) | 0.00 (0.00; 0.00) | 10.48 (10.26; 10.70) | 0.00 (0.00; 0.00) | 0.00 (0.00; 0.00) | 16.73 (16.49; 16.97) | 0.00 (0.00; 0.00) | 0.00 (0.00; 0.00) | 16.55 (16.06; 17.04) | 0.00 (0.00; 0.00) | 0.00 (0.00; 0.00) | 7.29 (7.07; 7.50) | 0.00 (0.00; 0.00) | 0.00 (0.00; 0.00) | 7.85 (7.64; 8.05) | 0.00 (0.00; 0.00) | 0.00 (0.00; 0.00) | 32.23 (31.93; 32.53) |
| **France** | 0.05 (0.04; 0.05) | 0.04 (0.04; 0.05) | -1.51 (-1.67; -1.36) | 0.01 (0.01; 0.01) | 0.01 (0.01; 0.01) | -5.62 (-5.79; -5.46) | 3.91 (2.82; 5.34) | 3.88 (2.80; 5.37) | -0.67 (-1.14; -0.21) | 46.34 (41.79; 51.16) | 40.16 (34.54; 46.34) | -12.51 (-12.68; -12.35) | 50.25 (45.35; 55.39) | 44.04 (38.25; 50.43) | -11.59 (-11.74; -11.43) | 1.77 (1.58; 1.95) | 1.60 (1.38; 1.82) | -9.34 (-9.50; -9.18) | 0.05 (0.05; 0.06) | 0.06 (0.05; 0.06) | 9.86 (9.63; 10.08) | 0.01 (0.01; 0.01) | 0.01 (0.01; 0.01) | 11.74 (11.50; 11.98) | 0.00 (0.00; 0.01) | 0.01 (0.00; 0.01) | 14.97 (14.48; 15.46) | 0.05 (0.05; 0.06) | 0.05 (0.04; 0.06) | -0.47 (-0.69; -0.24) | 0.06 (0.05; 0.06) | 0.06 (0.05; 0.07) | 0.75 (0.53; 0.96) | 0.00 (0.00; 0.00) | 0.00 (0.00; 0.00) | 22.60 (22.33; 22.87) |
| **Germany** | 0.02 (0.02; 0.03) | 0.02 (0.02; 0.03) | 0.90 (0.72; 1.08) | 0.00 (0.00; 0.01) | 0.00 (0.00; 0.01) | -1.45 (-1.63; -1.27) | 2.19 (1.52; 2.92) | 2.24 (1.57; 3.05) | 2.16 (1.69; 2.62) | 40.08 (35.92; 44.33) | 36.84 (31.74; 43.08) | -7.48 (-7.66; -7.30) | 42.28 (38.17; 47.14) | 39.08 (33.71; 45.79) | -6.98 (-7.16; -6.80) | 1.58 (1.41; 1.75) | 1.53 (1.32; 1.78) | -3.26 (-3.44; -3.09) | 0.04 (0.03; 0.04) | 0.04 (0.03; 0.05) | 6.37 (6.13; 6.60) | 0.01 (0.01; 0.01) | 0.01 (0.01; 0.01) | 10.93 (10.69; 11.17) | 0.00 (0.00; 0.00) | 0.00 (0.00; 0.01) | 12.01 (11.53; 12.49) | 0.06 (0.06; 0.07) | 0.07 (0.06; 0.08) | 0.75 (0.53; 0.97) | 0.07 (0.06; 0.08) | 0.07 (0.06; 0.08) | 1.35 (1.14; 1.56) | 0.00 (0.00; 0.00) | 0.00 (0.00; 0.00) | 18.78 (18.53; 19.02) |
| **Greece** | 0.02 (0.02; 0.02) | 0.02 (0.02; 0.02) | -5.61 (-5.75; -5.47) | 0.00 (0.00; 0.00) | 0.00 (0.00; 0.00) | -5.86 (-5.99; -5.72) | 1.78 (1.29; 2.39) | 1.69 (1.20; 2.28) | -4.82 (-5.25; -4.39) | 32.95 (31.01; 34.83) | 30.29 (26.81; 34.14) | -7.93 (-8.06; -7.80) | 34.72 (32.75; 36.89) | 31.98 (28.31; 36.03) | -7.77 (-7.90; -7.65) | 1.31 (1.22; 1.38) | 1.25 (1.11; 1.40) | -4.43 (-4.56; -4.30) | 0.00 (0.00; 0.00) | 0.00 (0.00; 0.00) | -8.39 (-8.62; -8.17) | 0.00 (0.00; 0.00) | 0.00 (0.00; 0.00) | -0.91 (-1.15; -0.67) | 0.00 (0.00; 0.00) | 0.00 (0.00; 0.00) | -2.74 (-3.21; -2.27) | 0.01 (0.01; 0.01) | 0.01 (0.00; 0.01) | -8.68 (-8.90; -8.46) | 0.01 (0.01; 0.01) | 0.01 (0.01; 0.01) | -8.37 (-8.59; -8.15) | 0.00 (0.00; 0.00) | 0.00 (0.00; 0.00) | 11.24 (10.97; 11.50) |
| **Iceland** | 0.03 (0.02; 0.03) | 0.03 (0.02; 0.03) | -2.45 (-2.63; -2.28) | 0.00 (0.00; 0.01) | 0.00 (0.00; 0.01) | -2.61 (-2.79; -2.44) | 2.28 (1.68; 3.15) | 2.23 (1.62; 3.07) | -1.50 (-1.95; -1.05) | 32.18 (28.89; 35.39) | 30.37 (26.87; 35.02) | -5.11 (-5.27; -4.95) | 34.45 (31.00; 38.37) | 32.60 (28.80; 37.33) | -4.87 (-5.03; -4.72) | 1.32 (1.17; 1.45) | 1.30 (1.12; 1.47) | -1.55 (-1.72; -1.38) | 0.00 (0.00; 0.00) | 0.00 (0.00; 0.00) | 53.05 (52.76; 53.35) | 0.00 (0.00; 0.00) | 0.00 (0.00; 0.00) | 65.72 (65.40; 66.04) | 0.00 (0.00; 0.00) | 0.00 (0.00; 0.00) | 60.31 (59.69; 60.92) | 0.00 (0.00; 0.00) | 0.00 (0.00; 0.00) | 55.62 (55.35; 55.90) | 0.00 (0.00; 0.00) | 0.00 (0.00; 0.00) | 55.94 (55.66; 56.21) | 0.00 (0.00; 0.00) | 0.00 (0.00; 0.00) | 90.80 (90.43; 91.16) |
| **Ireland** | 0.02 (0.02; 0.03) | 0.02 (0.02; 0.02) | -13.60 (-13.74; -13.45) | 0.00 (0.00; 0.00) | 0.00 (0.00; 0.00) | -15.86 (-16.01; -15.72) | 2.12 (1.55; 2.84) | 1.83 (1.33; 2.51) | -12.58 (-13; -12.17) | 32.24 (29.28; 35.15) | 25.04 (22.01; 28.16) | -21.01 (-21.14; -20.88) | 34.36 (31.22; 37.36) | 26.87 (23.80; 30.18) | -20.48 (-20.61; -20.36) | 1.31 (1.18; 1.43) | 1.07 (0.93; 1.21) | -17.68 (-17.82; -17.53) | 0.00 (0.00; 0.00) | 0.00 (0.00; 0.00) | 32.58 (32.33; 32.84) | 0.00 (0.00; 0.00) | 0.00 (0.00; 0.00) | 41.59 (41.31; 41.86) | 0.00 (0.00; 0.00) | 0.00 (0.00; 0.00) | 41.39 (40.79; 41.98) | 0.00 (0.00; 0.00) | 0.00 (0.00; 0.00) | 25.25 (25.01; 25.49) | 0.00 (0.00; 0.00) | 0.00 (0.00; 0.00) | 26.26 (26.03; 26.50) | 0.00 (0.00; 0.00) | 0.00 (0.00; 0.00) | 61.64 (61.32; 61.97) |
| **Israel** | 0.01 (0.01; 0.01) | 0.01 (0.01; 0.01) | 7.47 (7.30; 7.64) | 0.00 (0.00; 0.00) | 0.00 (0.00; 0.00) | 1.45 (1.29; 1.62) | 1.05 (0.74; 1.44) | 1.14 (0.80; 1.55) | 8.58 (8.09; 9.07) | 19.19 (17.45; 20.99) | 17.73 (15.63; 20.15) | -6.69 (-6.84; -6.55) | 20.24 (18.41; 22.03) | 18.86 (16.63; 21.42) | -5.90 (-6.05; -5.75) | 0.81 (0.72; 0.88) | 0.77 (0.67; 0.87) | -4.00 (-4.16; -3.84) | 0.00 (0.00; 0.00) | 0.00 (0.00; 0.00) | 78.36 (77.99; 78.73) | 0.00 (0.00; 0.00) | 0.00 (0.00; 0.00) | 79.60 (79.21; 79.99) | 0.00 (0.00; 0.00) | 0.00 (0.00; 0.00) | 88.18 (87.45; 88.92) | 0.00 (0.00; 0.00) | 0.00 (0.00; 0.00) | 59.85 (59.49; 60.21) | 0.00 (0.00; 0.00) | 0.00 (0.00; 0.00) | 61.35 (61; 61.71) | 0.00 (0.00; 0.00) | 0.00 (0.00; 0.00) | 89.35 (88.92; 89.77) |
| **Italy** | 0.02 (0.02; 0.02) | 0.02 (0.02; 0.02) | 0.96 (0.82; 1.10) | 0.00 (0.00; 0.00) | 0.00 (0.00; 0.00) | -4.33 (-4.47; -4.18) | 1.88 (1.37; 2.53) | 1.92 (1.37; 2.58) | 2.53 (2.08; 2.98) | 36.14 (33.87; 38.08) | 31.22 (27.68; 35.25) | -12.88 (-13.01; -12.76) | 38.02 (35.59; 40.04) | 33.15 (29.45; 37.41) | -12.12 (-12.24; -12) | 1.52 (1.38; 1.61) | 1.40 (1.24; 1.58) | -7.84 (-7.98; -7.70) | 0.02 (0.02; 0.03) | 0.02 (0.02; 0.03) | 6.52 (6.32; 6.72) | 0.01 (0.01; 0.01) | 0.01 (0.01; 0.01) | 12.57 (12.34; 12.79) | 0.00 (0.00; 0.00) | 0.00 (0.00; 0.00) | 15.42 (14.96; 15.87) | 0.04 (0.04; 0.05) | 0.04 (0.04; 0.05) | -1.94 (-2.13; -1.76) | 0.05 (0.04; 0.05) | 0.05 (0.04; 0.05) | -1.08 (-1.26; -0.90) | 0.00 (0.00; 0.00) | 0.00 (0.00; 0.00) | 24.19 (23.93; 24.45) |
| **Luxembourg** | 0.03 (0.02; 0.03) | 0.02 (0.02; 0.03) | -2.26 (-2.44; -2.07) | 0.00 (0.00; 0.01) | 0.00 (0.00; 0.01) | -4.49 (-4.66; -4.32) | 2.22 (1.56; 2.97) | 2.19 (1.51; 2.93) | -1.17 (-1.62; -0.71) | 37.55 (33.70; 41.42) | 33.81 (29.38; 39.11) | -9.44 (-9.61; -9.28) | 39.76 (35.54; 44.01) | 36.00 (31.28; 41.44) | -8.98 (-9.14; -8.81) | 1.51 (1.35; 1.66) | 1.45 (1.25; 1.63) | -4.72 (-4.88; -4.55) | 0.00 (0.00; 0.00) | 0.00 (0.00; 0.00) | 67.02 (66.72; 67.31) | 0.00 (0.00; 0.00) | 0.00 (0.00; 0.00) | 73.48 (73.19; 73.78) | 0.00 (0.00; 0.00) | 0.00 (0.00; 0.00) | 75.25 (74.58; 75.91) | 0.00 (0.00; 0.00) | 0.00 (0.00; 0.00) | 56.86 (56.60; 57.12) | 0.00 (0.00; 0.00) | 0.00 (0.00; 0.00) | 57.89 (57.64; 58.15) | 0.00 (0.00; 0.00) | 0.00 (0.00; 0.00) | 89.75 (89.44; 90.06) |
| **Malta** | 0.02 (0.02; 0.02) | 0.02 (0.02; 0.03) | 12.47 (12.25; 12.69) | 0.00 (0.00; 0.00) | 0.00 (0.00; 0.01) | 8.63 (8.42; 8.84) | 1.87 (1.33; 2.53) | 2.12 (1.48; 2.91) | 13.11 (12.61; 13.61) | 32.47 (28.56; 36.69) | 32.49 (27.64; 38.70) | 0.51 (0.30; 0.72) | 34.33 (30.05; 38.47) | 34.61 (29.36; 40.92) | 1.20 (1.00; 1.41) | 1.27 (1.11; 1.43) | 1.32 (1.12; 1.54) | 3.84 (3.63; 4.05) | 0.00 (0.00; 0.00) | 0.00 (0.00; 0.00) | 38.66 (38.33; 38.98) | 0.00 (0.00; 0.00) | 0.00 (0.00; 0.00) | 44.11 (43.78; 44.43) | 0.00 (0.00; 0.00) | 0.00 (0.00; 0.00) | 44.91 (44.28; 45.54) | 0.00 (0.00; 0.00) | 0.00 (0.00; 0.00) | 31.93 (31.64; 32.22) | 0.00 (0.00; 0.00) | 0.00 (0.00; 0.00) | 32.67 (32.37; 32.96) | 0.00 (0.00; 0.00) | 0.00 (0.00; 0.00) | 57.54 (57.18; 57.89) |
| **Monaco** | 0.01 (0.01; 0.02) | 0.01 (0.01; 0.02) | 0.25 (-0.16; 0.66) | 0.00 (0.00; 0.00) | 0.00 (0.00; 0.00) | 0.72 (0.32; 1.11) | 1.31 (0.87; 1.91) | 1.33 (0.88; 1.98) | 0.88 (0.31; 1.46) | 21.08 (16.08; 27.48) | 20.48 (15.49; 26.56) | -2.92 (-3.29; -2.56) | 22.40 (17.09; 29.26) | 21.81 (16.80; 27.94) | -2.70 (-3.04; -2.36) | 0.81 (0.63; 1.03) | 0.82 (0.64; 1.04) | 1.30 (0.96; 1.64) | 0.00 (0.00; 0.00) | 0.00 (0.00; 0.00) | -17.16 (-17.52; -16.80) | 0.00 (0.00; 0.00) | 0.00 (0.00; 0.00) | -6.73 (-7.10; -6.36) | 0.00 (0.00; 0.00) | 0.00 (0.00; 0.00) | -10.37 (-10.92; -9.82) | 0.00 (0.00; 0.00) | 0.00 (0.00; 0.00) | -15.66 (-15.98; -15.35) | 0.00 (0.00; 0.00) | 0.00 (0.00; 0.00) | -15.34 (-15.65; -15.03) | 0.00 (0.00; 0.00) | 0.00 (0.00; 0.00) | 7.10 (6.76; 7.45) |
| **Netherlands** | 0.02 (0.02; 0.02) | 0.02 (0.02; 0.02) | -2.52 (-2.66; -2.38) | 0.00 (0.00; 0.00) | 0.00 (0.00; 0.00) | -4.67 (-4.82; -4.53) | 1.84 (1.31; 2.44) | 1.81 (1.30; 2.42) | -1.67 (-2.10; -1.24) | 28.93 (26.75; 30.82) | 25.81 (23.15; 28.76) | -10.42 (-10.54; -10.29) | 30.77 (28.45; 32.83) | 27.62 (24.74; 30.73) | -9.89 (-10.01; -9.77) | 1.28 (1.15; 1.37) | 1.21 (1.07; 1.35) | -5.60 (-5.73; -5.46) | 0.01 (0.01; 0.01) | 0.01 (0.01; 0.01) | 16.41 (16.18; 16.65) | 0.00 (0.00; 0.00) | 0.00 (0.00; 0.00) | 23.42 (23.18; 23.67) | 0.00 (0.00; 0.00) | 0.00 (0.00; 0.00) | 22.58 (22.03; 23.13) | 0.01 (0.01; 0.01) | 0.01 (0.01; 0.01) | 11.30 (11.08; 11.51) | 0.01 (0.01; 0.01) | 0.01 (0.01; 0.01) | 11.99 (11.78; 12.20) | 0.00 (0.00; 0.00) | 0.00 (0.00; 0.00) | 36.86 (36.58; 37.15) |
| **Norway** | 0.02 (0.02; 0.02) | 0.02 (0.02; 0.02) | -6.58 (-6.71; -6.46) | 0.00 (0.00; 0.00) | 0.00 (0.00; 0.00) | -6.27 (-6.40; -6.14) | 1.69 (1.25; 2.23) | 1.60 (1.15; 2.13) | -5.33 (-5.74; -4.92) | 24.67 (23.18; 25.74) | 22.02 (19.81; 24.43) | -10.29 (-10.40; -10.18) | 26.36 (24.69; 27.59) | 23.61 (21.22; 26.30) | -9.97 (-10.08; -9.85) | 1.12 (1.02; 1.18) | 1.07 (0.93; 1.20) | -4.41 (-4.55; -4.27) | 0.00 (0.00; 0.00) | 0.00 (0.00; 0.00) | 33.96 (33.74; 34.19) | 0.00 (0.00; 0.00) | 0.00 (0.00; 0.00) | 44.77 (44.53; 45.02) | 0.00 (0.00; 0.00) | 0.00 (0.00; 0.00) | 40.70 (40.17; 41.23) | 0.00 (0.00; 0.00) | 0.00 (0.00; 0.00) | 34.27 (34.05; 34.49) | 0.00 (0.00; 0.00) | 0.00 (0.00; 0.00) | 34.69 (34.46; 34.91) | 0.00 (0.00; 0.00) | 0.00 (0.00; 0.00) | 62.69 (62.39; 62.98) |
| **Portugal** | 0.04 (0.03; 0.04) | 0.04 (0.04; 0.05) | 12.42 (12.23; 12.61) | 0.01 (0.01; 0.01) | 0.01 (0.01; 0.01) | 6.68 (6.49; 6.87) | 3.22 (2.26; 4.30) | 3.65 (2.52; 4.96) | 13.02 (12.53; 13.51) | 55.81 (49.97; 62.38) | 50.35 (42.25; 59.26) | -8.98 (-9.17; -8.78) | 59.03 (52.47; 65.67) | 54.00 (45.60; 62.81) | -7.76 (-7.95; -7.58) | 2.04 (1.82; 2.29) | 1.90 (1.61; 2.18) | -6.73 (-6.91; -6.54) | 0.01 (0.01; 0.01) | 0.01 (0.01; 0.01) | 24.02 (23.73; 24.31) | 0.00 (0.00; 0.00) | 0.00 (0.00; 0.00) | 23.71 (23.42; 24) | 0.00 (0.00; 0.00) | 0.00 (0.00; 0.00) | 29.99 (29.42; 30.56) | 0.01 (0.01; 0.01) | 0.01 (0.01; 0.01) | -3.19 (-3.45; -2.93) | 0.01 (0.01; 0.01) | 0.01 (0.01; 0.01) | -1.25 (-1.51; -1) | 0.00 (0.00; 0.00) | 0.00 (0.00; 0.00) | 19.49 (19.20; 19.78) |
| **San Marino** | 0.02 (0.01; 0.03) | 0.02 (0.01; 0.02) | -0.87 (-1.55; -0.19) | 0.00 (0.00; 0.00) | 0.00 (0.00; 0.00) | -1.63 (-2.29; -0.97) | 1.45 (0.79; 2.40) | 1.45 (0.80; 2.43) | -0.35 (-1.15; 0.44) | 22.74 (13.33; 34.77) | 22.20 (13.00; 34.93) | -3.08 (-3.76; -2.39) | 24.20 (13.94; 37.70) | 23.65 (14.45; 36.30) | -2.91 (-3.56; -2.27) | 0.89 (0.54; 1.34) | 0.90 (0.55; 1.38) | 0.32 (-0.34; 0.97) | 0.00 (0.00; 0.00) | 0.00 (0.00; 0.00) | 11.41 (10.69; 12.12) | 0.00 (0.00; 0.00) | 0.00 (0.00; 0.00) | 30.66 (29.91; 31.40) | 0.00 (0.00; 0.00) | 0.00 (0.00; 0.00) | 24.34 (23.45; 25.23) | 0.00 (0.00; 0.00) | 0.00 (0.00; 0.00) | 19 (18.32; 19.69) | 0.00 (0.00; 0.00) | 0.00 (0.00; 0.00) | 19.33 (18.69; 19.96) | 0.00 (0.00; 0.00) | 0.00 (0.00; 0.00) | 68.26 (67.54; 68.99) |
| **Spain** | 0.04 (0.04; 0.05) | 0.04 (0.04; 0.05) | -7.22 (-7.39; -7.06) | 0.01 (0.01; 0.01) | 0.01 (0.01; 0.01) | -11.61 (-11.78; -11.45) | 3.69 (2.61; 4.92) | 3.45 (2.44; 4.62) | -6.36 (-6.79; -5.93) | 39.11 (35.89; 42.30) | 30.49 (26.53; 34.73) | -20.93 (-21.06; -20.79) | 42.81 (39.02; 46.59) | 33.94 (29.82; 38.39) | -19.66 (-19.79; -19.53) | 1.58 (1.43; 1.72) | 1.33 (1.15; 1.51) | -15.51 (-15.66; -15.36) | 0.04 (0.03; 0.04) | 0.05 (0.04; 0.06) | 24.22 (23.96; 24.48) | 0.01 (0.01; 0.01) | 0.01 (0.01; 0.01) | 26.44 (26.16; 26.72) | 0.00 (0.00; 0.00) | 0.00 (0.00; 0.01) | 29.22 (28.69; 29.75) | 0.03 (0.03; 0.04) | 0.04 (0.03; 0.04) | 5.89 (5.68; 6.10) | 0.04 (0.03; 0.04) | 0.04 (0.03; 0.05) | 7.97 (7.76; 8.18) | 0.00 (0.00; 0.00) | 0.00 (0.00; 0.00) | 33.76 (33.48; 34.04) |
| **Sweden** | 0.02 (0.02; 0.02) | 0.02 (0.02; 0.02) | -2.60 (-2.77; -2.42) | 0.00 (0.00; 0.00) | 0.00 (0.00; 0.00) | -2.63 (-2.81; -2.46) | 1.80 (1.30; 2.43) | 1.77 (1.26; 2.40) | -1.29 (-1.73; -0.84) | 26.89 (23.99; 30.04) | 25.39 (22.14; 28.66) | -5.12 (-5.29; -4.96) | 28.69 (25.67; 32.09) | 27.16 (23.83; 30.57) | -4.88 (-5.04; -4.72) | 1.19 (1.05; 1.33) | 1.19 (1.02; 1.34) | -0.46 (-0.64; -0.29) | 0.00 (0.00; 0.00) | 0.00 (0.00; 0.01) | 30.78 (30.52; 31.05) | 0.00 (0.00; 0.00) | 0.00 (0.00; 0.00) | 38.60 (38.32; 38.88) | 0.00 (0.00; 0.00) | 0.00 (0.00; 0.00) | 36.01 (35.45; 36.56) | 0.01 (0.00; 0.01) | 0.01 (0.01; 0.01) | 33.39 (33.14; 33.65) | 0.01 (0.00; 0.01) | 0.01 (0.01; 0.01) | 33.56 (33.31; 33.81) | 0.00 (0.00; 0.00) | 0.00 (0.00; 0.00) | 53.18 (52.86; 53.50) |
| **Switzerland** | 0.02 (0.02; 0.02) | 0.02 (0.01; 0.02) | -12.87 (-13.05; -12.70) | 0.00 (0.00; 0.00) | 0.00 (0.00; 0.00) | -12.52 (-12.70; -12.35) | 1.75 (1.24; 2.38) | 1.52 (1.06; 2.13) | -12 (-12.45; -11.56) | 28.02 (24.93; 31.47) | 23.65 (20.28; 28.17) | -14.63 (-14.82; -14.44) | 29.77 (26.32; 33.33) | 25.18 (21.73; 29.82) | -14.47 (-14.66; -14.29) | 1.18 (1.04; 1.32) | 1.06 (0.91; 1.23) | -10.12 (-10.31; -9.94) | 0.00 (0.00; 0.00) | 0.00 (0.00; 0.00) | 19.15 (18.89; 19.41) | 0.00 (0.00; 0.00) | 0.00 (0.00; 0.00) | 31.32 (31.02; 31.61) | 0.00 (0.00; 0.00) | 0.00 (0.00; 0.00) | 26.33 (25.78; 26.89) | 0.00 (0.00; 0.01) | 0.01 (0.00; 0.01) | 22.55 (22.27; 22.83) | 0.00 (0.00; 0.01) | 0.01 (0.00; 0.01) | 22.77 (22.51; 23.04) | 0.00 (0.00; 0.00) | 0.00 (0.00; 0.00) | 52.74 (52.39; 53.09) |
| **United Kingdom** | 0.03 (0.03; 0.04) | 0.03 (0.03; 0.04) | 0.07 (-0.03; 0.18) | 0.01 (0.01; 0.01) | 0.01 (0.01; 0.01) | -3.94 (-4.04; -3.83) | 2.96 (2.17; 3.93) | 2.98 (2.20; 3.86) | 1.00 (0.59; 1.41) | 40.16 (38.62; 41.31) | 33.87 (30.43; 37.47) | -15.02 (-15.11; -14.92) | 43.12 (41.20; 44.65) | 36.86 (32.96; 40.90) | -13.91 (-14.01; -13.81) | 1.64 (1.54; 1.70) | 1.46 (1.30; 1.61) | -10.76 (-10.87; -10.65) | 0.04 (0.04; 0.04) | 0.05 (0.04; 0.05) | 26.42 (26.27; 26.57) | 0.01 (0.01; 0.01) | 0.01 (0.01; 0.01) | 28.57 (28.41; 28.73) | 0.00 (0.00; 0.00) | 0.00 (0.00; 0.01) | 31.96 (31.50; 32.42) | 0.05 (0.04; 0.05) | 0.05 (0.04; 0.06) | 12.28 (12.13; 12.43) | 0.05 (0.05; 0.05) | 0.06 (0.05; 0.06) | 13.64 (13.49; 13.79) | 0.00 (0.00; 0.00) | 0.00 (0.00; 0.00) | 35.06 (34.87; 35.26) |
| **Latin America and Caribbean** | 0.01 (0.01; 0.01) | 0.01 (0.01; 0.01) | 8.58 (8.44; 8.73) | 0.00 (0.00; 0.00) | 0.00 (0.00; 0.00) | 1.90 (1.76; 2.04) | 1.02 (0.74; 1.34) | 1.12 (0.79; 1.50) | 10.38 (9.92; 10.83) | 40.86 (38.32; 43.37) | 38.56 (34.15; 43.70) | -5.26 (-5.40; -5.13) | 41.88 (39.23; 44.30) | 39.69 (35.02; 45.08) | -4.88 (-5.02; -4.74) | 1.61 (1.50; 1.72) | 1.58 (1.39; 1.80) | -2.47 (-2.61; -2.33) | 0.06 (0.05; 0.06) | 0.11 (0.09; 0.12) | 82.18 (81.93; 82.42) | 0.02 (0.02; 0.02) | 0.03 (0.03; 0.04) | 91.86 (91.59; 92.13) | 0.01 (0.00; 0.01) | 0.01 (0.01; 0.02) | 98.96 (98.26; 99.65) | 0.26 (0.24; 0.28) | 0.43 (0.38; 0.50) | 64.45 (64.21; 64.69) | 0.27 (0.25; 0.28) | 0.45 (0.40; 0.51) | 65.29 (65.04; 65.53) | 0.01 (0.01; 0.01) | 0.02 (0.02; 0.02) | 101.10 (100.80; 101.40) |
| **Andean Latin America** | 0.01 (0.00; 0.01) | 0.01 (0.01; 0.01) | 26.44 (26.06; 26.82) | 0.00 (0.00; 0.00) | 0.00 (0.00; 0.00) | 14.29 (13.94; 14.63) | 0.59 (0.39; 0.83) | 0.77 (0.51; 1.06) | 28.08 (27.49; 28.67) | 21.96 (17.61; 27.14) | 22.70 (18.02; 28.63) | 3.64 (3.32; 3.96) | 22.55 (17.89; 27.68) | 23.46 (18.82; 29.28) | 4.29 (3.97; 4.60) | 0.92 (0.74; 1.13) | 0.99 (0.78; 1.23) | 6.39 (6.07; 6.71) | 0.00 (0.00; 0.00) | 0.01 (0.01; 0.01) | 142.69 (142.01; 143.37) | 0.00 (0.00; 0.00) | 0.00 (0.00; 0.00) | 145.78 (145.11; 146.45) | 0.00 (0.00; 0.00) | 0.00 (0.00; 0.00) | 165.70 (164.62; 166.78) | 0.01 (0.01; 0.02) | 0.03 (0.02; 0.04) | 107.67 (107.17; 108.17) | 0.01 (0.01; 0.02) | 0.03 (0.02; 0.04) | 109.20 (108.70; 109.69) | 0.00 (0.00; 0.00) | 0.00 (0.00; 0.00) | 149.90 (149.35; 150.45) |
| **Bolivia (Plurinational State of)** | 0.00 (0.00; 0.01) | 0.01 (0.00; 0.01) | 31.86 (31.23; 32.49) | 0.00 (0.00; 0.00) | 0.00 (0.00; 0.00) | 18.97 (18.40; 19.54) | 0.62 (0.38; 0.96) | 0.84 (0.52; 1.29) | 34.35 (33.58; 35.13) | 30.88 (21.27; 43.66) | 33.26 (22.75; 47.84) | 7.37 (6.83; 7.91) | 31.50 (21.29; 43.75) | 34.10 (23.74; 48.68) | 7.90 (7.37; 8.43) | 1.28 (0.88; 1.81) | 1.46 (0.99; 2.10) | 11.99 (11.43; 12.56) | 0.00 (0.00; 0.00) | 0.00 (0.00; 0.00) | 159.66 (158.50; 160.82) | 0.00 (0.00; 0.00) | 0.00 (0.00; 0.00) | 154.13 (152.98; 155.28) | 0.00 (0.00; 0.00) | 0.00 (0.00; 0.00) | 180.59 (179.14; 182.04) | 0.00 (0.00; 0.00) | 0.01 (0.00; 0.01) | 117.71 (116.83; 118.60) | 0.00 (0.00; 0.00) | 0.01 (0.00; 0.01) | 118.93 (118.05; 119.80) | 0.00 (0.00; 0.00) | 0.00 (0.00; 0.00) | 149.23 (148.25; 150.21) |
| **Ecuador** | 0.00 (0.00; 0.01) | 0.01 (0.00; 0.01) | 27.65 (27.23; 28.08) | 0.00 (0.00; 0.00) | 0.00 (0.00; 0.00) | 15.17 (14.80; 15.55) | 0.55 (0.36; 0.77) | 0.72 (0.47; 1.02) | 28.85 (28.23; 29.48) | 21.37 (16.53; 27.13) | 22.08 (16.80; 28.48) | 3.66 (3.29; 4.03) | 21.91 (17.07; 27.69) | 22.80 (17.41; 29.21) | 4.30 (3.94; 4.67) | 0.92 (0.73; 1.15) | 0.99 (0.77; 1.26) | 6.88 (6.54; 7.23) | 0.00 (0.00; 0.00) | 0.00 (0.00; 0.00) | 144.45 (143.69; 145.21) | 0.00 (0.00; 0.00) | 0.00 (0.00; 0.00) | 147.60 (146.83; 148.36) | 0.00 (0.00; 0.00) | 0.00 (0.00; 0.00) | 166.55 (165.40; 167.70) | 0.00 (0.00; 0.00) | 0.01 (0.01; 0.01) | 109.17 (108.59; 109.74) | 0.00 (0.00; 0.00) | 0.01 (0.01; 0.01) | 110.63 (110.06; 111.20) | 0.00 (0.00; 0.00) | 0.00 (0.00; 0.00) | 150.81 (150.19; 151.42) |
| **Peru** | 0.01 (0.00; 0.01) | 0.01 (0.00; 0.01) | 24.72 (24.23; 25.21) | 0.00 (0.00; 0.00) | 0.00 (0.00; 0.00) | 12.23 (11.78; 12.68) | 0.61 (0.38; 0.89) | 0.77 (0.47; 1.15) | 25.80 (25.10; 26.50) | 19.81 (14.31; 26.51) | 19.97 (13.73; 27.57) | 1.07 (0.61; 1.53) | 20.42 (14.73; 26.79) | 20.74 (14.54; 28.59) | 1.81 (1.36; 2.27) | 0.83 (0.60; 1.10) | 0.87 (0.62; 1.21) | 3.96 (3.51; 4.42) | 0.00 (0.00; 0.00) | 0.00 (0.00; 0.01) | 137.84 (136.98; 138.70) | 0.00 (0.00; 0.00) | 0.00 (0.00; 0.00) | 142.34 (141.46; 143.22) | 0.00 (0.00; 0.00) | 0.00 (0.00; 0.00) | 161.16 (159.90; 162.43) | 0.01 (0.00; 0.01) | 0.01 (0.01; 0.02) | 102.51 (101.79; 103.22) | 0.01 (0.01; 0.01) | 0.01 (0.01; 0.02) | 104.25 (103.55; 104.96) | 0.00 (0.00; 0.00) | 0.00 (0.00; 0.00) | 149.70 (148.90; 150.49) |
| **Caribbean** | 0.01 (0.01; 0.02) | 0.01 (0.01; 0.01) | -5.27 (-5.49; -5.04) | 0.00 (0.00; 0.00) | 0.00 (0.00; 0.00) | -2.94 (-3.17; -2.71) | 1.46 (1.05; 1.91) | 1.45 (1.02; 1.95) | -0.58 (-1.01; -0.14) | 55.13 (47.31; 64.07) | 53.45 (44.50; 65.29) | -2.82 (-3.06; -2.58) | 56.57 (48.30; 66.02) | 54.90 (45.81; 66.97) | -2.76 (-3.00; -2.53) | 2.22 (1.93; 2.55) | 2.23 (1.87; 2.67) | -0.01 (-0.24; 0.22) | 0.01 (0.01; 0.01) | 0.01 (0.01; 0.01) | 41.97 (41.66; 42.28) | 0.00 (0.00; 0.00) | 0.00 (0.00; 0.00) | 59.51 (59.18; 59.85) | 0.00 (0.00; 0.00) | 0.00 (0.00; 0.00) | 56.99 (56.41; 57.58) | 0.03 (0.03; 0.03) | 0.04 (0.04; 0.05) | 46.90 (46.61; 47.19) | 0.03 (0.03; 0.04) | 0.05 (0.04; 0.05) | 47.16 (46.88; 47.45) | 0.00 (0.00; 0.00) | 0.00 (0.00; 0.00) | 75.05 (74.76; 75.34) |
| **Antigua and Barbuda** | 0.01 (0.01; 0.01) | 0.01 (0.01; 0.01) | 14.66 (14.42; 14.90) | 0.00 (0.00; 0.00) | 0.00 (0.00; 0.00) | 11.01 (10.77; 11.25) | 1.01 (0.72; 1.37) | 1.16 (0.80; 1.60) | 15.66 (15.15; 16.17) | 37.00 (33.25; 40.75) | 39.01 (32.12; 46.47) | 6.09 (5.87; 6.31) | 38.01 (34.26; 41.86) | 40.17 (32.98; 47.78) | 6.35 (6.13; 6.57) | 1.60 (1.44; 1.77) | 1.75 (1.44; 2.07) | 8.94 (8.72; 9.16) | 0.00 (0.00; 0.00) | 0.00 (0.00; 0.00) | 82.24 (81.80; 82.67) | 0.00 (0.00; 0.00) | 0.00 (0.00; 0.00) | 96.35 (95.88; 96.82) | 0.00 (0.00; 0.00) | 0.00 (0.00; 0.00) | 95.75 (94.92; 96.57) | 0.00 (0.00; 0.00) | 0.00 (0.00; 0.00) | 72.51 (72.09; 72.93) | 0.00 (0.00; 0.00) | 0.00 (0.00; 0.00) | 73.13 (72.70; 73.55) | 0.00 (0.00; 0.00) | 0.00 (0.00; 0.00) | 105.95 (105.46; 106.44) |
| **Bahamas** | 0.01 (0.01; 0.02) | 0.01 (0.01; 0.02) | -5.86 (-6.22; -5.51) | 0.00 (0.00; 0.01) | 0.00 (0.00; 0.01) | -6.53 (-6.87; -6.19) | 1.62 (1.06; 2.29) | 1.54 (1.01; 2.25) | -4.73 (-5.27; -4.19) | 71.07 (55.76; 89.21) | 62.87 (47.47; 81.39) | -10.59 (-10.93; -10.25) | 72.67 (57.35; 91.61) | 64.41 (48.88; 82.70) | -10.46 (-10.79; -10.12) | 2.75 (2.19; 3.39) | 2.55 (1.95; 3.21) | -6.88 (-7.20; -6.56) | 0.00 (0.00; 0.00) | 0.00 (0.00; 0.00) | 52.86 (52.33; 53.40) | 0.00 (0.00; 0.00) | 0.00 (0.00; 0.00) | 73.65 (73.06; 74.23) | 0.00 (0.00; 0.00) | 0.00 (0.00; 0.00) | 67.73 (66.91; 68.55) | 0.00 (0.00; 0.00) | 0.00 (0.00; 0.00) | 49.32 (48.87; 49.77) | 0.00 (0.00; 0.00) | 0.00 (0.00; 0.00) | 49.73 (49.29; 50.17) | 0.00 (0.00; 0.00) | 0.00 (0.00; 0.00) | 89.28 (88.79; 89.77) |
| **Barbados** | 0.01 (0.01; 0.01) | 0.01 (0.01; 0.02) | 16.47 (16.04; 16.89) | 0.00 (0.00; 0.00) | 0.00 (0.00; 0.00) | 10.28 (9.89; 10.67) | 1.25 (0.83; 1.81) | 1.48 (0.95; 2.15) | 17.69 (17.08; 18.31) | 44.50 (34.28; 56.57) | 46.01 (34.09; 60.07) | 3.71 (3.32; 4.09) | 45.77 (35.29; 58.40) | 47.49 (35.34; 61.75) | 4.09 (3.71; 4.47) | 1.92 (1.51; 2.39) | 2.05 (1.55; 2.58) | 6.45 (6.09; 6.80) | 0.00 (0.00; 0.00) | 0.00 (0.00; 0.00) | 32.62 (32.10; 33.13) | 0.00 (0.00; 0.00) | 0.00 (0.00; 0.00) | 39.69 (39.18; 40.21) | 0.00 (0.00; 0.00) | 0.00 (0.00; 0.00) | 42.84 (42.09; 43.60) | 0.00 (0.00; 0.00) | 0.00 (0.00; 0.00) | 21.55 (21.15; 21.95) | 0.00 (0.00; 0.00) | 0.00 (0.00; 0.00) | 22.14 (21.76; 22.53) | 0.00 (0.00; 0.00) | 0.00 (0.00; 0.00) | 45.24 (44.85; 45.63) |
| **Belize** | 0.01 (0.00; 0.01) | 0.01 (0.01; 0.01) | 20.56 (20.30; 20.82) | 0.00 (0.00; 0.00) | 0.00 (0.00; 0.00) | 15.18 (14.93; 15.43) | 0.63 (0.44; 0.84) | 0.77 (0.52; 1.09) | 20.64 (20.09; 21.18) | 27.97 (24.02; 31.99) | 31.26 (25.95; 38.25) | 11.52 (11.26; 11.78) | 28.60 (24.75; 32.72) | 32.03 (26.57; 39.13) | 11.72 (11.46; 11.98) | 1.12 (0.98; 1.27) | 1.25 (1.04; 1.52) | 11.22 (10.97; 11.47) | 0.00 (0.00; 0.00) | 0.00 (0.00; 0.00) | 191.67 (190.98; 192.36) | 0.00 (0.00; 0.00) | 0.00 (0.00; 0.00) | 205.91 (205.23; 206.60) | 0.00 (0.00; 0.00) | 0.00 (0.00; 0.00) | 210.68 (209.51; 211.85) | 0.00 (0.00; 0.00) | 0.00 (0.00; 0.00) | 177.86 (177.24; 178.48) | 0.00 (0.00; 0.00) | 0.00 (0.00; 0.00) | 178.57 (177.95; 179.19) | 0.00 (0.00; 0.00) | 0.00 (0.00; 0.00) | 215.18 (214.52; 215.84) |
| **Bermuda** | 0.02 (0.02; 0.03) | 0.02 (0.02; 0.03) | -3.58 (-3.90; -3.26) | 0.00 (0.00; 0.01) | 0.00 (0.00; 0.01) | -8.01 (-8.31; -7.71) | 2.11 (1.44; 2.93) | 2.04 (1.36; 2.89) | -2.18 (-2.69; -1.67) | 46.39 (38.08; 56.29) | 38.49 (29.98; 48.56) | -15.54 (-15.83; -15.26) | 48.52 (39.70; 60.17) | 40.53 (31.57; 50.75) | -14.96 (-15.24; -14.68) | 2.00 (1.67; 2.42) | 1.76 (1.36; 2.19) | -11.01 (-11.30; -10.72) | 0.00 (0.00; 0.00) | 0.00 (0.00; 0.00) | 11.33 (10.94; 11.72) | 0.00 (0.00; 0.00) | 0.00 (0.00; 0.00) | 25.19 (24.76; 25.62) | 0.00 (0.00; 0.00) | 0.00 (0.00; 0.00) | 22.86 (22.23; 23.49) | 0.00 (0.00; 0.00) | 0.00 (0.00; 0.00) | 4.67 (4.35; 4.99) | 0.00 (0.00; 0.00) | 0.00 (0.00; 0.00) | 5.48 (5.17; 5.79) | 0.00 (0.00; 0.00) | 0.00 (0.00; 0.00) | 44.10 (43.72; 44.48) |
| **Cuba** | 0.02 (0.02; 0.02) | 0.02 (0.02; 0.03) | 11.90 (11.60; 12.20) | 0.01 (0.00; 0.01) | 0.01 (0.00; 0.01) | 7.47 (7.18; 7.75) | 2.19 (1.53; 2.96) | 2.48 (1.69; 3.46) | 12.73 (12.21; 13.24) | 67.93 (56.96; 80.11) | 69.83 (54.74; 87.90) | 3.20 (2.91; 3.50) | 70.10 (59.10; 82.10) | 72.31 (56.97; 90.75) | 3.50 (3.21; 3.79) | 2.76 (2.34; 3.22) | 2.89 (2.34; 3.56) | 4.22 (3.95; 4.49) | 0.00 (0.00; 0.00) | 0.00 (0.00; 0.01) | 22.44 (22.08; 22.79) | 0.00 (0.00; 0.00) | 0.00 (0.00; 0.00) | 33.67 (33.31; 34.03) | 0.00 (0.00; 0.00) | 0.00 (0.00; 0.00) | 32.40 (31.79; 33) | 0.01 (0.01; 0.02) | 0.02 (0.01; 0.02) | 15.49 (15.16; 15.81) | 0.01 (0.01; 0.02) | 0.02 (0.01; 0.02) | 16.02 (15.69; 16.34) | 0.00 (0.00; 0.00) | 0.00 (0.00; 0.00) | 43.90 (43.54; 44.26) |
| **Dominica** | 0.01 (0.01; 0.01) | 0.01 (0.01; 0.01) | -1.65 (-2.04; -1.26) | 0.00 (0.00; 0.01) | 0.00 (0.00; 0.01) | 1.16 (0.80; 1.52) | 1.38 (0.90; 2.00) | 1.39 (0.87; 2.02) | 0.31 (-0.26; 0.88) | 68.27 (52.87; 87.29) | 66.48 (49.39; 86.67) | -2.73 (-3.09; -2.36) | 69.62 (53.26; 88.15) | 67.88 (50.65; 87.95) | -2.67 (-3.03; -2.31) | 2.84 (2.23; 3.52) | 2.96 (2.23; 3.84) | 3.16 (2.80; 3.53) | 0.00 (0.00; 0.00) | 0.00 (0.00; 0.00) | 31.83 (31.33; 32.34) | 0.00 (0.00; 0.00) | 0.00 (0.00; 0.00) | 47.28 (46.75; 47.82) | 0.00 (0.00; 0.00) | 0.00 (0.00; 0.00) | 40.89 (40.15; 41.63) | 0.00 (0.00; 0.00) | 0.00 (0.00; 0.00) | 33.10 (32.64; 33.56) | 0.00 (0.00; 0.00) | 0.00 (0.00; 0.00) | 33.26 (32.80; 33.72) | 0.00 (0.00; 0.00) | 0.00 (0.00; 0.00) | 56.63 (56.13; 57.12) |
| **Dominican Republic** | 0.01 (0.01; 0.01) | 0.01 (0.01; 0.02) | 15.67 (15.12; 16.21) | 0.00 (0.00; 0.01) | 0.00 (0.00; 0.01) | 9.49 (8.97; 10.02) | 1.23 (0.76; 1.85) | 1.43 (0.89; 2.15) | 15.80 (15.12; 16.49) | 58.20 (41.48; 83.52) | 60.17 (42.17; 86.44) | 2.65 (2.14; 3.16) | 59.38 (42.23; 85.51) | 61.60 (43.30; 88.03) | 2.92 (2.41; 3.43) | 2.41 (1.74; 3.41) | 2.56 (1.81; 3.58) | 4.72 (4.23; 5.22) | 0.00 (0.00; 0.00) | 0.00 (0.00; 0.00) | 109.20 (108.28; 110.12) | 0.00 (0.00; 0.00) | 0.00 (0.00; 0.00) | 125.09 (124.14; 126.03) | 0.00 (0.00; 0.00) | 0.00 (0.00; 0.00) | 125.38 (124.22; 126.54) | 0.01 (0.00; 0.01) | 0.01 (0.01; 0.02) | 95.30 (94.48; 96.11) | 0.01 (0.00; 0.01) | 0.01 (0.01; 0.02) | 95.91 (95.11; 96.71) | 0.00 (0.00; 0.00) | 0.00 (0.00; 0.00) | 133.67 (132.82; 134.53) |
| **Grenada** | 0.01 (0.01; 0.01) | 0.01 (0.01; 0.01) | 4.57 (4.28; 4.85) | 0.00 (0.00; 0.00) | 0.00 (0.00; 0.00) | 5.45 (5.16; 5.73) | 1.27 (0.85; 1.75) | 1.37 (0.90; 1.97) | 7.42 (6.87; 7.98) | 58.10 (48.15; 69.65) | 57.66 (44.23; 73.66) | -0.09 (-0.40; 0.23) | 59.37 (49.30; 71.09) | 59.04 (45.44; 75.07) | 0.08 (-0.23; 0.39) | 2.30 (1.93; 2.73) | 2.46 (1.91; 3.10) | 6.29 (5.98; 6.60) | 0.00 (0.00; 0.00) | 0.00 (0.00; 0.00) | 43.66 (43.25; 44.07) | 0.00 (0.00; 0.00) | 0.00 (0.00; 0.00) | 60.06 (59.60; 60.51) | 0.00 (0.00; 0.00) | 0.00 (0.00; 0.00) | 57.21 (56.46; 57.97) | 0.00 (0.00; 0.00) | 0.00 (0.00; 0.00) | 40.73 (40.32; 41.14) | 0.00 (0.00; 0.00) | 0.00 (0.00; 0.00) | 41.08 (40.68; 41.49) | 0.00 (0.00; 0.00) | 0.00 (0.00; 0.00) | 70.18 (69.73; 70.63) |
| **Guyana** | 0.00 (0.00; 0.01) | 0.01 (0.00; 0.01) | 5.16 (4.76; 5.56) | 0.00 (0.00; 0.00) | 0.00 (0.00; 0.00) | 2.81 (2.43; 3.19) | 0.68 (0.45; 0.99) | 0.73 (0.47; 1.05) | 6.14 (5.56; 6.72) | 38.46 (28.78; 50.06) | 37.78 (27.30; 49.55) | -1.58 (-1.98; -1.17) | 39.14 (29.45; 50.77) | 38.51 (28.18; 50.45) | -1.44 (-1.84; -1.04) | 1.44 (1.09; 1.84) | 1.46 (1.09; 1.91) | 1 (0.61; 1.39) | 0.00 (0.00; 0.00) | 0.00 (0.00; 0.00) | 31.35 (30.80; 31.91) | 0.00 (0.00; 0.00) | 0.00 (0.00; 0.00) | 50 (49.42; 50.59) | 0.00 (0.00; 0.00) | 0.00 (0.00; 0.00) | 48.01 (47.27; 48.76) | 0.00 (0.00; 0.00) | 0.00 (0.00; 0.00) | 27.65 (27.19; 28.12) | 0.00 (0.00; 0.00) | 0.00 (0.00; 0.00) | 28 (27.54; 28.46) | 0.00 (0.00; 0.00) | 0.00 (0.00; 0.00) | 60.69 (60.21; 61.16) |
| **Haiti** | 0.01 (0.00; 0.01) | 0.01 (0.00; 0.01) | 0.45 (-0.12; 1.03) | 0.00 (0.00; 0.00) | 0.00 (0.00; 0.00) | -6.00 (-6.54; -5.46) | 0.82 (0.49; 1.25) | 0.84 (0.48; 1.29) | 1.77 (1.10; 2.45) | 56.71 (38.29; 83.17) | 49.65 (32.54; 73.94) | -11.68 (-12.21; -11.15) | 57.52 (38.71; 84.05) | 50.49 (33.42; 74.74) | -11.49 (-12.01; -10.96) | 2.29 (1.57; 3.29) | 2.08 (1.37; 3.03) | -9.11 (-9.62; -8.60) | 0.00 (0.00; 0.00) | 0.00 (0.00; 0.00) | 113.13 (112.06; 114.20) | 0.00 (0.00; 0.00) | 0.00 (0.00; 0.00) | 119.63 (118.56; 120.69) | 0.00 (0.00; 0.00) | 0.00 (0.00; 0.00) | 130.61 (129.29; 131.94) | 0.00 (0.00; 0.01) | 0.01 (0.01; 0.01) | 91.53 (90.69; 92.38) | 0.00 (0.00; 0.01) | 0.01 (0.01; 0.01) | 92.07 (91.23; 92.91) | 0.00 (0.00; 0.00) | 0.00 (0.00; 0.00) | 119.29 (118.38; 120.21) |
| **Jamaica** | 0.01 (0.00; 0.01) | 0.01 (0.01; 0.01) | 9.80 (9.35; 10.24) | 0.00 (0.00; 0.00) | 0.00 (0.00; 0.00) | 7.91 (7.47; 8.34) | 0.75 (0.50; 1.09) | 0.84 (0.55; 1.22) | 10.36 (9.76; 10.95) | 28.25 (21.08; 37.68) | 29.30 (21.30; 39.41) | 3.60 (3.16; 4.03) | 28.99 (22.11; 39.26) | 30.14 (22.06; 40.13) | 3.77 (3.35; 4.20) | 1.18 (0.90; 1.54) | 1.27 (0.95; 1.68) | 6.19 (5.77; 6.60) | 0.00 (0.00; 0.00) | 0.00 (0.00; 0.00) | 77.05 (76.37; 77.74) | 0.00 (0.00; 0.00) | 0.00 (0.00; 0.00) | 88.51 (87.81; 89.21) | 0.00 (0.00; 0.00) | 0.00 (0.00; 0.00) | 88.03 (87.14; 88.93) | 0.00 (0.00; 0.00) | 0.00 (0.00; 0.00) | 72.03 (71.47; 72.58) | 0.00 (0.00; 0.00) | 0.00 (0.00; 0.00) | 72.44 (71.89; 72.99) | 0.00 (0.00; 0.00) | 0.00 (0.00; 0.00) | 94.37 (93.86; 94.87) |
| **Puerto Rico** | 0.01 (0.01; 0.02) | 0.01 (0.01; 0.02) | 0.65 (0.33; 0.97) | 0.00 (0.00; 0.00) | 0.00 (0.00; 0.00) | -2.27 (-2.58; -1.96) | 1.24 (0.88; 1.72) | 1.26 (0.86; 1.80) | 1.54 (1.03; 2.06) | 31.93 (25.57; 39.07) | 29.27 (22.90; 36.91) | -7.37 (-7.67; -7.07) | 33.16 (26.56; 40.38) | 30.53 (24.11; 38.11) | -7.04 (-7.33; -6.75) | 1.27 (1.03; 1.55) | 1.20 (0.95; 1.49) | -4.70 (-4.99; -4.41) | 0.00 (0.00; 0.00) | 0.00 (0.00; 0.00) | -0.09 (-0.42; 0.24) | 0.00 (0.00; 0.00) | 0.00 (0.00; 0.00) | 10.87 (10.53; 11.21) | 0.00 (0.00; 0.00) | 0.00 (0.00; 0.00) | 8.50 (7.96; 9.04) | 0.00 (0.00; 0.00) | 0.00 (0.00; 0.00) | -1.43 (-1.73; -1.14) | 0.00 (0.00; 0.00) | 0.00 (0.00; 0.00) | -1.05 (-1.34; -0.76) | 0.00 (0.00; 0.00) | 0.00 (0.00; 0.00) | 25.57 (25.25; 25.89) |
| **Saint Kitts and Nevis** | 0.01 (0.01; 0.01) | 0.01 (0.01; 0.01) | -10.47 (-10.77; -10.18) | 0.00 (0.00; 0.00) | 0.00 (0.00; 0.00) | -7.93 (-8.21; -7.65) | 1.09 (0.75; 1.54) | 1.00 (0.68; 1.44) | -7.56 (-8.06; -7.06) | 47.54 (38.44; 57.15) | 39.98 (31.54; 49.32) | -14.79 (-15.06; -14.52) | 48.62 (39.63; 57.73) | 40.98 (32.48; 50.37) | -14.63 (-14.89; -14.36) | 2.01 (1.65; 2.36) | 1.84 (1.47; 2.25) | -8.02 (-8.28; -7.76) | 0.00 (0.00; 0.00) | 0.00 (0.00; 0.00) | 37.85 (37.36; 38.35) | 0.00 (0.00; 0.00) | 0.00 (0.00; 0.00) | 63.38 (62.82; 63.93) | 0.00 (0.00; 0.00) | 0.00 (0.00; 0.00) | 54.81 (54.04; 55.59) | 0.00 (0.00; 0.00) | 0.00 (0.00; 0.00) | 34.31 (33.85; 34.77) | 0.00 (0.00; 0.00) | 0.00 (0.00; 0.00) | 34.76 (34.31; 35.21) | 0.00 (0.00; 0.00) | 0.00 (0.00; 0.00) | 76.51 (75.98; 77.05) |
| **Saint Lucia** | 0.01 (0.01; 0.02) | 0.01 (0.01; 0.02) | 1.35 (1.02; 1.69) | 0.00 (0.00; 0.01) | 0.00 (0.00; 0.01) | -3.15 (-3.47; -2.84) | 1.46 (1.00; 2.03) | 1.49 (1.00; 2.09) | 2.26 (1.74; 2.77) | 63.96 (51.53; 78.28) | 57.95 (44.31; 73.86) | -8.11 (-8.43; -7.79) | 65.41 (53.06; 79.66) | 59.44 (45.79; 75.49) | -7.87 (-8.19; -7.56) | 2.60 (2.11; 3.14) | 2.43 (1.87; 3.08) | -5.80 (-6.11; -5.49) | 0.00 (0.00; 0.00) | 0.00 (0.00; 0.00) | 46.67 (46.20; 47.13) | 0.00 (0.00; 0.00) | 0.00 (0.00; 0.00) | 61.57 (61.06; 62.08) | 0.00 (0.00; 0.00) | 0.00 (0.00; 0.00) | 60.74 (60.04; 61.44) | 0.00 (0.00; 0.00) | 0.00 (0.00; 0.00) | 36.92 (36.52; 37.33) | 0.00 (0.00; 0.00) | 0.00 (0.00; 0.00) | 37.46 (37.06; 37.86) | 0.00 (0.00; 0.00) | 0.00 (0.00; 0.00) | 72.14 (71.69; 72.59) |
| **Saint Vincent and the Grenadines** | 0.02 (0.01; 0.02) | 0.02 (0.01; 0.02) | 16.75 (16.47; 17.02) | 0.01 (0.00; 0.01) | 0.01 (0.01; 0.01) | 14.77 (14.50; 15.04) | 1.83 (1.29; 2.47) | 2.16 (1.48; 3.02) | 17.10 (16.57; 17.62) | 90.49 (77.36; 105.75) | 104.84 (84.51; 131.08) | 14.78 (14.48; 15.08) | 92.30 (79.41; 107.84) | 107.00 (86.61; 133.53) | 14.83 (14.53; 15.13) | 3.59 (3.12; 4.15) | 4.21 (3.45; 5.15) | 15.87 (15.60; 16.15) | 0.00 (0.00; 0.00) | 0.00 (0.00; 0.00) | 39.83 (39.42; 40.24) | 0.00 (0.00; 0.00) | 0.00 (0.00; 0.00) | 54.89 (54.47; 55.32) | 0.00 (0.00; 0.00) | 0.00 (0.00; 0.00) | 50.25 (49.57; 50.92) | 0.00 (0.00; 0.00) | 0.00 (0.00; 0.00) | 40.68 (40.30; 41.06) | 0.00 (0.00; 0.00) | 0.00 (0.00; 0.00) | 40.87 (40.49; 41.25) | 0.00 (0.00; 0.00) | 0.00 (0.00; 0.00) | 66.95 (66.54; 67.37) |
| **Suriname** | 0.00 (0.00; 0.01) | 0.00 (0.00; 0.01) | 4.26 (3.78; 4.74) | 0.00 (0.00; 0.00) | 0.00 (0.00; 0.00) | 1.65 (1.18; 2.13) | 0.61 (0.40; 0.93) | 0.65 (0.41; 1.00) | 5.62 (4.97; 6.27) | 30.94 (22.43; 42.65) | 29.68 (20.93; 41.62) | -4.14 (-4.61; -3.68) | 31.55 (23.14; 43.53) | 30.33 (21.61; 42.34) | -3.95 (-4.41; -3.50) | 1.21 (0.87; 1.67) | 1.20 (0.85; 1.68) | -0.94 (-1.41; -0.47) | 0.00 (0.00; 0.00) | 0.00 (0.00; 0.00) | 55.35 (54.68; 56.02) | 0.00 (0.00; 0.00) | 0.00 (0.00; 0.00) | 74.46 (73.72; 75.21) | 0.00 (0.00; 0.00) | 0.00 (0.00; 0.00) | 73.25 (72.34; 74.15) | 0.00 (0.00; 0.00) | 0.00 (0.00; 0.00) | 47.07 (46.47; 47.67) | 0.00 (0.00; 0.00) | 0.00 (0.00; 0.00) | 47.57 (46.98; 48.15) | 0.00 (0.00; 0.00) | 0.00 (0.00; 0.00) | 84.12 (83.47; 84.77) |
| **Trinidad and Tobago** | 0.01 (0.01; 0.01) | 0.01 (0.00; 0.01) | 1.85 (1.44; 2.26) | 0.00 (0.00; 0.00) | 0.00 (0.00; 0.00) | -1.22 (-1.61; -0.83) | 0.81 (0.54; 1.17) | 0.83 (0.54; 1.24) | 3.31 (2.73; 3.89) | 34.05 (25.63; 44.40) | 31.62 (23.73; 42.12) | -6.34 (-6.72; -5.97) | 34.85 (26.32; 44.90) | 32.45 (24.51; 42.95) | -6.12 (-6.49; -5.75) | 1.32 (1.01; 1.69) | 1.27 (0.94; 1.69) | -3.10 (-3.47; -2.72) | 0.00 (0.00; 0.00) | 0.00 (0.00; 0.00) | 17.90 (17.41; 18.40) | 0.00 (0.00; 0.00) | 0.00 (0.00; 0.00) | 35.30 (34.76; 35.84) | 0.00 (0.00; 0.00) | 0.00 (0.00; 0.00) | 34.15 (33.39; 34.91) | 0.00 (0.00; 0.00) | 0.00 (0.00; 0.00) | 14.33 (13.90; 14.75) | 0.00 (0.00; 0.00) | 0.00 (0.00; 0.00) | 14.79 (14.38; 15.21) | 0.00 (0.00; 0.00) | 0.00 (0.00; 0.00) | 49.52 (49.07; 49.98) |
| **United States Virgin Islands** | 0.01 (0.00; 0.01) | 0.01 (0.00; 0.01) | 0.07 (-0.37; 0.51) | 0.00 (0.00; 0.00) | 0.00 (0.00; 0.00) | 1.92 (1.44; 2.40) | 0.73 (0.47; 1.12) | 0.76 (0.46; 1.17) | 3.47 (2.81; 4.13) | 28.12 (20.24; 38.08) | 27.38 (19.66; 37.26) | -2.66 (-3.10; -2.22) | 28.83 (20.93; 39.08) | 28.14 (20.38; 38.16) | -2.51 (-2.94; -2.07) | 1.07 (0.76; 1.45) | 1.11 (0.79; 1.49) | 2.85 (2.41; 3.30) | 0.00 (0.00; 0.00) | 0.00 (0.00; 0.00) | -8.80 (-9.27; -8.33) | 0.00 (0.00; 0.00) | 0.00 (0.00; 0.00) | 21.84 (21.27; 22.41) | 0.00 (0.00; 0.00) | 0.00 (0.00; 0.00) | 9.77 (9.03; 10.50) | 0.00 (0.00; 0.00) | 0.00 (0.00; 0.00) | 1.30 (0.85; 1.76) | 0.00 (0.00; 0.00) | 0.00 (0.00; 0.00) | 1.53 (1.09; 1.97) | 0.00 (0.00; 0.00) | 0.00 (0.00; 0.00) | 48.96 (48.40; 49.52) |
| **Central Latin America** | 0.01 (0.01; 0.01) | 0.01 (0.01; 0.01) | 15.47 (15.27; 15.67) | 0.00 (0.00; 0.00) | 0.00 (0.00; 0.00) | 7.91 (7.72; 8.10) | 0.66 (0.47; 0.90) | 0.78 (0.55; 1.05) | 16.86 (16.36; 17.36) | 24.47 (21.69; 27.48) | 24.60 (21.53; 27.94) | 0.47 (0.30; 0.65) | 25.13 (22.32; 28.30) | 25.38 (22.29; 28.94) | 0.91 (0.73; 1.08) | 1.04 (0.92; 1.17) | 1.08 (0.94; 1.22) | 2.85 (2.67; 3.02) | 0.01 (0.01; 0.02) | 0.03 (0.03; 0.03) | 103.33 (103.02; 103.64) | 0.00 (0.00; 0.00) | 0.01 (0.01; 0.01) | 115.59 (115.26; 115.91) | 0.00 (0.00; 0.00) | 0.00 (0.00; 0.01) | 123.71 (122.89; 124.52) | 0.06 (0.06; 0.07) | 0.12 (0.10; 0.13) | 85.17 (84.93; 85.41) | 0.06 (0.06; 0.07) | 0.12 (0.11; 0.14) | 86.18 (85.94; 86.42) | 0.00 (0.00; 0.00) | 0.01 (0.01; 0.01) | 126.63 (126.32; 126.94) |
| **Colombia** | 0.01 (0.01; 0.01) | 0.01 (0.01; 0.01) | 11.43 (11.11; 11.74) | 0.00 (0.00; 0.00) | 0.00 (0.00; 0.00) | 0.86 (0.58; 1.15) | 0.81 (0.56; 1.13) | 0.91 (0.62; 1.28) | 12.76 (12.22; 13.31) | 25.14 (20.76; 30.02) | 22.73 (18.27; 27.70) | -8.98 (-9.24; -8.71) | 25.94 (21.33; 31.01) | 23.65 (19.30; 28.67) | -8.29 (-8.54; -8.04) | 1.09 (0.91; 1.30) | 1.03 (0.84; 1.25) | -5.78 (-6.03; -5.52) | 0.00 (0.00; 0.00) | 0.01 (0.01; 0.01) | 105.38 (104.88; 105.89) | 0.00 (0.00; 0.00) | 0.00 (0.00; 0.00) | 114.41 (113.90; 114.91) | 0.00 (0.00; 0.00) | 0.00 (0.00; 0.00) | 128.62 (127.68; 129.56) | 0.01 (0.01; 0.02) | 0.03 (0.02; 0.03) | 79.28 (78.88; 79.67) | 0.01 (0.01; 0.02) | 0.03 (0.02; 0.03) | 80.82 (80.43; 81.21) | 0.00 (0.00; 0.00) | 0.00 (0.00; 0.00) | 128.70 (128.23; 129.18) |
| **Costa Rica** | 0.01 (0.01; 0.01) | 0.01 (0.01; 0.01) | 5.31 (5.08; 5.55) | 0.00 (0.00; 0.00) | 0.00 (0.00; 0.00) | 0.39 (0.16; 0.61) | 0.87 (0.63; 1.17) | 0.93 (0.66; 1.25) | 6.15 (5.69; 6.60) | 25.59 (22.18; 29.19) | 24.09 (20.38; 28.88) | -5.67 (-5.89; -5.45) | 26.46 (22.97; 30.60) | 25.03 (21.24; 30.14) | -5.27 (-5.49; -5.06) | 1.10 (0.95; 1.24) | 1.06 (0.89; 1.27) | -3.54 (-3.76; -3.32) | 0.00 (0.00; 0.00) | 0.00 (0.00; 0.00) | 84.52 (84.08; 84.96) | 0.00 (0.00; 0.00) | 0.00 (0.00; 0.00) | 102.64 (102.19; 103.09) | 0.00 (0.00; 0.00) | 0.00 (0.00; 0.00) | 102.95 (102.21; 103.69) | 0.00 (0.00; 0.00) | 0.00 (0.00; 0.00) | 75.74 (75.32; 76.15) | 0.00 (0.00; 0.00) | 0.00 (0.00; 0.00) | 76.64 (76.22; 77.05) | 0.00 (0.00; 0.00) | 0.00 (0.00; 0.00) | 122.21 (121.71; 122.72) |
| **El Salvador** | 0.01 (0.01; 0.01) | 0.01 (0.01; 0.01) | 26.27 (25.88; 26.66) | 0.00 (0.00; 0.00) | 0.00 (0.00; 0.00) | 16.67 (16.30; 17.03) | 0.74 (0.51; 1.04) | 0.95 (0.65; 1.39) | 27.28 (26.66; 27.89) | 27.18 (21.34; 34.47) | 28.88 (22.30; 36.70) | 6.38 (6.03; 6.74) | 27.92 (22.43; 34.79) | 29.83 (23.22; 37.71) | 6.95 (6.60; 7.29) | 1.11 (0.88; 1.38) | 1.22 (0.93; 1.54) | 9.52 (9.17; 9.88) | 0.00 (0.00; 0.00) | 0.00 (0.00; 0.00) | 84.81 (84.16; 85.46) | 0.00 (0.00; 0.00) | 0.00 (0.00; 0.00) | 88.69 (88.05; 89.32) | 0.00 (0.00; 0.00) | 0.00 (0.00; 0.00) | 99.51 (98.58; 100.45) | 0.00 (0.00; 0.00) | 0.00 (0.00; 0.00) | 61.56 (61.01; 62.12) | 0.00 (0.00; 0.00) | 0.00 (0.00; 0.00) | 62.58 (62.03; 63.13) | 0.00 (0.00; 0.00) | 0.00 (0.00; 0.00) | 90.46 (89.85; 91.07) |
| **Guatemala** | 0.00 (0.00; 0.00) | 0.00 (0.00; 0.00) | 8.57 (8.30; 8.83) | 0.00 (0.00; 0.00) | 0.00 (0.00; 0.00) | -2.19 (-2.43; -1.95) | 0.46 (0.32; 0.61) | 0.50 (0.35; 0.69) | 9.84 (9.35; 10.34) | 21.57 (18.32; 25.17) | 19.12 (15.86; 22.82) | -10.65 (-10.87; -10.43) | 22.02 (18.89; 25.61) | 19.62 (16.26; 23.45) | -10.22 (-10.44; -10) | 0.92 (0.79; 1.06) | 0.85 (0.70; 1.01) | -7.68 (-7.90; -7.46) | 0.00 (0.00; 0.00) | 0.00 (0.00; 0.00) | 123.67 (123.17; 124.16) | 0.00 (0.00; 0.00) | 0.00 (0.00; 0.00) | 126.67 (126.18; 127.15) | 0.00 (0.00; 0.00) | 0.00 (0.00; 0.00) | 145.52 (144.60; 146.45) | 0.00 (0.00; 0.00) | 0.00 (0.00; 0.01) | 94.09 (93.71; 94.46) | 0.00 (0.00; 0.00) | 0.01 (0.00; 0.01) | 95.15 (94.77; 95.52) | 0.00 (0.00; 0.00) | 0.00 (0.00; 0.00) | 131.33 (130.91; 131.76) |
| **Honduras** | 0.00 (0.00; 0.01) | 0.00 (0.00; 0.01) | 12.12 (11.62; 12.62) | 0.00 (0.00; 0.00) | 0.00 (0.00; 0.00) | 5.40 (4.90; 5.89) | 0.58 (0.36; 0.88) | 0.67 (0.41; 1.05) | 13.90 (13.20; 14.61) | 29.13 (21.07; 39.91) | 28.04 (19.55; 39.43) | -3.68 (-4.15; -3.20) | 29.70 (21.98; 40.43) | 28.70 (20.32; 40.15) | -3.33 (-3.80; -2.86) | 1.25 (0.89; 1.74) | 1.27 (0.86; 1.80) | 0.86 (0.35; 1.36) | 0.00 (0.00; 0.00) | 0.00 (0.00; 0.00) | 150.97 (149.95; 151.98) | 0.00 (0.00; 0.00) | 0.00 (0.00; 0.00) | 148.99 (147.98; 150) | 0.00 (0.00; 0.00) | 0.00 (0.00; 0.00) | 165.41 (164.01; 166.80) | 0.00 (0.00; 0.00) | 0.00 (0.00; 0.01) | 121.14 (120.26; 122.01) | 0.00 (0.00; 0.00) | 0.00 (0.00; 0.01) | 121.98 (121.12; 122.85) | 0.00 (0.00; 0.00) | 0.00 (0.00; 0.00) | 144.30 (143.29; 145.32) |
| **Mexico** | 0.00 (0.00; 0.01) | 0.01 (0.01; 0.01) | 31.67 (31.42; 31.92) | 0.00 (0.00; 0.00) | 0.00 (0.00; 0.00) | 20.21 (19.99; 20.44) | 0.57 (0.40; 0.78) | 0.76 (0.53; 1.04) | 32.11 (31.56; 32.67) | 21.80 (19.29; 24.57) | 24.12 (20.44; 28.18) | 10.22 (10; 10.43) | 22.37 (19.80; 25.14) | 24.88 (21.02; 28.98) | 10.78 (10.57; 10.99) | 0.92 (0.82; 1.03) | 1.04 (0.88; 1.22) | 11.76 (11.54; 11.97) | 0.01 (0.01; 0.01) | 0.01 (0.01; 0.02) | 131.39 (130.94; 131.83) | 0.00 (0.00; 0.00) | 0.00 (0.00; 0.01) | 139.93 (139.47; 140.39) | 0.00 (0.00; 0.00) | 0.00 (0.00; 0.00) | 153.09 (152.19; 153.99) | 0.03 (0.03; 0.03) | 0.06 (0.05; 0.07) | 103.01 (102.67; 103.34) | 0.03 (0.03; 0.03) | 0.06 (0.05; 0.07) | 104.27 (103.94; 104.61) | 0.00 (0.00; 0.00) | 0.00 (0.00; 0.00) | 145.23 (144.82; 145.63) |
| **Nicaragua** | 0.00 (0.00; 0.00) | 0.00 (0.00; 0.01) | 33.44 (33.01; 33.87) | 0.00 (0.00; 0.00) | 0.00 (0.00; 0.00) | 21.71 (21.31; 22.12) | 0.35 (0.24; 0.49) | 0.50 (0.34; 0.71) | 37.68 (37.06; 38.31) | 13.80 (10.62; 17.24) | 14.91 (11.37; 18.90) | 7.93 (7.56; 8.30) | 14.16 (10.95; 17.59) | 15.40 (11.90; 19.50) | 8.69 (8.33; 9.05) | 0.58 (0.45; 0.72) | 0.67 (0.50; 0.84) | 13.76 (13.39; 14.14) | 0.00 (0.00; 0.00) | 0.00 (0.00; 0.00) | 194.93 (193.95; 195.91) | 0.00 (0.00; 0.00) | 0.00 (0.00; 0.00) | 205.42 (204.40; 206.44) | 0.00 (0.00; 0.00) | 0.00 (0.00; 0.00) | 230.97 (229.62; 232.33) | 0.00 (0.00; 0.00) | 0.00 (0.00; 0.00) | 151.65 (150.82; 152.47) | 0.00 (0.00; 0.00) | 0.00 (0.00; 0.00) | 153.64 (152.82; 154.45) | 0.00 (0.00; 0.00) | 0.00 (0.00; 0.00) | 217.12 (216.10; 218.13) |
| **Panama** | 0.01 (0.01; 0.01) | 0.01 (0.01; 0.01) | 19.57 (19.22; 19.93) | 0.00 (0.00; 0.00) | 0.00 (0.00; 0.00) | 11.99 (11.65; 12.32) | 0.85 (0.57; 1.21) | 1.03 (0.68; 1.45) | 19.92 (19.33; 20.50) | 27.69 (21.73; 33.54) | 28.88 (22.71; 36.10) | 4.46 (4.13; 4.78) | 28.54 (22.25; 34.35) | 29.91 (23.66; 37.35) | 4.92 (4.60; 5.24) | 1.23 (0.97; 1.48) | 1.31 (1.03; 1.64) | 6.46 (6.14; 6.78) | 0.00 (0.00; 0.00) | 0.00 (0.00; 0.00) | 137.54 (136.81; 138.28) | 0.00 (0.00; 0.00) | 0.00 (0.00; 0.00) | 150.06 (149.27; 150.84) | 0.00 (0.00; 0.00) | 0.00 (0.00; 0.00) | 155.56 (154.44; 156.67) | 0.00 (0.00; 0.00) | 0.00 (0.00; 0.00) | 117.89 (117.32; 118.46) | 0.00 (0.00; 0.00) | 0.00 (0.00; 0.00) | 119.02 (118.47; 119.57) | 0.00 (0.00; 0.00) | 0.00 (0.00; 0.00) | 163.05 (162.39; 163.71) |
| **Venezuela (Bolivarian Republic of)** | 0.01 (0.01; 0.01) | 0.01 (0.00; 0.01) | -15.61 (-16.02; -15.20) | 0.00 (0.00; 0.00) | 0.00 (0.00; 0.00) | -4.42 (-4.84; -4.01) | 0.90 (0.59; 1.33) | 0.74 (0.44; 1.12) | -14.90 (-15.46; -14.34) | 35.44 (26.80; 45.80) | 36.71 (25.91; 51.68) | 2.62 (2.18; 3.06) | 36.33 (27.69; 46.26) | 37.44 (26.40; 52.70) | 2.21 (1.77; 2.65) | 1.48 (1.13; 1.87) | 1.58 (1.14; 2.17) | 5.67 (5.25; 6.09) | 0.00 (0.00; 0.00) | 0.00 (0.00; 0.00) | 5.60 (5.09; 6.10) | 0.00 (0.00; 0.00) | 0.00 (0.00; 0.00) | 39.04 (38.37; 39.71) | 0.00 (0.00; 0.00) | 0.00 (0.00; 0.00) | 16.48 (15.79; 17.17) | 0.01 (0.01; 0.01) | 0.01 (0.01; 0.02) | 33.68 (33.19; 34.17) | 0.01 (0.01; 0.01) | 0.02 (0.01; 0.02) | 33.28 (32.79; 33.76) | 0.00 (0.00; 0.00) | 0.00 (0.00; 0.00) | 66.57 (65.87; 67.27) |
| **Tropical Latin America** | 0.01 (0.01; 0.01) | 0.01 (0.01; 0.02) | 11.30 (11.12; 11.49) | 0.00 (0.00; 0.00) | 0.00 (0.00; 0.00) | 3.02 (2.85; 3.20) | 1.37 (0.99; 1.79) | 1.54 (1.07; 2.07) | 12.28 (11.82; 12.75) | 58.50 (54.96; 61.99) | 55.60 (47.57; 65.00) | -4.50 (-4.66; -4.34) | 59.87 (56.35; 63.39) | 57.15 (48.91; 66.74) | -4.11 (-4.28; -3.95) | 2.21 (2.05; 2.35) | 2.16 (1.85; 2.55) | -2.58 (-2.75; -2.40) | 0.03 (0.03; 0.03) | 0.06 (0.05; 0.07) | 75.26 (74.89; 75.63) | 0.01 (0.01; 0.01) | 0.02 (0.01; 0.02) | 82.81 (82.41; 83.21) | 0.00 (0.00; 0.00) | 0.01 (0.00; 0.01) | 89.83 (89.11; 90.56) | 0.15 (0.15; 0.16) | 0.24 (0.20; 0.30) | 55.61 (55.26; 55.96) | 0.16 (0.15; 0.17) | 0.25 (0.21; 0.31) | 56.40 (56.04; 56.75) | 0.01 (0.01; 0.01) | 0.01 (0.01; 0.01) | 90.51 (90.10; 90.91) |
| **Brazil** | 0.01 (0.01; 0.01) | 0.01 (0.01; 0.02) | 11.36 (11.17; 11.55) | 0.00 (0.00; 0.00) | 0.00 (0.00; 0.00) | 2.95 (2.78; 3.13) | 1.38 (1.00; 1.80) | 1.55 (1.08; 2.09) | 12.31 (11.85; 12.78) | 58.85 (55.25; 62.36) | 55.87 (47.73; 65.11) | -4.60 (-4.77; -4.44) | 60.23 (56.77; 63.88) | 57.43 (49.12; 66.95) | -4.21 (-4.38; -4.05) | 2.22 (2.06; 2.36) | 2.16 (1.85; 2.55) | -2.70 (-2.87; -2.52) | 0.03 (0.03; 0.03) | 0.06 (0.05; 0.07) | 74.37 (74; 74.74) | 0.01 (0.01; 0.01) | 0.02 (0.01; 0.02) | 81.90 (81.50; 82.30) | 0.00 (0.00; 0.00) | 0.01 (0.00; 0.01) | 88.95 (88.23; 89.68) | 0.15 (0.14; 0.16) | 0.24 (0.20; 0.30) | 54.62 (54.26; 54.97) | 0.16 (0.15; 0.16) | 0.24 (0.20; 0.30) | 55.41 (55.05; 55.77) | 0.01 (0.01; 0.01) | 0.01 (0.01; 0.01) | 89.69 (89.28; 90.10) |
| **Paraguay** | 0.01 (0.01; 0.01) | 0.01 (0.01; 0.01) | 18.07 (17.54; 18.61) | 0.00 (0.00; 0.00) | 0.00 (0.00; 0.00) | 12.78 (12.29; 13.27) | 1.01 (0.65; 1.51) | 1.22 (0.75; 1.79) | 18.51 (17.85; 19.17) | 44.23 (31.35; 62.21) | 47.19 (32.99; 66.04) | 6.76 (6.27; 7.25) | 45.21 (32.14; 61.91) | 48.41 (34.16; 67.57) | 7.03 (6.54; 7.51) | 1.72 (1.24; 2.35) | 1.87 (1.34; 2.59) | 7.90 (7.43; 8.37) | 0.00 (0.00; 0.00) | 0.00 (0.00; 0.00) | 127.93 (126.97; 128.89) | 0.00 (0.00; 0.00) | 0.00 (0.00; 0.00) | 135.21 (134.23; 136.19) | 0.00 (0.00; 0.00) | 0.00 (0.00; 0.00) | 140.09 (138.83; 141.34) | 0.00 (0.00; 0.00) | 0.01 (0.00; 0.01) | 111.11 (110.24; 111.98) | 0.00 (0.00; 0.00) | 0.01 (0.00; 0.01) | 111.76 (110.91; 112.62) | 0.00 (0.00; 0.00) | 0.00 (0.00; 0.00) | 136.65 (135.77; 137.53) |
| **North Africa and Middle East** | 0.00 (0.00; 0.01) | 0.01 (0.00; 0.01) | 18.39 (18.16; 18.62) | 0.00 (0.00; 0.00) | 0.00 (0.00; 0.00) | 13.04 (12.83; 13.25) | 0.52 (0.37; 0.72) | 0.65 (0.46; 0.87) | 21.85 (21.34; 22.35) | 17.22 (15.13; 19.71) | 17.52 (15.06; 20.32) | 1.93 (1.73; 2.13) | 17.74 (15.64; 20.22) | 18.16 (15.67; 20.98) | 2.52 (2.33; 2.72) | 0.71 (0.63; 0.81) | 0.78 (0.67; 0.89) | 7.94 (7.75; 8.13) | 0.02 (0.02; 0.03) | 0.07 (0.06; 0.08) | 159.02 (158.63; 159.41) | 0.01 (0.01; 0.01) | 0.02 (0.02; 0.02) | 178.51 (178.13; 178.89) | 0.00 (0.00; 0.00) | 0.01 (0.01; 0.01) | 189.21 (188.23; 190.19) | 0.09 (0.08; 0.10) | 0.20 (0.18; 0.23) | 129.46 (129.12; 129.80) | 0.09 (0.08; 0.10) | 0.21 (0.19; 0.24) | 131.19 (130.87; 131.52) | 0.00 (0.00; 0.00) | 0.01 (0.01; 0.01) | 193.16 (192.79; 193.53) |
| **North Africa and Middle East** | 0.00 (0.00; 0.01) | 0.01 (0.00; 0.01) | 18.39 (18.16; 18.62) | 0.00 (0.00; 0.00) | 0.00 (0.00; 0.00) | 13.04 (12.83; 13.25) | 0.52 (0.37; 0.72) | 0.65 (0.46; 0.87) | 21.85 (21.34; 22.35) | 17.22 (15.13; 19.71) | 17.52 (15.06; 20.32) | 1.93 (1.73; 2.13) | 17.74 (15.64; 20.22) | 18.16 (15.67; 20.98) | 2.52 (2.33; 2.72) | 0.71 (0.63; 0.81) | 0.78 (0.67; 0.89) | 7.94 (7.75; 8.13) | 0.02 (0.02; 0.03) | 0.07 (0.06; 0.08) | 159.02 (158.63; 159.41) | 0.01 (0.01; 0.01) | 0.02 (0.02; 0.02) | 178.51 (178.13; 178.89) | 0.00 (0.00; 0.00) | 0.01 (0.01; 0.01) | 189.21 (188.23; 190.19) | 0.09 (0.08; 0.10) | 0.20 (0.18; 0.23) | 129.46 (129.12; 129.80) | 0.09 (0.08; 0.10) | 0.21 (0.19; 0.24) | 131.19 (130.87; 131.52) | 0.00 (0.00; 0.00) | 0.01 (0.01; 0.01) | 193.16 (192.79; 193.53) |
| **Afghanistan** | 0.00 (0.00; 0.00) | 0.00 (0.00; 0.00) | 15.27 (14.53; 16.01) | 0.00 (0.00; 0.00) | 0.00 (0.00; 0.00) | 3.72 (3.12; 4.32) | 0.36 (0.20; 0.58) | 0.43 (0.23; 0.70) | 17.54 (16.71; 18.37) | 20.92 (12.63; 32.23) | 19.76 (12.22; 29.74) | -5.78 (-6.40; -5.17) | 21.26 (13.06; 31.75) | 20.19 (12.66; 30.27) | -5.39 (-5.99; -4.78) | 0.82 (0.54; 1.17) | 0.82 (0.54; 1.15) | -1.14 (-1.67; -0.61) | 0.00 (0.00; 0.00) | 0.00 (0.00; 0.00) | 255 (252.81; 257.19) | 0.00 (0.00; 0.00) | 0.00 (0.00; 0.00) | 239.53 (237.57; 241.49) | 0.00 (0.00; 0.00) | 0.00 (0.00; 0.00) | 279.34 (276.92; 281.77) | 0.00 (0.00; 0.00) | 0.01 (0.00; 0.01) | 190.11 (188.28; 191.95) | 0.00 (0.00; 0.00) | 0.01 (0.00; 0.01) | 191.48 (189.66; 193.31) | 0.00 (0.00; 0.00) | 0.00 (0.00; 0.00) | 228.26 (226.38; 230.14) |
| **Algeria** | 0.00 (0.00; 0.01) | 0.01 (0.00; 0.01) | 21.33 (20.82; 21.84) | 0.00 (0.00; 0.00) | 0.00 (0.00; 0.00) | 11.64 (11.18; 12.11) | 0.54 (0.35; 0.78) | 0.66 (0.43; 1.01) | 22.42 (21.75; 23.08) | 18.72 (13.58; 25.13) | 19.01 (13.83; 25.37) | 2.45 (2.02; 2.88) | 19.25 (14.10; 25.45) | 19.68 (14.49; 26.04) | 3.02 (2.60; 3.44) | 0.81 (0.60; 1.06) | 0.85 (0.63; 1.12) | 5.23 (4.82; 5.64) | 0.00 (0.00; 0.00) | 0.00 (0.00; 0.01) | 144.91 (143.94; 145.88) | 0.00 (0.00; 0.00) | 0.00 (0.00; 0.00) | 165 (164; 166) | 0.00 (0.00; 0.00) | 0.00 (0.00; 0.00) | 175.59 (174.29; 176.89) | 0.01 (0.01; 0.01) | 0.02 (0.01; 0.02) | 117.64 (116.90; 118.37) | 0.01 (0.01; 0.01) | 0.02 (0.01; 0.02) | 119.26 (118.55; 119.98) | 0.00 (0.00; 0.00) | 0.00 (0.00; 0.00) | 183.58 (182.72; 184.44) |
| **Bahrain** | 0.01 (0.01; 0.01) | 0.01 (0.01; 0.01) | 13.13 (12.61; 13.65) | 0.00 (0.00; 0.00) | 0.00 (0.00; 0.00) | 6.05 (5.57; 6.53) | 1.06 (0.64; 1.56) | 1.26 (0.77; 1.88) | 18.36 (17.68; 19.04) | 29.01 (19.63; 38.52) | 26.72 (17.71; 36.44) | -7.25 (-7.70; -6.79) | 30.06 (20.48; 40.35) | 27.98 (19.03; 37.59) | -6.34 (-6.77; -5.90) | 1.36 (0.97; 1.77) | 1.41 (0.96; 1.93) | 2.95 (2.49; 3.41) | 0.00 (0.00; 0.00) | 0.00 (0.00; 0.00) | 287.35 (285.88; 288.82) | 0.00 (0.00; 0.00) | 0.00 (0.00; 0.00) | 340.77 (339.12; 342.41) | 0.00 (0.00; 0.00) | 0.00 (0.00; 0.00) | 358.11 (355.92; 360.31) | 0.00 (0.00; 0.00) | 0.00 (0.00; 0.00) | 228.08 (226.85; 229.31) | 0.00 (0.00; 0.00) | 0.00 (0.00; 0.00) | 232.46 (231.26; 233.67) | 0.00 (0.00; 0.00) | 0.00 (0.00; 0.00) | 400.96 (399.19; 402.74) |
| **Egypt** | 0.00 (0.00; 0.00) | 0.00 (0.00; 0.00) | 29.33 (28.95; 29.70) | 0.00 (0.00; 0.00) | 0.00 (0.00; 0.00) | 15.64 (15.32; 15.97) | 0.37 (0.26; 0.53) | 0.50 (0.35; 0.69) | 31.88 (31.31; 32.45) | 14.42 (11.60; 17.97) | 14.60 (11.70; 18.26) | 0.92 (0.61; 1.24) | 14.79 (11.96; 18.20) | 15.10 (12.12; 18.78) | 1.71 (1.40; 2.02) | 0.60 (0.50; 0.74) | 0.64 (0.52; 0.80) | 5.71 (5.40; 6.01) | 0.00 (0.00; 0.00) | 0.01 (0.00; 0.01) | 166.51 (165.78; 167.24) | 0.00 (0.00; 0.00) | 0.00 (0.00; 0.00) | 159.95 (159.24; 160.66) | 0.00 (0.00; 0.00) | 0.00 (0.00; 0.00) | 188.84 (187.71; 189.96) | 0.01 (0.01; 0.01) | 0.02 (0.02; 0.03) | 114.06 (113.52; 114.60) | 0.01 (0.01; 0.01) | 0.02 (0.02; 0.03) | 115.89 (115.35; 116.42) | 0.00 (0.00; 0.00) | 0.00 (0.00; 0.00) | 150.97 (150.37; 151.56) |
| **Iran (Islamic Republic of)** | 0.00 (0.00; 0.00) | 0.00 (0.00; 0.00) | 14.57 (14.34; 14.79) | 0.00 (0.00; 0.00) | 0.00 (0.00; 0.00) | 10.09 (9.88; 10.31) | 0.38 (0.26; 0.51) | 0.44 (0.30; 0.60) | 16.55 (16.05; 17.06) | 10.42 (9.25; 11.67) | 10.67 (9.09; 12.55) | 2.73 (2.53; 2.93) | 10.79 (9.64; 12.06) | 11.11 (9.49; 13.00) | 3.22 (3.02; 3.42) | 0.46 (0.41; 0.52) | 0.50 (0.42; 0.58) | 6.38 (6.17; 6.58) | 0.00 (0.00; 0.00) | 0.01 (0.01; 0.01) | 151.88 (151.40; 152.36) | 0.00 (0.00; 0.00) | 0.00 (0.00; 0.00) | 190.50 (189.94; 191.05) | 0.00 (0.00; 0.00) | 0.00 (0.00; 0.00) | 189.61 (188.57; 190.64) | 0.01 (0.01; 0.01) | 0.02 (0.02; 0.03) | 142.37 (141.89; 142.86) | 0.01 (0.01; 0.01) | 0.02 (0.02; 0.03) | 144.01 (143.53; 144.49) | 0.00 (0.00; 0.00) | 0.00 (0.00; 0.00) | 231.95 (231.27; 232.62) |
| **Iraq** | 0.01 (0.00; 0.01) | 0.01 (0.00; 0.01) | 12.37 (11.93; 12.81) | 0.00 (0.00; 0.00) | 0.00 (0.00; 0.00) | 3.68 (3.28; 4.08) | 0.67 (0.42; 1.00) | 0.77 (0.48; 1.13) | 14.67 (14.03; 15.32) | 23.03 (16.63; 29.66) | 21.61 (15.46; 28.78) | -5.89 (-6.30; -5.47) | 23.69 (17.47; 30.58) | 22.38 (16.13; 29.62) | -5.30 (-5.71; -4.90) | 0.91 (0.66; 1.16) | 0.91 (0.65; 1.19) | -0.65 (-1.06; -0.24) | 0.00 (0.00; 0.00) | 0.00 (0.00; 0.01) | 168.59 (167.42; 169.76) | 0.00 (0.00; 0.00) | 0.00 (0.00; 0.00) | 175.76 (174.55; 176.97) | 0.00 (0.00; 0.00) | 0.00 (0.00; 0.00) | 195.04 (193.46; 196.62) | 0.01 (0.00; 0.01) | 0.02 (0.01; 0.02) | 133.60 (132.54; 134.66) | 0.01 (0.00; 0.01) | 0.02 (0.01; 0.02) | 135.29 (134.24; 136.35) | 0.00 (0.00; 0.00) | 0.00 (0.00; 0.00) | 187.79 (186.56; 189.01) |
| **Jordan** | 0.01 (0.00; 0.01) | 0.01 (0.00; 0.01) | 3.90 (3.43; 4.37) | 0.00 (0.00; 0.00) | 0.00 (0.00; 0.00) | -1.08 (-1.51; -0.64) | 0.72 (0.47; 1.10) | 0.77 (0.49; 1.20) | 6.44 (5.78; 7.09) | 21.35 (15.27; 28.75) | 18.96 (12.80; 26.43) | -10.11 (-10.56; -9.66) | 22.09 (16.01; 29.91) | 19.73 (13.65; 27.18) | -9.57 (-10.01; -9.13) | 0.88 (0.65; 1.17) | 0.84 (0.58; 1.16) | -4.60 (-5.05; -4.15) | 0.00 (0.00; 0.00) | 0.00 (0.00; 0.00) | 214.65 (213.52; 215.78) | 0.00 (0.00; 0.00) | 0.00 (0.00; 0.00) | 249.28 (248.09; 250.47) | 0.00 (0.00; 0.00) | 0.00 (0.00; 0.00) | 255.90 (254.18; 257.63) | 0.00 (0.00; 0.00) | 0.01 (0.00; 0.01) | 186.15 (185.14; 187.17) | 0.00 (0.00; 0.00) | 0.01 (0.00; 0.01) | 188.37 (187.38; 189.36) | 0.00 (0.00; 0.00) | 0.00 (0.00; 0.00) | 291.80 (290.53; 293.08) |
| **Kuwait** | 0.01 (0.00; 0.01) | 0.01 (0.00; 0.01) | -0.23 (-0.50; 0.04) | 0.00 (0.00; 0.00) | 0.00 (0.00; 0.00) | -0.30 (-0.57; -0.03) | 0.58 (0.41; 0.80) | 0.59 (0.41; 0.86) | 2.16 (1.64; 2.68) | 11.92 (9.82; 14.39) | 11.16 (9.07; 13.64) | -6.44 (-6.71; -6.17) | 12.49 (10.33; 15.27) | 11.75 (9.60; 14.34) | -6.04 (-6.31; -5.78) | 0.54 (0.45; 0.65) | 0.55 (0.44; 0.67) | -0.39 (-0.67; -0.11) | 0.00 (0.00; 0.00) | 0.00 (0.00; 0.00) | 245.51 (244.64; 246.39) | 0.00 (0.00; 0.00) | 0.00 (0.00; 0.00) | 318.29 (317.25; 319.33) | 0.00 (0.00; 0.00) | 0.00 (0.00; 0.00) | 302.98 (301.40; 304.56) | 0.00 (0.00; 0.00) | 0.00 (0.00; 0.00) | 243.23 (242.39; 244.08) | 0.00 (0.00; 0.00) | 0.00 (0.00; 0.00) | 245.89 (245.08; 246.71) | 0.00 (0.00; 0.00) | 0.00 (0.00; 0.00) | 425.79 (424.49; 427.09) |
| **Lebanon** | 0.01 (0.01; 0.01) | 0.01 (0.01; 0.01) | 13.63 (13.22; 14.04) | 0.00 (0.00; 0.00) | 0.00 (0.00; 0.00) | 7.18 (6.80; 7.57) | 0.90 (0.61; 1.32) | 1.04 (0.70; 1.52) | 15.94 (15.34; 16.55) | 23.11 (17.40; 29.86) | 22.27 (16.49; 29.05) | -2.76 (-3.14; -2.37) | 24.01 (18.08; 31.15) | 23.31 (17.49; 29.98) | -2.05 (-2.42; -1.68) | 0.99 (0.76; 1.26) | 1.01 (0.76; 1.30) | 1.89 (1.52; 2.27) | 0.00 (0.00; 0.00) | 0.00 (0.00; 0.00) | 112.38 (111.66; 113.10) | 0.00 (0.00; 0.00) | 0.00 (0.00; 0.00) | 110.37 (109.67; 111.06) | 0.00 (0.00; 0.00) | 0.00 (0.00; 0.00) | 124.70 (123.69; 125.71) | 0.00 (0.00; 0.00) | 0.00 (0.00; 0.00) | 86.09 (85.44; 86.73) | 0.00 (0.00; 0.00) | 0.00 (0.00; 0.00) | 87.56 (86.93; 88.19) | 0.00 (0.00; 0.00) | 0.00 (0.00; 0.00) | 109.39 (108.73; 110.05) |
| **Libya** | 0.01 (0.01; 0.01) | 0.01 (0.01; 0.01) | 16.17 (15.69; 16.66) | 0.00 (0.00; 0.00) | 0.00 (0.00; 0.00) | 15.51 (15.04; 15.97) | 0.90 (0.58; 1.34) | 1.07 (0.69; 1.60) | 17.77 (17.12; 18.43) | 31.69 (22.57; 43.06) | 34.65 (25.39; 47.67) | 9.40 (8.93; 9.86) | 32.58 (23.93; 44.11) | 35.72 (26.16; 48.69) | 9.63 (9.18; 10.09) | 1.21 (0.88; 1.61) | 1.40 (1.03; 1.90) | 14.77 (14.31; 15.23) | 0.00 (0.00; 0.00) | 0.00 (0.00; 0.00) | 124.70 (123.82; 125.58) | 0.00 (0.00; 0.00) | 0.00 (0.00; 0.00) | 158.58 (157.59; 159.57) | 0.00 (0.00; 0.00) | 0.00 (0.00; 0.00) | 152.24 (151.03; 153.46) | 0.00 (0.00; 0.00) | 0.00 (0.00; 0.01) | 120.06 (119.27; 120.85) | 0.00 (0.00; 0.00) | 0.00 (0.00; 0.01) | 120.93 (120.16; 121.69) | 0.00 (0.00; 0.00) | 0.00 (0.00; 0.00) | 188.05 (187.11; 188.99) |
| **Morocco** | 0.00 (0.00; 0.01) | 0.00 (0.00; 0.01) | 21.46 (20.98; 21.94) | 0.00 (0.00; 0.00) | 0.00 (0.00; 0.00) | 6.77 (6.36; 7.18) | 0.45 (0.28; 0.66) | 0.56 (0.36; 0.82) | 23.32 (22.66; 23.97) | 18.93 (13.61; 24.31) | 17.51 (12.57; 22.82) | -6.87 (-7.25; -6.48) | 19.38 (14.09; 25.22) | 18.07 (13.09; 23.50) | -6.15 (-6.53; -5.78) | 0.74 (0.54; 0.92) | 0.72 (0.53; 0.92) | -2.30 (-2.66; -1.94) | 0.00 (0.00; 0.00) | 0.00 (0.00; 0.00) | 98.60 (97.90; 99.30) | 0.00 (0.00; 0.00) | 0.00 (0.00; 0.00) | 93.47 (92.79; 94.16) | 0.00 (0.00; 0.00) | 0.00 (0.00; 0.00) | 116.83 (115.82; 117.84) | 0.01 (0.01; 0.01) | 0.01 (0.01; 0.01) | 58.55 (58.02; 59.09) | 0.01 (0.01; 0.01) | 0.01 (0.01; 0.01) | 59.91 (59.38; 60.43) | 0.00 (0.00; 0.00) | 0.00 (0.00; 0.00) | 90.32 (89.75; 90.88) |
| **Oman** | 0.01 (0.01; 0.01) | 0.01 (0.00; 0.01) | -0.51 (-0.96; -0.06) | 0.00 (0.00; 0.00) | 0.00 (0.00; 0.00) | 1.15 (0.73; 1.58) | 0.78 (0.50; 1.15) | 0.81 (0.52; 1.18) | 4.55 (3.95; 5.14) | 21.99 (15.57; 29.51) | 20.11 (13.91; 27.49) | -7.18 (-7.62; -6.75) | 22.77 (16.55; 30.07) | 20.93 (14.57; 28.21) | -6.78 (-7.20; -6.36) | 0.87 (0.64; 1.14) | 0.88 (0.63; 1.18) | 1.71 (1.29; 2.12) | 0.00 (0.00; 0.00) | 0.00 (0.00; 0.00) | 286.22 (284.88; 287.56) | 0.00 (0.00; 0.00) | 0.00 (0.00; 0.00) | 349.79 (348.31; 351.26) | 0.00 (0.00; 0.00) | 0.00 (0.00; 0.00) | 347.48 (345.55; 349.42) | 0.00 (0.00; 0.00) | 0.00 (0.00; 0.00) | 278.74 (277.49; 280) | 0.00 (0.00; 0.00) | 0.00 (0.00; 0.00) | 281 (279.79; 282.22) | 0.00 (0.00; 0.00) | 0.00 (0.00; 0.00) | 412.09 (410.58; 413.59) |
| **Palestine** | 0.00 (0.00; 0.00) | 0.00 (0.00; 0.00) | 5.39 (5.04; 5.74) | 0.00 (0.00; 0.00) | 0.00 (0.00; 0.00) | 3.72 (3.38; 4.06) | 0.35 (0.24; 0.49) | 0.38 (0.25; 0.54) | 8.08 (7.53; 8.62) | 11.81 (9.40; 14.79) | 11.37 (8.76; 14.73) | -3.30 (-3.64; -2.97) | 12.16 (9.63; 15.23) | 11.76 (9.12; 15.16) | -2.97 (-3.30; -2.64) | 0.53 (0.43; 0.65) | 0.55 (0.43; 0.71) | 2.87 (2.54; 3.21) | 0.00 (0.00; 0.00) | 0.00 (0.00; 0.00) | 165.77 (165; 166.54) | 0.00 (0.00; 0.00) | 0.00 (0.00; 0.00) | 192.83 (192; 193.66) | 0.00 (0.00; 0.00) | 0.00 (0.00; 0.00) | 195.33 (194.13; 196.52) | 0.00 (0.00; 0.00) | 0.00 (0.00; 0.00) | 153.33 (152.65; 154.01) | 0.00 (0.00; 0.00) | 0.00 (0.00; 0.00) | 154.51 (153.85; 155.18) | 0.00 (0.00; 0.00) | 0.00 (0.00; 0.00) | 214.18 (213.41; 214.94) |
| **Qatar** | 0.01 (0.01; 0.01) | 0.01 (0.01; 0.01) | 2.35 (1.82; 2.89) | 0.00 (0.00; 0.00) | 0.00 (0.00; 0.00) | 1.69 (1.19; 2.19) | 0.94 (0.55; 1.42) | 1.00 (0.58; 1.56) | 6.19 (5.49; 6.89) | 20.41 (13.32; 28.21) | 18.84 (11.23; 26.71) | -7.06 (-7.60; -6.51) | 21.35 (13.85; 29.60) | 19.84 (12.08; 27.78) | -6.47 (-6.99; -5.95) | 1.00 (0.67; 1.35) | 1.04 (0.64; 1.43) | 1.99 (1.47; 2.52) | 0.00 (0.00; 0.00) | 0.00 (0.00; 0.00) | 606.23 (603.42; 609.04) | 0.00 (0.00; 0.00) | 0.00 (0.00; 0.00) | 757.96 (754.73; 761.20) | 0.00 (0.00; 0.00) | 0.00 (0.00; 0.00) | 737.56 (733.52; 741.61) | 0.00 (0.00; 0.00) | 0.00 (0.00; 0.00) | 567.18 (564.42; 569.95) | 0.00 (0.00; 0.00) | 0.00 (0.00; 0.00) | 574.28 (571.64; 576.92) | 0.00 (0.00; 0.00) | 0.00 (0.00; 0.00) | 979.40 (975.35; 983.46) |
| **Saudi Arabia** | 0.01 (0.01; 0.01) | 0.01 (0.01; 0.01) | 26.95 (26.49; 27.41) | 0.00 (0.00; 0.00) | 0.00 (0.00; 0.00) | 20.38 (19.97; 20.79) | 0.95 (0.64; 1.36) | 1.22 (0.80; 1.82) | 26.26 (25.61; 26.91) | 28.38 (21.88; 36.50) | 30.57 (22.95; 39.66) | 7.54 (7.15; 7.93) | 29.34 (22.90; 38.36) | 31.78 (24.24; 40.87) | 8.16 (7.78; 8.53) | 1.13 (0.88; 1.41) | 1.25 (0.95; 1.59) | 9.78 (9.41; 10.15) | 0.00 (0.00; 0.00) | 0.01 (0.01; 0.01) | 226.71 (225.64; 227.78) | 0.00 (0.00; 0.00) | 0.00 (0.00; 0.00) | 284.58 (283.40; 285.76) | 0.00 (0.00; 0.00) | 0.00 (0.00; 0.00) | 277.55 (275.95; 279.15) | 0.01 (0.01; 0.01) | 0.03 (0.02; 0.03) | 205.72 (204.82; 206.62) | 0.01 (0.01; 0.01) | 0.03 (0.02; 0.04) | 207.99 (207.11; 208.87) | 0.00 (0.00; 0.00) | 0.00 (0.00; 0.00) | 333.91 (332.79; 335.03) |
| **Sudan** | 0.00 (0.00; 0.00) | 0.00 (0.00; 0.00) | 7.48 (6.94; 8.01) | 0.00 (0.00; 0.00) | 0.00 (0.00; 0.00) | -0.98 (-1.45; -0.51) | 0.29 (0.19; 0.44) | 0.32 (0.20; 0.48) | 8.36 (7.74; 8.98) | 13.76 (9.65; 19.47) | 12.39 (8.66; 17.85) | -9.29 (-9.78; -8.80) | 14.05 (10.26; 19.74) | 12.71 (8.98; 18.23) | -8.92 (-9.39; -8.44) | 0.55 (0.41; 0.76) | 0.52 (0.38; 0.73) | -5.55 (-6.01; -5.10) | 0.00 (0.00; 0.00) | 0.00 (0.00; 0.00) | 181.42 (180.23; 182.61) | 0.00 (0.00; 0.00) | 0.00 (0.00; 0.00) | 188.75 (187.61; 189.89) | 0.00 (0.00; 0.00) | 0.00 (0.00; 0.00) | 206.80 (205.31; 208.29) | 0.00 (0.00; 0.00) | 0.01 (0.01; 0.01) | 148.16 (147.16; 149.17) | 0.00 (0.00; 0.00) | 0.01 (0.01; 0.01) | 149.35 (148.36; 150.33) | 0.00 (0.00; 0.00) | 0.00 (0.00; 0.00) | 192.07 (191.08; 193.06) |
| **Syrian Arab Republic** | 0.00 (0.00; 0.00) | 0.00 (0.00; 0.00) | 0.02 (-0.44; 0.49) | 0.00 (0.00; 0.00) | 0.00 (0.00; 0.00) | -2.67 (-3.09; -2.25) | 0.35 (0.22; 0.52) | 0.35 (0.22; 0.52) | 2.63 (2.03; 3.23) | 10.84 (7.93; 14.34) | 9.40 (6.73; 12.99) | -12.35 (-12.76; -11.93) | 11.17 (8.38; 14.84) | 9.75 (7.05; 13.40) | -11.88 (-12.28; -11.47) | 0.47 (0.36; 0.61) | 0.45 (0.33; 0.60) | -4.90 (-5.30; -4.51) | 0.00 (0.00; 0.00) | 0.00 (0.00; 0.00) | 61.06 (60.37; 61.75) | 0.00 (0.00; 0.00) | 0.00 (0.00; 0.00) | 77.09 (76.36; 77.83) | 0.00 (0.00; 0.00) | 0.00 (0.00; 0.00) | 79.80 (78.83; 80.76) | 0.00 (0.00; 0.00) | 0.00 (0.00; 0.00) | 42.53 (41.96; 43.09) | 0.00 (0.00; 0.00) | 0.00 (0.00; 0.00) | 43.66 (43.11; 44.21) | 0.00 (0.00; 0.00) | 0.00 (0.00; 0.00) | 88.32 (87.68; 88.96) |
| **Tunisia** | 0.01 (0.01; 0.01) | 0.01 (0.01; 0.02) | 21.29 (20.74; 21.84) | 0.00 (0.00; 0.00) | 0.00 (0.00; 0.00) | 10.44 (9.94; 10.94) | 1.07 (0.67; 1.62) | 1.31 (0.83; 1.97) | 21.16 (20.48; 21.85) | 32.15 (22.33; 45.11) | 32.16 (22.11; 45.13) | 0.16 (-0.35; 0.66) | 33.21 (23.58; 45.88) | 33.47 (23.44; 46.63) | 0.84 (0.35; 1.33) | 1.31 (0.92; 1.80) | 1.34 (0.93; 1.87) | 1.58 (1.09; 2.08) | 0.00 (0.00; 0.00) | 0.00 (0.00; 0.00) | 91.07 (90.32; 91.82) | 0.00 (0.00; 0.00) | 0.00 (0.00; 0.00) | 100.18 (99.42; 100.94) | 0.00 (0.00; 0.00) | 0.00 (0.00; 0.00) | 109.15 (108.17; 110.13) | 0.00 (0.00; 0.01) | 0.01 (0.01; 0.01) | 64.42 (63.80; 65.04) | 0.00 (0.00; 0.01) | 0.01 (0.01; 0.01) | 65.86 (65.26; 66.46) | 0.00 (0.00; 0.00) | 0.00 (0.00; 0.00) | 108.48 (107.82; 109.14) |
| **Türkiye** | 0.01 (0.00; 0.01) | 0.01 (0.00; 0.01) | 18.96 (18.56; 19.35) | 0.00 (0.00; 0.00) | 0.00 (0.00; 0.00) | 8.90 (8.55; 9.25) | 0.59 (0.40; 0.83) | 0.73 (0.49; 1.02) | 22.30 (21.73; 22.88) | 16.50 (12.78; 20.63) | 15.51 (11.87; 19.55) | -5.35 (-5.68; -5.02) | 17.09 (13.12; 21.33) | 16.23 (12.60; 20.28) | -4.39 (-4.70; -4.07) | 0.71 (0.56; 0.88) | 0.73 (0.57; 0.92) | 2.43 (2.10; 2.75) | 0.01 (0.00; 0.01) | 0.01 (0.01; 0.01) | 102.50 (101.90; 103.10) | 0.00 (0.00; 0.00) | 0.00 (0.00; 0.00) | 111.12 (110.50; 111.73) | 0.00 (0.00; 0.00) | 0.00 (0.00; 0.00) | 127.92 (127; 128.84) | 0.02 (0.01; 0.02) | 0.03 (0.02; 0.03) | 67.70 (67.26; 68.15) | 0.02 (0.01; 0.02) | 0.03 (0.02; 0.03) | 69.80 (69.36; 70.23) | 0.00 (0.00; 0.00) | 0.00 (0.00; 0.00) | 122.68 (122.17; 123.18) |
| **United Arab Emirates** | 0.01 (0.01; 0.01) | 0.01 (0.01; 0.01) | -8.16 (-8.52; -7.81) | 0.00 (0.00; 0.00) | 0.00 (0.00; 0.00) | -10.24 (-10.60; -9.89) | 1.03 (0.69; 1.51) | 0.94 (0.60; 1.39) | -8.28 (-8.83; -7.73) | 36.60 (27.96; 46.44) | 31.94 (24.18; 41.25) | -11.71 (-12.06; -11.37) | 37.62 (28.81; 47.41) | 32.87 (25.03; 42.24) | -11.62 (-11.95; -11.29) | 1.76 (1.35; 2.22) | 1.56 (1.17; 2.02) | -11.82 (-12.15; -11.48) | 0.00 (0.00; 0.00) | 0.00 (0.00; 0.00) | 437.17 (435.42; 438.92) | 0.00 (0.00; 0.00) | 0.00 (0.00; 0.00) | 645.73 (643.27; 648.19) | 0.00 (0.00; 0.00) | 0.00 (0.00; 0.00) | 547.50 (544.47; 550.54) | 0.00 (0.00; 0.00) | 0.01 (0.01; 0.02) | 442.66 (441.01; 444.31) | 0.00 (0.00; 0.00) | 0.01 (0.01; 0.02) | 445.30 (443.67; 446.93) | 0.00 (0.00; 0.00) | 0.00 (0.00; 0.00) | 843.12 (840.36; 845.89) |
| **Yemen** | 0.00 (0.00; 0.00) | 0.00 (0.00; 0.00) | 0.96 (0.47; 1.45) | 0.00 (0.00; 0.00) | 0.00 (0.00; 0.00) | -7.06 (-7.53; -6.59) | 0.30 (0.19; 0.47) | 0.31 (0.19; 0.49) | 2.59 (1.89; 3.29) | 15.36 (10.56; 21.40) | 13.00 (8.42; 18.47) | -15.23 (-15.73; -14.74) | 15.65 (10.86; 21.57) | 13.31 (8.75; 18.85) | -14.89 (-15.38; -14.41) | 0.63 (0.43; 0.86) | 0.56 (0.36; 0.79) | -11.34 (-11.83; -10.85) | 0.00 (0.00; 0.00) | 0.00 (0.00; 0.00) | 183.62 (182.47; 184.76) | 0.00 (0.00; 0.00) | 0.00 (0.00; 0.00) | 182.60 (181.50; 183.71) | 0.00 (0.00; 0.00) | 0.00 (0.00; 0.00) | 205.16 (203.57; 206.75) | 0.00 (0.00; 0.00) | 0.01 (0.00; 0.01) | 144.48 (143.41; 145.54) | 0.00 (0.00; 0.00) | 0.01 (0.00; 0.01) | 145.58 (144.53; 146.63) | 0.00 (0.00; 0.00) | 0.00 (0.00; 0.00) | 178.29 (177.21; 179.38) |
| **South Asia** | 0.03 (0.02; 0.03) | 0.03 (0.03; 0.04) | 13.39 (13.17; 13.60) | 0.01 (0.01; 0.01) | 0.01 (0.01; 0.01) | 4.96 (4.76; 5.16) | 3.21 (2.27; 4.22) | 3.69 (2.58; 4.90) | 14.33 (13.86; 14.80) | 179.07 (155.29; 201.21) | 178.39 (151.01; 207.74) | -0.33 (-0.53; -0.13) | 182.29 (157.06; 203.68) | 182.09 (154.19; 211.33) | -0.07 (-0.27; 0.13) | 6.52 (5.71; 7.27) | 6.58 (5.59; 7.53) | 0.49 (0.30; 0.68) | 0.45 (0.38; 0.50) | 0.95 (0.79; 1.12) | 107.26 (106.86; 107.65) | 0.15 (0.13; 0.17) | 0.33 (0.28; 0.39) | 113.22 (112.82; 113.63) | 0.05 (0.04; 0.07) | 0.12 (0.08; 0.16) | 122.28 (121.48; 123.08) | 2.91 (2.51; 3.28) | 5.61 (4.76; 6.57) | 88.72 (88.38; 89.07) | 2.96 (2.54; 3.33) | 5.73 (4.86; 6.70) | 89.31 (88.97; 89.65) | 0.10 (0.08; 0.11) | 0.22 (0.18; 0.25) | 119.41 (119.05; 119.78) |
| **South Asia** | 0.03 (0.02; 0.03) | 0.03 (0.03; 0.04) | 13.39 (13.17; 13.60) | 0.01 (0.01; 0.01) | 0.01 (0.01; 0.01) | 4.96 (4.76; 5.16) | 3.21 (2.27; 4.22) | 3.69 (2.58; 4.90) | 14.33 (13.86; 14.80) | 179.07 (155.29; 201.21) | 178.39 (151.01; 207.74) | -0.33 (-0.53; -0.13) | 182.29 (157.06; 203.68) | 182.09 (154.19; 211.33) | -0.07 (-0.27; 0.13) | 6.52 (5.71; 7.27) | 6.58 (5.59; 7.53) | 0.49 (0.30; 0.68) | 0.45 (0.38; 0.50) | 0.95 (0.79; 1.12) | 107.26 (106.86; 107.65) | 0.15 (0.13; 0.17) | 0.33 (0.28; 0.39) | 113.22 (112.82; 113.63) | 0.05 (0.04; 0.07) | 0.12 (0.08; 0.16) | 122.28 (121.48; 123.08) | 2.91 (2.51; 3.28) | 5.61 (4.76; 6.57) | 88.72 (88.38; 89.07) | 2.96 (2.54; 3.33) | 5.73 (4.86; 6.70) | 89.31 (88.97; 89.65) | 0.10 (0.08; 0.11) | 0.22 (0.18; 0.25) | 119.41 (119.05; 119.78) |
| **Bangladesh** | 0.02 (0.01; 0.03) | 0.02 (0.01; 0.03) | -2.60 (-3.18; -2.03) | 0.01 (0.00; 0.01) | 0.01 (0.00; 0.01) | -15.09 (-15.62; -14.57) | 2.47 (1.44; 3.89) | 2.43 (1.34; 3.94) | -1.82 (-2.55; -1.10) | 124.91 (78.95; 175.61) | 95.76 (56.69; 140.10) | -22.72 (-23.24; -22.20) | 127.46 (80.66; 179.96) | 98.19 (58.65; 142.45) | -22.31 (-22.82; -21.80) | 4.75 (3.10; 6.59) | 3.72 (2.30; 5.29) | -21.63 (-22.12; -21.14) | 0.03 (0.02; 0.04) | 0.06 (0.03; 0.09) | 78.89 (77.95; 79.82) | 0.01 (0.01; 0.01) | 0.02 (0.01; 0.03) | 74.89 (74; 75.79) | 0.00 (0.00; 0.01) | 0.01 (0.00; 0.01) | 92.98 (91.80; 94.17) | 0.18 (0.12; 0.26) | 0.28 (0.17; 0.41) | 48.10 (47.32; 48.89) | 0.19 (0.12; 0.27) | 0.28 (0.17; 0.42) | 48.98 (48.20; 49.75) | 0.01 (0.00; 0.01) | 0.01 (0.01; 0.02) | 74.66 (73.84; 75.48) |
| **Bhutan** | 0.02 (0.01; 0.03) | 0.02 (0.02; 0.03) | 23.89 (23.31; 24.46) | 0.01 (0.00; 0.01) | 0.01 (0.00; 0.01) | 4.29 (3.82; 4.76) | 2.30 (1.41; 3.39) | 2.91 (1.72; 4.19) | 25.54 (24.85; 26.22) | 119.83 (83.17; 167.71) | 108.10 (75.45; 151.65) | -10.74 (-11.21; -10.28) | 122.12 (83.91; 173.03) | 111.01 (78.26; 154.90) | -10.06 (-10.52; -9.60) | 4.58 (3.36; 6.06) | 4.38 (3.11; 5.84) | -5.95 (-6.37; -5.54) | 0.00 (0.00; 0.00) | 0.00 (0.00; 0.00) | 152.86 (151.91; 153.81) | 0.00 (0.00; 0.00) | 0.00 (0.00; 0.00) | 135.22 (134.42; 136.03) | 0.00 (0.00; 0.00) | 0.00 (0.00; 0.00) | 173.56 (172.31; 174.81) | 0.00 (0.00; 0.00) | 0.00 (0.00; 0.00) | 90.75 (90.02; 91.49) | 0.00 (0.00; 0.00) | 0.00 (0.00; 0.00) | 92.29 (91.56; 93.03) | 0.00 (0.00; 0.00) | 0.00 (0.00; 0.00) | 124.42 (123.71; 125.13) |
| **India** | 0.03 (0.02; 0.03) | 0.03 (0.02; 0.03) | 14.64 (14.41; 14.86) | 0.01 (0.01; 0.01) | 0.01 (0.01; 0.01) | 5.58 (5.37; 5.79) | 2.99 (2.07; 3.94) | 3.48 (2.41; 4.65) | 15.48 (15; 15.97) | 160.61 (137.67; 181.05) | 163.99 (135.66; 194.44) | 2.09 (1.86; 2.31) | 163.61 (139.58; 184.48) | 167.48 (139.10; 198.33) | 2.34 (2.11; 2.56) | 5.92 (5.16; 6.65) | 6.11 (5.08; 7.07) | 2.66 (2.45; 2.86) | 0.34 (0.29; 0.38) | 0.72 (0.58; 0.87) | 107.78 (107.32; 108.24) | 0.11 (0.10; 0.13) | 0.24 (0.20; 0.30) | 112.43 (111.96; 112.91) | 0.04 (0.03; 0.05) | 0.09 (0.06; 0.12) | 122.12 (121.25; 122.99) | 2.08 (1.78; 2.35) | 4.07 (3.32; 4.90) | 91.69 (91.28; 92.11) | 2.12 (1.79; 2.40) | 4.16 (3.39; 5.00) | 92.25 (91.84; 92.66) | 0.07 (0.06; 0.08) | 0.16 (0.13; 0.19) | 121.70 (121.25; 122.15) |
| **Nepal** | 0.02 (0.01; 0.03) | 0.02 (0.01; 0.03) | 13.41 (12.90; 13.91) | 0.01 (0.01; 0.01) | 0.01 (0.00; 0.01) | -4.17 (-4.60; -3.75) | 2.28 (1.45; 3.43) | 2.56 (1.62; 3.95) | 11.21 (10.54; 11.87) | 126.86 (92.10; 169.80) | 113.03 (79.93; 154.72) | -11.69 (-12.11; -11.27) | 129.18 (94.08; 173.87) | 115.59 (82.47; 157.84) | -11.28 (-11.70; -10.87) | 4.82 (3.61; 6.33) | 4.21 (3.07; 5.71) | -13.71 (-14.10; -13.32) | 0.00 (0.00; 0.01) | 0.01 (0.01; 0.01) | 106.52 (105.68; 107.35) | 0.00 (0.00; 0.00) | 0.00 (0.00; 0.00) | 95.40 (94.63; 96.16) | 0.00 (0.00; 0.00) | 0.00 (0.00; 0.00) | 116.37 (115.26; 117.48) | 0.03 (0.02; 0.04) | 0.06 (0.04; 0.08) | 68.39 (67.74; 69.04) | 0.03 (0.02; 0.04) | 0.06 (0.04; 0.08) | 69.23 (68.59; 69.87) | 0.00 (0.00; 0.00) | 0.00 (0.00; 0.00) | 88.44 (87.83; 89.05) |
| **Pakistan** | 0.05 (0.04; 0.06) | 0.05 (0.04; 0.06) | 3.49 (3.12; 3.87) | 0.02 (0.02; 0.03) | 0.02 (0.02; 0.03) | -3.03 (-3.38; -2.67) | 6.03 (4.04; 8.36) | 6.28 (4.18; 8.85) | 3.81 (3.28; 4.34) | 404.21 (314.72; 516.91) | 351.05 (267.59; 459.43) | -12.67 (-13.01; -12.33) | 410.22 (321.29; 518.79) | 357.34 (273.55; 466.37) | -12.43 (-12.77; -12.09) | 14.58 (11.41; 18.63) | 13.10 (10.12; 17.09) | -10.33 (-10.67; -9.99) | 0.07 (0.06; 0.10) | 0.17 (0.13; 0.22) | 116.78 (116.13; 117.43) | 0.03 (0.02; 0.04) | 0.07 (0.05; 0.09) | 131.27 (130.61; 131.92) | 0.01 (0.01; 0.01) | 0.02 (0.01; 0.03) | 135.01 (134; 136.03) | 0.62 (0.48; 0.79) | 1.21 (0.92; 1.53) | 91.79 (91.24; 92.34) | 0.63 (0.49; 0.80) | 1.23 (0.94; 1.55) | 92.42 (91.87; 92.96) | 0.02 (0.01; 0.02) | 0.04 (0.03; 0.05) | 128.03 (127.44; 128.62) |
| **Southeast Asia, East Asia, and Oceania** | 0.01 (0.01; 0.01) | 0.02 (0.01; 0.02) | 32.39 (32.12; 32.65) | 0.00 (0.00; 0.00) | 0.00 (0.00; 0.00) | 21.77 (21.52; 22.01) | 1.26 (0.90; 1.67) | 1.73 (1.23; 2.31) | 35.10 (34.58; 35.62) | 37.65 (32.31; 43.31) | 43.79 (37.09; 51.38) | 16.59 (16.35; 16.83) | 38.90 (33.48; 44.32) | 45.52 (38.54; 53.09) | 17.20 (16.96; 17.44) | 1.51 (1.30; 1.73) | 1.79 (1.52; 2.08) | 17.91 (17.68; 18.15) | 0.35 (0.30; 0.40) | 0.66 (0.56; 0.78) | 83.01 (82.66; 83.36) | 0.09 (0.08; 0.10) | 0.18 (0.16; 0.22) | 95.33 (94.98; 95.69) | 0.04 (0.03; 0.05) | 0.08 (0.05; 0.11) | 104.52 (103.78; 105.25) | 1.09 (0.93; 1.26) | 1.83 (1.59; 2.08) | 66.06 (65.79; 66.32) | 1.12 (0.97; 1.28) | 1.91 (1.66; 2.16) | 67.33 (67.07; 67.58) | 0.04 (0.04; 0.05) | 0.09 (0.08; 0.10) | 112.96 (112.65; 113.27) |
| **East Asia** | 0.01 (0.01; 0.01) | 0.02 (0.01; 0.02) | 39.25 (38.88; 39.61) | 0.00 (0.00; 0.00) | 0.00 (0.00; 0.00) | 24.18 (23.84; 24.52) | 1.18 (0.81; 1.59) | 1.69 (1.15; 2.32) | 41.02 (40.43; 41.61) | 30.72 (24.59; 37.72) | 33.39 (26.41; 42.00) | 9.50 (9.17; 9.83) | 31.88 (25.83; 38.82) | 35.07 (27.99; 43.47) | 10.69 (10.37; 11.01) | 1.23 (0.99; 1.51) | 1.38 (1.10; 1.69) | 11.84 (11.52; 12.15) | 0.25 (0.21; 0.30) | 0.45 (0.36; 0.57) | 75.40 (74.94; 75.85) | 0.06 (0.05; 0.08) | 0.12 (0.10; 0.15) | 84.18 (83.69; 84.67) | 0.03 (0.02; 0.03) | 0.05 (0.04; 0.07) | 96.89 (96.10; 97.69) | 0.67 (0.54; 0.83) | 0.98 (0.79; 1.19) | 44.70 (44.33; 45.07) | 0.70 (0.56; 0.85) | 1.03 (0.85; 1.25) | 46.68 (46.32; 47.04) | 0.03 (0.02; 0.03) | 0.05 (0.04; 0.06) | 91.36 (90.96; 91.76) |
| **China** | 0.01 (0.01; 0.01) | 0.02 (0.01; 0.02) | 45.87 (45.44; 46.29) | 0.00 (0.00; 0.00) | 0.00 (0.00; 0.00) | 27.46 (27.08; 27.84) | 1.07 (0.73; 1.46) | 1.60 (1.07; 2.23) | 46.77 (46.14; 47.40) | 28.15 (21.62; 35.38) | 31.09 (23.92; 40.01) | 11.39 (11.02; 11.77) | 29.20 (23.18; 36.49) | 32.69 (25.39; 41.34) | 12.72 (12.36; 13.08) | 1.15 (0.90; 1.43) | 1.30 (1.02; 1.62) | 13.64 (13.30; 13.99) | 0.22 (0.17; 0.26) | 0.42 (0.32; 0.53) | 86.93 (86.41; 87.46) | 0.06 (0.05; 0.07) | 0.11 (0.09; 0.14) | 92.97 (92.43; 93.52) | 0.02 (0.02; 0.03) | 0.05 (0.03; 0.07) | 108.68 (107.82; 109.55) | 0.60 (0.46; 0.75) | 0.90 (0.71; 1.11) | 49.98 (49.56; 50.40) | 0.62 (0.49; 0.78) | 0.95 (0.76; 1.16) | 52.19 (51.79; 52.59) | 0.02 (0.02; 0.03) | 0.05 (0.04; 0.06) | 98.11 (97.66; 98.55) |
| **Democratic People's Republic of Korea** | 0.01 (0.00; 0.01) | 0.01 (0.01; 0.01) | 9.70 (9.19; 10.22) | 0.00 (0.00; 0.00) | 0.00 (0.00; 0.00) | 11.38 (10.89; 11.88) | 0.76 (0.48; 1.15) | 0.86 (0.54; 1.29) | 12.71 (12.06; 13.37) | 30.38 (21.17; 42.58) | 32.92 (23.06; 46.23) | 7.40 (6.89; 7.90) | 31.13 (21.66; 42.91) | 33.77 (23.90; 47.24) | 7.52 (7.03; 8.02) | 1.10 (0.78; 1.48) | 1.26 (0.90; 1.70) | 13.02 (12.54; 13.49) | 0.00 (0.00; 0.00) | 0.00 (0.00; 0.00) | 39.59 (38.98; 40.21) | 0.00 (0.00; 0.00) | 0.00 (0.00; 0.00) | 59.80 (59.13; 60.47) | 0.00 (0.00; 0.00) | 0.00 (0.00; 0.00) | 55.72 (54.87; 56.56) | 0.01 (0.01; 0.01) | 0.02 (0.01; 0.02) | 43.32 (42.68; 43.96) | 0.01 (0.01; 0.01) | 0.02 (0.01; 0.02) | 43.62 (42.99; 44.24) | 0.00 (0.00; 0.00) | 0.00 (0.00; 0.00) | 77.02 (76.33; 77.71) |
| **Taiwan (Province of China)** | 0.09 (0.08; 0.10) | 0.09 (0.08; 0.10) | 2.52 (2.34; 2.69) | 0.02 (0.01; 0.02) | 0.02 (0.01; 0.02) | 1.46 (1.30; 1.63) | 7.46 (5.22; 10.13) | 7.66 (5.38; 10.54) | 2.72 (2.24; 3.20) | 170.65 (151.66; 189.86) | 164.07 (139.06; 198.18) | -4.02 (-4.23; -3.82) | 178.11 (160.54; 197.74) | 171.74 (145.71; 205.60) | -3.74 (-3.94; -3.54) | 5.57 (4.96; 6.18) | 5.41 (4.59; 6.46) | -3.37 (-3.57; -3.17) | 0.03 (0.03; 0.04) | 0.03 (0.03; 0.04) | -0.02 (-0.24; 0.19) | 0.01 (0.01; 0.01) | 0.01 (0.01; 0.01) | 9.11 (8.88; 9.33) | 0.00 (0.00; 0.00) | 0.00 (0.00; 0.00) | 5.93 (5.42; 6.43) | 0.07 (0.06; 0.07) | 0.07 (0.05; 0.08) | -2.48 (-2.71; -2.24) | 0.07 (0.06; 0.08) | 0.07 (0.06; 0.08) | -2.12 (-2.36; -1.89) | 0.00 (0.00; 0.00) | 0.00 (0.00; 0.00) | 22.75 (22.49; 23.02) |
| **Oceania** | 0.01 (0.00; 0.01) | 0.01 (0.00; 0.01) | -9.61 (-9.99; -9.22) | 0.00 (0.00; 0.00) | 0.00 (0.00; 0.00) | -8.55 (-8.94; -8.16) | 0.75 (0.52; 1.11) | 0.71 (0.46; 1.02) | -5.98 (-6.52; -5.44) | 42.51 (31.13; 54.44) | 37.61 (25.12; 50.55) | -11.16 (-11.56; -10.75) | 43.26 (31.42; 55.93) | 38.32 (25.89; 51.34) | -11.06 (-11.46; -10.67) | 1.58 (1.18; 2.00) | 1.47 (1.00; 1.95) | -7.50 (-7.90; -7.11) | 0.00 (0.00; 0.00) | 0.00 (0.00; 0.00) | 107.84 (107.11; 108.57) | 0.00 (0.00; 0.00) | 0.00 (0.00; 0.00) | 127.74 (126.97; 128.51) | 0.00 (0.00; 0.00) | 0.00 (0.00; 0.00) | 128.12 (127.10; 129.13) | 0.00 (0.00; 0.01) | 0.01 (0.01; 0.01) | 108.48 (107.76; 109.20) | 0.00 (0.00; 0.01) | 0.01 (0.01; 0.01) | 108.81 (108.10; 109.51) | 0.00 (0.00; 0.00) | 0.00 (0.00; 0.00) | 142.15 (141.41; 142.90) |
| **American Samoa** | 0.00 (0.00; 0.00) | 0.00 (0.00; 0.01) | 14.81 (14.40; 15.21) | 0.00 (0.00; 0.00) | 0.00 (0.00; 0.00) | 14.03 (13.66; 14.40) | 0.43 (0.29; 0.61) | 0.50 (0.34; 0.72) | 15.74 (15.16; 16.32) | 17.16 (13.25; 21.55) | 18.98 (14.60; 23.87) | 9.22 (8.86; 9.59) | 17.58 (13.72; 21.89) | 19.48 (15.06; 24.36) | 9.38 (9.03; 9.73) | 0.68 (0.54; 0.84) | 0.78 (0.62; 0.97) | 12.24 (11.91; 12.58) | 0.00 (0.00; 0.00) | 0.00 (0.00; 0.00) | 65.72 (65.17; 66.27) | 0.00 (0.00; 0.00) | 0.00 (0.00; 0.00) | 85.95 (85.38; 86.53) | 0.00 (0.00; 0.00) | 0.00 (0.00; 0.00) | 80.47 (79.65; 81.28) | 0.00 (0.00; 0.00) | 0.00 (0.00; 0.00) | 64.82 (64.40; 65.24) | 0.00 (0.00; 0.00) | 0.00 (0.00; 0.00) | 65.19 (64.78; 65.60) | 0.00 (0.00; 0.00) | 0.00 (0.00; 0.00) | 101.84 (101.37; 102.31) |
| **Cook Islands** | 0.01 (0.01; 0.01) | 0.01 (0.01; 0.02) | 23.93 (23.45; 24.40) | 0.00 (0.00; 0.00) | 0.00 (0.00; 0.00) | 20.18 (19.74; 20.61) | 1.06 (0.70; 1.58) | 1.33 (0.86; 1.99) | 24.09 (23.42; 24.77) | 32.54 (24.54; 42.01) | 38.11 (28.40; 50.01) | 16.56 (16.13; 16.98) | 33.60 (25.60; 43.90) | 39.44 (29.39; 51.43) | 16.80 (16.38; 17.21) | 1.25 (0.95; 1.59) | 1.47 (1.10; 1.89) | 16.82 (16.41; 17.22) | 0.00 (0.00; 0.00) | 0.00 (0.00; 0.00) | 46.06 (45.52; 46.60) | 0.00 (0.00; 0.00) | 0.00 (0.00; 0.00) | 57.57 (57.02; 58.11) | 0.00 (0.00; 0.00) | 0.00 (0.00; 0.00) | 55.49 (54.70; 56.29) | 0.00 (0.00; 0.00) | 0.00 (0.00; 0.00) | 43.17 (42.68; 43.67) | 0.00 (0.00; 0.00) | 0.00 (0.00; 0.00) | 43.56 (43.08; 44.04) | 0.00 (0.00; 0.00) | 0.00 (0.00; 0.00) | 69.41 (68.89; 69.93) |
| **Fiji** | 0.01 (0.01; 0.01) | 0.01 (0.01; 0.01) | 6.07 (5.63; 6.50) | 0.00 (0.00; 0.00) | 0.00 (0.00; 0.00) | 7.45 (7.02; 7.88) | 1.02 (0.69; 1.52) | 1.11 (0.73; 1.64) | 7.99 (7.39; 8.59) | 51.27 (37.53; 67.20) | 53.13 (39.11; 69.81) | 3.33 (2.93; 3.74) | 52.29 (38.55; 67.49) | 54.24 (40.30; 70.97) | 3.43 (3.03; 3.82) | 2.00 (1.50; 2.58) | 2.19 (1.65; 2.81) | 7.96 (7.57; 8.34) | 0.00 (0.00; 0.00) | 0.00 (0.00; 0.00) | 39.85 (39.29; 40.42) | 0.00 (0.00; 0.00) | 0.00 (0.00; 0.00) | 63.40 (62.79; 64.01) | 0.00 (0.00; 0.00) | 0.00 (0.00; 0.00) | 56.22 (55.43; 57.02) | 0.00 (0.00; 0.00) | 0.00 (0.00; 0.00) | 43.19 (42.73; 43.64) | 0.00 (0.00; 0.00) | 0.00 (0.00; 0.00) | 43.44 (42.99; 43.89) | 0.00 (0.00; 0.00) | 0.00 (0.00; 0.00) | 79.21 (78.72; 79.69) |
| **Guam** | 0.01 (0.01; 0.01) | 0.01 (0.01; 0.01) | -1.37 (-1.62; -1.13) | 0.00 (0.00; 0.00) | 0.00 (0.00; 0.00) | -1.38 (-1.62; -1.13) | 1.24 (0.84; 1.73) | 1.22 (0.81; 1.70) | -1.43 (-1.94; -0.93) | 46.41 (38.87; 53.98) | 45.82 (37.26; 55.70) | -1.43 (-1.69; -1.17) | 47.65 (39.94; 55.35) | 47.04 (38.47; 56.90) | -1.43 (-1.68; -1.18) | 1.46 (1.22; 1.70) | 1.44 (1.18; 1.73) | -1.31 (-1.56; -1.07) | 0.00 (0.00; 0.00) | 0.00 (0.00; 0.00) | -3.08 (-3.36; -2.79) | 0.00 (0.00; 0.00) | 0.00 (0.00; 0.00) | 11.36 (11.05; 11.66) | 0.00 (0.00; 0.00) | 0.00 (0.00; 0.00) | 5.95 (5.44; 6.46) | 0.00 (0.00; 0.00) | 0.00 (0.00; 0.00) | 2.01 (1.72; 2.30) | 0.00 (0.00; 0.00) | 0.00 (0.00; 0.00) | 2.11 (1.82; 2.40) | 0.00 (0.00; 0.00) | 0.00 (0.00; 0.00) | 27.45 (27.13; 27.77) |
| **Kiribati** | 0.01 (0.01; 0.02) | 0.01 (0.01; 0.02) | 3.16 (2.69; 3.63) | 0.01 (0.00; 0.01) | 0.01 (0.00; 0.01) | 3.02 (2.59; 3.46) | 1.80 (1.17; 2.76) | 1.89 (1.23; 2.90) | 4.56 (3.92; 5.20) | 136.80 (100.19; 184.50) | 126.48 (91.76; 169.60) | -6.98 (-7.41; -6.56) | 138.61 (101.81; 192.57) | 128.37 (93.56; 171.18) | -6.83 (-7.25; -6.42) | 4.72 (3.46; 6.24) | 4.67 (3.42; 6.16) | -0.82 (-1.23; -0.42) | 0.00 (0.00; 0.00) | 0.00 (0.00; 0.00) | 68.84 (68.10; 69.58) | 0.00 (0.00; 0.00) | 0.00 (0.00; 0.00) | 83.42 (82.65; 84.19) | 0.00 (0.00; 0.00) | 0.00 (0.00; 0.00) | 80.10 (79.13; 81.07) | 0.00 (0.00; 0.00) | 0.00 (0.00; 0.00) | 55.25 (54.64; 55.86) | 0.00 (0.00; 0.00) | 0.00 (0.00; 0.00) | 55.57 (54.96; 56.17) | 0.00 (0.00; 0.00) | 0.00 (0.00; 0.00) | 81.73 (81.08; 82.38) |
| **Marshall Islands** | 0.01 (0.00; 0.01) | 0.01 (0.00; 0.01) | 11.28 (10.69; 11.86) | 0.00 (0.00; 0.00) | 0.00 (0.00; 0.00) | 9.15 (8.63; 9.66) | 0.86 (0.52; 1.31) | 0.98 (0.60; 1.48) | 12.41 (11.73; 13.10) | 50.99 (35.07; 73.99) | 53.18 (35.81; 79.81) | 4.25 (3.66; 4.84) | 51.79 (34.98; 73.46) | 54.16 (36.66; 80.82) | 4.38 (3.80; 4.97) | 1.86 (1.34; 2.57) | 2.01 (1.43; 2.82) | 7.03 (6.52; 7.53) | 0.00 (0.00; 0.00) | 0.00 (0.00; 0.00) | 90.63 (89.73; 91.53) | 0.00 (0.00; 0.00) | 0.00 (0.00; 0.00) | 109.28 (108.36; 110.20) | 0.00 (0.00; 0.00) | 0.00 (0.00; 0.00) | 108.19 (107.08; 109.30) | 0.00 (0.00; 0.00) | 0.00 (0.00; 0.00) | 85.33 (84.41; 86.25) | 0.00 (0.00; 0.00) | 0.00 (0.00; 0.00) | 85.70 (84.79; 86.60) | 0.00 (0.00; 0.00) | 0.00 (0.00; 0.00) | 118.72 (117.82; 119.63) |
| **Micronesia (Federated States of)** | 0.01 (0.01; 0.01) | 0.01 (0.01; 0.01) | 11.65 (11.13; 12.16) | 0.00 (0.00; 0.00) | 0.00 (0.00; 0.00) | 8.27 (7.82; 8.72) | 0.93 (0.59; 1.35) | 1.08 (0.69; 1.55) | 14.89 (14.28; 15.51) | 51.99 (37.11; 72.16) | 51.31 (36.57; 72.08) | -0.84 (-1.32; -0.36) | 52.84 (38.25; 73.10) | 52.38 (37.71; 73.09) | -0.56 (-1.03; -0.09) | 1.91 (1.38; 2.59) | 2.01 (1.45; 2.69) | 4.79 (4.35; 5.24) | 0.00 (0.00; 0.00) | 0.00 (0.00; 0.00) | 53.46 (52.73; 54.19) | 0.00 (0.00; 0.00) | 0.00 (0.00; 0.00) | 65.37 (64.61; 66.13) | 0.00 (0.00; 0.00) | 0.00 (0.00; 0.00) | 68.98 (68.08; 69.87) | 0.00 (0.00; 0.00) | 0.00 (0.00; 0.00) | 40.18 (39.59; 40.76) | 0.00 (0.00; 0.00) | 0.00 (0.00; 0.00) | 40.67 (40.09; 41.25) | 0.00 (0.00; 0.00) | 0.00 (0.00; 0.00) | 69.88 (69.26; 70.50) |
| **Nauru** | 0.01 (0.01; 0.01) | 0.01 (0.01; 0.02) | 16.08 (15.33; 16.83) | 0.00 (0.00; 0.00) | 0.00 (0.00; 0.01) | 11.11 (10.42; 11.79) | 1.09 (0.59; 1.81) | 1.30 (0.72; 2.16) | 18.28 (17.42; 19.15) | 58.97 (37.39; 94.38) | 59.88 (36.68; 96.89) | 1.90 (1.17; 2.62) | 59.93 (38.27; 95.59) | 61.18 (38.00; 98.19) | 2.20 (1.49; 2.91) | 2.16 (1.43; 3.28) | 2.27 (1.46; 3.45) | 4.72 (4.09; 5.36) | 0.00 (0.00; 0.00) | 0.00 (0.00; 0.00) | 124.48 (123.14; 125.82) | 0.00 (0.00; 0.00) | 0.00 (0.00; 0.00) | 123.37 (122.08; 124.67) | 0.00 (0.00; 0.00) | 0.00 (0.00; 0.00) | 134.99 (133.49; 136.49) | 0.00 (0.00; 0.00) | 0.00 (0.00; 0.00) | 100.38 (99.23; 101.54) | 0.00 (0.00; 0.00) | 0.00 (0.00; 0.00) | 101 (99.86; 102.14) | 0.00 (0.00; 0.00) | 0.00 (0.00; 0.00) | 113.56 (112.48; 114.63) |
| **Niue** | 0.01 (0.01; 0.01) | 0.01 (0.01; 0.01) | 9.40 (9.02; 9.78) | 0.00 (0.00; 0.00) | 0.00 (0.00; 0.00) | 8.72 (8.36; 9.09) | 1.09 (0.71; 1.59) | 1.23 (0.80; 1.79) | 12 (11.40; 12.60) | 46.24 (35.16; 59.11) | 45.63 (34.95; 60.41) | -1.21 (-1.59; -0.83) | 47.33 (36.62; 60.20) | 46.86 (36.06; 61.75) | -0.90 (-1.28; -0.53) | 1.75 (1.36; 2.19) | 1.83 (1.44; 2.37) | 4.51 (4.15; 4.86) | 0.00 (0.00; 0.00) | 0.00 (0.00; 0.00) | 26.72 (26.26; 27.17) | 0.00 (0.00; 0.00) | 0.00 (0.00; 0.00) | 34.47 (34; 34.95) | 0.00 (0.00; 0.00) | 0.00 (0.00; 0.00) | 35.13 (34.46; 35.80) | 0.00 (0.00; 0.00) | 0.00 (0.00; 0.00) | 16.74 (16.32; 17.17) | 0.00 (0.00; 0.00) | 0.00 (0.00; 0.00) | 17.17 (16.75; 17.59) | 0.00 (0.00; 0.00) | 0.00 (0.00; 0.00) | 35.28 (34.84; 35.72) |
| **Northern Mariana Islands** | 0.05 (0.04; 0.06) | 0.05 (0.04; 0.05) | -3.20 (-3.47; -2.93) | 0.01 (0.01; 0.02) | 0.01 (0.01; 0.02) | -2.73 (-2.98; -2.47) | 4.86 (3.34; 6.66) | 4.74 (3.23; 6.42) | -2.33 (-2.80; -1.86) | 184.35 (148.50; 219.11) | 170.16 (137.11; 206.37) | -6.98 (-7.24; -6.71) | 189.26 (154.33; 225.59) | 174.90 (142.23; 211.08) | -6.86 (-7.11; -6.60) | 7.21 (5.89; 8.53) | 7.07 (5.73; 8.52) | -2.18 (-2.44; -1.92) | 0.00 (0.00; 0.00) | 0.00 (0.00; 0.00) | 27.58 (27.25; 27.92) | 0.00 (0.00; 0.00) | 0.00 (0.00; 0.00) | 57.11 (56.73; 57.48) | 0.00 (0.00; 0.00) | 0.00 (0.00; 0.00) | 42.21 (41.62; 42.81) | 0.00 (0.00; 0.00) | 0.00 (0.00; 0.00) | 32.75 (32.41; 33.08) | 0.00 (0.00; 0.00) | 0.00 (0.00; 0.00) | 32.99 (32.66; 33.32) | 0.00 (0.00; 0.00) | 0.00 (0.00; 0.00) | 87.87 (87.43; 88.31) |
| **Palau** | 0.09 (0.07; 0.12) | 0.09 (0.07; 0.12) | -0.92 (-1.29; -0.56) | 0.03 (0.02; 0.03) | 0.03 (0.02; 0.03) | -2.78 (-3.12; -2.44) | 9.41 (6.20; 13.85) | 9.27 (6.07; 13.73) | -1.58 (-2.15; -1.00) | 422.97 (324.86; 541.24) | 454.12 (343.61; 591.88) | 6.89 (6.50; 7.28) | 432.66 (329.61; 558.56) | 463.38 (352.47; 600.75) | 6.71 (6.33; 7.08) | 15.47 (12.08; 19.56) | 16.64 (12.83; 21.37) | 7.20 (6.84; 7.57) | 0.00 (0.00; 0.00) | 0.00 (0.00; 0.00) | 0.56 (0.15; 0.97) | 0.00 (0.00; 0.00) | 0.00 (0.00; 0.00) | 21.99 (21.53; 22.44) | 0.00 (0.00; 0.00) | 0.00 (0.00; 0.00) | 10.97 (10.32; 11.62) | 0.00 (0.00; 0.00) | 0.00 (0.00; 0.00) | 17.63 (17.21; 18.06) | 0.00 (0.00; 0.00) | 0.00 (0.00; 0.00) | 17.49 (17.08; 17.91) | 0.00 (0.00; 0.00) | 0.00 (0.00; 0.00) | 60.96 (60.51; 61.42) |
| **Papua New Guinea** | 0.00 (0.00; 0.01) | 0.00 (0.00; 0.01) | -0.68 (-1.25; -0.11) | 0.00 (0.00; 0.00) | 0.00 (0.00; 0.00) | -4.08 (-4.64; -3.53) | 0.62 (0.36; 0.97) | 0.62 (0.35; 0.94) | 0.70 (0.01; 1.38) | 37.28 (22.48; 52.18) | 34.02 (20.03; 49.21) | -8.40 (-8.97; -7.84) | 37.89 (22.82; 53.98) | 34.64 (20.64; 49.94) | -8.25 (-8.81; -7.70) | 1.38 (0.85; 1.92) | 1.31 (0.79; 1.91) | -5.52 (-6.09; -4.95) | 0.00 (0.00; 0.00) | 0.00 (0.00; 0.00) | 152.14 (150.97; 153.31) | 0.00 (0.00; 0.00) | 0.00 (0.00; 0.00) | 167.18 (165.97; 168.39) | 0.00 (0.00; 0.00) | 0.00 (0.00; 0.00) | 172.85 (171.34; 174.35) | 0.00 (0.00; 0.00) | 0.01 (0.00; 0.01) | 139.65 (138.58; 140.71) | 0.00 (0.00; 0.00) | 0.01 (0.00; 0.01) | 140.16 (139.11; 141.20) | 0.00 (0.00; 0.00) | 0.00 (0.00; 0.00) | 177.30 (176.19; 178.42) |
| **Samoa** | 0.01 (0.00; 0.01) | 0.01 (0.00; 0.01) | -1.77 (-2.20; -1.35) | 0.00 (0.00; 0.00) | 0.00 (0.00; 0.00) | -2.50 (-2.88; -2.12) | 0.75 (0.48; 1.09) | 0.75 (0.49; 1.09) | -0.45 (-1.02; 0.12) | 36.85 (26.24; 48.09) | 33.89 (24.02; 45.31) | -7.48 (-7.89; -7.06) | 37.60 (26.78; 48.89) | 34.64 (24.79; 46.06) | -7.33 (-7.74; -6.92) | 1.35 (1.00; 1.73) | 1.30 (0.95; 1.74) | -3.21 (-3.62; -2.81) | 0.00 (0.00; 0.00) | 0.00 (0.00; 0.00) | 59.76 (59.14; 60.39) | 0.00 (0.00; 0.00) | 0.00 (0.00; 0.00) | 65.23 (64.63; 65.82) | 0.00 (0.00; 0.00) | 0.00 (0.00; 0.00) | 66.16 (65.35; 66.97) | 0.00 (0.00; 0.00) | 0.00 (0.00; 0.00) | 51.36 (50.79; 51.92) | 0.00 (0.00; 0.00) | 0.00 (0.00; 0.00) | 51.65 (51.10; 52.20) | 0.00 (0.00; 0.00) | 0.00 (0.00; 0.00) | 68.82 (68.27; 69.37) |
| **Solomon Islands** | 0.01 (0.00; 0.01) | 0.01 (0.00; 0.01) | 9.47 (8.95; 9.98) | 0.00 (0.00; 0.00) | 0.00 (0.00; 0.00) | 4.95 (4.47; 5.42) | 0.78 (0.50; 1.17) | 0.88 (0.57; 1.31) | 11.72 (11.08; 12.36) | 47.14 (33.06; 66.65) | 45.94 (31.40; 64.42) | -2.27 (-2.77; -1.77) | 47.91 (33.34; 67.66) | 46.82 (32.09; 65.20) | -2.04 (-2.53; -1.55) | 1.71 (1.22; 2.34) | 1.74 (1.20; 2.43) | 1.54 (1.06; 2.03) | 0.00 (0.00; 0.00) | 0.00 (0.00; 0.00) | 105.25 (104.31; 106.19) | 0.00 (0.00; 0.00) | 0.00 (0.00; 0.00) | 111.55 (110.62; 112.48) | 0.00 (0.00; 0.00) | 0.00 (0.00; 0.00) | 120.46 (119.29; 121.63) | 0.00 (0.00; 0.00) | 0.00 (0.00; 0.00) | 86.42 (85.65; 87.19) | 0.00 (0.00; 0.00) | 0.00 (0.00; 0.00) | 86.95 (86.19; 87.71) | 0.00 (0.00; 0.00) | 0.00 (0.00; 0.00) | 111.41 (110.65; 112.17) |
| **Tokelau** | 0.01 (0.01; 0.01) | 0.01 (0.01; 0.02) | 19.25 (18.80; 19.71) | 0.00 (0.00; 0.00) | 0.00 (0.00; 0.00) | 13.92 (13.48; 14.35) | 1.06 (0.71; 1.57) | 1.30 (0.84; 1.92) | 20.85 (20.20; 21.50) | 46.97 (34.84; 62.75) | 49.56 (36.43; 68.18) | 5.53 (5.09; 5.97) | 48.02 (35.43; 63.59) | 50.86 (37.74; 69.29) | 5.87 (5.44; 6.30) | 1.72 (1.30; 2.25) | 1.88 (1.40; 2.54) | 8.76 (8.33; 9.18) | 0.00 (0.00; 0.00) | 0.00 (0.00; 0.00) | 70.03 (69.38; 70.68) | 0.00 (0.00; 0.00) | 0.00 (0.00; 0.00) | 69.07 (68.46; 69.68) | 0.00 (0.00; 0.00) | 0.00 (0.00; 0.00) | 77.24 (76.37; 78.11) | 0.00 (0.00; 0.00) | 0.00 (0.00; 0.00) | 51.81 (51.28; 52.34) | 0.00 (0.00; 0.00) | 0.00 (0.00; 0.00) | 52.38 (51.86; 52.90) | 0.00 (0.00; 0.00) | 0.00 (0.00; 0.00) | 65.74 (65.21; 66.27) |
| **Tonga** | 0.01 (0.01; 0.01) | 0.01 (0.01; 0.01) | 1.15 (0.64; 1.66) | 0.00 (0.00; 0.00) | 0.00 (0.00; 0.00) | -0.48 (-0.95; -0.01) | 0.88 (0.55; 1.32) | 0.90 (0.54; 1.38) | 2.27 (1.63; 2.91) | 39.82 (27.56; 55.39) | 37.77 (25.70; 52.96) | -4.76 (-5.25; -4.28) | 40.71 (28.24; 55.72) | 38.67 (26.54; 53.88) | -4.61 (-5.08; -4.14) | 1.54 (1.09; 2.11) | 1.52 (1.04; 2.09) | -1.66 (-2.13; -1.19) | 0.00 (0.00; 0.00) | 0.00 (0.00; 0.00) | 51 (50.31; 51.68) | 0.00 (0.00; 0.00) | 0.00 (0.00; 0.00) | 58.47 (57.80; 59.13) | 0.00 (0.00; 0.00) | 0.00 (0.00; 0.00) | 59.02 (58.17; 59.87) | 0.00 (0.00; 0.00) | 0.00 (0.00; 0.00) | 44.02 (43.41; 44.63) | 0.00 (0.00; 0.00) | 0.00 (0.00; 0.00) | 44.34 (43.75; 44.93) | 0.00 (0.00; 0.00) | 0.00 (0.00; 0.00) | 63.23 (62.62; 63.85) |
| **Tuvalu** | 0.01 (0.01; 0.01) | 0.01 (0.01; 0.01) | 12.56 (12.16; 12.95) | 0.00 (0.00; 0.00) | 0.00 (0.00; 0.00) | 8.92 (8.55; 9.28) | 0.89 (0.60; 1.29) | 1.03 (0.69; 1.49) | 14.76 (14.17; 15.35) | 46.44 (36.30; 59.73) | 47.22 (36.37; 61.44) | 1.71 (1.34; 2.08) | 47.32 (37.08; 61.06) | 48.25 (37.37; 62.66) | 1.96 (1.59; 2.32) | 1.72 (1.38; 2.17) | 1.83 (1.44; 2.32) | 5.44 (5.10; 5.78) | 0.00 (0.00; 0.00) | 0.00 (0.00; 0.00) | 77.87 (77.28; 78.46) | 0.00 (0.00; 0.00) | 0.00 (0.00; 0.00) | 77.64 (77.08; 78.21) | 0.00 (0.00; 0.00) | 0.00 (0.00; 0.00) | 85.21 (84.34; 86.07) | 0.00 (0.00; 0.00) | 0.00 (0.00; 0.00) | 62.87 (62.37; 63.36) | 0.00 (0.00; 0.00) | 0.00 (0.00; 0.00) | 63.28 (62.80; 63.77) | 0.00 (0.00; 0.00) | 0.00 (0.00; 0.00) | 75.58 (75.09; 76.06) |
| **Vanuatu** | 0.01 (0.00; 0.01) | 0.00 (0.00; 0.01) | -6.90 (-7.30; -6.51) | 0.00 (0.00; 0.00) | 0.00 (0.00; 0.00) | -6.56 (-6.93; -6.19) | 0.70 (0.48; 1.01) | 0.66 (0.44; 0.95) | -5.79 (-6.32; -5.27) | 41.62 (31.78; 56.87) | 37.19 (27.90; 50.48) | -10.01 (-10.42; -9.60) | 42.31 (33.04; 57.19) | 37.85 (28.75; 51.24) | -9.94 (-10.34; -9.54) | 1.55 (1.22; 2.03) | 1.45 (1.12; 1.88) | -6.18 (-6.54; -5.82) | 0.00 (0.00; 0.00) | 0.00 (0.00; 0.00) | 90.91 (90.25; 91.58) | 0.00 (0.00; 0.00) | 0.00 (0.00; 0.00) | 105.46 (104.80; 106.12) | 0.00 (0.00; 0.00) | 0.00 (0.00; 0.00) | 102.77 (101.84; 103.70) | 0.00 (0.00; 0.00) | 0.00 (0.00; 0.00) | 88.32 (87.64; 89.01) | 0.00 (0.00; 0.00) | 0.00 (0.00; 0.00) | 88.55 (87.88; 89.23) | 0.00 (0.00; 0.00) | 0.00 (0.00; 0.00) | 115.05 (114.40; 115.69) |
| **Southeast Asia** | 0.01 (0.01; 0.02) | 0.02 (0.01; 0.02) | 16.71 (16.46; 16.96) | 0.00 (0.00; 0.00) | 0.00 (0.00; 0.01) | 10.85 (10.63; 11.07) | 1.54 (1.12; 2.05) | 1.86 (1.34; 2.50) | 19.15 (18.67; 19.64) | 59.42 (51.52; 67.89) | 66.01 (57.10; 75.84) | 10.72 (10.51; 10.92) | 60.97 (52.95; 69.50) | 67.87 (58.77; 77.41) | 10.93 (10.73; 11.14) | 2.49 (2.17; 2.82) | 2.82 (2.46; 3.21) | 12.25 (12.05; 12.45) | 0.10 (0.08; 0.11) | 0.21 (0.17; 0.24) | 102.13 (101.76; 102.51) | 0.03 (0.02; 0.03) | 0.06 (0.05; 0.07) | 120.59 (120.23; 120.95) | 0.01 (0.01; 0.01) | 0.02 (0.02; 0.03) | 122.99 (122.26; 123.72) | 0.41 (0.36; 0.47) | 0.84 (0.74; 0.95) | 99.95 (99.67; 100.23) | 0.42 (0.37; 0.48) | 0.87 (0.77; 0.97) | 100.53 (100.26; 100.81) | 0.02 (0.01; 0.02) | 0.04 (0.04; 0.04) | 149.34 (149.01; 149.66) |
| **Cambodia** | 0.01 (0.01; 0.01) | 0.01 (0.01; 0.02) | 45.46 (44.90; 46.01) | 0.00 (0.00; 0.00) | 0.00 (0.00; 0.01) | 23.50 (23.03; 23.96) | 1.24 (0.80; 1.86) | 1.85 (1.16; 2.85) | 46.55 (45.76; 47.34) | 63.29 (46.02; 84.60) | 70.54 (50.63; 95.66) | 10.95 (10.49; 11.42) | 64.54 (47.20; 86.75) | 72.40 (52.62; 97.77) | 11.65 (11.19; 12.11) | 2.63 (1.97; 3.39) | 2.96 (2.16; 3.91) | 11.41 (10.98; 11.83) | 0.00 (0.00; 0.00) | 0.00 (0.00; 0.01) | 183.23 (182.09; 184.36) | 0.00 (0.00; 0.00) | 0.00 (0.00; 0.00) | 163.72 (162.70; 164.74) | 0.00 (0.00; 0.00) | 0.00 (0.00; 0.00) | 201.32 (199.86; 202.78) | 0.01 (0.01; 0.01) | 0.02 (0.01; 0.03) | 123.99 (123.17; 124.81) | 0.01 (0.01; 0.01) | 0.02 (0.01; 0.03) | 125.48 (124.66; 126.29) | 0.00 (0.00; 0.00) | 0.00 (0.00; 0.00) | 151.09 (150.25; 151.94) |
| **Indonesia** | 0.01 (0.01; 0.01) | 0.01 (0.01; 0.01) | 8.84 (8.43; 9.25) | 0.00 (0.00; 0.00) | 0.00 (0.00; 0.00) | 3.88 (3.49; 4.27) | 0.96 (0.60; 1.35) | 1.07 (0.67; 1.51) | 10.36 (9.77; 10.94) | 45.19 (34.01; 56.96) | 43.61 (31.72; 56.77) | -3.23 (-3.61; -2.86) | 46.19 (33.93; 58.01) | 44.68 (32.81; 58.00) | -2.95 (-3.32; -2.58) | 1.92 (1.45; 2.39) | 1.95 (1.41; 2.52) | 0.73 (0.35; 1.11) | 0.02 (0.02; 0.03) | 0.04 (0.03; 0.06) | 98.95 (98.31; 99.60) | 0.01 (0.00; 0.01) | 0.02 (0.01; 0.02) | 126.12 (125.40; 126.85) | 0.00 (0.00; 0.00) | 0.01 (0.00; 0.01) | 125.25 (124.29; 126.22) | 0.12 (0.09; 0.15) | 0.23 (0.17; 0.29) | 88.52 (87.95; 89.09) | 0.12 (0.09; 0.15) | 0.23 (0.17; 0.29) | 89.28 (88.72; 89.84) | 0.00 (0.00; 0.01) | 0.01 (0.01; 0.01) | 144.69 (144; 145.39) |
| **Lao People's Democratic Republic** | 0.01 (0.00; 0.01) | 0.01 (0.01; 0.01) | 37.83 (37.29; 38.37) | 0.00 (0.00; 0.00) | 0.00 (0.00; 0.00) | 22 (21.55; 22.46) | 0.89 (0.57; 1.30) | 1.27 (0.82; 1.88) | 40.24 (39.53; 40.95) | 49.21 (36.08; 65.05) | 54.67 (39.55; 74.92) | 9.90 (9.43; 10.36) | 50.07 (36.38; 64.22) | 55.94 (40.65; 76.23) | 10.44 (9.98; 10.89) | 2.02 (1.50; 2.67) | 2.35 (1.75; 3.12) | 13.78 (13.35; 14.21) | 0.00 (0.00; 0.00) | 0.00 (0.00; 0.00) | 200.14 (199.07; 201.21) | 0.00 (0.00; 0.00) | 0.00 (0.00; 0.00) | 200.74 (199.73; 201.74) | 0.00 (0.00; 0.00) | 0.00 (0.00; 0.00) | 233.58 (232.09; 235.07) | 0.00 (0.00; 0.00) | 0.01 (0.01; 0.01) | 150.63 (149.85; 151.42) | 0.00 (0.00; 0.00) | 0.01 (0.01; 0.01) | 152.06 (151.29; 152.83) | 0.00 (0.00; 0.00) | 0.00 (0.00; 0.00) | 198.91 (198.07; 199.75) |
| **Malaysia** | 0.02 (0.02; 0.02) | 0.02 (0.02; 0.03) | 20.70 (20.39; 21) | 0.01 (0.00; 0.01) | 0.01 (0.00; 0.01) | 11.81 (11.53; 12.10) | 1.96 (1.34; 2.64) | 2.41 (1.59; 3.34) | 21.49 (20.93; 22.04) | 68.80 (57.59; 83.93) | 69.78 (56.81; 85.19) | 0.81 (0.52; 1.09) | 70.76 (59.20; 84.83) | 72.19 (59.05; 88.53) | 1.38 (1.10; 1.67) | 2.86 (2.42; 3.41) | 3.02 (2.43; 3.67) | 4.05 (3.77; 4.32) | 0.01 (0.00; 0.01) | 0.01 (0.01; 0.02) | 134.72 (134.13; 135.31) | 0.00 (0.00; 0.00) | 0.00 (0.00; 0.00) | 138.59 (138; 139.18) | 0.00 (0.00; 0.00) | 0.00 (0.00; 0.00) | 148.71 (147.72; 149.69) | 0.02 (0.02; 0.03) | 0.04 (0.03; 0.06) | 106.03 (105.48; 106.58) | 0.02 (0.02; 0.03) | 0.04 (0.04; 0.06) | 107.22 (106.67; 107.77) | 0.00 (0.00; 0.00) | 0.00 (0.00; 0.00) | 142.66 (142.03; 143.29) |
| **Maldives** | 0.01 (0.01; 0.02) | 0.01 (0.01; 0.02) | 12.23 (11.81; 12.64) | 0.00 (0.00; 0.00) | 0.00 (0.00; 0.00) | 1.34 (0.98; 1.69) | 1.32 (0.88; 1.89) | 1.51 (1.02; 2.15) | 15.20 (14.62; 15.78) | 40.15 (30.71; 50.79) | 35.48 (26.82; 46.04) | -10.42 (-10.77; -10.07) | 41.48 (31.65; 52.25) | 36.99 (28.49; 47.18) | -9.60 (-9.93; -9.27) | 1.98 (1.55; 2.46) | 1.91 (1.47; 2.43) | -3.79 (-4.13; -3.46) | 0.00 (0.00; 0.00) | 0.00 (0.00; 0.00) | 317.91 (316.59; 319.23) | 0.00 (0.00; 0.00) | 0.00 (0.00; 0.00) | 341.48 (340.17; 342.80) | 0.00 (0.00; 0.00) | 0.00 (0.00; 0.00) | 375.22 (373.28; 377.15) | 0.00 (0.00; 0.00) | 0.00 (0.00; 0.00) | 258.26 (257.26; 259.26) | 0.00 (0.00; 0.00) | 0.00 (0.00; 0.00) | 261.95 (260.98; 262.91) | 0.00 (0.00; 0.00) | 0.00 (0.00; 0.00) | 377.73 (376.54; 378.92) |
| **Mauritius** | 0.02 (0.01; 0.02) | 0.02 (0.01; 0.02) | 2.95 (2.79; 3.11) | 0.00 (0.00; 0.00) | 0.00 (0.00; 0.01) | 0.61 (0.47; 0.76) | 1.69 (1.21; 2.27) | 1.76 (1.21; 2.36) | 4.41 (3.95; 4.87) | 62.73 (57.70; 66.62) | 60.09 (52.92; 67.99) | -3.86 (-4.00; -3.72) | 64.42 (59.47; 68.28) | 61.85 (54.47; 70.29) | -3.64 (-3.79; -3.50) | 2.51 (2.31; 2.66) | 2.50 (2.20; 2.81) | -0.41 (-0.56; -0.27) | 0.00 (0.00; 0.00) | 0.00 (0.00; 0.00) | 23.88 (23.60; 24.15) | 0.00 (0.00; 0.00) | 0.00 (0.00; 0.00) | 43.58 (43.29; 43.86) | 0.00 (0.00; 0.00) | 0.00 (0.00; 0.00) | 37.82 (37.25; 38.40) | 0.00 (0.00; 0.00) | 0.00 (0.00; 0.00) | 24.92 (24.66; 25.19) | 0.00 (0.00; 0.00) | 0.00 (0.00; 0.00) | 25.27 (25; 25.53) | 0.00 (0.00; 0.00) | 0.00 (0.00; 0.00) | 64.41 (64.09; 64.73) |
| **Myanmar** | 0.01 (0.00; 0.01) | 0.01 (0.01; 0.01) | 31.10 (30.54; 31.65) | 0.00 (0.00; 0.00) | 0.00 (0.00; 0.00) | 20.16 (19.66; 20.65) | 0.89 (0.55; 1.30) | 1.20 (0.76; 1.74) | 33.78 (33.08; 34.47) | 43.55 (31.01; 60.21) | 48.95 (34.72; 68.08) | 11.92 (11.43; 12.42) | 44.41 (32.17; 59.90) | 50.15 (35.70; 69.45) | 12.36 (11.87; 12.85) | 1.81 (1.33; 2.47) | 2.07 (1.50; 2.82) | 13.45 (12.97; 13.92) | 0.00 (0.00; 0.00) | 0.01 (0.01; 0.01) | 118.44 (117.58; 119.30) | 0.00 (0.00; 0.00) | 0.00 (0.00; 0.00) | 124.64 (123.77; 125.51) | 0.00 (0.00; 0.00) | 0.00 (0.00; 0.00) | 141.48 (140.31; 142.65) | 0.02 (0.02; 0.03) | 0.04 (0.03; 0.06) | 93.40 (92.72; 94.09) | 0.02 (0.02; 0.03) | 0.05 (0.03; 0.06) | 94.35 (93.68; 95.01) | 0.00 (0.00; 0.00) | 0.00 (0.00; 0.00) | 128.11 (127.38; 128.85) |
| **Philippines** | 0.01 (0.01; 0.01) | 0.01 (0.01; 0.01) | 7.79 (7.54; 8.04) | 0.00 (0.00; 0.00) | 0.00 (0.00; 0.00) | 7.37 (7.13; 7.62) | 0.91 (0.64; 1.25) | 1.00 (0.69; 1.36) | 8.45 (7.96; 8.94) | 46.34 (38.82; 54.91) | 48.20 (39.92; 58.47) | 4.10 (3.83; 4.37) | 47.25 (39.92; 55.29) | 49.20 (40.87; 59.46) | 4.18 (3.92; 4.45) | 1.86 (1.58; 2.19) | 1.99 (1.66; 2.39) | 6.28 (6.03; 6.54) | 0.01 (0.01; 0.01) | 0.01 (0.01; 0.02) | 116.48 (115.97; 117) | 0.00 (0.00; 0.00) | 0.01 (0.00; 0.01) | 143.30 (142.74; 143.86) | 0.00 (0.00; 0.00) | 0.00 (0.00; 0.00) | 135.27 (134.31; 136.23) | 0.04 (0.03; 0.05) | 0.09 (0.08; 0.11) | 119.35 (118.88; 119.83) | 0.04 (0.04; 0.05) | 0.10 (0.08; 0.12) | 119.66 (119.19; 120.12) | 0.00 (0.00; 0.00) | 0.00 (0.00; 0.00) | 160.34 (159.85; 160.83) |
| **Seychelles** | 0.03 (0.03; 0.04) | 0.04 (0.03; 0.04) | 6.56 (6.31; 6.81) | 0.01 (0.01; 0.01) | 0.01 (0.01; 0.01) | 5.84 (5.61; 6.06) | 3.68 (2.45; 4.96) | 4.01 (2.68; 5.47) | 8.05 (7.54; 8.56) | 159.08 (133.04; 186.35) | 163.68 (132.23; 202.14) | 3.14 (2.86; 3.42) | 162.75 (137.26; 189.83) | 167.69 (135.83; 205.89) | 3.25 (2.98; 3.52) | 6.34 (5.36; 7.38) | 6.92 (5.63; 8.40) | 8.51 (8.24; 8.78) | 0.00 (0.00; 0.00) | 0.00 (0.00; 0.00) | 69.68 (69.25; 70.11) | 0.00 (0.00; 0.00) | 0.00 (0.00; 0.00) | 93.95 (93.48; 94.42) | 0.00 (0.00; 0.00) | 0.00 (0.00; 0.00) | 85.17 (84.38; 85.96) | 0.00 (0.00; 0.00) | 0.00 (0.00; 0.00) | 73.43 (72.97; 73.88) | 0.00 (0.00; 0.00) | 0.00 (0.00; 0.00) | 73.69 (73.24; 74.14) | 0.00 (0.00; 0.00) | 0.00 (0.00; 0.00) | 120.40 (119.85; 120.95) |
| **Sri Lanka** | 0.03 (0.02; 0.05) | 0.04 (0.02; 0.06) | 24.49 (23.84; 25.13) | 0.01 (0.01; 0.01) | 0.01 (0.01; 0.01) | 18.37 (17.76; 18.98) | 3.23 (1.93; 5.10) | 4.10 (2.34; 6.40) | 25.74 (24.94; 26.53) | 107.21 (67.42; 156.10) | 122.63 (77.35; 176.88) | 14.51 (13.90; 15.13) | 110.56 (69.19; 157.92) | 126.73 (81.54; 180.99) | 14.85 (14.25; 15.45) | 4.46 (2.87; 6.31) | 5.36 (3.51; 7.63) | 19.21 (18.63; 19.80) | 0.01 (0.01; 0.01) | 0.02 (0.01; 0.02) | 73.79 (72.92; 74.67) | 0.00 (0.00; 0.00) | 0.00 (0.00; 0.01) | 92.79 (91.86; 93.72) | 0.00 (0.00; 0.00) | 0.00 (0.00; 0.00) | 89.55 (88.43; 90.66) | 0.03 (0.02; 0.04) | 0.05 (0.04; 0.07) | 73.06 (72.32; 73.80) | 0.03 (0.02; 0.04) | 0.05 (0.04; 0.07) | 73.55 (72.84; 74.27) | 0.00 (0.00; 0.00) | 0.00 (0.00; 0.00) | 124.66 (123.89; 125.42) |
| **Thailand** | 0.03 (0.02; 0.03) | 0.03 (0.02; 0.04) | 24.72 (24.32; 25.13) | 0.01 (0.01; 0.01) | 0.01 (0.01; 0.01) | 12.83 (12.44; 13.21) | 2.56 (1.71; 3.57) | 3.23 (2.16; 4.57) | 25.31 (24.72; 25.91) | 77.15 (59.73; 99.54) | 77.90 (59.37; 102.49) | 1.04 (0.66; 1.43) | 79.71 (61.59; 100.69) | 81.12 (62.50; 105.71) | 1.83 (1.46; 2.20) | 3.14 (2.44; 3.95) | 3.27 (2.50; 4.22) | 3.51 (3.13; 3.88) | 0.03 (0.02; 0.03) | 0.04 (0.03; 0.06) | 53.93 (53.38; 54.49) | 0.01 (0.01; 0.01) | 0.01 (0.01; 0.02) | 74.25 (73.62; 74.88) | 0.00 (0.00; 0.00) | 0.00 (0.00; 0.01) | 72.42 (71.63; 73.21) | 0.08 (0.06; 0.10) | 0.11 (0.09; 0.15) | 39.20 (38.73; 39.66) | 0.08 (0.06; 0.11) | 0.12 (0.09; 0.15) | 40.27 (39.82; 40.72) | 0.00 (0.00; 0.00) | 0.01 (0.01; 0.01) | 104.04 (103.47; 104.61) |
| **Timor-Leste** | 0.01 (0.00; 0.01) | 0.01 (0.01; 0.01) | 45.93 (45.33; 46.52) | 0.00 (0.00; 0.00) | 0.00 (0.00; 0.00) | 26.05 (25.54; 26.55) | 0.79 (0.49; 1.18) | 1.20 (0.74; 1.76) | 47.49 (46.73; 48.26) | 41.86 (30.16; 56.02) | 47.33 (33.07; 64.63) | 11.69 (11.20; 12.17) | 42.64 (30.99; 56.59) | 48.53 (34.01; 65.85) | 12.36 (11.88; 12.84) | 1.74 (1.28; 2.32) | 2.02 (1.44; 2.72) | 13.93 (13.45; 14.40) | 0.00 (0.00; 0.00) | 0.00 (0.00; 0.00) | 195.10 (194.01; 196.19) | 0.00 (0.00; 0.00) | 0.00 (0.00; 0.00) | 162.86 (161.90; 163.82) | 0.00 (0.00; 0.00) | 0.00 (0.00; 0.00) | 204.74 (203.24; 206.25) | 0.00 (0.00; 0.00) | 0.00 (0.00; 0.00) | 128.56 (127.72; 129.41) | 0.00 (0.00; 0.00) | 0.00 (0.00; 0.00) | 129.97 (129.13; 130.81) | 0.00 (0.00; 0.00) | 0.00 (0.00; 0.00) | 141.35 (140.58; 142.13) |
| **Viet Nam** | 0.02 (0.02; 0.03) | 0.04 (0.03; 0.05) | 57.30 (56.78; 57.82) | 0.01 (0.00; 0.01) | 0.01 (0.01; 0.01) | 47.03 (46.56; 47.50) | 2.32 (1.53; 3.23) | 3.79 (2.41; 5.42) | 58.95 (58.22; 59.69) | 80.93 (60.71; 102.82) | 137.27 (103.94; 178.15) | 66.04 (65.52; 66.55) | 83.23 (63.08; 106.35) | 141.05 (108.26; 181.76) | 65.84 (65.34; 66.33) | 3.13 (2.42; 3.87) | 5.28 (4.11; 6.65) | 64.58 (64.13; 65.04) | 0.02 (0.02; 0.03) | 0.06 (0.05; 0.09) | 146.10 (145.27; 146.93) | 0.01 (0.00; 0.01) | 0.02 (0.01; 0.02) | 156.95 (156.13; 157.78) | 0.00 (0.00; 0.00) | 0.01 (0.00; 0.01) | 165.48 (164.26; 166.71) | 0.09 (0.06; 0.11) | 0.24 (0.19; 0.31) | 166.07 (165.33; 166.80) | 0.09 (0.07; 0.12) | 0.25 (0.19; 0.32) | 166.05 (165.35; 166.75) | 0.00 (0.00; 0.00) | 0.01 (0.01; 0.01) | 214.32 (213.59; 215.04) |
| **Sub-Saharan Africa** | 0.01 (0.00; 0.01) | 0.01 (0.01; 0.01) | 8.82 (8.55; 9.08) | 0.00 (0.00; 0.00) | 0.00 (0.00; 0.00) | -0.04 (-0.26; 0.19) | 0.81 (0.59; 1.11) | 0.91 (0.65; 1.25) | 11.35 (10.87; 11.84) | 48.63 (41.01; 57.03) | 45.15 (36.94; 53.73) | -6.68 (-6.91; -6.44) | 49.45 (41.76; 57.99) | 46.06 (37.66; 54.59) | -6.38 (-6.61; -6.14) | 1.85 (1.59; 2.13) | 1.80 (1.51; 2.11) | -3.03 (-3.25; -2.81) | 0.04 (0.03; 0.04) | 0.11 (0.09; 0.13) | 196.12 (195.54; 196.70) | 0.01 (0.01; 0.02) | 0.04 (0.03; 0.04) | 187.51 (186.99; 188.03) | 0.00 (0.00; 0.01) | 0.01 (0.01; 0.02) | 214.17 (213.07; 215.27) | 0.28 (0.23; 0.33) | 0.74 (0.61; 0.87) | 158.60 (158.12; 159.09) | 0.28 (0.24; 0.34) | 0.75 (0.62; 0.88) | 159.49 (159.01; 159.96) | 0.01 (0.01; 0.01) | 0.03 (0.02; 0.03) | 186.51 (186.07; 186.95) |
| **Central Sub-Saharan Africa** | 0.01 (0.00; 0.01) | 0.01 (0.00; 0.01) | 25.37 (24.92; 25.81) | 0.00 (0.00; 0.00) | 0.00 (0.00; 0.00) | 14.73 (14.31; 15.15) | 0.73 (0.49; 1.06) | 0.94 (0.62; 1.37) | 26.87 (26.23; 27.51) | 44.92 (33.99; 57.55) | 47.60 (35.16; 63.51) | 5.62 (5.21; 6.03) | 45.66 (34.71; 58.78) | 48.54 (36.07; 64.68) | 5.96 (5.55; 6.37) | 1.76 (1.33; 2.28) | 1.95 (1.42; 2.61) | 9.49 (9.05; 9.93) | 0.00 (0.00; 0.00) | 0.01 (0.01; 0.02) | 241.01 (239.90; 242.13) | 0.00 (0.00; 0.00) | 0.00 (0.00; 0.01) | 231.23 (230.15; 232.31) | 0.00 (0.00; 0.00) | 0.00 (0.00; 0.00) | 259.78 (258.13; 261.42) | 0.03 (0.02; 0.04) | 0.09 (0.06; 0.12) | 190.68 (189.72; 191.64) | 0.03 (0.02; 0.04) | 0.09 (0.07; 0.12) | 191.72 (190.77; 192.67) | 0.00 (0.00; 0.00) | 0.00 (0.00; 0.00) | 223.98 (222.95; 225.02) |
| **Angola** | 0.01 (0.00; 0.01) | 0.01 (0.01; 0.01) | 37.11 (36.53; 37.68) | 0.00 (0.00; 0.00) | 0.00 (0.00; 0.00) | 18.97 (18.47; 19.47) | 0.83 (0.53; 1.22) | 1.16 (0.73; 1.70) | 38.16 (37.45; 38.87) | 50.52 (35.42; 68.20) | 53.84 (37.39; 76.44) | 6 (5.50; 6.50) | 51.33 (37.03; 69.40) | 55.00 (38.58; 77.71) | 6.53 (6.03; 7.02) | 1.97 (1.42; 2.62) | 2.20 (1.54; 3.07) | 10.29 (9.79; 10.79) | 0.00 (0.00; 0.00) | 0.00 (0.00; 0.01) | 302.59 (301.03; 304.15) | 0.00 (0.00; 0.00) | 0.00 (0.00; 0.00) | 271.40 (270.02; 272.78) | 0.00 (0.00; 0.00) | 0.00 (0.00; 0.00) | 322.12 (320.16; 324.09) | 0.01 (0.01; 0.01) | 0.02 (0.02; 0.03) | 213.45 (212.30; 214.59) | 0.01 (0.01; 0.01) | 0.02 (0.02; 0.03) | 215.11 (213.98; 216.23) | 0.00 (0.00; 0.00) | 0.00 (0.00; 0.00) | 251.76 (250.64; 252.89) |
| **Central African Republic** | 0.00 (0.00; 0.01) | 0.00 (0.00; 0.01) | -2.03 (-2.52; -1.54) | 0.00 (0.00; 0.00) | 0.00 (0.00; 0.00) | -4.52 (-4.97; -4.07) | 0.64 (0.41; 0.97) | 0.64 (0.41; 0.97) | -0.33 (-0.95; 0.30) | 47.23 (32.25; 69.32) | 42.20 (28.34; 61.34) | -9.67 (-10.21; -9.14) | 47.80 (33.80; 68.70) | 42.84 (29.11; 62.07) | -9.54 (-10.07; -9.02) | 1.81 (1.27; 2.48) | 1.70 (1.18; 2.36) | -5.34 (-5.82; -4.87) | 0.00 (0.00; 0.00) | 0.00 (0.00; 0.00) | 80.69 (79.72; 81.65) | 0.00 (0.00; 0.00) | 0.00 (0.00; 0.00) | 86.89 (85.90; 87.87) | 0.00 (0.00; 0.00) | 0.00 (0.00; 0.00) | 91.70 (90.56; 92.85) | 0.00 (0.00; 0.00) | 0.00 (0.00; 0.00) | 68.11 (67.21; 69.02) | 0.00 (0.00; 0.00) | 0.00 (0.00; 0.00) | 68.41 (67.51; 69.30) | 0.00 (0.00; 0.00) | 0.00 (0.00; 0.00) | 89.20 (88.27; 90.13) |
| **Congo** | 0.01 (0.00; 0.01) | 0.01 (0.01; 0.01) | 36.23 (35.71; 36.76) | 0.00 (0.00; 0.00) | 0.00 (0.00; 0.00) | 25.61 (25.16; 26.05) | 0.93 (0.57; 1.37) | 1.30 (0.84; 1.89) | 38.30 (37.60; 39.01) | 54.08 (40.00; 70.84) | 62.92 (45.70; 86.06) | 16.87 (16.38; 17.35) | 54.98 (41.14; 72.74) | 64.22 (47.25; 87.51) | 17.23 (16.76; 17.71) | 2.12 (1.61; 2.70) | 2.57 (1.95; 3.39) | 21.11 (20.68; 21.54) | 0.00 (0.00; 0.00) | 0.00 (0.00; 0.00) | 208.66 (207.49; 209.84) | 0.00 (0.00; 0.00) | 0.00 (0.00; 0.00) | 212.44 (211.36; 213.52) | 0.00 (0.00; 0.00) | 0.00 (0.00; 0.00) | 235.38 (233.72; 237.03) | 0.00 (0.00; 0.00) | 0.00 (0.00; 0.01) | 170.57 (169.67; 171.47) | 0.00 (0.00; 0.00) | 0.01 (0.00; 0.01) | 171.62 (170.73; 172.51) | 0.00 (0.00; 0.00) | 0.00 (0.00; 0.00) | 214.16 (213.25; 215.06) |
| **Democratic Republic of the Congo** | 0.00 (0.00; 0.01) | 0.01 (0.00; 0.01) | 18.41 (17.92; 18.91) | 0.00 (0.00; 0.00) | 0.00 (0.00; 0.00) | 11.67 (11.19; 12.15) | 0.67 (0.43; 1.01) | 0.82 (0.52; 1.21) | 20.79 (20.13; 21.45) | 41.85 (29.90; 56.11) | 43.96 (30.65; 59.99) | 4.74 (4.27; 5.21) | 42.56 (30.48; 57.13) | 44.78 (31.22; 60.81) | 5 (4.53; 5.46) | 1.64 (1.16; 2.22) | 1.80 (1.23; 2.50) | 8.22 (7.71; 8.73) | 0.00 (0.00; 0.00) | 0.01 (0.00; 0.01) | 223.42 (222; 224.85) | 0.00 (0.00; 0.00) | 0.00 (0.00; 0.00) | 223.02 (221.59; 224.44) | 0.00 (0.00; 0.00) | 0.00 (0.00; 0.00) | 243.80 (241.89; 245.70) | 0.02 (0.01; 0.02) | 0.05 (0.03; 0.08) | 189.52 (188.20; 190.85) | 0.02 (0.01; 0.03) | 0.06 (0.03; 0.08) | 190.33 (189.03; 191.64) | 0.00 (0.00; 0.00) | 0.00 (0.00; 0.00) | 220.72 (219.25; 222.18) |
| **Equatorial Guinea** | 0.01 (0.01; 0.01) | 0.01 (0.01; 0.02) | 45.48 (44.77; 46.20) | 0.00 (0.00; 0.00) | 0.00 (0.00; 0.01) | 35.57 (34.94; 36.20) | 1.01 (0.59; 1.60) | 1.52 (0.92; 2.41) | 48.01 (47.14; 48.89) | 49.71 (32.76; 71.48) | 63.15 (41.44; 90.32) | 27.95 (27.33; 28.58) | 50.72 (33.30; 72.59) | 64.66 (43.01; 91.42) | 28.36 (27.75; 28.97) | 1.97 (1.36; 2.76) | 2.58 (1.76; 3.59) | 31 (30.42; 31.58) | 0.00 (0.00; 0.00) | 0.00 (0.00; 0.00) | 478.17 (475.51; 480.83) | 0.00 (0.00; 0.00) | 0.00 (0.00; 0.00) | 470.05 (467.55; 472.56) | 0.00 (0.00; 0.00) | 0.00 (0.00; 0.00) | 509.83 (506.37; 513.28) | 0.00 (0.00; 0.00) | 0.00 (0.00; 0.00) | 426.02 (423.94; 428.10) | 0.00 (0.00; 0.00) | 0.00 (0.00; 0.00) | 427.65 (425.60; 429.70) | 0.00 (0.00; 0.00) | 0.00 (0.00; 0.00) | 464.28 (462.43; 466.13) |
| **Gabon** | 0.01 (0.01; 0.01) | 0.01 (0.01; 0.01) | 23.58 (23.11; 24.05) | 0.00 (0.00; 0.00) | 0.00 (0.00; 0.00) | 15.84 (15.43; 16.26) | 1.07 (0.69; 1.55) | 1.35 (0.89; 1.97) | 24.74 (24.11; 25.38) | 56.71 (41.32; 75.07) | 60.17 (43.19; 79.88) | 7.11 (6.66; 7.56) | 57.74 (42.42; 75.09) | 61.52 (44.45; 81.18) | 7.45 (7.01; 7.89) | 2.26 (1.70; 2.90) | 2.53 (1.84; 3.27) | 12.23 (11.81; 12.65) | 0.00 (0.00; 0.00) | 0.00 (0.00; 0.00) | 178.60 (177.65; 179.55) | 0.00 (0.00; 0.00) | 0.00 (0.00; 0.00) | 174.34 (173.44; 175.23) | 0.00 (0.00; 0.00) | 0.00 (0.00; 0.00) | 189.89 (188.61; 191.16) | 0.00 (0.00; 0.00) | 0.00 (0.00; 0.00) | 144.13 (143.31; 144.96) | 0.00 (0.00; 0.00) | 0.00 (0.00; 0.00) | 144.97 (144.15; 145.78) | 0.00 (0.00; 0.00) | 0.00 (0.00; 0.00) | 171.26 (170.49; 172.03) |
| **Eastern Sub-Saharan Africa** | 0.01 (0.01; 0.01) | 0.01 (0.01; 0.01) | 10.36 (10.05; 10.66) | 0.00 (0.00; 0.00) | 0.00 (0.00; 0.00) | -2.31 (-2.57; -2.05) | 1.10 (0.79; 1.49) | 1.25 (0.88; 1.69) | 12.34 (11.85; 12.82) | 68.56 (54.90; 82.16) | 62.17 (49.78; 75.18) | -8.97 (-9.25; -8.70) | 69.68 (55.98; 84.13) | 63.42 (51.08; 76.46) | -8.63 (-8.90; -8.37) | 2.61 (2.15; 3.09) | 2.46 (2.01; 2.91) | -5.92 (-6.17; -5.68) | 0.02 (0.01; 0.02) | 0.06 (0.04; 0.07) | 217.74 (217.01; 218.47) | 0.01 (0.01; 0.01) | 0.02 (0.02; 0.02) | 199.24 (198.59; 199.88) | 0.00 (0.00; 0.00) | 0.01 (0.01; 0.01) | 236.62 (235.40; 237.85) | 0.14 (0.11; 0.17) | 0.39 (0.31; 0.47) | 168.84 (168.24; 169.44) | 0.14 (0.11; 0.17) | 0.40 (0.32; 0.48) | 169.87 (169.28; 170.46) | 0.00 (0.00; 0.01) | 0.01 (0.01; 0.02) | 195.79 (195.21; 196.37) |
| **Burundi** | 0.01 (0.00; 0.01) | 0.01 (0.01; 0.01) | 20.22 (19.65; 20.79) | 0.00 (0.00; 0.00) | 0.00 (0.00; 0.00) | -8.79 (-9.26; -8.32) | 0.97 (0.59; 1.48) | 1.19 (0.73; 1.80) | 22.78 (22.07; 23.50) | 65.86 (44.41; 91.37) | 51.92 (33.55; 73.61) | -20.67 (-21.14; -20.19) | 66.86 (44.46; 95.27) | 53.10 (34.51; 74.64) | -20.03 (-20.51; -19.56) | 2.48 (1.72; 3.39) | 2.05 (1.40; 2.87) | -17.03 (-17.48; -16.57) | 0.00 (0.00; 0.00) | 0.00 (0.00; 0.00) | 240.44 (239; 241.87) | 0.00 (0.00; 0.00) | 0.00 (0.00; 0.00) | 173.36 (172.21; 174.51) | 0.00 (0.00; 0.00) | 0.00 (0.00; 0.00) | 261.15 (259.30; 263) | 0.00 (0.00; 0.01) | 0.01 (0.01; 0.01) | 129.97 (128.99; 130.95) | 0.00 (0.00; 0.01) | 0.01 (0.01; 0.01) | 131.77 (130.78; 132.75) | 0.00 (0.00; 0.00) | 0.00 (0.00; 0.00) | 151.27 (150.29; 152.26) |
| **Comoros** | 0.01 (0.01; 0.01) | 0.01 (0.01; 0.01) | 8.70 (8.19; 9.20) | 0.00 (0.00; 0.00) | 0.00 (0.00; 0.00) | 0.41 (-0.05; 0.87) | 1.10 (0.69; 1.63) | 1.22 (0.75; 1.83) | 10.19 (9.55; 10.83) | 67.82 (48.63; 95.23) | 63.13 (44.02; 87.30) | -6.52 (-6.97; -6.07) | 68.93 (49.87; 95.69) | 64.35 (45.24; 88.35) | -6.25 (-6.69; -5.81) | 2.57 (1.90; 3.58) | 2.49 (1.79; 3.40) | -3.59 (-4.02; -3.15) | 0.00 (0.00; 0.00) | 0.00 (0.00; 0.00) | 109.47 (108.55; 110.39) | 0.00 (0.00; 0.00) | 0.00 (0.00; 0.00) | 113.35 (112.46; 114.25) | 0.00 (0.00; 0.00) | 0.00 (0.00; 0.00) | 127.15 (125.91; 128.39) | 0.00 (0.00; 0.00) | 0.00 (0.00; 0.00) | 85.03 (84.28; 85.79) | 0.00 (0.00; 0.00) | 0.00 (0.00; 0.00) | 85.69 (84.95; 86.43) | 0.00 (0.00; 0.00) | 0.00 (0.00; 0.00) | 113.78 (113.01; 114.56) |
| **Djibouti** | 0.01 (0.01; 0.01) | 0.01 (0.01; 0.01) | 12.80 (12.17; 13.43) | 0.00 (0.00; 0.01) | 0.00 (0.00; 0.01) | 0.60 (0.07; 1.14) | 1.22 (0.72; 1.88) | 1.40 (0.86; 2.11) | 13.95 (13.25; 14.64) | 74.10 (48.55; 106.69) | 66.60 (43.01; 96.62) | -9.02 (-9.56; -8.48) | 75.35 (48.95; 109.84) | 68.00 (44.69; 97.60) | -8.64 (-9.17; -8.11) | 2.84 (1.96; 3.93) | 2.67 (1.83; 3.71) | -5.54 (-6.02; -5.06) | 0.00 (0.00; 0.00) | 0.00 (0.00; 0.00) | 212.12 (210.65; 213.59) | 0.00 (0.00; 0.00) | 0.00 (0.00; 0.00) | 213.46 (212.05; 214.87) | 0.00 (0.00; 0.00) | 0.00 (0.00; 0.00) | 240.27 (238.54; 242.01) | 0.00 (0.00; 0.00) | 0.00 (0.00; 0.00) | 159.31 (158.29; 160.33) | 0.00 (0.00; 0.00) | 0.00 (0.00; 0.00) | 160.58 (159.57; 161.58) | 0.00 (0.00; 0.00) | 0.00 (0.00; 0.00) | 210.39 (209.37; 211.40) |
| **Eritrea** | 0.01 (0.01; 0.01) | 0.01 (0.01; 0.01) | 14.05 (13.56; 14.54) | 0.00 (0.00; 0.00) | 0.00 (0.00; 0.00) | 2.16 (1.74; 2.58) | 1.09 (0.67; 1.61) | 1.28 (0.79; 1.92) | 17.15 (16.47; 17.82) | 73.76 (52.80; 100.13) | 67.84 (48.56; 94.37) | -7.22 (-7.68; -6.76) | 74.78 (53.96; 101.13) | 69.12 (49.60; 95.80) | -6.86 (-7.32; -6.40) | 2.72 (1.99; 3.61) | 2.64 (1.92; 3.62) | -2.82 (-3.26; -2.38) | 0.00 (0.00; 0.00) | 0.00 (0.00; 0.00) | 163.52 (162.21; 164.82) | 0.00 (0.00; 0.00) | 0.00 (0.00; 0.00) | 159.95 (158.68; 161.23) | 0.00 (0.00; 0.00) | 0.00 (0.00; 0.00) | 190.70 (189.09; 192.32) | 0.00 (0.00; 0.00) | 0.01 (0.00; 0.01) | 119.95 (118.84; 121.05) | 0.00 (0.00; 0.00) | 0.01 (0.00; 0.01) | 120.92 (119.81; 122.03) | 0.00 (0.00; 0.00) | 0.00 (0.00; 0.00) | 156.66 (155.42; 157.89) |
| **Ethiopia** | 0.01 (0.00; 0.01) | 0.01 (0.01; 0.01) | 33.55 (33.22; 33.88) | 0.00 (0.00; 0.00) | 0.00 (0.00; 0.00) | 6.55 (6.25; 6.85) | 0.80 (0.53; 1.09) | 1.09 (0.74; 1.51) | 35.22 (34.62; 35.81) | 47.45 (38.72; 57.76) | 45.83 (36.79; 56.38) | -3.65 (-3.95; -3.36) | 48.23 (39.31; 58.04) | 46.92 (37.85; 57.50) | -3.00 (-3.29; -2.71) | 2.00 (1.63; 2.43) | 1.97 (1.58; 2.41) | -1.88 (-2.17; -1.59) | 0.00 (0.00; 0.00) | 0.01 (0.01; 0.02) | 325.54 (324.40; 326.68) | 0.00 (0.00; 0.00) | 0.00 (0.00; 0.01) | 264.83 (263.86; 265.80) | 0.00 (0.00; 0.00) | 0.00 (0.00; 0.00) | 352.96 (351.18; 354.74) | 0.02 (0.02; 0.03) | 0.08 (0.06; 0.10) | 214.82 (213.99; 215.66) | 0.02 (0.02; 0.03) | 0.08 (0.06; 0.10) | 217.02 (216.19; 217.85) | 0.00 (0.00; 0.00) | 0.00 (0.00; 0.00) | 242.67 (241.77; 243.57) |
| **Kenya** | 0.01 (0.01; 0.02) | 0.01 (0.01; 0.02) | -4.93 (-5.31; -4.54) | 0.00 (0.00; 0.01) | 0.00 (0.00; 0.01) | -8.99 (-9.34; -8.63) | 1.61 (1.09; 2.28) | 1.56 (1.04; 2.23) | -3.55 (-4.07; -3.03) | 94.46 (71.54; 120.49) | 86.94 (65.12; 114.78) | -7.25 (-7.62; -6.87) | 96.09 (72.46; 123.36) | 88.49 (66.64; 116.34) | -7.18 (-7.55; -6.82) | 3.57 (2.76; 4.48) | 3.43 (2.62; 4.50) | -3.69 (-4.05; -3.33) | 0.00 (0.00; 0.00) | 0.01 (0.01; 0.01) | 145.99 (145.17; 146.81) | 0.00 (0.00; 0.00) | 0.00 (0.00; 0.00) | 154.17 (153.37; 154.97) | 0.00 (0.00; 0.00) | 0.00 (0.00; 0.00) | 161.95 (160.87; 163.02) | 0.03 (0.02; 0.03) | 0.07 (0.05; 0.09) | 148.33 (147.57; 149.08) | 0.03 (0.02; 0.03) | 0.07 (0.05; 0.09) | 148.55 (147.80; 149.30) | 0.00 (0.00; 0.00) | 0.00 (0.00; 0.00) | 180.48 (179.72; 181.24) |
| **Madagascar** | 0.01 (0.00; 0.01) | 0.01 (0.00; 0.01) | -4.49 (-4.93; -4.05) | 0.00 (0.00; 0.00) | 0.00 (0.00; 0.00) | -7.23 (-7.65; -6.81) | 0.86 (0.55; 1.28) | 0.84 (0.54; 1.24) | -2.94 (-3.52; -2.35) | 55.28 (39.65; 74.52) | 48.07 (33.40; 64.49) | -12.64 (-13.05; -12.23) | 56.16 (40.14; 76.36) | 48.91 (34.41; 65.40) | -12.49 (-12.90; -12.09) | 2.03 (1.49; 2.68) | 1.85 (1.31; 2.44) | -8.93 (-9.33; -8.52) | 0.00 (0.00; 0.00) | 0.00 (0.00; 0.00) | 157.27 (156.32; 158.22) | 0.00 (0.00; 0.00) | 0.00 (0.00; 0.00) | 168.12 (167.14; 169.09) | 0.00 (0.00; 0.00) | 0.00 (0.00; 0.00) | 175.29 (173.99; 176.59) | 0.01 (0.01; 0.01) | 0.02 (0.01; 0.03) | 139.63 (138.82; 140.45) | 0.01 (0.01; 0.01) | 0.02 (0.01; 0.03) | 140.15 (139.35; 140.95) | 0.00 (0.00; 0.00) | 0.00 (0.00; 0.00) | 171.63 (170.83; 172.43) |
| **Malawi** | 0.01 (0.00; 0.01) | 0.01 (0.01; 0.01) | 20.54 (20.05; 21.03) | 0.00 (0.00; 0.00) | 0.00 (0.00; 0.00) | 8.71 (8.28; 9.13) | 0.93 (0.60; 1.37) | 1.15 (0.74; 1.68) | 22.04 (21.39; 22.69) | 57.86 (41.32; 74.76) | 58.76 (42.47; 77.51) | 1.74 (1.33; 2.15) | 58.78 (41.68; 75.60) | 59.91 (43.54; 78.80) | 2.07 (1.66; 2.47) | 2.10 (1.54; 2.68) | 2.22 (1.66; 2.80) | 4.87 (4.49; 5.25) | 0.00 (0.00; 0.00) | 0.00 (0.00; 0.00) | 242.45 (241.23; 243.68) | 0.00 (0.00; 0.00) | 0.00 (0.00; 0.00) | 223.44 (222.35; 224.53) | 0.00 (0.00; 0.00) | 0.00 (0.00; 0.00) | 257.48 (255.83; 259.13) | 0.01 (0.00; 0.01) | 0.02 (0.01; 0.02) | 196.79 (195.86; 197.72) | 0.01 (0.00; 0.01) | 0.02 (0.01; 0.02) | 197.71 (196.78; 198.63) | 0.00 (0.00; 0.00) | 0.00 (0.00; 0.00) | 216.55 (215.66; 217.44) |
| **Mozambique** | 0.00 (0.00; 0.01) | 0.01 (0.00; 0.01) | 30.99 (30.51; 31.47) | 0.00 (0.00; 0.00) | 0.00 (0.00; 0.00) | 17.69 (17.27; 18.12) | 0.69 (0.46; 1.02) | 0.92 (0.59; 1.35) | 31.32 (30.64; 32) | 45.66 (33.57; 59.68) | 48.91 (35.97; 64.16) | 7.24 (6.82; 7.65) | 46.34 (34.14; 60.82) | 49.82 (36.87; 65.13) | 7.60 (7.19; 8.01) | 1.78 (1.32; 2.27) | 1.94 (1.45; 2.49) | 8.84 (8.45; 9.23) | 0.00 (0.00; 0.00) | 0.00 (0.00; 0.00) | 282.08 (280.84; 283.32) | 0.00 (0.00; 0.00) | 0.00 (0.00; 0.00) | 253.55 (252.43; 254.67) | 0.00 (0.00; 0.00) | 0.00 (0.00; 0.00) | 290.30 (288.58; 292.03) | 0.01 (0.00; 0.01) | 0.02 (0.01; 0.03) | 214.48 (213.53; 215.43) | 0.01 (0.00; 0.01) | 0.02 (0.01; 0.03) | 215.55 (214.61; 216.48) | 0.00 (0.00; 0.00) | 0.00 (0.00; 0.00) | 228.43 (227.56; 229.30) |
| **Rwanda** | 0.01 (0.01; 0.01) | 0.01 (0.01; 0.02) | 24.06 (23.50; 24.62) | 0.00 (0.00; 0.00) | 0.00 (0.00; 0.00) | -4.98 (-5.44; -4.53) | 1.19 (0.76; 1.82) | 1.54 (0.97; 2.26) | 27.36 (26.67; 28.05) | 73.83 (50.29; 103.79) | 60.30 (40.20; 87.47) | -18.10 (-18.58; -17.61) | 74.98 (52.13; 104.08) | 61.84 (41.91; 88.81) | -17.36 (-17.84; -16.89) | 2.79 (1.96; 3.86) | 2.41 (1.70; 3.36) | -14.20 (-14.64; -13.75) | 0.00 (0.00; 0.00) | 0.00 (0.00; 0.00) | 262.05 (260.61; 263.48) | 0.00 (0.00; 0.00) | 0.00 (0.00; 0.00) | 196.02 (194.88; 197.17) | 0.00 (0.00; 0.00) | 0.00 (0.00; 0.00) | 287.63 (285.76; 289.51) | 0.01 (0.00; 0.01) | 0.01 (0.01; 0.02) | 144.98 (143.94; 146.01) | 0.01 (0.00; 0.01) | 0.01 (0.01; 0.02) | 147.19 (146.16; 148.21) | 0.00 (0.00; 0.00) | 0.00 (0.00; 0.00) | 173.93 (172.91; 174.95) |
| **Somalia** | 0.00 (0.00; 0.01) | 0.01 (0.00; 0.01) | 12.44 (11.88; 13) | 0.00 (0.00; 0.00) | 0.00 (0.00; 0.00) | 4.28 (3.78; 4.79) | 0.80 (0.51; 1.25) | 0.92 (0.58; 1.46) | 14.74 (14.02; 15.45) | 59.13 (39.76; 85.71) | 59.90 (38.87; 93.53) | 1.73 (1.12; 2.34) | 59.88 (41.15; 84.40) | 60.82 (39.64; 94.27) | 1.90 (1.30; 2.50) | 2.17 (1.46; 3.02) | 2.25 (1.52; 3.39) | 3.35 (2.79; 3.92) | 0.00 (0.00; 0.00) | 0.00 (0.00; 0.00) | 203.15 (201.57; 204.72) | 0.00 (0.00; 0.00) | 0.00 (0.00; 0.00) | 193.47 (192; 194.93) | 0.00 (0.00; 0.00) | 0.00 (0.00; 0.00) | 218.36 (216.52; 220.21) | 0.00 (0.00; 0.01) | 0.01 (0.01; 0.02) | 180.24 (178.81; 181.66) | 0.00 (0.00; 0.01) | 0.01 (0.01; 0.02) | 180.71 (179.30; 182.12) | 0.00 (0.00; 0.00) | 0.00 (0.00; 0.00) | 197.35 (195.94; 198.75) |
| **South Sudan** | 0.01 (0.00; 0.01) | 0.01 (0.00; 0.01) | 7.07 (6.55; 7.58) | 0.00 (0.00; 0.00) | 0.00 (0.00; 0.00) | -4.20 (-4.66; -3.74) | 0.96 (0.61; 1.43) | 1.03 (0.64; 1.56) | 7.35 (6.71; 7.99) | 61.98 (43.74; 84.93) | 54.76 (38.55; 78.49) | -10.78 (-11.27; -10.29) | 62.97 (44.04; 85.52) | 55.79 (39.55; 79.38) | -10.50 (-10.98; -10.02) | 2.36 (1.71; 3.20) | 2.14 (1.53; 3.00) | -8.92 (-9.39; -8.46) | 0.00 (0.00; 0.00) | 0.00 (0.00; 0.00) | 169.60 (168.55; 170.64) | 0.00 (0.00; 0.00) | 0.00 (0.00; 0.00) | 153.18 (152.19; 154.17) | 0.00 (0.00; 0.00) | 0.00 (0.00; 0.00) | 178.40 (177.04; 179.76) | 0.00 (0.00; 0.00) | 0.01 (0.00; 0.01) | 125.94 (125.07; 126.80) | 0.00 (0.00; 0.00) | 0.01 (0.00; 0.01) | 126.69 (125.83; 127.55) | 0.00 (0.00; 0.00) | 0.00 (0.00; 0.00) | 143.61 (142.76; 144.46) |
| **Uganda** | 0.01 (0.01; 0.01) | 0.01 (0.01; 0.01) | -0.27 (-0.71; 0.16) | 0.00 (0.00; 0.01) | 0.00 (0.00; 0.01) | -9.64 (-10.03; -9.25) | 1.51 (0.97; 2.20) | 1.53 (0.98; 2.23) | 0.85 (0.28; 1.43) | 96.07 (67.99; 125.25) | 79.83 (57.13; 106.76) | -16.64 (-17.03; -16.25) | 97.63 (68.16; 128.45) | 81.36 (58.66; 108.30) | -16.36 (-16.75; -15.98) | 3.56 (2.65; 4.56) | 3.12 (2.34; 4.08) | -12.55 (-12.92; -12.19) | 0.00 (0.00; 0.00) | 0.01 (0.00; 0.01) | 222.24 (221.02; 223.46) | 0.00 (0.00; 0.00) | 0.00 (0.00; 0.00) | 203.94 (202.88; 204.99) | 0.00 (0.00; 0.00) | 0.00 (0.00; 0.00) | 234.45 (232.80; 236.10) | 0.02 (0.01; 0.02) | 0.05 (0.03; 0.07) | 173.31 (172.35; 174.27) | 0.02 (0.01; 0.02) | 0.05 (0.04; 0.07) | 174.22 (173.28; 175.16) | 0.00 (0.00; 0.00) | 0.00 (0.00; 0.00) | 197.66 (196.74; 198.57) |
| **United Republic of Tanzania** | 0.01 (0.01; 0.01) | 0.01 (0.01; 0.01) | 1.68 (1.17; 2.19) | 0.00 (0.00; 0.00) | 0.00 (0.00; 0.00) | -7.35 (-7.81; -6.88) | 1.11 (0.68; 1.67) | 1.15 (0.72; 1.68) | 2.41 (1.79; 3.03) | 67.76 (45.34; 94.21) | 58.27 (38.80; 81.87) | -13.75 (-14.23; -13.26) | 68.85 (45.76; 95.69) | 59.42 (39.86; 82.59) | -13.48 (-13.96; -13.01) | 2.54 (1.75; 3.50) | 2.27 (1.58; 3.09) | -10.95 (-11.41; -10.50) | 0.00 (0.00; 0.00) | 0.01 (0.00; 0.01) | 173.08 (171.96; 174.20) | 0.00 (0.00; 0.00) | 0.00 (0.00; 0.00) | 162.93 (161.89; 163.97) | 0.00 (0.00; 0.00) | 0.00 (0.00; 0.00) | 185.23 (183.94; 186.52) | 0.02 (0.01; 0.03) | 0.05 (0.03; 0.07) | 135 (134.04; 135.97) | 0.02 (0.01; 0.03) | 0.05 (0.03; 0.07) | 135.79 (134.83; 136.74) | 0.00 (0.00; 0.00) | 0.00 (0.00; 0.00) | 158.30 (157.34; 159.26) |
| **Zambia** | 0.01 (0.01; 0.02) | 0.02 (0.01; 0.03) | 17.81 (16.92; 18.70) | 0.01 (0.00; 0.01) | 0.01 (0.00; 0.01) | 5.40 (4.63; 6.17) | 1.91 (0.82; 3.27) | 2.29 (1.04; 3.96) | 18.90 (17.91; 19.89) | 126.26 (57.03; 200.93) | 121.73 (55.27; 197.70) | -2.77 (-3.59; -1.95) | 128.59 (57.07; 203.23) | 124.02 (58.06; 200.23) | -2.44 (-3.25; -1.64) | 4.39 (2.18; 6.73) | 4.39 (2.22; 6.85) | 0.28 (-0.46; 1.03) | 0.00 (0.00; 0.00) | 0.01 (0.00; 0.01) | 260.48 (258.12; 262.84) | 0.00 (0.00; 0.00) | 0.00 (0.00; 0.00) | 241.28 (239.11; 243.45) | 0.00 (0.00; 0.00) | 0.00 (0.00; 0.00) | 276.54 (273.89; 279.19) | 0.01 (0.00; 0.02) | 0.04 (0.02; 0.06) | 204.03 (202.14; 205.92) | 0.01 (0.00; 0.02) | 0.04 (0.02; 0.06) | 205.08 (203.22; 206.95) | 0.00 (0.00; 0.00) | 0.00 (0.00; 0.00) | 230.85 (228.96; 232.74) |
| **Southern Sub-Saharan Africa** | 0.01 (0.01; 0.01) | 0.01 (0.01; 0.01) | 3.69 (3.51; 3.88) | 0.00 (0.00; 0.00) | 0.00 (0.00; 0.00) | 4.29 (4.11; 4.46) | 1.38 (0.99; 1.83) | 1.46 (1.04; 1.92) | 5.51 (5.07; 5.95) | 74.98 (65.88; 83.41) | 75.23 (64.14; 85.43) | 0.88 (0.69; 1.07) | 76.37 (67.34; 85.36) | 76.69 (65.57; 87.04) | 0.96 (0.78; 1.15) | 2.72 (2.42; 3.01) | 2.85 (2.48; 3.22) | 4.58 (4.41; 4.76) | 0.01 (0.01; 0.01) | 0.01 (0.01; 0.02) | 90.61 (90.25; 90.96) | 0.00 (0.00; 0.00) | 0.01 (0.00; 0.01) | 105.46 (105.11; 105.82) | 0.00 (0.00; 0.00) | 0.00 (0.00; 0.00) | 102.76 (102.04; 103.48) | 0.05 (0.04; 0.05) | 0.09 (0.08; 0.11) | 89.36 (89.02; 89.69) | 0.05 (0.04; 0.06) | 0.09 (0.08; 0.11) | 89.59 (89.26; 89.93) | 0.00 (0.00; 0.00) | 0.00 (0.00; 0.00) | 114.62 (114.27; 114.97) |
| **Botswana** | 0.01 (0.01; 0.01) | 0.01 (0.01; 0.01) | 10.91 (10.36; 11.46) | 0.00 (0.00; 0.01) | 0.00 (0.00; 0.01) | 6.33 (5.84; 6.82) | 1.29 (0.82; 1.93) | 1.46 (0.93; 2.22) | 13.33 (12.68; 13.98) | 75.41 (49.25; 106.55) | 73.73 (47.85; 106.44) | -1.96 (-2.50; -1.42) | 76.77 (50.74; 109.18) | 75.20 (49.40; 108.03) | -1.70 (-2.23; -1.17) | 2.80 (1.96; 3.83) | 2.89 (2.03; 3.99) | 2.77 (2.29; 3.25) | 0.00 (0.00; 0.00) | 0.00 (0.00; 0.00) | 150.11 (149.07; 151.16) | 0.00 (0.00; 0.00) | 0.00 (0.00; 0.00) | 166.74 (165.68; 167.80) | 0.00 (0.00; 0.00) | 0.00 (0.00; 0.00) | 173.93 (172.55; 175.31) | 0.00 (0.00; 0.00) | 0.00 (0.00; 0.00) | 129.41 (128.40; 130.42) | 0.00 (0.00; 0.00) | 0.00 (0.00; 0.00) | 130.13 (129.14; 131.13) | 0.00 (0.00; 0.00) | 0.00 (0.00; 0.00) | 171.83 (170.80; 172.85) |
| **Eswatini** | 0.01 (0.01; 0.02) | 0.01 (0.01; 0.01) | -3.92 (-4.54; -3.31) | 0.00 (0.00; 0.01) | 0.00 (0.00; 0.01) | -0.96 (-1.55; -0.37) | 1.39 (0.79; 2.27) | 1.36 (0.77; 2.21) | -2.29 (-3.03; -1.55) | 89.42 (57.70; 139.33) | 84.05 (54.08; 132.98) | -4.75 (-5.39; -4.11) | 90.71 (58.98; 137.28) | 85.41 (55.48; 134.05) | -4.71 (-5.35; -4.08) | 3.18 (2.11; 4.79) | 3.13 (2.09; 4.79) | -0.63 (-1.24; -0.02) | 0.00 (0.00; 0.00) | 0.00 (0.00; 0.00) | 90.24 (89.26; 91.22) | 0.00 (0.00; 0.00) | 0.00 (0.00; 0.00) | 108.22 (107.18; 109.27) | 0.00 (0.00; 0.00) | 0.00 (0.00; 0.00) | 100.74 (99.49; 101.99) | 0.00 (0.00; 0.00) | 0.00 (0.00; 0.00) | 93.56 (92.62; 94.50) | 0.00 (0.00; 0.00) | 0.00 (0.00; 0.00) | 93.67 (92.74; 94.59) | 0.00 (0.00; 0.00) | 0.00 (0.00; 0.00) | 113.41 (112.48; 114.34) |
| **Lesotho** | 0.01 (0.01; 0.01) | 0.01 (0.01; 0.01) | 1.52 (0.90; 2.13) | 0.00 (0.00; 0.01) | 0.00 (0.00; 0.01) | 7.50 (6.90; 8.11) | 1.38 (0.79; 2.17) | 1.42 (0.81; 2.28) | 3.14 (2.42; 3.87) | 97.68 (64.28; 149.39) | 104.14 (68.21; 163.75) | 6.31 (5.65; 6.97) | 98.99 (63.09; 149.68) | 105.56 (69.38; 164.94) | 6.27 (5.62; 6.92) | 3.46 (2.35; 5.21) | 3.83 (2.60; 5.80) | 9.74 (9.12; 10.36) | 0.00 (0.00; 0.00) | 0.00 (0.00; 0.00) | 90.10 (89.14; 91.06) | 0.00 (0.00; 0.00) | 0.00 (0.00; 0.00) | 100.06 (99.05; 101.08) | 0.00 (0.00; 0.00) | 0.00 (0.00; 0.00) | 92.80 (91.62; 93.99) | 0.00 (0.00; 0.00) | 0.00 (0.00; 0.00) | 101.58 (100.56; 102.60) | 0.00 (0.00; 0.00) | 0.00 (0.00; 0.00) | 101.46 (100.46; 102.47) | 0.00 (0.00; 0.00) | 0.00 (0.00; 0.00) | 102.78 (101.83; 103.73) |
| **Namibia** | 0.02 (0.01; 0.03) | 0.03 (0.02; 0.04) | 35.98 (35.42; 36.54) | 0.01 (0.01; 0.01) | 0.01 (0.01; 0.01) | 22.13 (21.65; 22.60) | 2.64 (1.66; 3.98) | 3.63 (2.34; 5.60) | 36.44 (35.68; 37.19) | 152.90 (108.43; 206.28) | 175.59 (123.30; 236.86) | 15.39 (14.90; 15.88) | 155.57 (110.30; 204.00) | 179.23 (127.41; 240.79) | 15.75 (15.28; 16.23) | 5.42 (4.02; 7.07) | 6.36 (4.69; 8.46) | 17.30 (16.85; 17.75) | 0.00 (0.00; 0.00) | 0.00 (0.00; 0.00) | 196.60 (195.38; 197.82) | 0.00 (0.00; 0.00) | 0.00 (0.00; 0.00) | 184.34 (183.23; 185.45) | 0.00 (0.00; 0.00) | 0.00 (0.00; 0.00) | 209.26 (207.75; 210.76) | 0.00 (0.00; 0.00) | 0.01 (0.00; 0.01) | 158.28 (157.36; 159.19) | 0.00 (0.00; 0.00) | 0.01 (0.00; 0.01) | 159.13 (158.23; 160.04) | 0.00 (0.00; 0.00) | 0.00 (0.00; 0.00) | 182.67 (181.83; 183.52) |
| **South Africa** | 0.01 (0.01; 0.01) | 0.01 (0.01; 0.01) | 3.30 (3.10; 3.49) | 0.00 (0.00; 0.00) | 0.00 (0.00; 0.00) | 3.81 (3.63; 4.00) | 1.38 (1.00; 1.82) | 1.45 (1.02; 1.94) | 5.26 (4.82; 5.71) | 71.91 (62.72; 80.62) | 71.31 (59.66; 81.40) | -0.26 (-0.47; -0.06) | 73.30 (63.97; 82.16) | 72.76 (60.87; 82.95) | -0.16 (-0.36; 0.04) | 2.63 (2.32; 2.93) | 2.73 (2.33; 3.09) | 3.77 (3.59; 3.96) | 0.01 (0.00; 0.01) | 0.01 (0.01; 0.01) | 83.83 (83.43; 84.23) | 0.00 (0.00; 0.00) | 0.00 (0.00; 0.00) | 99.47 (99.06; 99.88) | 0.00 (0.00; 0.00) | 0.00 (0.00; 0.00) | 96.66 (95.92; 97.40) | 0.04 (0.03; 0.04) | 0.07 (0.05; 0.08) | 81.89 (81.50; 82.27) | 0.04 (0.03; 0.04) | 0.07 (0.06; 0.08) | 82.16 (81.78; 82.55) | 0.00 (0.00; 0.00) | 0.00 (0.00; 0.00) | 109.09 (108.68; 109.51) |
| **Zimbabwe** | 0.01 (0.01; 0.01) | 0.01 (0.01; 0.01) | -13.25 (-13.64; -12.86) | 0.00 (0.00; 0.00) | 0.00 (0.00; 0.00) | -4.72 (-5.09; -4.35) | 1.12 (0.73; 1.63) | 0.99 (0.64; 1.47) | -11.60 (-12.15; -11.06) | 72.43 (54.42; 94.76) | 68.24 (51.92; 91.40) | -5.30 (-5.69; -4.91) | 73.52 (56.32; 96.53) | 69.23 (52.70; 92.57) | -5.40 (-5.78; -5.01) | 2.57 (2.00; 3.26) | 2.56 (1.99; 3.30) | -0.60 (-0.95; -0.24) | 0.00 (0.00; 0.00) | 0.00 (0.00; 0.00) | 75.26 (74.59; 75.93) | 0.00 (0.00; 0.00) | 0.00 (0.00; 0.00) | 99.22 (98.51; 99.92) | 0.00 (0.00; 0.00) | 0.00 (0.00; 0.00) | 83.69 (82.77; 84.62) | 0.01 (0.00; 0.01) | 0.01 (0.01; 0.02) | 93.88 (93.24; 94.52) | 0.01 (0.00; 0.01) | 0.01 (0.01; 0.02) | 93.73 (93.11; 94.36) | 0.00 (0.00; 0.00) | 0.00 (0.00; 0.00) | 111.70 (111.10; 112.30) |
| **Western Sub-Saharan Africa** | 0.00 (0.00; 0.00) | 0.00 (0.00; 0.00) | 14.72 (14.37; 15.06) | 0.00 (0.00; 0.00) | 0.00 (0.00; 0.00) | 4.98 (4.69; 5.27) | 0.43 (0.30; 0.60) | 0.51 (0.36; 0.72) | 16.63 (16.09; 17.16) | 25.50 (20.18; 31.37) | 24.29 (18.94; 29.80) | -4.37 (-4.67; -4.07) | 25.93 (20.51; 31.40) | 24.80 (19.38; 30.32) | -4.01 (-4.31; -3.71) | 0.99 (0.81; 1.19) | 0.99 (0.80; 1.19) | -0.27 (-0.53; 0) | 0.01 (0.01; 0.01) | 0.03 (0.02; 0.03) | 223.17 (222.36; 223.98) | 0.00 (0.00; 0.00) | 0.01 (0.01; 0.01) | 212.22 (211.50; 212.93) | 0.00 (0.00; 0.00) | 0.00 (0.00; 0.00) | 241.11 (239.80; 242.43) | 0.06 (0.05; 0.07) | 0.17 (0.13; 0.21) | 173.76 (173.12; 174.40) | 0.06 (0.05; 0.07) | 0.17 (0.13; 0.21) | 174.85 (174.21; 175.48) | 0.00 (0.00; 0.00) | 0.01 (0.00; 0.01) | 204.67 (204.09; 205.24) |
| **Benin** | 0.00 (0.00; 0.00) | 0.00 (0.00; 0.01) | 16.84 (16.27; 17.40) | 0.00 (0.00; 0.00) | 0.00 (0.00; 0.00) | 5.14 (4.65; 5.64) | 0.44 (0.28; 0.66) | 0.53 (0.34; 0.80) | 18.65 (17.97; 19.32) | 26.14 (17.95; 36.06) | 24.88 (16.61; 34.74) | -5.51 (-6; -5.01) | 26.58 (18.33; 36.89) | 25.42 (17.17; 35.38) | -5.10 (-5.59; -4.62) | 1.04 (0.75; 1.39) | 1.05 (0.73; 1.43) | -1.28 (-1.74; -0.82) | 0.00 (0.00; 0.00) | 0.00 (0.00; 0.00) | 262.42 (260.89; 263.96) | 0.00 (0.00; 0.00) | 0.00 (0.00; 0.00) | 234.24 (232.86; 235.61) | 0.00 (0.00; 0.00) | 0.00 (0.00; 0.00) | 274.47 (272.62; 276.32) | 0.00 (0.00; 0.00) | 0.00 (0.00; 0.01) | 193.96 (192.78; 195.15) | 0.00 (0.00; 0.00) | 0.00 (0.00; 0.01) | 195.24 (194.08; 196.41) | 0.00 (0.00; 0.00) | 0.00 (0.00; 0.00) | 215.60 (214.45; 216.74) |
| **Burkina Faso** | 0.00 (0.00; 0.00) | 0.00 (0.00; 0.01) | 29.45 (28.91; 29.99) | 0.00 (0.00; 0.00) | 0.00 (0.00; 0.00) | 13.64 (13.16; 14.12) | 0.47 (0.31; 0.70) | 0.64 (0.41; 0.98) | 32.85 (32.13; 33.57) | 28.66 (19.98; 38.45) | 29.18 (20.00; 39.61) | 0.92 (0.46; 1.39) | 29.14 (20.84; 39.50) | 29.82 (20.65; 40.27) | 1.44 (0.99; 1.90) | 1.14 (0.82; 1.51) | 1.24 (0.88; 1.66) | 6.44 (5.99; 6.89) | 0.00 (0.00; 0.00) | 0.00 (0.00; 0.00) | 263.24 (261.90; 264.59) | 0.00 (0.00; 0.00) | 0.00 (0.00; 0.00) | 221.28 (220.10; 222.46) | 0.00 (0.00; 0.00) | 0.00 (0.00; 0.00) | 275.45 (273.69; 277.20) | 0.00 (0.00; 0.00) | 0.01 (0.01; 0.01) | 182.35 (181.37; 183.34) | 0.00 (0.00; 0.00) | 0.01 (0.01; 0.01) | 183.80 (182.82; 184.78) | 0.00 (0.00; 0.00) | 0.00 (0.00; 0.00) | 199.19 (198.26; 200.12) |
| **Cabo Verde** | 0.02 (0.01; 0.02) | 0.02 (0.01; 0.02) | 10.17 (9.71; 10.63) | 0.01 (0.00; 0.01) | 0.01 (0.00; 0.01) | 3.09 (2.66; 3.52) | 1.80 (1.15; 2.64) | 2.04 (1.29; 3.06) | 12.66 (12.02; 13.29) | 79.54 (58.02; 107.68) | 72.72 (54.69; 99.48) | -8.69 (-9.10; -8.29) | 81.38 (59.49; 110.82) | 74.76 (56.48; 101.66) | -8.22 (-8.62; -7.82) | 3.16 (2.38; 4.17) | 3.17 (2.37; 4.32) | -0.95 (-1.36; -0.54) | 0.00 (0.00; 0.00) | 0.00 (0.00; 0.00) | 126.74 (125.87; 127.60) | 0.00 (0.00; 0.00) | 0.00 (0.00; 0.00) | 125.04 (124.18; 125.90) | 0.00 (0.00; 0.00) | 0.00 (0.00; 0.00) | 140.84 (139.69; 141.98) | 0.00 (0.00; 0.00) | 0.00 (0.00; 0.00) | 88.95 (88.28; 89.61) | 0.00 (0.00; 0.00) | 0.00 (0.00; 0.00) | 90.08 (89.42; 90.73) | 0.00 (0.00; 0.00) | 0.00 (0.00; 0.00) | 123.08 (122.40; 123.75) |
| **Cameroon** | 0.00 (0.00; 0.01) | 0.00 (0.00; 0.01) | 18.03 (17.50; 18.56) | 0.00 (0.00; 0.00) | 0.00 (0.00; 0.00) | 8.46 (7.99; 8.93) | 0.57 (0.35; 0.87) | 0.69 (0.44; 1.06) | 20.33 (19.62; 21.04) | 32.71 (23.20; 45.67) | 31.70 (21.91; 45.41) | -2.67 (-3.18; -2.17) | 33.26 (23.94; 46.23) | 32.39 (22.66; 46.15) | -2.28 (-2.77; -1.78) | 1.28 (0.95; 1.74) | 1.32 (0.96; 1.82) | 2.83 (2.37; 3.28) | 0.00 (0.00; 0.00) | 0.00 (0.00; 0.00) | 240.87 (239.41; 242.32) | 0.00 (0.00; 0.00) | 0.00 (0.00; 0.00) | 226.91 (225.56; 228.27) | 0.00 (0.00; 0.00) | 0.00 (0.00; 0.00) | 257.50 (255.72; 259.27) | 0.00 (0.00; 0.01) | 0.01 (0.01; 0.02) | 183.68 (182.60; 184.76) | 0.00 (0.00; 0.01) | 0.01 (0.01; 0.02) | 184.88 (183.82; 185.95) | 0.00 (0.00; 0.00) | 0.00 (0.00; 0.00) | 214.01 (212.96; 215.06) |
| **Chad** | 0.00 (0.00; 0.00) | 0.00 (0.00; 0.01) | 34.20 (33.68; 34.73) | 0.00 (0.00; 0.00) | 0.00 (0.00; 0.00) | 23.69 (23.22; 24.16) | 0.43 (0.27; 0.62) | 0.59 (0.38; 0.85) | 35.81 (35.13; 36.48) | 28.35 (20.52; 38.04) | 32.80 (23.44; 45.06) | 15.26 (14.77; 15.76) | 28.77 (21.10; 38.56) | 33.38 (23.89; 45.52) | 15.57 (15.08; 16.06) | 1.09 (0.81; 1.44) | 1.30 (0.94; 1.76) | 17.63 (17.15; 18.10) | 0.00 (0.00; 0.00) | 0.00 (0.00; 0.00) | 333.05 (331.46; 334.63) | 0.00 (0.00; 0.00) | 0.00 (0.00; 0.00) | 298.30 (296.87; 299.73) | 0.00 (0.00; 0.00) | 0.00 (0.00; 0.00) | 338.18 (336.25; 340.11) | 0.00 (0.00; 0.00) | 0.01 (0.01; 0.01) | 270.87 (269.66; 272.08) | 0.00 (0.00; 0.00) | 0.01 (0.01; 0.01) | 271.82 (270.62; 273.02) | 0.00 (0.00; 0.00) | 0.00 (0.00; 0.00) | 276.09 (274.99; 277.18) |
| **Côte d'Ivoire** | 0.01 (0.00; 0.01) | 0.01 (0.00; 0.01) | 9.45 (8.91; 9.98) | 0.00 (0.00; 0.00) | 0.00 (0.00; 0.00) | -0.11 (-0.59; 0.38) | 0.77 (0.48; 1.18) | 0.86 (0.55; 1.35) | 10.41 (9.72; 11.09) | 45.52 (30.06; 63.63) | 41.17 (27.84; 58.49) | -8.50 (-9; -8) | 46.35 (30.98; 65.60) | 42.03 (28.65; 59.45) | -8.18 (-8.67; -7.68) | 1.69 (1.19; 2.30) | 1.61 (1.15; 2.24) | -4.34 (-4.80; -3.88) | 0.00 (0.00; 0.00) | 0.00 (0.00; 0.00) | 191.81 (190.54; 193.08) | 0.00 (0.00; 0.00) | 0.00 (0.00; 0.00) | 192.31 (191.12; 193.51) | 0.00 (0.00; 0.00) | 0.00 (0.00; 0.00) | 213.84 (212.21; 215.47) | 0.01 (0.00; 0.01) | 0.02 (0.01; 0.02) | 150.78 (149.73; 151.83) | 0.01 (0.00; 0.01) | 0.02 (0.01; 0.02) | 151.79 (150.76; 152.83) | 0.00 (0.00; 0.00) | 0.00 (0.00; 0.00) | 193.58 (192.56; 194.59) |
| **Gambia** | 0.00 (0.00; 0.01) | 0.00 (0.00; 0.01) | 7.45 (6.96; 7.93) | 0.00 (0.00; 0.00) | 0.00 (0.00; 0.00) | 2.43 (1.97; 2.89) | 0.58 (0.37; 0.85) | 0.63 (0.39; 0.96) | 8.22 (7.59; 8.86) | 32.67 (23.04; 42.69) | 31.19 (21.76; 41.18) | -4.38 (-4.80; -3.96) | 33.28 (23.04; 44.33) | 31.82 (22.42; 41.91) | -4.15 (-4.57; -3.74) | 1.23 (0.88; 1.60) | 1.23 (0.88; 1.61) | -0.62 (-1.02; -0.22) | 0.00 (0.00; 0.00) | 0.00 (0.00; 0.00) | 177.83 (176.84; 178.82) | 0.00 (0.00; 0.00) | 0.00 (0.00; 0.00) | 177.91 (176.96; 178.87) | 0.00 (0.00; 0.00) | 0.00 (0.00; 0.00) | 189.90 (188.43; 191.37) | 0.00 (0.00; 0.00) | 0.00 (0.00; 0.00) | 153.09 (152.17; 154.01) | 0.00 (0.00; 0.00) | 0.00 (0.00; 0.00) | 153.71 (152.82; 154.61) | 0.00 (0.00; 0.00) | 0.00 (0.00; 0.00) | 173.59 (172.71; 174.48) |
| **Ghana** | 0.00 (0.00; 0.00) | 0.00 (0.00; 0.00) | 19.52 (19.08; 19.96) | 0.00 (0.00; 0.00) | 0.00 (0.00; 0.00) | 16.47 (16.06; 16.88) | 0.17 (0.12; 0.25) | 0.22 (0.14; 0.32) | 24.42 (23.77; 25.06) | 8.71 (6.44; 11.28) | 9.45 (6.93; 12.39) | 7.34 (6.93; 7.74) | 8.88 (6.75; 11.64) | 9.67 (7.10; 12.63) | 7.67 (7.27; 8.07) | 0.35 (0.27; 0.45) | 0.41 (0.31; 0.54) | 14.96 (14.54; 15.38) | 0.00 (0.00; 0.00) | 0.00 (0.00; 0.00) | 191.62 (190.58; 192.66) | 0.00 (0.00; 0.00) | 0.00 (0.00; 0.00) | 205.04 (204.03; 206.05) | 0.00 (0.00; 0.00) | 0.00 (0.00; 0.00) | 218.82 (217.28; 220.37) | 0.00 (0.00; 0.00) | 0.00 (0.00; 0.01) | 170.39 (169.55; 171.23) | 0.00 (0.00; 0.00) | 0.00 (0.00; 0.01) | 171.31 (170.48; 172.14) | 0.00 (0.00; 0.00) | 0.00 (0.00; 0.00) | 213.53 (212.71; 214.36) |
| **Guinea** | 0.01 (0.01; 0.01) | 0.01 (0.01; 0.01) | 7.92 (7.48; 8.37) | 0.00 (0.00; 0.01) | 0.00 (0.00; 0.01) | -0.24 (-0.64; 0.16) | 1.40 (0.91; 2.05) | 1.53 (1.00; 2.22) | 8.45 (7.86; 9.05) | 93.60 (68.38; 123.17) | 85.11 (60.81; 115.96) | -8.29 (-8.70; -7.87) | 95.02 (69.55; 125.64) | 86.64 (62.79; 117.70) | -8.04 (-8.44; -7.63) | 3.43 (2.55; 4.43) | 3.24 (2.40; 4.31) | -5.25 (-5.64; -4.86) | 0.00 (0.00; 0.00) | 0.00 (0.00; 0.00) | 191.75 (190.63; 192.86) | 0.00 (0.00; 0.00) | 0.00 (0.00; 0.00) | 174.63 (173.61; 175.66) | 0.00 (0.00; 0.00) | 0.00 (0.00; 0.00) | 197.16 (195.81; 198.51) | 0.01 (0.00; 0.01) | 0.02 (0.01; 0.02) | 149.78 (148.98; 150.58) | 0.01 (0.00; 0.01) | 0.02 (0.01; 0.02) | 150.46 (149.67; 151.26) | 0.00 (0.00; 0.00) | 0.00 (0.00; 0.00) | 160.94 (160.20; 161.69) |
| **Guinea-Bissau** | 0.00 (0.00; 0.01) | 0.00 (0.00; 0.01) | 20.73 (20.23; 21.24) | 0.00 (0.00; 0.00) | 0.00 (0.00; 0.00) | 8.63 (8.17; 9.08) | 0.57 (0.36; 0.84) | 0.71 (0.44; 1.06) | 23.59 (22.90; 24.28) | 37.55 (27.54; 51.46) | 37.10 (27.30; 50.54) | -2.30 (-2.75; -1.86) | 38.10 (28.32; 51.53) | 37.81 (27.97; 51.20) | -1.92 (-2.35; -1.48) | 1.44 (1.08; 1.92) | 1.50 (1.14; 1.99) | 2.29 (1.89; 2.70) | 0.00 (0.00; 0.00) | 0.00 (0.00; 0.00) | 252.79 (251.46; 254.11) | 0.00 (0.00; 0.00) | 0.00 (0.00; 0.00) | 231.79 (230.56; 233.03) | 0.00 (0.00; 0.00) | 0.00 (0.00; 0.00) | 271.86 (270.04; 273.69) | 0.00 (0.00; 0.00) | 0.00 (0.00; 0.00) | 191.03 (189.97; 192.09) | 0.00 (0.00; 0.00) | 0.00 (0.00; 0.00) | 192.16 (191.10; 193.21) | 0.00 (0.00; 0.00) | 0.00 (0.00; 0.00) | 216.22 (215.15; 217.30) |
| **Liberia** | 0.00 (0.00; 0.00) | 0.00 (0.00; 0.01) | 21.34 (20.75; 21.94) | 0.00 (0.00; 0.00) | 0.00 (0.00; 0.00) | 9.84 (9.30; 10.38) | 0.47 (0.31; 0.70) | 0.58 (0.37; 0.90) | 23.08 (22.38; 23.78) | 26.73 (18.42; 38.13) | 26.53 (17.74; 38.89) | -0.55 (-1.09; -0.01) | 27.21 (19.00; 38.97) | 27.11 (18.47; 39.39) | -0.14 (-0.66; 0.39) | 1.03 (0.72; 1.43) | 1.07 (0.76; 1.53) | 3.53 (3.03; 4.03) | 0.00 (0.00; 0.00) | 0.00 (0.00; 0.00) | 253.78 (252.26; 255.30) | 0.00 (0.00; 0.00) | 0.00 (0.00; 0.00) | 244.01 (242.50; 245.52) | 0.00 (0.00; 0.00) | 0.00 (0.00; 0.00) | 278.01 (276.07; 279.95) | 0.00 (0.00; 0.00) | 0.00 (0.00; 0.00) | 192.67 (191.49; 193.85) | 0.00 (0.00; 0.00) | 0.00 (0.00; 0.00) | 194.07 (192.91; 195.23) | 0.00 (0.00; 0.00) | 0.00 (0.00; 0.00) | 233.20 (231.98; 234.42) |
| **Mali** | 0.00 (0.00; 0.01) | 0.01 (0.00; 0.01) | 32.39 (31.85; 32.93) | 0.00 (0.00; 0.00) | 0.00 (0.00; 0.00) | 11.58 (11.11; 12.04) | 0.59 (0.37; 0.85) | 0.79 (0.52; 1.16) | 33.31 (32.64; 33.99) | 36.30 (26.12; 49.11) | 35.95 (25.33; 49.09) | -1.01 (-1.46; -0.57) | 36.92 (26.56; 51.25) | 36.74 (26.12; 49.66) | -0.46 (-0.90; -0.03) | 1.34 (0.99; 1.79) | 1.39 (1.02; 1.85) | 2.36 (1.94; 2.77) | 0.00 (0.00; 0.00) | 0.00 (0.00; 0.00) | 279.18 (277.66; 280.69) | 0.00 (0.00; 0.00) | 0.00 (0.00; 0.00) | 226.32 (225.08; 227.56) | 0.00 (0.00; 0.00) | 0.00 (0.00; 0.00) | 285.42 (283.60; 287.25) | 0.00 (0.00; 0.01) | 0.01 (0.01; 0.02) | 184.73 (183.67; 185.79) | 0.00 (0.00; 0.01) | 0.01 (0.01; 0.02) | 186.27 (185.21; 187.32) | 0.00 (0.00; 0.00) | 0.00 (0.00; 0.00) | 199.72 (198.70; 200.74) |
| **Mauritania** | 0.00 (0.00; 0.01) | 0.01 (0.00; 0.01) | 32.18 (31.61; 32.74) | 0.00 (0.00; 0.00) | 0.00 (0.00; 0.00) | 12.74 (12.24; 13.23) | 0.59 (0.37; 0.87) | 0.80 (0.51; 1.23) | 34.60 (33.86; 35.33) | 28.66 (19.89; 38.79) | 28.24 (19.45; 39.66) | -1.35 (-1.85; -0.86) | 29.26 (20.67; 39.71) | 29.04 (20.16; 40.63) | -0.62 (-1.11; -0.14) | 1.14 (0.81; 1.53) | 1.19 (0.84; 1.62) | 3.48 (3.01; 3.95) | 0.00 (0.00; 0.00) | 0.00 (0.00; 0.00) | 237.09 (235.82; 238.35) | 0.00 (0.00; 0.00) | 0.00 (0.00; 0.00) | 194.22 (193.10; 195.34) | 0.00 (0.00; 0.00) | 0.00 (0.00; 0.00) | 248.05 (246.48; 249.62) | 0.00 (0.00; 0.00) | 0.00 (0.00; 0.00) | 152.84 (151.86; 153.81) | 0.00 (0.00; 0.00) | 0.00 (0.00; 0.00) | 154.73 (153.79; 155.67) | 0.00 (0.00; 0.00) | 0.00 (0.00; 0.00) | 171.74 (170.84; 172.63) |
| **Niger** | 0.00 (0.00; 0.00) | 0.00 (0.00; 0.01) | 51.85 (51.12; 52.58) | 0.00 (0.00; 0.00) | 0.00 (0.00; 0.00) | 23.88 (23.27; 24.49) | 0.36 (0.22; 0.57) | 0.58 (0.36; 0.91) | 55.98 (55.09; 56.87) | 23.04 (15.09; 32.64) | 24.49 (15.88; 35.38) | 6.31 (5.74; 6.88) | 23.42 (15.21; 32.99) | 25.06 (16.32; 36.16) | 7.09 (6.52; 7.66) | 0.92 (0.62; 1.29) | 1.03 (0.68; 1.47) | 10.63 (10.06; 11.19) | 0.00 (0.00; 0.00) | 0.00 (0.00; 0.00) | 364.30 (362.29; 366.32) | 0.00 (0.00; 0.00) | 0.00 (0.00; 0.00) | 274.16 (272.54; 275.78) | 0.00 (0.00; 0.00) | 0.00 (0.00; 0.00) | 373.05 (370.60; 375.50) | 0.00 (0.00; 0.00) | 0.01 (0.00; 0.01) | 221.26 (219.90; 222.62) | 0.00 (0.00; 0.00) | 0.01 (0.00; 0.01) | 223.52 (222.19; 224.86) | 0.00 (0.00; 0.00) | 0.00 (0.00; 0.00) | 225.94 (224.65; 227.23) |
| **Nigeria** | 0.00 (0.00; 0.00) | 0.00 (0.00; 0.00) | 9.04 (8.53; 9.54) | 0.00 (0.00; 0.00) | 0.00 (0.00; 0.00) | 3.37 (2.95; 3.80) | 0.33 (0.21; 0.48) | 0.37 (0.24; 0.54) | 11.41 (10.79; 12.03) | 18.56 (13.29; 25.24) | 17.51 (12.18; 23.55) | -5.33 (-5.77; -4.89) | 18.85 (13.83; 25.27) | 17.87 (12.62; 24.00) | -5.04 (-5.47; -4.61) | 0.73 (0.55; 0.96) | 0.74 (0.56; 0.95) | 0.62 (0.25; 1) | 0.00 (0.00; 0.00) | 0.01 (0.01; 0.01) | 211.38 (210.04; 212.71) | 0.00 (0.00; 0.00) | 0.00 (0.00; 0.00) | 216.56 (215.34; 217.78) | 0.00 (0.00; 0.00) | 0.00 (0.00; 0.00) | 234.12 (232.56; 235.67) | 0.02 (0.01; 0.03) | 0.06 (0.04; 0.08) | 175.65 (174.64; 176.66) | 0.02 (0.01; 0.03) | 0.06 (0.04; 0.08) | 176.62 (175.63; 177.61) | 0.00 (0.00; 0.00) | 0.00 (0.00; 0.00) | 219.90 (218.96; 220.85) |
| **Sao Tome and Principe** | 0.00 (0.00; 0.00) | 0.00 (0.00; 0.00) | 24.58 (24.07; 25.09) | 0.00 (0.00; 0.00) | 0.00 (0.00; 0.00) | 16.66 (16.20; 17.13) | 0.05 (0.04; 0.08) | 0.08 (0.05; 0.11) | 34.60 (33.90; 35.30) | 2.44 (1.83; 3.26) | 2.61 (1.94; 3.48) | 6.38 (5.96; 6.80) | 2.49 (1.88; 3.33) | 2.69 (2.01; 3.58) | 7.01 (6.60; 7.43) | 0.11 (0.08; 0.15) | 0.12 (0.09; 0.16) | 10.86 (10.43; 11.29) | 0.00 (0.00; 0.00) | 0.00 (0.00; 0.00) | 184.60 (183.53; 185.67) | 0.00 (0.00; 0.00) | 0.00 (0.00; 0.00) | 187.15 (186.14; 188.15) | 0.00 (0.00; 0.00) | 0.00 (0.00; 0.00) | 223.99 (222.60; 225.38) | 0.00 (0.00; 0.00) | 0.00 (0.00; 0.00) | 146.79 (145.95; 147.62) | 0.00 (0.00; 0.00) | 0.00 (0.00; 0.00) | 148.44 (147.62; 149.26) | 0.00 (0.00; 0.00) | 0.00 (0.00; 0.00) | 183.70 (182.78; 184.63) |
| **Senegal** | 0.00 (0.00; 0.01) | 0.00 (0.00; 0.01) | 14.36 (13.81; 14.91) | 0.00 (0.00; 0.00) | 0.00 (0.00; 0.00) | 3.77 (3.28; 4.26) | 0.53 (0.33; 0.81) | 0.63 (0.40; 0.97) | 16.52 (15.82; 17.22) | 29.87 (20.63; 41.50) | 27.78 (19.56; 39.56) | -6.28 (-6.78; -5.79) | 30.43 (21.09; 42.76) | 28.41 (20.14; 40.12) | -5.88 (-6.37; -5.39) | 1.18 (0.86; 1.58) | 1.16 (0.85; 1.60) | -1.54 (-2.00; -1.09) | 0.00 (0.00; 0.00) | 0.00 (0.00; 0.00) | 200.48 (199.20; 201.75) | 0.00 (0.00; 0.00) | 0.00 (0.00; 0.00) | 183.88 (182.74; 185.01) | 0.00 (0.00; 0.00) | 0.00 (0.00; 0.00) | 214.30 (212.71; 215.89) | 0.00 (0.00; 0.00) | 0.01 (0.00; 0.01) | 149.17 (148.17; 150.16) | 0.00 (0.00; 0.00) | 0.01 (0.00; 0.01) | 150.28 (149.31; 151.26) | 0.00 (0.00; 0.00) | 0.00 (0.00; 0.00) | 173.99 (173.06; 174.92) |
| **Sierra Leone** | 0.00 (0.00; 0.00) | 0.00 (0.00; 0.00) | 2.31 (1.80; 2.82) | 0.00 (0.00; 0.00) | 0.00 (0.00; 0.00) | -4.31 (-4.76; -3.85) | 0.45 (0.28; 0.69) | 0.47 (0.29; 0.72) | 3.39 (2.73; 4.05) | 27.18 (19.07; 37.71) | 23.60 (16.22; 33.25) | -13.07 (-13.54; -12.61) | 27.64 (19.47; 38.24) | 24.07 (16.72; 33.69) | -12.80 (-13.26; -12.35) | 1.06 (0.76; 1.44) | 0.97 (0.69; 1.34) | -8.98 (-9.42; -8.54) | 0.00 (0.00; 0.00) | 0.00 (0.00; 0.00) | 193.54 (192.25; 194.82) | 0.00 (0.00; 0.00) | 0.00 (0.00; 0.00) | 181.79 (180.60; 182.98) | 0.00 (0.00; 0.00) | 0.00 (0.00; 0.00) | 202.58 (200.98; 204.18) | 0.00 (0.00; 0.00) | 0.00 (0.00; 0.00) | 151.19 (150.23; 152.16) | 0.00 (0.00; 0.00) | 0.00 (0.00; 0.00) | 152 (151.04; 152.96) | 0.00 (0.00; 0.00) | 0.00 (0.00; 0.00) | 169.28 (168.37; 170.20) |
| **Togo** | 0.00 (0.00; 0.01) | 0.00 (0.00; 0.01) | 10.06 (9.54; 10.57) | 0.00 (0.00; 0.00) | 0.00 (0.00; 0.00) | 1.55 (1.09; 2.01) | 0.56 (0.36; 0.83) | 0.63 (0.41; 0.93) | 11.83 (11.20; 12.46) | 32.03 (22.33; 44.63) | 29.71 (20.29; 41.59) | -6.71 (-7.20; -6.22) | 32.59 (22.97; 44.98) | 30.34 (21.01; 42.25) | -6.39 (-6.86; -5.91) | 1.26 (0.90; 1.69) | 1.22 (0.85; 1.69) | -3.00 (-3.46; -2.54) | 0.00 (0.00; 0.00) | 0.00 (0.00; 0.00) | 180.93 (179.78; 182.08) | 0.00 (0.00; 0.00) | 0.00 (0.00; 0.00) | 180.50 (179.40; 181.61) | 0.00 (0.00; 0.00) | 0.00 (0.00; 0.00) | 201.07 (199.64; 202.50) | 0.00 (0.00; 0.00) | 0.00 (0.00; 0.00) | 141.21 (140.34; 142.08) | 0.00 (0.00; 0.00) | 0.00 (0.00; 0.01) | 142.19 (141.33; 143.04) | 0.00 (0.00; 0.00) | 0.00 (0.00; 0.00) | 178.26 (177.36; 179.16) |

*Footnote*

Different colors reflect different geographic levels. Red: Global; Blue: Super-Regions; Green: Regions; Yellow: Country.

TPC, total percentage change.

**Appendix Table 2.** Prevalence, incidence and burden of untreated caries of deciduous teeth in 2021, with projections to 2050, in 21 GBD regions and 204 countries.

| **CARIES OF DECIDUOUS TEETH** | | | | | | | | | | | | | | | | | | |
| --- | --- | --- | --- | --- | --- | --- | --- | --- | --- | --- | --- | --- | --- | --- | --- | --- | --- | --- |
| **Location** | **Age-standardized** | | | | | | | | | **All Ages (nº of cases)** | | | | | | | | |
|  | **Prevalence (%)** | | | **Incidence (%)** | | | **YLDs (per 100,000)** | | | **Prevalence (Millions)** | | | **Incidence (Millions)** | | | **YLDs (Millions)** | | |
|  | **2021** | **2050** | **2021-2050 TPC (%)** | **2021** | **2050** | **2021-2050 TPC (%)** | **2021** | **2050** | **2021-2050 TPC (%)** | **2021** | **2050** | **2021-2050 TPC (%)** | **2021** | **2050** | **2021-2050 TPC (%)** | **2021** | **2050** | **2021-2050 TPC (%)** |
| **Global** | 7.55 (6.29; 8.78) | 7.27 (6.08; 8.45) | -2.93 (-3.16; -2.71) | 17.78 (13.95; 23.04) | 17.49 (13.74; 22.34) | -0.98 (-1.33; -0.62) | 2.89 (1.27; 5.54) | 2.78 (1.22; 5.35) | -2.97 (-4.01; -1.94) | 524.63 (437.68; 611.23) | 436.76 (348.71; 534.17) | -15.80 (-16.04; -15.55) | 1253.26 (978.66; 1637.29) | 1071.34 (787.37; 1415.41) | -14.02 (-14.38; -13.66) | 0.20 (0.09; 0.39) | 0.17 (0.07; 0.33) | -15.82 (-16.81; -14.83) |
| **Central Europe, Eastern Europe, and Central Asia** | 8.93 (7.44; 10.59) | 8.86 (7.39; 10.52) | -0.24 (-0.49; 0.00) | 20.62 (15.31; 29.35) | 20.64 (15.32; 29.40) | 0.78 (0.30; 1.27) | 3.43 (1.48; 6.56) | 3.40 (1.46; 6.54) | -0.30 (-1.35; 0.75) | 24.84 (20.61; 29.54) | 19.02 (15.35; 23.46) | -21.95 (-22.20; -21.69) | 58.88 (43.41; 84.73) | 45.51 (32.18; 65.51) | -21.75 (-22.23; -21.28) | 0.01 (0.00; 0.02) | 0.01 (0.00; 0.01) | -21.96 (-22.93; -21) |
| **Central Asia** | 8.95 (7.46; 10.69) | 8.83 (7.34; 10.54) | -0.36 (-0.61; -0.10) | 20.66 (15.17; 29.10) | 20.60 (15.13; 29.08) | 0.51 (0.03; 0.99) | 3.42 (1.48; 6.73) | 3.37 (1.46; 6.61) | -0.37 (-1.45; 0.71) | 8.78 (7.33; 10.47) | 8.78 (6.76; 11.18) | -0.71 (-1.05; -0.37) | 20.07 (14.85; 28.01) | 20.69 (14.05; 29.80) | 1.88 (1.36; 2.41) | 0.00 (0.00; 0.01) | 0.00 (0.00; 0.01) | -0.69 (-1.80; 0.41) |
| **Armenia** | 8.90 (7.34; 10.61) | 8.85 (7.29; 10.54) | -0.23 (-0.49; 0.03) | 20.62 (15.26; 29.49) | 20.57 (15.21; 29.48) | 0.40 (-0.09; 0.89) | 3.41 (1.45; 6.95) | 3.38 (1.44; 6.90) | -0.23 (-1.37; 0.91) | 0.18 (0.15; 0.22) | 0.09 (0.06; 0.12) | -50.32 (-50.61; -50.02) | 0.43 (0.32; 0.63) | 0.21 (0.13; 0.32) | -49.99 (-50.44; -49.53) | 0.00 (0.00; 0.00) | 0.00 (0.00; 0.00) | -50.22 (-51.12; -49.32) |
| **Azerbaijan** | 8.86 (7.33; 10.53) | 8.82 (7.30; 10.49) | -0.22 (-0.48; 0.03) | 20.78 (15.06; 29.33) | 20.79 (15.04; 29.34) | 0.60 (0.11; 1.09) | 3.41 (1.49; 6.77) | 3.39 (1.48; 6.75) | -0.22 (-1.31; 0.88) | 0.72 (0.60; 0.87) | 0.47 (0.34; 0.64) | -32.94 (-33.27; -32.62) | 1.75 (1.25; 2.50) | 1.16 (0.75; 1.75) | -32.67 (-33.14; -32.20) | 0.00 (0.00; 0.00) | 0.00 (0.00; 0.00) | -32.89 (-33.86; -31.92) |
| **Georgia** | 9.90 (8.13; 11.36) | 9.86 (8.09; 11.30) | -0.23 (-0.46; 0.00) | 19.35 (13.74; 28.17) | 18.54 (13.12; 27.19) | -3.34 (-3.86; -2.81) | 3.79 (1.57; 7.89) | 3.77 (1.56; 7.85) | -0.22 (-1.40; 0.96) | 0.26 (0.21; 0.30) | 0.20 (0.15; 0.25) | -23.23 (-23.52; -22.94) | 0.51 (0.36; 0.73) | 0.38 (0.25; 0.58) | -25.45 (-25.96; -24.95) | 0.00 (0.00; 0.00) | 0.00 (0.00; 0.00) | -23.26 (-24.27; -22.25) |
| **Kazakhstan** | 8.87 (7.30; 10.66) | 8.73 (7.18; 10.50) | -0.27 (-0.53; 0.00) | 20.71 (15.20; 29.51) | 20.68 (15.14; 29.32) | 0.69 (0.20; 1.18) | 3.38 (1.45; 6.69) | 3.33 (1.43; 6.58) | -0.26 (-1.35; 0.83) | 1.68 (1.39; 2.02) | 1.76 (1.35; 2.21) | 3.11 (2.77; 3.44) | 3.92 (2.88; 5.54) | 4.19 (2.85; 5.89) | 5.31 (4.80; 5.81) | 0.00 (0.00; 0.00) | 0.00 (0.00; 0.00) | 3.06 (1.93; 4.20) |
| **Kyrgyzstan** | 9.01 (7.48; 10.72) | 8.92 (7.41; 10.61) | -0.25 (-0.50; 0.00) | 20.74 (15.17; 29.91) | 20.69 (15.16; 30.00) | 0.44 (-0.07; 0.94) | 3.44 (1.49; 6.73) | 3.41 (1.47; 6.67) | -0.24 (-1.32; 0.83) | 0.73 (0.61; 0.87) | 0.65 (0.47; 0.88) | -10.44 (-10.80; -10.07) | 1.68 (1.23; 2.39) | 1.53 (1.01; 2.22) | -9.08 (-9.59; -8.56) | 0.00 (0.00; 0.00) | 0.00 (0.00; 0.00) | -10.42 (-11.50; -9.35) |
| **Mongolia** | 8.83 (7.23; 10.53) | 8.73 (7.15; 10.41) | -0.34 (-0.61; -0.08) | 20.85 (15.04; 29.59) | 20.96 (15.19; 29.64) | 1.28 (0.78; 1.77) | 3.40 (1.43; 6.57) | 3.36 (1.42; 6.50) | -0.34 (-1.40; 0.73) | 0.35 (0.29; 0.42) | 0.45 (0.33; 0.57) | 27.20 (26.80; 27.61) | 0.82 (0.60; 1.12) | 1.08 (0.73; 1.60) | 29.87 (29.26; 30.48) | 0.00 (0.00; 0.00) | 0.00 (0.00; 0.00) | 27.25 (25.99; 28.51) |
| **Tajikistan** | 9.04 (7.45; 10.76) | 8.90 (7.33; 10.61) | -0.32 (-0.58; -0.06) | 20.76 (15.20; 29.34) | 20.76 (15.19; 29.42) | 0.82 (0.33; 1.30) | 3.47 (1.49; 6.50) | 3.41 (1.47; 6.40) | -0.31 (-1.33; 0.71) | 1.16 (0.96; 1.37) | 1.35 (1.01; 1.74) | 15.09 (14.69; 15.48) | 2.60 (1.93; 3.62) | 3.17 (2.15; 4.58) | 19.21 (18.63; 19.78) | 0.00 (0.00; 0.00) | 0.00 (0.00; 0.00) | 15.13 (13.96; 16.29) |
| **Turkmenistan** | 8.85 (7.17; 10.78) | 8.74 (7.08; 10.65) | -0.26 (-0.55; 0.02) | 20.47 (15.20; 28.75) | 20.50 (15.19; 28.84) | 0.90 (0.42; 1.37) | 3.38 (1.44; 6.79) | 3.34 (1.42; 6.71) | -0.26 (-1.38; 0.86) | 0.47 (0.38; 0.58) | 0.47 (0.33; 0.63) | -0.88 (-1.26; -0.49) | 1.09 (0.81; 1.52) | 1.12 (0.76; 1.63) | 1.63 (1.12; 2.15) | 0.00 (0.00; 0.00) | 0.00 (0.00; 0.00) | -0.71 (-1.86; 0.44) |
| **Uzbekistan** | 8.93 (7.38; 10.76) | 8.80 (7.26; 10.61) | -0.28 (-0.55; -0.02) | 20.66 (15.03; 28.58) | 20.54 (14.96; 28.51) | 0.21 (-0.25; 0.68) | 3.41 (1.50; 6.82) | 3.36 (1.47; 6.73) | -0.28 (-1.38; 0.83) | 3.23 (2.68; 3.88) | 3.34 (2.18; 4.88) | 2.18 (1.64; 2.71) | 7.28 (5.36; 9.92) | 7.85 (4.62; 12.43) | 6.08 (5.40; 6.76) | 0.00 (0.00; 0.00) | 0.00 (0.00; 0.00) | 2.22 (1.03; 3.41) |
| **Central Europe** | 8.92 (7.37; 10.55) | 8.88 (7.34; 10.51) | 0.09 (-0.16; 0.34) | 20.30 (14.96; 28.72) | 20.73 (15.27; 29.29) | 2.70 (2.22; 3.19) | 3.43 (1.47; 6.58) | 3.42 (1.46; 6.57) | 0.10 (-0.96; 1.16) | 5.30 (4.37; 6.29) | 3.39 (2.67; 4.25) | -34.12 (-34.36; -33.88) | 12.39 (8.98; 18.03) | 8.20 (5.65; 12.01) | -32.12 (-32.57; -31.67) | 0.00 (0.00; 0.00) | 0.00 (0.00; 0.00) | -34.07 (-34.99; -33.15) |
| **Albania** | 8.61 (7.04; 10.29) | 8.53 (6.97; 10.19) | -0.24 (-0.51; 0.02) | 20.44 (14.90; 29.38) | 20.39 (14.90; 29.36) | 0.44 (-0.06; 0.94) | 3.30 (1.40; 6.61) | 3.27 (1.38; 6.55) | -0.24 (-1.35; 0.88) | 0.13 (0.11; 0.16) | 0.08 (0.05; 0.11) | -39.41 (-39.71; -39.10) | 0.31 (0.23; 0.46) | 0.19 (0.12; 0.30) | -38.37 (-38.85; -37.89) | 0.00 (0.00; 0.00) | 0.00 (0.00; 0.00) | -39.33 (-40.26; -38.40) |
| **Bosnia and Herzegovina** | 9.38 (7.65; 11.41) | 9.32 (7.61; 11.34) | -0.25 (-0.54; 0.03) | 19.84 (14.14; 29.02) | 20.23 (14.46; 29.43) | 2.45 (1.92; 2.98) | 3.61 (1.55; 7.16) | 3.59 (1.54; 7.11) | -0.25 (-1.34; 0.85) | 0.16 (0.13; 0.19) | 0.07 (0.05; 0.10) | -55.35 (-55.65; -55.05) | 0.34 (0.24; 0.51) | 0.15 (0.09; 0.23) | -54.15 (-54.60; -53.70) | 0.00 (0.00; 0.00) | 0.00 (0.00; 0.00) | -55.35 (-56.25; -54.46) |
| **Bulgaria** | 8.76 (7.09; 10.48) | 8.69 (7.03; 10.39) | -0.17 (-0.45; 0.10) | 20.19 (14.59; 29.53) | 20.09 (14.48; 29.30) | 0.21 (-0.32; 0.73) | 3.37 (1.41; 6.74) | 3.34 (1.40; 6.69) | -0.17 (-1.29; 0.95) | 0.28 (0.23; 0.34) | 0.18 (0.14; 0.23) | -35.37 (-35.66; -35.09) | 0.68 (0.48; 1.03) | 0.43 (0.29; 0.64) | -35.16 (-35.64; -34.69) | 0.00 (0.00; 0.00) | 0.00 (0.00; 0.00) | -35.31 (-36.24; -34.37) |
| **Croatia** | 8.89 (7.27; 10.71) | 8.80 (7.21; 10.61) | -0.20 (-0.48; 0.07) | 18.60 (14.85; 23.34) | 18.48 (14.64; 23.22) | 0.16 (-0.17; 0.49) | 3.43 (1.44; 6.88) | 3.40 (1.42; 6.81) | -0.20 (-1.32; 0.92) | 0.18 (0.14; 0.21) | 0.08 (0.06; 0.12) | -50.42 (-50.70; -50.13) | 0.38 (0.30; 0.48) | 0.18 (0.13; 0.26) | -50.17 (-50.51; -49.83) | 0.00 (0.00; 0.00) | 0.00 (0.00; 0.00) | -50.40 (-51.28; -49.51) |
| **Czechia** | 8.77 (7.14; 10.48) | 8.70 (7.08; 10.41) | -0.14 (-0.41; 0.13) | 20.39 (14.32; 29.46) | 20.30 (14.24; 29.44) | 0.26 (-0.27; 0.79) | 3.38 (1.46; 6.54) | 3.35 (1.45; 6.49) | -0.14 (-1.20; 0.93) | 0.51 (0.41; 0.61) | 0.40 (0.31; 0.52) | -22.05 (-22.36; -21.75) | 1.21 (0.84; 1.78) | 0.94 (0.63; 1.38) | -21.50 (-22; -21.01) | 0.00 (0.00; 0.00) | 0.00 (0.00; 0.00) | -22 (-22.94; -21.05) |
| **Hungary** | 8.89 (7.26; 10.63) | 8.81 (7.19; 10.53) | -0.18 (-0.45; 0.08) | 20.21 (14.78; 28.36) | 20.82 (15.19; 29.33) | 3.52 (3.04; 4.01) | 3.41 (1.45; 6.68) | 3.38 (1.43; 6.61) | -0.18 (-1.26; 0.90) | 0.42 (0.34; 0.50) | 0.38 (0.28; 0.49) | -10.19 (-10.53; -9.86) | 0.97 (0.70; 1.39) | 0.91 (0.63; 1.35) | -5.90 (-6.42; -5.38) | 0.00 (0.00; 0.00) | 0.00 (0.00; 0.00) | -10.14 (-11.18; -9.09) |
| **Montenegro** | 8.63 (6.99; 10.31) | 8.55 (6.93; 10.21) | -0.19 (-0.46; 0.08) | 20.40 (14.69; 28.78) | 20.30 (14.65; 28.56) | 0.24 (-0.25; 0.73) | 3.32 (1.41; 6.38) | 3.29 (1.40; 6.32) | -0.19 (-1.24; 0.87) | 0.03 (0.03; 0.04) | 0.02 (0.02; 0.03) | -33.98 (-34.28; -33.69) | 0.08 (0.06; 0.11) | 0.05 (0.04; 0.08) | -33.08 (-33.53; -32.62) | 0.00 (0.00; 0.00) | 0.00 (0.00; 0.00) | -34 (-34.94; -33.05) |
| **North Macedonia** | 8.63 (7.02; 10.24) | 8.58 (6.98; 10.18) | -0.21 (-0.47; 0.06) | 20.33 (14.57; 28.79) | 20.29 (14.54; 28.48) | 0.41 (-0.08; 0.90) | 3.30 (1.39; 6.67) | 3.28 (1.38; 6.62) | -0.20 (-1.33; 0.92) | 0.10 (0.08; 0.11) | 0.05 (0.04; 0.06) | -49.04 (-49.36; -48.72) | 0.23 (0.16; 0.34) | 0.12 (0.08; 0.18) | -48.37 (-48.83; -47.90) | 0.00 (0.00; 0.00) | 0.00 (0.00; 0.00) | -48.98 (-49.91; -48.05) |
| **Poland** | 8.63 (7.07; 10.35) | 8.59 (7.04; 10.29) | -0.21 (-0.48; 0.06) | 20.64 (15.03; 29.04) | 20.48 (14.94; 28.77) | -0.13 (-0.61; 0.35) | 3.31 (1.42; 6.28) | 3.30 (1.42; 6.25) | -0.20 (-1.24; 0.83) | 1.72 (1.41; 2.08) | 1.03 (0.78; 1.34) | -39.47 (-39.74; -39.20) | 4.20 (3.03; 6.05) | 2.54 (1.71; 3.66) | -39.02 (-39.44; -38.60) | 0.00 (0.00; 0.00) | 0.00 (0.00; 0.00) | -39.43 (-40.34; -38.53) |
| **Romania** | 10.52 (8.86; 12.26) | 10.43 (8.78; 12.14) | -0.21 (-0.44; 0.02) | 21.72 (15.51; 31.14) | 23.86 (17.09; 34.07) | 10.17 (9.64; 10.70) | 4.06 (1.72; 8.07) | 4.02 (1.71; 8.00) | -0.20 (-1.31; 0.90) | 1.05 (0.87; 1.23) | 0.68 (0.51; 0.89) | -33.63 (-33.92; -33.33) | 2.25 (1.57; 3.39) | 1.65 (1.03; 2.56) | -26.13 (-26.68; -25.58) | 0.00 (0.00; 0.00) | 0.00 (0.00; 0.00) | -33.51 (-34.51; -32.51) |
| **Serbia** | 7.03 (5.62; 8.61) | 6.99 (5.59; 8.57) | -0.30 (-0.60; 0.00) | 16.33 (11.02; 23.66) | 16.01 (10.81; 23.09) | -1.45 (-1.99; -0.91) | 2.70 (1.09; 5.35) | 2.68 (1.08; 5.32) | -0.29 (-1.41; 0.82) | 0.30 (0.24; 0.37) | 0.15 (0.11; 0.20) | -48.15 (-48.45; -47.86) | 0.73 (0.47; 1.12) | 0.36 (0.22; 0.57) | -49.27 (-49.82; -48.72) | 0.00 (0.00; 0.00) | 0.00 (0.00; 0.00) | -48.19 (-49.08; -47.30) |
| **Slovakia** | 8.76 (7.17; 10.55) | 8.69 (7.10; 10.46) | -0.13 (-0.41; 0.14) | 20.33 (14.64; 28.46) | 20.26 (14.56; 28.41) | 0.42 (-0.07; 0.90) | 3.37 (1.45; 6.61) | 3.34 (1.43; 6.55) | -0.13 (-1.21; 0.95) | 0.26 (0.21; 0.31) | 0.19 (0.15; 0.24) | -25.12 (-25.40; -24.84) | 0.60 (0.43; 0.86) | 0.46 (0.32; 0.65) | -23.68 (-24.14; -23.23) | 0.00 (0.00; 0.00) | 0.00 (0.00; 0.00) | -25.07 (-26.05; -24.10) |
| **Slovenia** | 8.63 (7.04; 10.30) | 8.57 (6.99; 10.22) | -0.17 (-0.44; 0.09) | 20.27 (14.73; 28.74) | 20.41 (14.85; 28.86) | 1.33 (0.83; 1.82) | 3.31 (1.40; 6.79) | 3.29 (1.39; 6.74) | -0.17 (-1.32; 0.98) | 0.09 (0.07; 0.11) | 0.09 (0.07; 0.11) | -5.45 (-5.76; -5.15) | 0.22 (0.16; 0.32) | 0.21 (0.15; 0.30) | -5.14 (-5.64; -4.64) | 0.00 (0.00; 0.00) | 0.00 (0.00; 0.00) | -5.53 (-6.61; -4.45) |
| **Eastern Europe** | 8.93 (7.46; 10.56) | 8.91 (7.46; 10.52) | -0.22 (-0.46; 0.02) | 20.74 (15.48; 29.31) | 20.65 (15.43; 29.11) | 0.24 (-0.23; 0.71) | 3.43 (1.47; 6.55) | 3.42 (1.47; 6.54) | -0.24 (-1.28; 0.81) | 10.76 (8.97; 12.84) | 6.86 (5.42; 8.59) | -34.01 (-34.26; -33.76) | 26.42 (19.46; 38.04) | 16.62 (11.57; 24.42) | -35.51 (-35.97; -35.06) | 0.00 (0.00; 0.01) | 0.00 (0.00; 0.01) | -33.97 (-34.89; -33.05) |
| **Belarus** | 8.99 (7.32; 10.82) | 8.96 (7.30; 10.78) | -0.19 (-0.46; 0.09) | 20.68 (15.04; 30.69) | 20.66 (15.03; 30.70) | 0.53 (-0.01; 1.07) | 3.46 (1.49; 6.80) | 3.45 (1.49; 6.77) | -0.18 (-1.26; 0.90) | 0.49 (0.40; 0.60) | 0.32 (0.23; 0.44) | -33.53 (-33.86; -33.19) | 1.18 (0.85; 1.77) | 0.77 (0.49; 1.18) | -34.28 (-34.78; -33.78) | 0.00 (0.00; 0.00) | 0.00 (0.00; 0.00) | -33.47 (-34.42; -32.52) |
| **Estonia** | 8.90 (7.14; 10.95) | 8.84 (7.09; 10.87) | -0.17 (-0.47; 0.13) | 20.68 (15.26; 30.65) | 20.62 (15.14; 30.49) | 0.37 (-0.15; 0.90) | 3.42 (1.50; 6.71) | 3.40 (1.48; 6.66) | -0.17 (-1.24; 0.91) | 0.06 (0.05; 0.08) | 0.05 (0.03; 0.06) | -26.84 (-27.17; -26.51) | 0.15 (0.11; 0.23) | 0.11 (0.08; 0.17) | -26.39 (-26.90; -25.89) | 0.00 (0.00; 0.00) | 0.00 (0.00; 0.00) | -26.78 (-27.75; -25.80) |
| **Latvia** | 8.12 (6.25; 10.10) | 8.09 (6.22; 10.05) | -0.15 (-0.48; 0.19) | 19.54 (13.97; 27.64) | 19.42 (13.85; 27.57) | 0 (-0.50; 0.49) | 3.14 (1.33; 6.08) | 3.12 (1.33; 6.06) | -0.14 (-1.21; 0.93) | 0.08 (0.06; 0.10) | 0.04 (0.03; 0.06) | -45.16 (-45.48; -44.84) | 0.21 (0.15; 0.29) | 0.11 (0.07; 0.17) | -45.52 (-45.97; -45.06) | 0.00 (0.00; 0.00) | 0.00 (0.00; 0.00) | -45.02 (-45.94; -44.09) |
| **Lithuania** | 9.67 (7.83; 11.42) | 9.62 (7.80; 11.35) | -0.18 (-0.44; 0.08) | 21.46 (15.66; 31.78) | 20.85 (15.23; 30.96) | -1.91 (-2.44; -1.38) | 3.73 (1.61; 7.35) | 3.71 (1.60; 7.31) | -0.18 (-1.27; 0.91) | 0.14 (0.11; 0.16) | 0.07 (0.05; 0.09) | -50.44 (-50.70; -50.19) | 0.31 (0.22; 0.46) | 0.15 (0.10; 0.22) | -51.61 (-52.04; -51.19) | 0.00 (0.00; 0.00) | 0.00 (0.00; 0.00) | -50.41 (-51.23; -49.59) |
| **Republic of Moldova** | 9.17 (7.42; 11.05) | 9.12 (7.39; 10.99) | -0.18 (-0.46; 0.10) | 20.54 (14.90; 29.98) | 20.49 (14.88; 30.08) | 0.46 (-0.06; 0.99) | 3.51 (1.54; 6.91) | 3.49 (1.53; 6.88) | -0.17 (-1.25; 0.91) | 0.16 (0.13; 0.20) | 0.05 (0.03; 0.08) | -66.52 (-66.84; -66.19) | 0.38 (0.27; 0.57) | 0.12 (0.06; 0.20) | -66.74 (-67.23; -66.26) | 0.00 (0.00; 0.00) | 0.00 (0.00; 0.00) | -66.55 (-67.37; -65.73) |
| **Russian Federation** | 8.90 (7.47; 10.49) | 8.89 (7.47; 10.46) | -0.14 (-0.38; 0.10) | 20.63 (15.51; 28.61) | 20.59 (15.45; 28.60) | 0.48 (0.02; 0.93) | 3.42 (1.46; 6.53) | 3.41 (1.46; 6.51) | -0.14 (-1.18; 0.91) | 7.97 (6.70; 9.46) | 5.52 (4.31; 6.84) | -28.57 (-28.84; -28.30) | 19.40 (14.42; 27.31) | 13.33 (9.29; 19.57) | -29.87 (-30.33; -29.41) | 0.00 (0.00; 0.01) | 0.00 (0.00; 0.00) | -28.53 (-29.45; -27.60) |
| **Ukraine** | 9.01 (7.36; 10.76) | 9.02 (7.37; 10.77) | -0.07 (-0.34; 0.19) | 21.20 (15.37; 30.80) | 21.07 (15.30; 30.58) | 0.09 (-0.43; 0.60) | 3.47 (1.50; 6.72) | 3.47 (1.50; 6.72) | -0.07 (-1.14; 0.99) | 1.85 (1.50; 2.23) | 0.81 (0.57; 1.12) | -53.76 (-54.09; -53.43) | 4.79 (3.37; 7.29) | 2.03 (1.28; 3.21) | -55.60 (-56.10; -55.10) | 0.00 (0.00; 0.00) | 0.00 (0.00; 0.00) | -53.73 (-54.63; -52.82) |
| **High-income** | 5.86 (4.75; 7.13) | 5.79 (4.68; 7.05) | -0.56 (-0.84; -0.27) | 16.20 (12.15; 20.77) | 16.38 (12.26; 21.00) | 1.62 (1.24; 2.00) | 2.25 (0.94; 4.42) | 2.22 (0.93; 4.38) | -0.57 (-1.66; 0.53) | 35.23 (28.39; 43.05) | 30.56 (23.74; 38.11) | -12.18 (-12.47; -11.89) | 99.82 (74.34; 129.48) | 88.20 (65.19; 117.70) | -10.81 (-11.20; -10.43) | 0.01 (0.01; 0.03) | 0.01 (0.00; 0.02) | -12.16 (-13.19; -11.13) |
| **Australasia** | 8.15 (6.85; 9.35) | 8.11 (6.80; 9.34) | 0.20 (-0.02; 0.42) | 20.19 (14.37; 25.11) | 20.08 (14.30; 24.89) | -0.23 (-0.60; 0.15) | 3.12 (1.33; 6.27) | 3.10 (1.32; 6.23) | 0.22 (-0.90; 1.34) | 1.64 (1.38; 1.88) | 1.85 (1.46; 2.26) | 12.35 (12.06; 12.65) | 4.20 (2.98; 5.27) | 4.73 (3.28; 6.17) | 11.60 (11.16; 12.04) | 0.00 (0.00; 0.00) | 0.00 (0.00; 0.00) | 12.35 (11.20; 13.50) |
| **Australia** | 9.09 (7.60; 10.39) | 9.01 (7.54; 10.30) | -0.28 (-0.49; -0.06) | 21.65 (15.49; 27.11) | 21.38 (15.21; 26.76) | -0.93 (-1.31; -0.55) | 3.48 (1.50; 6.98) | 3.45 (1.48; 6.92) | -0.26 (-1.37; 0.85) | 1.52 (1.27; 1.74) | 1.72 (1.35; 2.10) | 12.78 (12.48; 13.08) | 3.75 (2.65; 4.72) | 4.23 (2.86; 5.57) | 11.73 (11.26; 12.20) | 0.00 (0.00; 0.00) | 0.00 (0.00; 0.00) | 12.78 (11.61; 13.94) |
| **New Zealand** | 3.53 (2.18; 5.10) | 3.50 (2.16; 5.06) | -0.25 (-0.83; 0.34) | 13.19 (7.59; 19.48) | 13.54 (7.88; 20.07) | 3.24 (2.59; 3.89) | 1.35 (0.51; 2.96) | 1.34 (0.50; 2.93) | -0.24 (-1.52; 1.04) | 0.12 (0.07; 0.17) | 0.13 (0.08; 0.19) | 7.04 (6.39; 7.69) | 0.46 (0.26; 0.68) | 0.51 (0.29; 0.76) | 10.53 (9.83; 11.24) | 0.00 (0.00; 0.00) | 0.00 (0.00; 0.00) | 7 (5.66; 8.34) |
| **High-income Asia Pacific** | 6.58 (5.54; 7.59) | 6.47 (5.45; 7.52) | -1.51 (-1.73; -1.29) | 17.73 (14.06; 22.40) | 17.71 (14.13; 22.12) | 0.27 (-0.06; 0.60) | 2.53 (1.13; 4.71) | 2.48 (1.12; 4.68) | -1.53 (-2.52; -0.53) | 4.99 (4.18; 5.77) | 3.44 (2.77; 4.14) | -29.13 (-29.35; -28.91) | 13.99 (10.97; 18.09) | 9.68 (7.41; 12.60) | -29.30 (-29.62; -28.98) | 0.00 (0.00; 0.00) | 0.00 (0.00; 0.00) | -29.10 (-30.02; -28.19) |
| **Brunei Darussalam** | 7.65 (5.95; 9.42) | 7.56 (5.88; 9.31) | -0.26 (-0.58; 0.06) | 20.13 (14.76; 27.20) | 20.07 (14.66; 27.02) | 0.46 (0.02; 0.90) | 2.93 (1.20; 5.71) | 2.89 (1.19; 5.64) | -0.25 (-1.34; 0.84) | 0.02 (0.02; 0.03) | 0.01 (0.01; 0.02) | -41.93 (-42.26; -41.61) | 0.07 (0.05; 0.09) | 0.04 (0.02; 0.06) | -40.80 (-41.25; -40.35) | 0.00 (0.00; 0.00) | 0.00 (0.00; 0.00) | -41.77 (-42.67; -40.87) |
| **Japan** | 5.94 (4.78; 7.06) | 5.91 (4.76; 7.03) | -0.19 (-0.46; 0.08) | 16.17 (12.69; 19.75) | 16.27 (12.76; 19.88) | 1.08 (0.77; 1.39) | 2.28 (1.02; 4.46) | 2.27 (1.01; 4.44) | -0.19 (-1.25; 0.88) | 3.09 (2.50; 3.69) | 2.29 (1.73; 2.87) | -24.57 (-24.86; -24.28) | 8.65 (6.73; 10.63) | 6.43 (4.68; 8.15) | -24.36 (-24.68; -24.04) | 0.00 (0.00; 0.00) | 0.00 (0.00; 0.00) | -24.54 (-25.52; -23.57) |
| **Republic of Korea** | 8.35 (6.73; 10.41) | 8.39 (6.76; 10.43) | -0.20 (-0.51; 0.11) | 21.25 (14.66; 31.34) | 21.78 (15.08; 31.83) | 2.60 (2.04; 3.15) | 3.22 (1.37; 6.40) | 3.24 (1.37; 6.44) | -0.19 (-1.30; 0.91) | 1.68 (1.36; 2.12) | 0.95 (0.75; 1.23) | -40.98 (-41.25; -40.70) | 4.68 (3.14; 7.17) | 2.58 (1.73; 3.90) | -42.75 (-43.26; -42.25) | 0.00 (0.00; 0.00) | 0.00 (0.00; 0.00) | -40.88 (-41.81; -39.95) |
| **Singapore** | 6.36 (4.86; 7.94) | 6.31 (4.82; 7.87) | -0.25 (-0.59; 0.09) | 20.21 (14.78; 26.39) | 20.57 (15.12; 26.61) | 2.32 (1.92; 2.73) | 2.44 (1.04; 4.79) | 2.42 (1.04; 4.75) | -0.25 (-1.33; 0.84) | 0.19 (0.14; 0.23) | 0.19 (0.14; 0.26) | -0.44 (-0.85; -0.04) | 0.60 (0.44; 0.77) | 0.63 (0.43; 0.85) | 2.58 (2.12; 3.04) | 0.00 (0.00; 0.00) | 0.00 (0.00; 0.00) | -0.48 (-1.59; 0.62) |
| **High-income North America** | 6.13 (4.84; 7.50) | 6.07 (4.78; 7.43) | -0.38 (-0.69; -0.07) | 17.07 (12.24; 21.61) | 17.01 (12.19; 21.52) | 0.31 (-0.07; 0.70) | 2.35 (0.98; 4.58) | 2.33 (0.97; 4.54) | -0.38 (-1.46; 0.70) | 13.55 (10.67; 16.66) | 12.53 (9.50; 15.94) | -6.76 (-7.10; -6.42) | 38.54 (27.41; 49.15) | 35.82 (25.28; 47.22) | -6.30 (-6.70; -5.89) | 0.01 (0.00; 0.01) | 0.00 (0.00; 0.01) | -6.75 (-7.82; -5.67) |
| **Canada** | 5.52 (4.10; 7.11) | 5.49 (4.08; 7.07) | -0.16 (-0.54; 0.23) | 16.28 (10.70; 21.87) | 16.23 (10.66; 21.77) | 0.25 (-0.23; 0.74) | 2.12 (0.86; 4.17) | 2.11 (0.86; 4.14) | -0.16 (-1.26; 0.95) | 1.16 (0.85; 1.51) | 1.36 (0.95; 1.83) | 17.20 (16.72; 17.68) | 3.51 (2.27; 4.79) | 4.10 (2.59; 5.74) | 16.67 (16.09; 17.24) | 0.00 (0.00; 0.00) | 0.00 (0.00; 0.00) | 17.16 (15.92; 18.40) |
| **Greenland** | 6.43 (4.76; 8.21) | 6.39 (4.72; 8.15) | -0.21 (-0.59; 0.17) | 16.96 (11.45; 22.74) | 16.89 (11.39; 22.67) | 0.21 (-0.26; 0.68) | 2.47 (1.04; 5.01) | 2.46 (1.03; 4.97) | -0.21 (-1.34; 0.92) | 0.00 (0.00; 0.00) | 0.00 (0.00; 0.00) | -14.06 (-14.44; -13.67) | 0.01 (0.00; 0.01) | 0.01 (0.00; 0.01) | -12.98 (-13.48; -12.48) | 0.00 (0.00; 0.00) | 0.00 (0.00; 0.00) | -14.09 (-15.13; -13.05) |
| **United States of America** | 6.20 (4.92; 7.55) | 6.15 (4.88; 7.50) | -0.10 (-0.41; 0.20) | 17.15 (12.46; 21.74) | 17.12 (12.44; 21.70) | 0.46 (0.08; 0.85) | 2.37 (0.99; 4.63) | 2.36 (0.98; 4.60) | -0.10 (-1.18; 0.98) | 12.38 (9.81; 15.17) | 11.17 (8.50; 14.15) | -9.03 (-9.36; -8.70) | 35.02 (25.13; 44.59) | 31.71 (22.61; 41.72) | -8.62 (-9.01; -8.23) | 0.00 (0.00; 0.01) | 0.00 (0.00; 0.01) | -9.01 (-10.08; -7.94) |
| **Southern Latin America** | 7.41 (5.88; 9.24) | 7.43 (5.92; 9.26) | 0.04 (-0.28; 0.36) | 18.96 (13.69; 25.97) | 19.07 (13.78; 26.07) | 0.87 (0.41; 1.33) | 2.85 (1.18; 5.71) | 2.85 (1.18; 5.72) | 0.07 (-1.06; 1.19) | 3.72 (2.93; 4.67) | 2.49 (1.81; 3.32) | -31.35 (-31.67; -31.03) | 9.84 (6.99; 13.66) | 6.57 (4.42; 9.81) | -31.86 (-32.30; -31.41) | 0.00 (0.00; 0.00) | 0.00 (0.00; 0.00) | -31.28 (-32.25; -30.31) |
| **Argentina** | 7.19 (5.49; 9.07) | 7.19 (5.49; 9.07) | -0.29 (-0.64; 0.06) | 17.96 (12.48; 24.68) | 17.89 (12.50; 24.44) | -0.18 (-0.66; 0.29) | 2.76 (1.14; 5.53) | 2.76 (1.14; 5.53) | -0.28 (-1.40; 0.84) | 2.55 (1.94; 3.24) | 1.61 (1.11; 2.22) | -34.81 (-35.17; -34.46) | 6.58 (4.54; 9.13) | 4.13 (2.69; 6.32) | -35.74 (-36.22; -35.26) | 0.00 (0.00; 0.00) | 0.00 (0.00; 0.00) | -34.76 (-35.71; -33.81) |
| **Chile** | 7.95 (6.32; 9.68) | 7.92 (6.29; 9.63) | -0.25 (-0.55; 0.04) | 21.56 (15.53; 29.89) | 21.68 (15.68; 29.89) | 1.02 (0.55; 1.49) | 3.06 (1.30; 6.16) | 3.05 (1.29; 6.14) | -0.25 (-1.37; 0.88) | 0.99 (0.79; 1.21) | 0.76 (0.56; 0.98) | -21.89 (-22.21; -21.57) | 2.78 (1.98; 3.96) | 2.14 (1.45; 3.05) | -22.09 (-22.54; -21.65) | 0.00 (0.00; 0.00) | 0.00 (0.00; 0.00) | -21.81 (-22.85; -20.77) |
| **Uruguay** | 7.92 (6.22; 9.88) | 7.90 (6.21; 9.86) | -0.29 (-0.62; 0.04) | 20.04 (14.43; 27.88) | 20.06 (14.54; 27.70) | 0.51 (0.04; 0.98) | 3.03 (1.28; 6.13) | 3.03 (1.27; 6.13) | -0.28 (-1.42; 0.85) | 0.18 (0.14; 0.23) | 0.11 (0.08; 0.16) | -35.05 (-35.39; -34.71) | 0.48 (0.34; 0.67) | 0.30 (0.20; 0.46) | -35.73 (-36.20; -35.26) | 0.00 (0.00; 0.00) | 0.00 (0.00; 0.00) | -34.96 (-35.91; -34.01) |
| **Western Europe** | 4.84 (3.81; 6.14) | 4.84 (3.80; 6.13) | 0.72 (0.38; 1.07) | 13.96 (10.27; 18.97) | 14.62 (10.68; 20.10) | 5.15 (4.68; 5.61) | 1.86 (0.78; 3.76) | 1.86 (0.78; 3.74) | 0.72 (-0.41; 1.86) | 11.32 (8.88; 14.45) | 10.24 (7.75; 13.28) | -8.75 (-9.10; -8.40) | 33.25 (24.18; 45.86) | 31.40 (22.27; 43.33) | -5.13 (-5.59; -4.67) | 0.00 (0.00; 0.01) | 0.00 (0.00; 0.01) | -8.72 (-9.78; -7.65) |
| **Andorra** | 5.65 (4.15; 7.43) | 5.61 (4.13; 7.40) | -0.12 (-0.53; 0.29) | 16.13 (10.82; 23.10) | 16.08 (10.77; 23.01) | 0.18 (-0.36; 0.71) | 2.17 (0.89; 4.55) | 2.16 (0.88; 4.52) | -0.12 (-1.31; 1.07) | 0.00 (0.00; 0.00) | 0.00 (0.00; 0.00) | -38.71 (-39.08; -38.34) | 0.01 (0.00; 0.01) | 0.00 (0.00; 0.00) | -40.10 (-40.60; -39.60) | 0.00 (0.00; 0.00) | 0.00 (0.00; 0.00) | -38.66 (-39.63; -37.70) |
| **Austria** | 5.65 (4.13; 7.51) | 5.60 (4.10; 7.45) | -0.19 (-0.61; 0.23) | 15.97 (10.58; 22.50) | 15.91 (10.51; 22.39) | 0.26 (-0.26; 0.79) | 2.18 (0.88; 4.71) | 2.16 (0.87; 4.68) | -0.18 (-1.43; 1.06) | 0.25 (0.19; 0.34) | 0.25 (0.17; 0.34) | -2.28 (-2.73; -1.84) | 0.72 (0.48; 1.02) | 0.72 (0.46; 1.05) | -1.26 (-1.84; -0.69) | 0.00 (0.00; 0.00) | 0.00 (0.00; 0.00) | -2.21 (-3.45; -0.98) |
| **Belgium** | 5.10 (3.60; 7.05) | 5.05 (3.57; 6.99) | -0.26 (-0.74; 0.22) | 14.01 (8.90; 19.83) | 14.62 (9.35; 20.84) | 4.74 (4.17; 5.31) | 1.96 (0.80; 3.95) | 1.94 (0.79; 3.91) | -0.25 (-1.39; 0.88) | 0.34 (0.23; 0.47) | 0.33 (0.22; 0.47) | -1.40 (-1.91; -0.89) | 0.94 (0.59; 1.35) | 0.96 (0.60; 1.42) | 3.03 (2.43; 3.63) | 0.00 (0.00; 0.00) | 0.00 (0.00; 0.00) | -1.34 (-2.53; -0.15) |
| **Cyprus** | 5.70 (4.19; 7.53) | 5.64 (4.14; 7.44) | -0.29 (-0.70; 0.12) | 16.03 (10.72; 22.31) | 15.89 (10.62; 22.10) | -0.09 (-0.60; 0.42) | 2.19 (0.91; 4.27) | 2.16 (0.89; 4.23) | -0.28 (-1.37; 0.81) | 0.04 (0.03; 0.06) | 0.03 (0.02; 0.05) | -22.89 (-23.34; -22.44) | 0.12 (0.08; 0.17) | 0.10 (0.06; 0.14) | -22.15 (-22.68; -21.62) | 0.00 (0.00; 0.00) | 0.00 (0.00; 0.00) | -22.88 (-23.91; -21.86) |
| **Denmark** | 3.27 (2.04; 4.78) | 3.24 (2.02; 4.73) | -0.21 (-0.80; 0.38) | 11.39 (6.91; 17.21) | 11.54 (6.94; 17.39) | 2.04 (1.39; 2.69) | 1.26 (0.48; 2.70) | 1.24 (0.48; 2.67) | -0.21 (-1.46; 1.04) | 0.10 (0.07; 0.15) | 0.11 (0.07; 0.16) | 5.51 (4.89; 6.14) | 0.37 (0.22; 0.56) | 0.40 (0.23; 0.62) | 8.02 (7.32; 8.72) | 0.00 (0.00; 0.00) | 0.00 (0.00; 0.00) | 5.55 (4.26; 6.85) |
| **Finland** | 5.71 (4.26; 7.46) | 5.66 (4.22; 7.40) | -0.24 (-0.63; 0.16) | 16.46 (11.01; 22.62) | 16.43 (10.99; 22.59) | 0.40 (-0.10; 0.90) | 2.19 (0.90; 4.40) | 2.17 (0.89; 4.36) | -0.23 (-1.36; 0.90) | 0.16 (0.12; 0.22) | 0.14 (0.10; 0.18) | -14.35 (-14.78; -13.93) | 0.49 (0.32; 0.69) | 0.41 (0.27; 0.59) | -14.98 (-15.49; -14.46) | 0.00 (0.00; 0.00) | 0.00 (0.00; 0.00) | -14.32 (-15.39; -13.26) |
| **France** | 4.81 (3.17; 6.62) | 4.76 (3.15; 6.56) | -0.28 (-0.79; 0.22) | 12.81 (7.61; 18.38) | 13.92 (8.18; 20.02) | 8.92 (8.30; 9.55) | 1.85 (0.70; 3.87) | 1.83 (0.70; 3.84) | -0.28 (-1.49; 0.94) | 1.93 (1.28; 2.65) | 1.55 (0.96; 2.29) | -18.50 (-19.01; -17.99) | 5.22 (3.03; 7.51) | 4.58 (2.53; 6.85) | -11.23 (-11.84; -10.62) | 0.00 (0.00; 0.00) | 0.00 (0.00; 0.00) | -18.46 (-19.58; -17.33) |
| **Germany** | 3.89 (3.10; 5.03) | 3.86 (3.07; 4.99) | -0.18 (-0.53; 0.17) | 14.93 (9.48; 21.22) | 15.73 (10.02; 22.43) | 5.81 (5.24; 6.39) | 1.49 (0.64; 2.99) | 1.48 (0.63; 2.97) | -0.18 (-1.30; 0.94) | 1.63 (1.29; 2.11) | 1.55 (1.18; 2.02) | -5.61 (-5.98; -5.23) | 6.27 (3.96; 8.92) | 6.38 (3.87; 9.70) | 0.50 (-0.15; 1.14) | 0.00 (0.00; 0.00) | 0.00 (0.00; 0.00) | -5.62 (-6.70; -4.53) |
| **Greece** | 3.47 (2.66; 4.35) | 3.44 (2.63; 4.31) | -0.30 (-0.64; 0.05) | 12.24 (6.24; 18.09) | 14.08 (7.14; 20.80) | 15.14 (14.40; 15.88) | 1.33 (0.56; 2.73) | 1.31 (0.56; 2.71) | -0.30 (-1.45; 0.86) | 0.16 (0.12; 0.21) | 0.11 (0.07; 0.14) | -33.79 (-34.16; -33.42) | 0.58 (0.29; 0.89) | 0.44 (0.21; 0.71) | -23.64 (-24.35; -22.93) | 0.00 (0.00; 0.00) | 0.00 (0.00; 0.00) | -33.65 (-34.67; -32.63) |
| **Iceland** | 5.67 (4.17; 7.52) | 5.59 (4.12; 7.43) | -0.18 (-0.60; 0.23) | 15.95 (10.59; 22.46) | 15.85 (10.53; 22.34) | 0.27 (-0.26; 0.79) | 2.18 (0.90; 4.52) | 2.16 (0.89; 4.47) | -0.18 (-1.35; 0.99) | 0.01 (0.01; 0.02) | 0.02 (0.01; 0.02) | 26.06 (25.54; 26.58) | 0.04 (0.02; 0.05) | 0.05 (0.03; 0.07) | 26.65 (25.98; 27.31) | 0.00 (0.00; 0.00) | 0.00 (0.00; 0.00) | 25.98 (24.63; 27.34) |
| **Ireland** | 6.15 (4.55; 8.03) | 6.11 (4.52; 7.98) | -0.24 (-0.64; 0.16) | 16.22 (10.61; 22.37) | 16.14 (10.53; 22.20) | 0.01 (-0.51; 0.52) | 2.37 (0.99; 4.86) | 2.35 (0.99; 4.83) | -0.23 (-1.38; 0.93) | 0.21 (0.16; 0.27) | 0.21 (0.15; 0.28) | 2.87 (2.43; 3.31) | 0.57 (0.36; 0.79) | 0.57 (0.36; 0.82) | 1.66 (1.09; 2.24) | 0.00 (0.00; 0.00) | 0.00 (0.00; 0.00) | 2.95 (1.79; 4.11) |
| **Israel** | 7.85 (6.17; 9.43) | 7.77 (6.11; 9.34) | -0.24 (-0.53; 0.05) | 18.60 (13.11; 26.75) | 18.44 (13.04; 26.59) | -0.17 (-0.69; 0.35) | 3.01 (1.28; 6.32) | 2.98 (1.26; 6.26) | -0.23 (-1.42; 0.95) | 0.72 (0.57; 0.87) | 0.83 (0.59; 1.06) | 13.31 (12.90; 13.72) | 1.71 (1.21; 2.46) | 1.97 (1.30; 2.87) | 13.86 (13.27; 14.45) | 0.00 (0.00; 0.00) | 0.00 (0.00; 0.00) | 13.36 (12.18; 14.54) |
| **Italy** | 5.34 (4.18; 6.51) | 5.32 (4.16; 6.48) | -0.25 (-0.56; 0.05) | 13.88 (10.72; 17.54) | 13.97 (10.83; 17.62) | 1.04 (0.69; 1.38) | 2.05 (0.93; 4.06) | 2.04 (0.92; 4.04) | -0.25 (-1.33; 0.83) | 1.36 (1.06; 1.64) | 0.90 (0.67; 1.17) | -31.47 (-31.77; -31.18) | 3.63 (2.76; 4.61) | 2.42 (1.75; 3.25) | -31.48 (-31.82; -31.13) | 0.00 (0.00; 0.00) | 0.00 (0.00; 0.00) | -31.45 (-32.39; -30.52) |
| **Luxembourg** | 5.56 (4.07; 7.35) | 5.51 (4.03; 7.28) | -0.15 (-0.57; 0.27) | 16.07 (10.59; 22.15) | 16.00 (10.54; 22.09) | 0.30 (-0.21; 0.82) | 2.13 (0.86; 4.15) | 2.11 (0.85; 4.11) | -0.15 (-1.24; 0.94) | 0.02 (0.01; 0.03) | 0.03 (0.02; 0.04) | 37.12 (36.57; 37.66) | 0.06 (0.04; 0.08) | 0.08 (0.05; 0.11) | 38.22 (37.57; 38.87) | 0.00 (0.00; 0.00) | 0.00 (0.00; 0.00) | 37.15 (35.78; 38.53) |
| **Malta** | 5.69 (4.25; 7.45) | 5.63 (4.21; 7.37) | -0.38 (-0.77; 0.02) | 16.12 (10.61; 22.86) | 16.15 (10.59; 22.93) | 0.85 (0.31; 1.39) | 2.19 (0.89; 4.40) | 2.16 (0.88; 4.35) | -0.37 (-1.50; 0.76) | 0.01 (0.01; 0.02) | 0.01 (0.01; 0.02) | 15.50 (14.97; 16.03) | 0.04 (0.02; 0.05) | 0.04 (0.03; 0.07) | 18.19 (17.54; 18.85) | 0.00 (0.00; 0.00) | 0.00 (0.00; 0.00) | 15.48 (14.22; 16.73) |
| **Monaco** | 5.50 (4.03; 7.19) | 5.45 (3.99; 7.14) | -0.17 (-0.57; 0.24) | 16.02 (10.51; 21.93) | 15.97 (10.54; 21.91) | 0.38 (-0.12; 0.89) | 2.10 (0.82; 4.15) | 2.08 (0.81; 4.13) | -0.16 (-1.28; 0.96) | 0.00 (0.00; 0.00) | 0.00 (0.00; 0.00) | -28.48 (-28.91; -28.05) | 0.00 (0.00; 0.00) | 0.00 (0.00; 0.00) | -28.03 (-28.52; -27.54) | 0.00 (0.00; 0.00) | 0.00 (0.00; 0.00) | -28.55 (-29.59; -27.51) |
| **Netherlands** | 5.45 (4.50; 6.42) | 5.38 (4.45; 6.34) | -0.21 (-0.46; 0.04) | 14.61 (10.93; 18.75) | 14.51 (10.86; 18.63) | 0.16 (-0.22; 0.53) | 2.08 (0.94; 4.23) | 2.06 (0.93; 4.18) | -0.21 (-1.32; 0.91) | 0.49 (0.41; 0.58) | 0.49 (0.40; 0.59) | 0.39 (0.12; 0.66) | 1.34 (1.00; 1.74) | 1.35 (0.99; 1.79) | 1.18 (0.76; 1.60) | 0.00 (0.00; 0.00) | 0.00 (0.00; 0.00) | 0.43 (-0.70; 1.55) |
| **Norway** | 5.22 (3.85; 6.82) | 5.18 (3.82; 6.77) | -0.13 (-0.53; 0.28) | 16.64 (11.10; 21.94) | 16.57 (11.07; 21.84) | 0.16 (-0.30; 0.62) | 2.00 (0.84; 3.97) | 1.99 (0.84; 3.95) | -0.12 (-1.23; 0.98) | 0.17 (0.12; 0.22) | 0.16 (0.11; 0.22) | -1.66 (-2.11; -1.21) | 0.54 (0.36; 0.72) | 0.52 (0.34; 0.70) | -2.05 (-2.53; -1.56) | 0.00 (0.00; 0.00) | 0.00 (0.00; 0.00) | -1.64 (-2.77; -0.51) |
| **Portugal** | 5.42 (4.22; 7.15) | 5.36 (4.17; 7.08) | -0.39 (-0.78; -0.01) | 16.11 (10.57; 22.29) | 16.24 (10.59; 22.63) | 1.37 (0.85; 1.89) | 2.09 (0.88; 4.31) | 2.07 (0.87; 4.26) | -0.38 (-1.54; 0.77) | 0.25 (0.19; 0.33) | 0.23 (0.16; 0.30) | -7.56 (-7.98; -7.14) | 0.74 (0.48; 1.04) | 0.70 (0.44; 1.03) | -5.21 (-5.78; -4.64) | 0.00 (0.00; 0.00) | 0.00 (0.00; 0.00) | -7.56 (-8.65; -6.48) |
| **San Marino** | 5.66 (4.15; 7.35) | 5.62 (4.12; 7.30) | -0.21 (-0.61; 0.19) | 13.64 (10.19; 17.41) | 13.63 (10.23; 17.37) | 0.44 (0.07; 0.82) | 2.18 (0.91; 4.44) | 2.16 (0.90; 4.41) | -0.20 (-1.34; 0.94) | 0.00 (0.00; 0.00) | 0.00 (0.00; 0.00) | -27.64 (-28.07; -27.21) | 0.00 (0.00; 0.00) | 0.00 (0.00; 0.00) | -27.60 (-28; -27.20) | 0.00 (0.00; 0.00) | 0.00 (0.00; 0.00) | -27.67 (-28.68; -26.67) |
| **Spain** | 5.29 (3.76; 7.10) | 5.25 (3.74; 7.06) | -0.30 (-0.75; 0.15) | 14.45 (9.33; 20.03) | 15.66 (10.15; 21.62) | 8.42 (7.88; 8.97) | 2.02 (0.82; 4.09) | 2.01 (0.81; 4.06) | -0.29 (-1.43; 0.85) | 1.17 (0.83; 1.58) | 1.19 (0.80; 1.65) | 2.99 (2.48; 3.49) | 3.29 (2.08; 4.60) | 3.60 (2.26; 5.16) | 10.35 (9.74; 10.95) | 0.00 (0.00; 0.00) | 0.00 (0.00; 0.00) | 3 (1.75; 4.25) |
| **Sweden** | 6.38 (4.78; 8.08) | 6.32 (4.74; 8.01) | -0.19 (-0.56; 0.17) | 19.73 (14.60; 27.69) | 19.72 (14.53; 27.91) | 0.64 (0.16; 1.11) | 2.44 (1.03; 4.75) | 2.42 (1.02; 4.71) | -0.19 (-1.26; 0.89) | 0.40 (0.30; 0.51) | 0.47 (0.34; 0.61) | 17.22 (16.78; 17.65) | 1.26 (0.92; 1.80) | 1.48 (1.06; 2.10) | 17.59 (17.05; 18.13) | 0.00 (0.00; 0.00) | 0.00 (0.00; 0.00) | 17.27 (16.04; 18.49) |
| **Switzerland** | 3.11 (2.28; 4.37) | 3.09 (2.26; 4.34) | -0.11 (-0.58; 0.37) | 12.61 (7.23; 19.03) | 12.56 (7.20; 18.97) | 0.30 (-0.37; 0.96) | 1.19 (0.50; 2.42) | 1.18 (0.49; 2.40) | -0.11 (-1.25; 1.04) | 0.14 (0.11; 0.20) | 0.14 (0.10; 0.20) | -3.75 (-4.25; -3.26) | 0.59 (0.34; 0.90) | 0.58 (0.33; 0.90) | -3.33 (-4.01; -2.64) | 0.00 (0.00; 0.00) | 0.00 (0.00; 0.00) | -3.76 (-4.89; -2.63) |
| **United Kingdom** | 4.28 (3.20; 5.49) | 4.25 (3.18; 5.46) | -0.24 (-0.62; 0.14) | 11.41 (7.88; 15.45) | 11.30 (7.80; 15.29) | -0.42 (-0.89; 0.05) | 1.64 (0.71; 3.27) | 1.63 (0.71; 3.25) | -0.23 (-1.33; 0.87) | 1.74 (1.29; 2.24) | 1.49 (1.08; 2.00) | -13.15 (-13.53; -12.77) | 4.71 (3.19; 6.40) | 4.02 (2.65; 5.60) | -13.77 (-14.23; -13.32) | 0.00 (0.00; 0.00) | 0.00 (0.00; 0.00) | -13.02 (-14.07; -11.98) |
| **Latin America and Caribbean** | 7.49 (6.18; 8.84) | 7.44 (6.13; 8.81) | 0.13 (-0.13; 0.38) | 18.62 (14.38; 24.24) | 18.60 (14.32; 24.39) | 0.63 (0.25; 1.01) | 2.87 (1.24; 5.48) | 2.85 (1.23; 5.46) | 0.11 (-0.93; 1.16) | 36.81 (30.30; 43.62) | 27.88 (22.01; 34.35) | -23.91 (-24.16; -23.66) | 92.72 (71.48; 122.15) | 71.50 (52.73; 97.70) | -22.67 (-23.04; -22.30) | 0.01 (0.01; 0.03) | 0.01 (0.00; 0.02) | -23.90 (-24.83; -22.97) |
| **Andean Latin America** | 7.87 (6.53; 9.24) | 7.78 (6.48; 9.15) | -0.29 (-0.53; -0.05) | 18.90 (14.47; 24.42) | 19.26 (14.84; 24.71) | 2.62 (2.25; 3.00) | 3.01 (1.29; 5.85) | 2.98 (1.27; 5.78) | -0.27 (-1.34; 0.80) | 4.93 (4.10; 5.80) | 4.01 (3.11; 5.09) | -18.82 (-19.10; -18.54) | 11.90 (9.09; 15.46) | 10.08 (7.31; 13.57) | -15.58 (-15.95; -15.21) | 0.00 (0.00; 0.00) | 0.00 (0.00; 0.00) | -18.76 (-19.72; -17.81) |
| **Bolivia (Plurinational State of)** | 7.84 (6.14; 9.63) | 7.74 (6.06; 9.51) | -0.37 (-0.68; -0.05) | 19.33 (13.98; 26.22) | 19.25 (13.93; 26.14) | 0.34 (-0.11; 0.79) | 3.01 (1.23; 5.84) | 2.97 (1.22; 5.77) | -0.36 (-1.44; 0.72) | 0.95 (0.74; 1.16) | 0.86 (0.61; 1.16) | -9.67 (-10.06; -9.28) | 2.35 (1.70; 3.20) | 2.17 (1.42; 3.04) | -7.88 (-8.36; -7.40) | 0.00 (0.00; 0.00) | 0.00 (0.00; 0.00) | -9.65 (-10.75; -8.55) |
| **Ecuador** | 8.03 (7.37; 8.69) | 7.96 (7.31; 8.61) | -0.32 (-0.43; -0.20) | 17.99 (14.00; 22.00) | 19.44 (15.27; 23.79) | 8.37 (8.05; 8.70) | 3.08 (1.37; 6.06) | 3.05 (1.36; 6.01) | -0.31 (-1.39; 0.77) | 1.40 (1.29; 1.52) | 1.17 (0.89; 1.53) | -15.96 (-16.24; -15.67) | 3.17 (2.46; 3.90) | 2.90 (2.01; 4.14) | -8.40 (-8.82; -7.97) | 0.00 (0.00; 0.00) | 0.00 (0.00; 0.00) | -15.70 (-16.74; -14.67) |
| **Peru** | 7.79 (6.11; 9.61) | 7.70 (6.04; 9.49) | -0.28 (-0.59; 0.04) | 19.24 (13.78; 25.50) | 19.18 (13.70; 25.42) | 0.51 (0.08; 0.95) | 2.97 (1.26; 6.01) | 2.94 (1.25; 5.93) | -0.27 (-1.40; 0.85) | 2.58 (2.02; 3.18) | 1.98 (1.44; 2.68) | -23.72 (-24.07; -23.37) | 6.38 (4.56; 8.48) | 5.01 (3.34; 7.03) | -21.95 (-22.37; -21.53) | 0.00 (0.00; 0.00) | 0.00 (0.00; 0.00) | -23.78 (-24.80; -22.75) |
| **Caribbean** | 7.76 (6.54; 8.98) | 7.67 (6.49; 8.90) | -0.22 (-0.44; 0.00) | 17.36 (13.44; 21.47) | 17.29 (13.33; 21.45) | 0.44 (0.11; 0.77) | 2.97 (1.35; 5.87) | 2.94 (1.33; 5.81) | -0.25 (-1.32; 0.83) | 3.06 (2.58; 3.55) | 2.31 (1.76; 2.96) | -24.21 (-24.47; -23.96) | 6.90 (5.33; 8.54) | 5.29 (3.73; 7.11) | -23.07 (-23.42; -22.72) | 0.00 (0.00; 0.00) | 0.00 (0.00; 0.00) | -24.23 (-25.17; -23.30) |
| **Antigua and Barbuda** | 8.12 (6.47; 9.92) | 8.04 (6.40; 9.82) | -0.25 (-0.55; 0.05) | 19.93 (14.31; 27.68) | 19.87 (14.20; 27.57) | 0.43 (-0.05; 0.91) | 3.10 (1.35; 6.34) | 3.07 (1.33; 6.28) | -0.24 (-1.38; 0.90) | 0.00 (0.00; 0.01) | 0.00 (0.00; 0.00) | -31.13 (-31.45; -30.81) | 0.01 (0.01; 0.02) | 0.01 (0.01; 0.01) | -30.08 (-30.56; -29.60) | 0.00 (0.00; 0.00) | 0.00 (0.00; 0.00) | -31.11 (-32.15; -30.08) |
| **Bahamas** | 7.88 (6.19; 9.66) | 7.84 (6.15; 9.61) | -0.17 (-0.48; 0.14) | 19.88 (14.46; 27.20) | 19.84 (14.39; 27.14) | 0.36 (-0.09; 0.81) | 3.01 (1.29; 5.98) | 2.99 (1.29; 5.95) | -0.16 (-1.27; 0.94) | 0.02 (0.02; 0.03) | 0.01 (0.01; 0.02) | -22.91 (-23.29; -22.52) | 0.05 (0.04; 0.08) | 0.04 (0.03; 0.06) | -24.81 (-25.31; -24.30) | 0.00 (0.00; 0.00) | 0.00 (0.00; 0.00) | -22.84 (-23.88; -21.80) |
| **Barbados** | 8.15 (6.55; 9.87) | 8.09 (6.51; 9.80) | -0.24 (-0.53; 0.05) | 20.00 (14.53; 28.06) | 19.97 (14.51; 27.98) | 0.46 (-0.02; 0.94) | 3.12 (1.34; 5.98) | 3.10 (1.33; 5.94) | -0.23 (-1.28; 0.82) | 0.01 (0.01; 0.02) | 0.01 (0.01; 0.01) | -35.34 (-35.68; -35) | 0.03 (0.02; 0.05) | 0.02 (0.01; 0.03) | -35.30 (-35.78; -34.81) | 0.00 (0.00; 0.00) | 0.00 (0.00; 0.00) | -35.24 (-36.14; -34.34) |
| **Belize** | 8.21 (6.54; 10.00) | 8.12 (6.47; 9.90) | -0.32 (-0.62; -0.02) | 19.99 (14.47; 28.52) | 19.94 (14.45; 28.36) | 0.41 (-0.09; 0.90) | 3.15 (1.37; 6.12) | 3.12 (1.36; 6.06) | -0.31 (-1.38; 0.75) | 0.03 (0.03; 0.04) | 0.03 (0.02; 0.05) | 0.58 (0.15; 1.01) | 0.08 (0.06; 0.12) | 0.08 (0.06; 0.13) | 0.98 (0.40; 1.56) | 0.00 (0.00; 0.00) | 0.00 (0.00; 0.00) | 0.68 (-0.40; 1.76) |
| **Bermuda** | 8.01 (6.41; 9.88) | 7.94 (6.35; 9.80) | -0.21 (-0.52; 0.09) | 20.05 (14.28; 27.66) | 20.05 (14.32; 27.77) | 0.63 (0.16; 1.11) | 3.06 (1.32; 6.11) | 3.04 (1.31; 6.06) | -0.21 (-1.32; 0.89) | 0.00 (0.00; 0.00) | 0.00 (0.00; 0.00) | -36.55 (-36.86; -36.24) | 0.01 (0.00; 0.01) | 0.00 (0.00; 0.01) | -36.31 (-36.77; -35.86) | 0.00 (0.00; 0.00) | 0.00 (0.00; 0.00) | -36.48 (-37.44; -35.52) |
| **Cuba** | 7.50 (5.97; 9.17) | 7.47 (5.94; 9.12) | -0.23 (-0.53; 0.08) | 15.78 (10.39; 20.48) | 15.79 (10.41; 20.52) | 0.41 (-0.04; 0.86) | 2.88 (1.26; 6.22) | 2.87 (1.25; 6.19) | -0.22 (-1.44; 0.99) | 0.45 (0.36; 0.56) | 0.25 (0.19; 0.32) | -43.12 (-43.41; -42.83) | 0.97 (0.63; 1.27) | 0.54 (0.35; 0.77) | -42.87 (-43.29; -42.44) | 0.00 (0.00; 0.00) | 0.00 (0.00; 0.00) | -43.05 (-44.02; -42.08) |
| **Dominica** | 8.21 (6.45; 9.90) | 8.18 (6.43; 9.86) | -0.24 (-0.54; 0.05) | 19.90 (14.45; 27.39) | 19.87 (14.48; 27.32) | 0.32 (-0.14; 0.78) | 3.14 (1.32; 6.10) | 3.13 (1.31; 6.08) | -0.24 (-1.31; 0.84) | 0.00 (0.00; 0.00) | 0.00 (0.00; 0.00) | -40.82 (-41.15; -40.48) | 0.01 (0.01; 0.01) | 0.01 (0.00; 0.01) | -42.53 (-43.03; -42.03) | 0.00 (0.00; 0.00) | 0.00 (0.00; 0.00) | -40.66 (-41.58; -39.74) |
| **Dominican Republic** | 8.13 (6.43; 9.93) | 8.02 (6.33; 9.79) | -0.41 (-0.72; -0.11) | 19.90 (14.29; 27.49) | 19.85 (14.24; 27.54) | 0.54 (0.06; 1.01) | 3.12 (1.32; 5.94) | 3.08 (1.30; 5.86) | -0.41 (-1.45; 0.64) | 0.83 (0.66; 1.01) | 0.68 (0.48; 0.94) | -18.51 (-18.90; -18.12) | 2.03 (1.45; 2.81) | 1.71 (1.06; 2.58) | -15.84 (-16.36; -15.31) | 0.00 (0.00; 0.00) | 0.00 (0.00; 0.00) | -18.53 (-19.54; -17.51) |
| **Grenada** | 8.19 (6.51; 10.05) | 8.11 (6.44; 9.94) | -0.34 (-0.65; -0.04) | 19.87 (14.41; 27.46) | 19.84 (14.31; 27.38) | 0.51 (0.05; 0.98) | 3.14 (1.33; 6.13) | 3.11 (1.32; 6.06) | -0.33 (-1.41; 0.74) | 0.01 (0.00; 0.01) | 0.00 (0.00; 0.00) | -41.05 (-41.39; -40.71) | 0.01 (0.01; 0.02) | 0.01 (0.01; 0.01) | -39.89 (-40.34; -39.43) | 0.00 (0.00; 0.00) | 0.00 (0.00; 0.00) | -41.16 (-42.11; -40.22) |
| **Guyana** | 8.20 (6.56; 10.00) | 8.09 (6.47; 9.86) | -0.49 (-0.79; -0.19) | 19.99 (14.37; 28.05) | 20.09 (14.49; 28.16) | 1.11 (0.62; 1.59) | 3.14 (1.34; 6.46) | 3.09 (1.32; 6.37) | -0.48 (-1.63; 0.68) | 0.06 (0.05; 0.07) | 0.04 (0.02; 0.05) | -40.08 (-40.43; -39.74) | 0.15 (0.11; 0.21) | 0.09 (0.05; 0.14) | -38.46 (-38.92; -38) | 0.00 (0.00; 0.00) | 0.00 (0.00; 0.00) | -39.64 (-40.66; -38.62) |
| **Haiti** | 7.59 (6.01; 9.24) | 7.46 (5.90; 9.09) | -0.42 (-0.73; -0.12) | 15.84 (10.68; 20.57) | 15.68 (10.64; 20.41) | 0.15 (-0.30; 0.59) | 2.90 (1.27; 6.10) | 2.85 (1.25; 6.00) | -0.42 (-1.60; 0.76) | 1.15 (0.91; 1.40) | 1.02 (0.67; 1.46) | -12.31 (-12.74; -11.89) | 2.39 (1.62; 3.10) | 2.17 (1.30; 3.31) | -10.69 (-11.24; -10.14) | 0.00 (0.00; 0.00) | 0.00 (0.00; 0.00) | -12.35 (-13.47; -11.22) |
| **Jamaica** | 8.21 (6.50; 10.22) | 8.15 (6.45; 10.15) | -0.23 (-0.55; 0.09) | 19.98 (14.42; 27.33) | 19.92 (14.39; 27.30) | 0.30 (-0.16; 0.76) | 3.15 (1.33; 6.32) | 3.13 (1.32; 6.28) | -0.23 (-1.34; 0.89) | 0.16 (0.12; 0.20) | 0.08 (0.05; 0.10) | -50.43 (-50.75; -50.12) | 0.40 (0.28; 0.58) | 0.19 (0.12; 0.30) | -50.17 (-50.62; -49.72) | 0.00 (0.00; 0.00) | 0.00 (0.00; 0.00) | -50.34 (-51.22; -49.46) |
| **Puerto Rico** | 7.31 (5.77; 8.97) | 7.33 (5.80; 8.98) | -0.16 (-0.47; 0.15) | 15.95 (10.72; 21.06) | 16.04 (10.79; 21.12) | 0.49 (0.03; 0.94) | 2.81 (1.23; 5.94) | 2.82 (1.23; 5.95) | -0.16 (-1.34; 1.02) | 0.10 (0.08; 0.12) | 0.03 (0.02; 0.04) | -65.66 (-65.92; -65.40) | 0.23 (0.15; 0.31) | 0.07 (0.05; 0.10) | -66.11 (-66.49; -65.73) | 0.00 (0.00; 0.00) | 0.00 (0.00; 0.00) | -65.70 (-66.58; -64.81) |
| **Saint Kitts and Nevis** | 8.10 (6.45; 9.89) | 8.04 (6.40; 9.82) | -0.27 (-0.57; 0.03) | 19.87 (14.54; 27.53) | 19.86 (14.53; 27.48) | 0.54 (0.08; 1.01) | 3.10 (1.32; 6.23) | 3.07 (1.31; 6.19) | -0.27 (-1.39; 0.86) | 0.00 (0.00; 0.00) | 0.00 (0.00; 0.00) | -46.75 (-47.09; -46.41) | 0.01 (0.00; 0.01) | 0.00 (0.00; 0.01) | -45.54 (-46.03; -45.05) | 0.00 (0.00; 0.00) | 0.00 (0.00; 0.00) | -46.68 (-47.60; -45.76) |
| **Saint Lucia** | 7.80 (6.73; 9.02) | 7.75 (6.68; 8.95) | -0.30 (-0.50; -0.09) | 17.92 (14.06; 22.75) | 17.85 (13.98; 22.71) | 0.18 (-0.17; 0.53) | 2.99 (1.34; 5.92) | 2.97 (1.33; 5.88) | -0.29 (-1.37; 0.79) | 0.01 (0.01; 0.01) | 0.00 (0.00; 0.01) | -39.11 (-39.40; -38.82) | 0.02 (0.01; 0.02) | 0.01 (0.01; 0.02) | -38.58 (-38.97; -38.19) | 0.00 (0.00; 0.00) | 0.00 (0.00; 0.00) | -39.04 (-39.95; -38.13) |
| **Saint Vincent and the Grenadines** | 7.58 (6.00; 9.24) | 7.56 (5.99; 9.21) | -0.33 (-0.63; -0.03) | 15.98 (10.89; 20.93) | 19.23 (12.85; 25.65) | 19.60 (19.10; 20.11) | 2.90 (1.31; 6.08) | 2.90 (1.30; 6.06) | -0.33 (-1.49; 0.83) | 0.01 (0.00; 0.01) | 0.00 (0.00; 0.00) | -44.18 (-44.50; -43.86) | 0.01 (0.01; 0.02) | 0.01 (0.01; 0.01) | -33.61 (-34.07; -33.16) | 0.00 (0.00; 0.00) | 0.00 (0.00; 0.00) | -43.99 (-44.94; -43.04) |
| **Suriname** | 8.74 (7.12; 10.40) | 8.65 (7.05; 10.30) | -0.31 (-0.57; -0.04) | 17.88 (13.94; 22.68) | 17.87 (13.95; 22.54) | 0.63 (0.29; 0.98) | 3.35 (1.42; 6.59) | 3.32 (1.41; 6.53) | -0.30 (-1.39; 0.79) | 0.04 (0.03; 0.05) | 0.03 (0.03; 0.05) | -16.62 (-16.97; -16.27) | 0.09 (0.07; 0.11) | 0.07 (0.05; 0.10) | -16.24 (-16.65; -15.83) | 0.00 (0.00; 0.00) | 0.00 (0.00; 0.00) | -16.44 (-17.45; -15.42) |
| **Trinidad and Tobago** | 6.54 (4.95; 8.11) | 6.52 (4.94; 8.08) | -0.26 (-0.60; 0.08) | 15.85 (10.70; 20.30) | 16.22 (10.93; 20.73) | 2.59 (2.16; 3.02) | 2.50 (1.11; 5.11) | 2.50 (1.11; 5.08) | -0.26 (-1.38; 0.87) | 0.06 (0.05; 0.08) | 0.03 (0.02; 0.04) | -56.63 (-56.95; -56.30) | 0.15 (0.10; 0.19) | 0.06 (0.04; 0.09) | -55.64 (-56.05; -55.24) | 0.00 (0.00; 0.00) | 0.00 (0.00; 0.00) | -56.59 (-57.47; -55.71) |
| **United States Virgin Islands** | 8.02 (6.37; 9.85) | 7.98 (6.34; 9.81) | -0.20 (-0.51; 0.10) | 20.00 (14.41; 28.13) | 19.97 (14.40; 28.07) | 0.39 (-0.09; 0.88) | 3.06 (1.31; 6.14) | 3.05 (1.30; 6.11) | -0.20 (-1.31; 0.91) | 0.00 (0.00; 0.00) | 0.00 (0.00; 0.00) | -37.46 (-37.78; -37.13) | 0.01 (0.01; 0.01) | 0.01 (0.00; 0.01) | -37.91 (-38.40; -37.41) | 0.00 (0.00; 0.00) | 0.00 (0.00; 0.00) | -37.53 (-38.48; -36.58) |
| **Central Latin America** | 7.15 (5.94; 8.31) | 7.10 (5.90; 8.24) | -0.17 (-0.41; 0.06) | 17.46 (13.71; 21.57) | 17.26 (13.52; 21.37) | -0.42 (-0.74; -0.10) | 2.75 (1.23; 5.20) | 2.73 (1.22; 5.16) | -0.17 (-1.19; 0.85) | 15.46 (12.88; 18.04) | 10.84 (8.22; 14.04) | -29 (-29.26; -28.74) | 38.44 (29.95; 48.19) | 27.03 (19.78; 36.94) | -28.92 (-29.25; -28.58) | 0.01 (0.00; 0.01) | 0.00 (0.00; 0.01) | -28.94 (-29.85; -28.03) |
| **Colombia** | 6.39 (5.36; 7.40) | 6.31 (5.30; 7.32) | -0.40 (-0.63; -0.18) | 14.60 (9.99; 19.49) | 13.84 (9.53; 18.39) | -4.30 (-4.75; -3.86) | 2.45 (1.12; 4.72) | 2.42 (1.11; 4.67) | -0.40 (-1.43; 0.64) | 2.29 (1.92; 2.66) | 1.59 (1.13; 2.20) | -30.01 (-30.31; -29.71) | 5.26 (3.60; 7.02) | 3.51 (2.12; 5.23) | -32.60 (-33.06; -32.14) | 0.00 (0.00; 0.00) | 0.00 (0.00; 0.00) | -29.89 (-30.84; -28.94) |
| **Costa Rica** | 7.22 (5.49; 9.03) | 7.20 (5.48; 9.01) | -0.30 (-0.64; 0.05) | 18.86 (13.37; 26.76) | 18.90 (13.48; 26.81) | 0.64 (0.13; 1.14) | 2.78 (1.16; 5.41) | 2.77 (1.15; 5.39) | -0.29 (-1.37; 0.79) | 0.25 (0.19; 0.32) | 0.14 (0.10; 0.20) | -41.21 (-41.54; -40.88) | 0.68 (0.47; 0.99) | 0.40 (0.26; 0.60) | -40.68 (-41.15; -40.22) | 0.00 (0.00; 0.00) | 0.00 (0.00; 0.00) | -41.17 (-42.15; -40.18) |
| **El Salvador** | 7.31 (5.53; 9.26) | 7.24 (5.48; 9.18) | -0.41 (-0.77; -0.05) | 18.87 (13.27; 26.71) | 18.93 (13.35; 26.76) | 0.92 (0.41; 1.42) | 2.81 (1.19; 5.57) | 2.78 (1.18; 5.52) | -0.40 (-1.50; 0.70) | 0.46 (0.35; 0.59) | 0.21 (0.13; 0.33) | -53.61 (-54; -53.23) | 1.21 (0.85; 1.73) | 0.58 (0.31; 0.94) | -52.04 (-52.50; -51.59) | 0.00 (0.00; 0.00) | 0.00 (0.00; 0.00) | -53.54 (-54.44; -52.64) |
| **Guatemala** | 7.29 (5.49; 9.19) | 7.23 (5.45; 9.11) | -0.48 (-0.84; -0.12) | 18.86 (13.24; 26.82) | 18.81 (13.19; 26.75) | 0.28 (-0.23; 0.79) | 2.81 (1.19; 5.60) | 2.79 (1.18; 5.56) | -0.47 (-1.57; 0.64) | 1.25 (0.94; 1.58) | 0.91 (0.60; 1.31) | -25.91 (-26.30; -25.52) | 3.31 (2.31; 4.76) | 2.45 (1.52; 3.77) | -25.26 (-25.75; -24.77) | 0.00 (0.00; 0.00) | 0.00 (0.00; 0.00) | -25.76 (-26.79; -24.73) |
| **Honduras** | 7.35 (5.63; 9.28) | 7.25 (5.56; 9.16) | -0.44 (-0.79; -0.09) | 16.83 (13.09; 21.46) | 16.80 (13.00; 21.46) | 0.61 (0.25; 0.96) | 2.83 (1.20; 5.60) | 2.79 (1.19; 5.52) | -0.43 (-1.53; 0.66) | 0.83 (0.63; 1.04) | 0.70 (0.47; 0.99) | -15.61 (-16.03; -15.19) | 1.91 (1.48; 2.45) | 1.64 (1.10; 2.33) | -14.02 (-14.45; -13.59) | 0.00 (0.00; 0.00) | 0.00 (0.00; 0.00) | -15.70 (-16.75; -14.64) |
| **Mexico** | 7.60 (6.27; 9.05) | 7.54 (6.23; 8.98) | -0.33 (-0.59; -0.08) | 19.00 (14.51; 24.46) | 18.94 (14.44; 24.44) | 0.26 (-0.11; 0.63) | 2.92 (1.27; 5.53) | 2.90 (1.26; 5.48) | -0.33 (-1.36; 0.70) | 8.25 (6.77; 9.87) | 5.80 (4.41; 7.60) | -28.52 (-28.80; -28.24) | 21.21 (16.13; 27.79) | 15.06 (10.92; 21.05) | -28.04 (-28.42; -27.67) | 0.00 (0.00; 0.01) | 0.00 (0.00; 0.00) | -28.47 (-29.38; -27.55) |
| **Nicaragua** | 6.45 (4.90; 8.09) | 6.38 (4.85; 8.01) | -0.38 (-0.73; -0.03) | 14.49 (9.94; 19.20) | 14.50 (9.92; 19.21) | 0.69 (0.24; 1.15) | 2.47 (1.09; 5.20) | 2.44 (1.08; 5.14) | -0.38 (-1.55; 0.79) | 0.44 (0.34; 0.55) | 0.30 (0.19; 0.44) | -31.73 (-32.16; -31.30) | 1.00 (0.68; 1.32) | 0.68 (0.40; 1.04) | -30.78 (-31.27; -30.30) | 0.00 (0.00; 0.00) | 0.00 (0.00; 0.00) | -31.60 (-32.62; -30.58) |
| **Panama** | 6.30 (4.80; 7.88) | 6.26 (4.77; 7.82) | -0.35 (-0.69; 0.00) | 14.50 (9.81; 19.45) | 14.61 (9.92; 19.59) | 1.14 (0.67; 1.61) | 2.41 (1.05; 5.11) | 2.40 (1.05; 5.07) | -0.35 (-1.53; 0.84) | 0.25 (0.19; 0.32) | 0.24 (0.17; 0.33) | -5.04 (-5.47; -4.61) | 0.59 (0.39; 0.79) | 0.56 (0.35; 0.82) | -3.99 (-4.52; -3.45) | 0.00 (0.00; 0.00) | 0.00 (0.00; 0.00) | -4.91 (-6.10; -3.73) |
| **Venezuela (Bolivarian Republic of)** | 6.35 (4.88; 7.89) | 6.32 (4.86; 7.85) | 0.28 (-0.06; 0.61) | 14.48 (9.68; 19.41) | 14.28 (9.58; 18.99) | -0.47 (-0.94; 0.00) | 2.44 (1.06; 5.06) | 2.43 (1.06; 5.04) | 0.28 (-0.89; 1.44) | 1.43 (1.10; 1.78) | 0.94 (0.59; 1.40) | -33.91 (-34.32; -33.51) | 3.28 (2.19; 4.39) | 2.15 (1.22; 3.31) | -33.98 (-34.46; -33.50) | 0.00 (0.00; 0.00) | 0.00 (0.00; 0.00) | -33.97 (-34.99; -32.94) |
| **Tropical Latin America** | 7.71 (6.08; 9.40) | 7.65 (6.04; 9.34) | 0.11 (-0.20; 0.41) | 20.35 (14.54; 29.16) | 20.15 (14.38; 29.12) | -0.24 (-0.75; 0.28) | 2.94 (1.23; 5.97) | 2.92 (1.22; 5.91) | 0.12 (-1.02; 1.26) | 13.36 (10.52; 16.33) | 10.73 (8.12; 14.09) | -19.91 (-20.25; -19.57) | 35.47 (25.34; 51.29) | 29.09 (19.62; 43.00) | -18.30 (-18.80; -17.81) | 0.01 (0.00; 0.01) | 0.00 (0.00; 0.01) | -19.94 (-20.97; -18.91) |
| **Brazil** | 7.68 (6.01; 9.34) | 7.63 (6.00; 9.29) | 0.17 (-0.13; 0.48) | 20.41 (14.57; 29.29) | 20.20 (14.40; 29.15) | -0.31 (-0.83; 0.20) | 2.93 (1.22; 5.91) | 2.91 (1.21; 5.86) | 0.19 (-0.94; 1.32) | 12.79 (10.01; 15.58) | 10.33 (7.76; 13.68) | -19.50 (-19.85; -19.15) | 34.16 (24.31; 49.30) | 28.16 (18.99; 41.64) | -17.94 (-18.43; -17.45) | 0.00 (0.00; 0.01) | 0.00 (0.00; 0.01) | -19.54 (-20.57; -18.50) |
| **Paraguay** | 8.35 (6.62; 10.13) | 8.26 (6.54; 10.02) | -0.36 (-0.66; -0.07) | 18.83 (13.49; 26.24) | 18.79 (13.44; 26.05) | 0.46 (-0.02; 0.94) | 3.19 (1.36; 6.66) | 3.16 (1.34; 6.59) | -0.35 (-1.53; 0.82) | 0.57 (0.45; 0.70) | 0.40 (0.25; 0.58) | -29.22 (-29.62; -28.83) | 1.32 (0.94; 1.88) | 0.94 (0.54; 1.57) | -27.93 (-28.49; -27.37) | 0.00 (0.00; 0.00) | 0.00 (0.00; 0.00) | -29.04 (-30.05; -28.04) |
| **North Africa and Middle East** | 7.45 (6.24; 8.67) | 7.29 (6.07; 8.61) | -1.45 (-1.69; -1.21) | 16.17 (12.51; 20.08) | 15.80 (11.81; 19.71) | -1.58 (-1.92; -1.24) | 2.85 (1.31; 5.52) | 2.79 (1.27; 5.54) | -1.43 (-2.48; -0.38) | 47.66 (39.92; 55.59) | 44.21 (32.94; 56.42) | -6.68 (-6.98; -6.38) | 104.32 (80.20; 129.93) | 97.00 (67.70; 127.93) | -6.78 (-7.16; -6.40) | 0.02 (0.01; 0.04) | 0.02 (0.01; 0.03) | -6.65 (-7.68; -5.62) |
| **North Africa and Middle East** | 7.45 (6.24; 8.67) | 7.29 (6.07; 8.61) | -1.45 (-1.69; -1.21) | 16.17 (12.51; 20.08) | 15.80 (11.81; 19.71) | -1.58 (-1.92; -1.24) | 2.85 (1.31; 5.52) | 2.79 (1.27; 5.54) | -1.43 (-2.48; -0.38) | 47.66 (39.92; 55.59) | 44.21 (32.94; 56.42) | -6.68 (-6.98; -6.38) | 104.32 (80.20; 129.93) | 97.00 (67.70; 127.93) | -6.78 (-7.16; -6.40) | 0.02 (0.01; 0.04) | 0.02 (0.01; 0.03) | -6.65 (-7.68; -5.62) |
| **Afghanistan** | 7.35 (5.78; 8.98) | 7.20 (5.66; 8.80) | -0.64 (-0.95; -0.34) | 14.76 (10.18; 19.13) | 14.66 (10.01; 19.12) | 0.48 (0.04; 0.92) | 2.81 (1.27; 5.93) | 2.76 (1.24; 5.81) | -0.64 (-1.81; 0.52) | 3.73 (2.95; 4.55) | 6.27 (3.68; 9.08) | 63.71 (62.91; 64.51) | 7.38 (5.17; 9.55) | 12.78 (6.91; 19.59) | 68.53 (67.59; 69.48) | 0.00 (0.00; 0.00) | 0.00 (0.00; 0.01) | 63.90 (62.17; 65.64) |
| **Algeria** | 7.10 (5.52; 8.78) | 7.03 (5.46; 8.68) | -0.47 (-0.79; -0.15) | 14.71 (10.18; 19.17) | 14.65 (10.11; 19.03) | 0.24 (-0.19; 0.68) | 2.72 (1.22; 5.59) | 2.69 (1.20; 5.53) | -0.47 (-1.60; 0.67) | 3.40 (2.64; 4.21) | 2.58 (1.78; 3.53) | -24.54 (-24.91; -24.18) | 7.04 (4.88; 9.18) | 5.40 (3.45; 7.95) | -23.90 (-24.36; -23.44) | 0.00 (0.00; 0.00) | 0.00 (0.00; 0.00) | -24.50 (-25.56; -23.45) |
| **Bahrain** | 7.53 (5.75; 9.47) | 7.47 (5.70; 9.39) | -0.34 (-0.69; 0.00) | 19.00 (13.52; 26.79) | 18.92 (13.50; 26.78) | 0.18 (-0.32; 0.67) | 2.88 (1.19; 5.74) | 2.86 (1.18; 5.69) | -0.34 (-1.45; 0.78) | 0.08 (0.06; 0.10) | 0.07 (0.05; 0.10) | -5.87 (-6.27; -5.46) | 0.20 (0.14; 0.28) | 0.19 (0.12; 0.27) | -5.89 (-6.42; -5.35) | 0.00 (0.00; 0.00) | 0.00 (0.00; 0.00) | -5.92 (-7.03; -4.81) |
| **Egypt** | 7.13 (5.61; 8.90) | 7.04 (5.54; 8.79) | -0.50 (-0.83; -0.17) | 14.68 (9.67; 19.54) | 14.77 (9.76; 19.70) | 1.32 (0.84; 1.79) | 2.74 (1.21; 5.65) | 2.70 (1.19; 5.57) | -0.50 (-1.64; 0.65) | 9.37 (7.37; 11.70) | 10.72 (7.51; 14.69) | 13.78 (13.33; 14.24) | 19.27 (12.67; 25.63) | 22.58 (13.96; 32.64) | 16.09 (15.49; 16.69) | 0.00 (0.00; 0.01) | 0.00 (0.00; 0.01) | 13.66 (12.41; 14.90) |
| **Iran (Islamic Republic of)** | 8.81 (7.38; 10.39) | 8.80 (7.37; 10.38) | -0.36 (-0.60; -0.12) | 20.26 (15.08; 28.79) | 19.99 (14.85; 28.46) | -0.86 (-1.34; -0.38) | 3.38 (1.45; 6.54) | 3.38 (1.46; 6.53) | -0.36 (-1.42; 0.70) | 6.29 (5.28; 7.49) | 4.01 (2.88; 5.35) | -34.40 (-34.69; -34.10) | 14.86 (10.96; 21.21) | 9.41 (6.15; 14.22) | -35.57 (-36.01; -35.13) | 0.00 (0.00; 0.00) | 0.00 (0.00; 0.00) | -34.33 (-35.27; -33.40) |
| **Iraq** | 7.11 (5.44; 8.78) | 7.04 (5.38; 8.69) | -0.43 (-0.76; -0.09) | 14.76 (9.96; 19.37) | 14.64 (9.84; 19.30) | -0.11 (-0.57; 0.34) | 2.73 (1.21; 5.69) | 2.70 (1.20; 5.64) | -0.42 (-1.58; 0.73) | 3.28 (2.53; 4.06) | 3.28 (2.12; 4.91) | 1.72 (1.16; 2.28) | 6.91 (4.58; 9.09) | 6.90 (3.90; 10.20) | 1.47 (0.85; 2.09) | 0.00 (0.00; 0.00) | 0.00 (0.00; 0.00) | 1.83 (0.57; 3.08) |
| **Jordan** | 7.41 (5.57; 9.29) | 7.35 (5.52; 9.22) | -0.29 (-0.65; 0.06) | 18.82 (13.20; 27.22) | 19.36 (13.51; 27.72) | 3.34 (2.81; 3.87) | 2.83 (1.20; 5.58) | 2.81 (1.19; 5.54) | -0.29 (-1.38; 0.81) | 0.90 (0.68; 1.14) | 1.17 (0.83; 1.59) | 31.42 (30.91; 31.92) | 2.37 (1.64; 3.55) | 3.16 (2.06; 4.78) | 34.57 (33.86; 35.27) | 0.00 (0.00; 0.00) | 0.00 (0.00; 0.00) | 31.35 (30.05; 32.66) |
| **Kuwait** | 7.34 (5.66; 9.15) | 7.30 (5.63; 9.11) | -0.16 (-0.50; 0.17) | 18.87 (13.11; 25.76) | 19.54 (13.62; 26.64) | 3.92 (3.44; 4.41) | 2.81 (1.19; 5.54) | 2.80 (1.19; 5.51) | -0.16 (-1.25; 0.93) | 0.21 (0.16; 0.27) | 0.20 (0.14; 0.28) | -6.48 (-6.91; -6.06) | 0.56 (0.39; 0.78) | 0.55 (0.37; 0.81) | -2.92 (-3.47; -2.36) | 0.00 (0.00; 0.00) | 0.00 (0.00; 0.00) | -6.55 (-7.65; -5.44) |
| **Lebanon** | 7.11 (5.55; 8.75) | 7.04 (5.50; 8.66) | -0.27 (-0.59; 0.05) | 14.73 (9.83; 19.45) | 14.66 (9.77; 19.41) | 0.32 (-0.14; 0.78) | 2.73 (1.22; 5.72) | 2.70 (1.20; 5.67) | -0.27 (-1.43; 0.90) | 0.31 (0.24; 0.38) | 0.19 (0.13; 0.28) | -36.54 (-36.91; -36.17) | 0.65 (0.43; 0.87) | 0.41 (0.24; 0.60) | -36.52 (-36.99; -36.06) | 0.00 (0.00; 0.00) | 0.00 (0.00; 0.00) | -36.61 (-37.60; -35.62) |
| **Libya** | 7.14 (5.58; 8.85) | 7.10 (5.54; 8.79) | -0.27 (-0.60; 0.05) | 14.69 (9.95; 19.44) | 14.65 (9.96; 19.33) | 0.23 (-0.22; 0.69) | 2.74 (1.19; 5.85) | 2.72 (1.19; 5.81) | -0.27 (-1.47; 0.93) | 0.35 (0.27; 0.43) | 0.19 (0.13; 0.28) | -41.77 (-42.14; -41.40) | 0.73 (0.48; 0.97) | 0.41 (0.23; 0.62) | -41.85 (-42.32; -41.37) | 0.00 (0.00; 0.00) | 0.00 (0.00; 0.00) | -41.85 (-42.85; -40.85) |
| **Morocco** | 7.15 (5.63; 9.01) | 7.06 (5.56; 8.89) | -0.54 (-0.87; -0.20) | 14.67 (9.92; 19.26) | 14.58 (9.99; 19.06) | 0.12 (-0.32; 0.57) | 2.74 (1.21; 5.75) | 2.71 (1.20; 5.67) | -0.53 (-1.70; 0.63) | 2.40 (1.90; 3.03) | 1.48 (1.01; 2.07) | -37.82 (-38.15; -37.48) | 4.96 (3.32; 6.52) | 3.12 (1.89; 4.61) | -36.76 (-37.18; -36.34) | 0.00 (0.00; 0.00) | 0.00 (0.00; 0.00) | -37.81 (-38.82; -36.80) |
| **Oman** | 7.76 (5.92; 9.86) | 7.70 (5.87; 9.78) | -0.33 (-0.69; 0.03) | 19.13 (13.65; 28.22) | 20.22 (14.46; 29.53) | 6.17 (5.62; 6.72) | 2.97 (1.22; 5.93) | 2.95 (1.21; 5.89) | -0.32 (-1.44; 0.80) | 0.35 (0.26; 0.44) | 0.31 (0.22; 0.43) | -9.23 (-9.63; -8.82) | 0.85 (0.61; 1.22) | 0.84 (0.53; 1.21) | -3.08 (-3.62; -2.54) | 0.00 (0.00; 0.00) | 0.00 (0.00; 0.00) | -9.05 (-10.17; -7.92) |
| **Palestine** | 6.67 (5.15; 8.27) | 6.61 (5.10; 8.18) | -0.35 (-0.68; -0.02) | 14.79 (10.02; 19.38) | 14.49 (9.79; 18.96) | -1.29 (-1.73; -0.85) | 2.56 (1.12; 5.44) | 2.54 (1.10; 5.38) | -0.35 (-1.54; 0.84) | 0.43 (0.33; 0.53) | 0.39 (0.27; 0.51) | -9.29 (-9.66; -8.92) | 0.96 (0.65; 1.26) | 0.86 (0.54; 1.20) | -10.18 (-10.65; -9.70) | 0.00 (0.00; 0.00) | 0.00 (0.00; 0.00) | -9.37 (-10.47; -8.26) |
| **Qatar** | 8.69 (7.05; 10.36) | 8.56 (6.95; 10.21) | -0.18 (-0.45; 0.09) | 19.55 (13.96; 26.98) | 18.75 (13.43; 26.08) | -2.98 (-3.45; -2.51) | 3.32 (1.46; 6.80) | 3.28 (1.44; 6.69) | -0.18 (-1.31; 0.96) | 0.15 (0.12; 0.18) | 0.25 (0.19; 0.32) | 61.11 (60.64; 61.58) | 0.34 (0.24; 0.46) | 0.56 (0.36; 0.82) | 59.02 (58.29; 59.75) | 0.00 (0.00; 0.00) | 0.00 (0.00; 0.00) | 60.89 (59.40; 62.38) |
| **Saudi Arabia** | 8.29 (6.89; 9.71) | 8.22 (6.83; 9.63) | -0.22 (-0.46; 0.02) | 17.28 (11.88; 24.75) | 17.17 (11.87; 24.51) | -0.04 (-0.56; 0.48) | 3.18 (1.36; 6.46) | 3.15 (1.35; 6.41) | -0.22 (-1.35; 0.91) | 2.19 (1.82; 2.56) | 1.39 (0.95; 1.85) | -36.29 (-36.59; -35.98) | 4.62 (3.14; 6.69) | 2.96 (1.70; 4.70) | -35.60 (-36.13; -35.06) | 0.00 (0.00; 0.00) | 0.00 (0.00; 0.00) | -36.25 (-37.17; -35.33) |
| **Sudan** | 6.73 (5.19; 8.48) | 6.64 (5.12; 8.37) | -0.52 (-0.86; -0.17) | 14.65 (10.04; 19.25) | 15.66 (10.55; 20.71) | 7.61 (7.14; 8.08) | 2.57 (1.14; 5.39) | 2.54 (1.12; 5.31) | -0.51 (-1.68; 0.65) | 3.86 (2.97; 4.87) | 3.58 (2.33; 5.20) | -7.36 (-7.83; -6.89) | 8.43 (5.76; 11.08) | 8.56 (5.01; 12.56) | 1.11 (0.53; 1.68) | 0.00 (0.00; 0.00) | 0.00 (0.00; 0.00) | -7.39 (-8.52; -6.26) |
| **Syrian Arab Republic** | 7.07 (5.45; 8.63) | 7.00 (5.39; 8.54) | -0.31 (-0.63; 0.01) | 15.03 (10.18; 19.45) | 14.95 (10.19; 19.29) | 0.22 (-0.22; 0.65) | 2.72 (1.17; 5.73) | 2.69 (1.16; 5.68) | -0.31 (-1.49; 0.88) | 0.77 (0.59; 0.94) | 0.70 (0.43; 1.00) | -7.37 (-7.84; -6.91) | 1.69 (1.12; 2.20) | 1.53 (0.86; 2.34) | -6.22 (-6.79; -5.64) | 0.00 (0.00; 0.00) | 0.00 (0.00; 0.00) | -7.39 (-8.59; -6.20) |
| **Tunisia** | 7.69 (5.85; 9.69) | 7.62 (5.81; 9.62) | -0.42 (-0.77; -0.06) | 19.00 (13.44; 27.32) | 18.95 (13.44; 27.11) | 0.29 (-0.22; 0.81) | 2.95 (1.22; 6.01) | 2.92 (1.21; 5.97) | -0.41 (-1.56; 0.74) | 0.74 (0.56; 0.94) | 0.43 (0.28; 0.62) | -41.44 (-41.79; -41.08) | 1.87 (1.32; 2.70) | 1.09 (0.69; 1.73) | -41.07 (-41.56; -40.58) | 0.00 (0.00; 0.00) | 0.00 (0.00; 0.00) | -41.51 (-42.48; -40.55) |
| **Türkiye** | 7.62 (5.91; 9.56) | 7.57 (5.88; 9.50) | -0.38 (-0.72; -0.04) | 19.33 (13.68; 27.97) | 18.96 (13.43; 27.58) | -1.36 (-1.88; -0.83) | 2.91 (1.23; 5.85) | 2.89 (1.23; 5.82) | -0.37 (-1.49; 0.75) | 4.86 (3.78; 6.14) | 2.85 (2.05; 3.78) | -39.72 (-40.04; -39.40) | 12.76 (8.88; 18.77) | 7.40 (4.88; 10.92) | -40.66 (-41.13; -40.19) | 0.00 (0.00; 0.00) | 0.00 (0.00; 0.00) | -39.75 (-40.67; -38.83) |
| **United Arab Emirates** | 8.35 (7.13; 9.65) | 8.35 (7.13; 9.64) | -0.11 (-0.32; 0.10) | 14.34 (10.62; 19.00) | 14.24 (10.55; 18.86) | -0.48 (-0.89; -0.07) | 3.20 (1.44; 6.67) | 3.20 (1.44; 6.67) | -0.11 (-1.27; 1.04) | 0.41 (0.35; 0.47) | 0.71 (0.53; 0.88) | 74.53 (74.05; 75.02) | 0.70 (0.52; 0.94) | 1.22 (0.83; 1.71) | 73.35 (72.63; 74.06) | 0.00 (0.00; 0.00) | 0.00 (0.00; 0.00) | 74.66 (73.06; 76.25) |
| **Yemen** | 7.39 (5.83; 9.07) | 7.27 (5.74; 8.93) | -0.59 (-0.90; -0.28) | 14.78 (10.06; 19.34) | 14.74 (9.98; 19.31) | 0.63 (0.18; 1.08) | 2.81 (1.24; 5.77) | 2.76 (1.23; 5.69) | -0.59 (-1.73; 0.55) | 3.52 (2.77; 4.33) | 3.39 (2.08; 5.00) | -3.99 (-4.49; -3.50) | 7.08 (4.81; 9.28) | 6.95 (4.03; 9.99) | -2.07 (-2.61; -1.52) | 0.00 (0.00; 0.00) | 0.00 (0.00; 0.00) | -3.86 (-5.00; -2.71) |
| **South Asia** | 7.74 (6.45; 9.00) | 7.64 (6.38; 8.87) | -0.67 (-0.90; -0.44) | 17.49 (13.71; 22.37) | 17.53 (13.74; 22.38) | 0.92 (0.57; 1.27) | 2.96 (1.33; 5.65) | 2.92 (1.30; 5.62) | -0.66 (-1.70; 0.38) | 131.97 (110.00; 152.94) | 82.10 (60.96; 108.54) | -36.80 (-37.07; -36.54) | 306.23 (237.84; 400.19) | 196.26 (135.68; 272.53) | -34.99 (-35.34; -34.63) | 0.05 (0.02; 0.10) | 0.03 (0.01; 0.06) | -36.81 (-37.71; -35.90) |
| **South Asia** | 7.74 (6.45; 9.00) | 7.64 (6.38; 8.87) | -0.67 (-0.90; -0.44) | 17.49 (13.71; 22.37) | 17.53 (13.74; 22.38) | 0.92 (0.57; 1.27) | 2.96 (1.33; 5.65) | 2.92 (1.30; 5.62) | -0.66 (-1.70; 0.38) | 131.97 (110.00; 152.94) | 82.10 (60.96; 108.54) | -36.80 (-37.07; -36.54) | 306.23 (237.84; 400.19) | 196.26 (135.68; 272.53) | -34.99 (-35.34; -34.63) | 0.05 (0.02; 0.10) | 0.03 (0.01; 0.06) | -36.81 (-37.71; -35.90) |
| **Bangladesh** | 8.37 (6.58; 10.22) | 8.27 (6.49; 10.10) | -0.57 (-0.87; -0.26) | 20.04 (14.42; 29.19) | 20.03 (14.42; 29.38) | 0.64 (0.11; 1.16) | 3.21 (1.40; 6.48) | 3.17 (1.38; 6.40) | -0.55 (-1.67; 0.56) | 13.00 (10.18; 15.92) | 6.73 (4.14; 9.79) | -47.39 (-47.74; -47.03) | 32.13 (22.77; 48.01) | 17.27 (9.69; 28.47) | -45.56 (-46.08; -45.05) | 0.00 (0.00; 0.01) | 0.00 (0.00; 0.01) | -47.35 (-48.24; -46.45) |
| **Bhutan** | 6.12 (4.55; 7.58) | 6.03 (4.47; 7.45) | -0.66 (-1.01; -0.31) | 13.84 (9.22; 18.47) | 13.89 (9.18; 18.61) | 1.21 (0.73; 1.69) | 2.33 (1.02; 4.88) | 2.29 (1.01; 4.80) | -0.66 (-1.83; 0.50) | 0.04 (0.03; 0.05) | 0.02 (0.01; 0.03) | -42.22 (-42.56; -41.88) | 0.09 (0.06; 0.12) | 0.05 (0.03; 0.08) | -39.82 (-40.27; -39.36) | 0.00 (0.00; 0.00) | 0.00 (0.00; 0.00) | -42.12 (-43.12; -41.12) |
| **India** | 7.70 (6.40; 8.98) | 7.63 (6.36; 8.91) | -0.36 (-0.59; -0.12) | 17.26 (13.56; 22.10) | 17.08 (13.41; 21.90) | -0.36 (-0.71; -0.01) | 2.94 (1.31; 5.63) | 2.91 (1.29; 5.61) | -0.36 (-1.41; 0.68) | 93.98 (78.11; 109.67) | 57.79 (41.70; 78.19) | -37.26 (-37.55; -36.96) | 217.81 (168.26; 284.51) | 135.33 (87.55; 190.68) | -36.64 (-37.02; -36.27) | 0.04 (0.02; 0.07) | 0.02 (0.01; 0.04) | -37.28 (-38.20; -36.36) |
| **Nepal** | 7.86 (6.60; 8.91) | 7.74 (6.50; 8.76) | -0.56 (-0.77; -0.35) | 14.63 (10.48; 19.32) | 13.86 (10.09; 18.21) | -4.22 (-4.63; -3.81) | 3.01 (1.38; 6.10) | 2.97 (1.36; 6.00) | -0.56 (-1.66; 0.54) | 2.46 (2.06; 2.78) | 1.23 (0.76; 1.79) | -49.75 (-50.03; -49.46) | 4.59 (3.29; 6.07) | 2.25 (1.28; 3.49) | -50.68 (-51.09; -50.27) | 0.00 (0.00; 0.00) | 0.00 (0.00; 0.00) | -49.67 (-50.58; -48.76) |
| **Pakistan** | 7.54 (6.27; 8.78) | 7.43 (6.17; 8.65) | -0.61 (-0.84; -0.37) | 17.28 (13.26; 21.53) | 18.37 (13.96; 23.13) | 6.94 (6.58; 7.30) | 2.88 (1.30; 5.77) | 2.84 (1.28; 5.68) | -0.60 (-1.69; 0.49) | 22.50 (18.69; 26.19) | 16.33 (11.00; 22.48) | -27.52 (-27.84; -27.20) | 51.61 (39.59; 64.31) | 41.37 (26.11; 59.31) | -20.32 (-20.73; -19.92) | 0.01 (0.00; 0.02) | 0.01 (0.00; 0.01) | -27.49 (-28.51; -26.47) |
| **Southeast Asia, East Asia, and Oceania** | 8.57 (7.14; 10.10) | 8.61 (7.15; 10.15) | 0.04 (-0.21; 0.29) | 19.86 (15.01; 27.56) | 20.14 (15.19; 28.07) | 1.84 (1.39; 2.30) | 3.29 (1.43; 6.52) | 3.30 (1.44; 6.55) | 0.01 (-1.08; 1.11) | 134.17 (111.70; 159.09) | 80.21 (64.12; 98.33) | -38.40 (-38.62; -38.17) | 317.28 (238.31; 444.04) | 195.94 (140.15; 274.28) | -37.35 (-37.75; -36.96) | 0.05 (0.02; 0.10) | 0.03 (0.01; 0.06) | -38.38 (-39.32; -37.44) |
| **East Asia** | 8.37 (6.97; 9.87) | 8.42 (7.00; 9.96) | -0.52 (-0.77; -0.28) | 19.54 (14.78; 26.42) | 19.78 (15.01; 26.77) | 1.58 (1.15; 2.00) | 3.21 (1.39; 6.37) | 3.23 (1.40; 6.41) | -0.52 (-1.61; 0.57) | 80.09 (66.47; 94.91) | 37.19 (29.08; 46.41) | -51.35 (-51.58; -51.13) | 191.23 (143.73; 259.78) | 92.65 (64.56; 131.26) | -50.49 (-50.85; -50.12) | 0.03 (0.01; 0.06) | 0.01 (0.01; 0.03) | -51.26 (-52.18; -50.34) |
| **China** | 8.37 (6.95; 9.86) | 8.43 (7.01; 9.95) | -0.50 (-0.74; -0.26) | 19.57 (14.79; 26.39) | 19.84 (15.05; 26.79) | 1.75 (1.32; 2.17) | 3.21 (1.39; 6.37) | 3.23 (1.40; 6.42) | -0.50 (-1.59; 0.60) | 77.86 (64.69; 92.19) | 35.94 (27.89; 44.77) | -51.61 (-51.83; -51.38) | 186.22 (139.85; 252.24) | 89.86 (62.63; 126.55) | -50.68 (-51.05; -50.32) | 0.03 (0.01; 0.06) | 0.01 (0.01; 0.03) | -51.51 (-52.44; -50.59) |
| **Democratic People's Republic of Korea** | 7.85 (5.96; 9.87) | 7.78 (5.91; 9.79) | -0.22 (-0.57; 0.14) | 18.79 (13.05; 26.91) | 18.67 (12.92; 26.80) | 0.12 (-0.41; 0.64) | 3.01 (1.22; 5.88) | 2.98 (1.21; 5.83) | -0.21 (-1.30; 0.88) | 1.26 (0.96; 1.60) | 0.76 (0.52; 1.05) | -38.60 (-38.95; -38.25) | 3.10 (2.13; 4.55) | 1.90 (1.18; 2.99) | -37.70 (-38.21; -37.18) | 0.00 (0.00; 0.00) | 0.00 (0.00; 0.00) | -38.56 (-39.48; -37.64) |
| **Taiwan (Province of China)** | 9.32 (7.37; 11.47) | 9.28 (7.34; 11.42) | -0.26 (-0.57; 0.05) | 17.96 (11.05; 28.17) | 16.21 (9.95; 25.77) | -8.82 (-9.47; -8.16) | 3.57 (1.49; 7.45) | 3.56 (1.49; 7.43) | -0.25 (-1.43; 0.93) | 0.98 (0.77; 1.21) | 0.49 (0.37; 0.63) | -48.34 (-48.62; -48.06) | 1.91 (1.16; 3.02) | 0.90 (0.52; 1.48) | -52.07 (-52.66; -51.47) | 0.00 (0.00; 0.00) | 0.00 (0.00; 0.00) | -48.33 (-49.25; -47.41) |
| **Oceania** | 8.91 (7.28; 10.65) | 8.75 (7.13; 10.48) | -0.49 (-0.76; -0.22) | 20.14 (14.58; 28.58) | 20.20 (14.66; 28.87) | 1.20 (0.70; 1.70) | 3.41 (1.47; 6.87) | 3.34 (1.44; 6.73) | -0.50 (-1.62; 0.62) | 1.60 (1.31; 1.90) | 2.51 (1.83; 3.28) | 52.53 (52.04; 53.03) | 3.53 (2.58; 4.94) | 5.78 (3.93; 8.26) | 58.89 (58.19; 59.59) | 0.00 (0.00; 0.00) | 0.00 (0.00; 0.00) | 52.43 (50.92; 53.95) |
| **American Samoa** | 7.84 (6.28; 9.56) | 7.84 (6.28; 9.53) | -0.26 (-0.55; 0.03) | 15.91 (10.70; 20.68) | 15.94 (10.72; 20.73) | 0.49 (0.05; 0.93) | 3.01 (1.36; 6.35) | 3.01 (1.36; 6.33) | -0.26 (-1.43; 0.91) | 0.00 (0.00; 0.00) | 0.00 (0.00; 0.00) | 3.57 (3.19; 3.95) | 0.01 (0.00; 0.01) | 0.01 (0.00; 0.01) | 1.59 (1.07; 2.11) | 0.00 (0.00; 0.00) | 0.00 (0.00; 0.00) | 3.67 (2.49; 4.86) |
| **Cook Islands** | 8.78 (7.14; 10.52) | 8.70 (7.07; 10.42) | -0.29 (-0.57; -0.02) | 18.12 (14.13; 23.19) | 18.13 (14.09; 23.17) | 0.70 (0.34; 1.05) | 3.38 (1.42; 6.58) | 3.35 (1.41; 6.52) | -0.29 (-1.36; 0.79) | 0.00 (0.00; 0.00) | 0.00 (0.00; 0.00) | -28.99 (-29.32; -28.67) | 0.00 (0.00; 0.00) | 0.00 (0.00; 0.00) | -28.52 (-28.91; -28.13) | 0.00 (0.00; 0.00) | 0.00 (0.00; 0.00) | -29.04 (-30.02; -28.05) |
| **Fiji** | 8.81 (7.12; 10.57) | 8.71 (7.04; 10.45) | -0.33 (-0.61; -0.05) | 20.15 (14.69; 28.28) | 20.60 (14.89; 28.90) | 2.89 (2.41; 3.38) | 3.37 (1.46; 6.63) | 3.33 (1.44; 6.56) | -0.32 (-1.41; 0.76) | 0.08 (0.07; 0.10) | 0.06 (0.04; 0.08) | -27.91 (-28.25; -27.57) | 0.19 (0.14; 0.27) | 0.14 (0.09; 0.22) | -25.24 (-25.75; -24.73) | 0.00 (0.00; 0.00) | 0.00 (0.00; 0.00) | -27.78 (-28.77; -26.79) |
| **Guam** | 8.71 (6.97; 10.47) | 8.60 (6.88; 10.34) | -0.13 (-0.41; 0.16) | 18.11 (13.94; 22.79) | 18.02 (13.87; 22.72) | 0.43 (0.08; 0.78) | 3.34 (1.44; 6.51) | 3.30 (1.43; 6.44) | -0.13 (-1.20; 0.95) | 0.01 (0.01; 0.01) | 0.01 (0.01; 0.01) | -22.60 (-22.94; -22.26) | 0.02 (0.02; 0.03) | 0.02 (0.01; 0.02) | -21.28 (-21.65; -20.90) | 0.00 (0.00; 0.00) | 0.00 (0.00; 0.00) | -22.45 (-23.43; -21.47) |
| **Kiribati** | 9.01 (7.45; 10.81) | 8.90 (7.36; 10.68) | -0.34 (-0.61; -0.08) | 20.17 (14.61; 29.29) | 20.14 (14.61; 29.36) | 0.62 (0.10; 1.14) | 3.46 (1.48; 6.74) | 3.42 (1.47; 6.66) | -0.34 (-1.41; 0.73) | 0.01 (0.01; 0.02) | 0.01 (0.01; 0.01) | -16.68 (-17.04; -16.31) | 0.03 (0.02; 0.04) | 0.03 (0.02; 0.04) | -15.19 (-15.73; -14.66) | 0.00 (0.00; 0.00) | 0.00 (0.00; 0.00) | -16.67 (-17.74; -15.61) |
| **Marshall Islands** | 8.95 (7.34; 10.83) | 8.84 (7.25; 10.71) | -0.36 (-0.64; -0.09) | 20.18 (14.52; 28.84) | 20.06 (14.36; 28.59) | 0.17 (-0.33; 0.67) | 3.43 (1.47; 6.72) | 3.39 (1.46; 6.64) | -0.35 (-1.43; 0.72) | 0.01 (0.00; 0.01) | 0.01 (0.00; 0.01) | -4.11 (-4.50; -3.72) | 0.01 (0.01; 0.02) | 0.01 (0.01; 0.02) | -3.60 (-4.19; -3.02) | 0.00 (0.00; 0.00) | 0.00 (0.00; 0.00) | -4.15 (-5.29; -3.00) |
| **Micronesia (Federated States of)** | 8.99 (7.33; 10.74) | 8.90 (7.26; 10.64) | -0.33 (-0.60; -0.06) | 20.02 (14.28; 29.56) | 19.96 (14.13; 29.33) | 0.43 (-0.11; 0.97) | 3.45 (1.44; 6.79) | 3.42 (1.43; 6.72) | -0.32 (-1.41; 0.77) | 0.01 (0.01; 0.01) | 0.01 (0.00; 0.01) | -26.29 (-26.67; -25.92) | 0.02 (0.01; 0.03) | 0.02 (0.01; 0.02) | -26.31 (-26.84; -25.78) | 0.00 (0.00; 0.00) | 0.00 (0.00; 0.00) | -26.37 (-27.34; -25.40) |
| **Nauru** | 8.82 (7.08; 10.61) | 8.70 (6.98; 10.47) | -0.34 (-0.62; -0.06) | 18.09 (14.16; 23.26) | 18.09 (14.16; 23.29) | 0.89 (0.53; 1.24) | 3.40 (1.50; 6.69) | 3.35 (1.48; 6.60) | -0.33 (-1.41; 0.75) | 0.00 (0.00; 0.00) | 0.00 (0.00; 0.00) | 14.20 (13.73; 14.66) | 0.00 (0.00; 0.00) | 0.00 (0.00; 0.00) | 16.38 (15.86; 16.90) | 0.00 (0.00; 0.00) | 0.00 (0.00; 0.00) | 14.13 (12.96; 15.31) |
| **Niue** | 8.79 (7.11; 10.60) | 8.71 (7.05; 10.50) | -0.31 (-0.59; -0.03) | 20.19 (14.62; 28.94) | 20.13 (14.58; 28.87) | 0.34 (-0.16; 0.85) | 3.36 (1.47; 6.82) | 3.34 (1.45; 6.77) | -0.30 (-1.42; 0.83) | 0.00 (0.00; 0.00) | 0.00 (0.00; 0.00) | -20.19 (-20.56; -19.83) | 0.00 (0.00; 0.00) | 0.00 (0.00; 0.00) | -20.49 (-21.06; -19.93) | 0.00 (0.00; 0.00) | 0.00 (0.00; 0.00) | -20.05 (-21.10; -19.01) |
| **Northern Mariana Islands** | 8.71 (7.10; 10.48) | 8.67 (7.07; 10.42) | -0.17 (-0.44; 0.11) | 20.18 (14.30; 28.71) | 20.12 (14.25; 28.68) | 0.30 (-0.21; 0.81) | 3.33 (1.44; 6.78) | 3.32 (1.43; 6.74) | -0.16 (-1.29; 0.97) | 0.00 (0.00; 0.00) | 0.00 (0.00; 0.00) | -17.52 (-17.85; -17.20) | 0.01 (0.01; 0.01) | 0.01 (0.00; 0.01) | -18.90 (-19.42; -18.38) | 0.00 (0.00; 0.00) | 0.00 (0.00; 0.00) | -17.51 (-18.56; -16.47) |
| **Palau** | 8.87 (7.32; 10.82) | 8.81 (7.27; 10.75) | -0.18 (-0.46; 0.10) | 19.83 (14.27; 27.58) | 19.76 (14.19; 27.52) | 0.25 (-0.22; 0.73) | 3.41 (1.47; 6.52) | 3.39 (1.46; 6.48) | -0.18 (-1.23; 0.87) | 0.00 (0.00; 0.00) | 0.00 (0.00; 0.00) | -20.25 (-20.58; -19.92) | 0.00 (0.00; 0.00) | 0.00 (0.00; 0.00) | -21.04 (-21.56; -20.51) | 0.00 (0.00; 0.00) | 0.00 (0.00; 0.00) | -20.21 (-21.23; -19.18) |
| **Papua New Guinea** | 8.92 (7.26; 10.67) | 8.74 (7.12; 10.48) | -0.50 (-0.77; -0.23) | 20.20 (14.61; 28.73) | 20.23 (14.65; 28.90) | 1.10 (0.60; 1.60) | 3.40 (1.47; 6.83) | 3.34 (1.44; 6.70) | -0.49 (-1.60; 0.63) | 1.24 (1.01; 1.47) | 2.11 (1.51; 2.79) | 65.18 (64.63; 65.74) | 2.72 (1.99; 3.80) | 4.86 (3.22; 7.00) | 72.77 (71.99; 73.54) | 0.00 (0.00; 0.00) | 0.00 (0.00; 0.00) | 65.07 (63.44; 66.70) |
| **Samoa** | 8.95 (7.32; 10.80) | 8.83 (7.21; 10.66) | -0.29 (-0.56; -0.01) | 20.08 (14.83; 29.62) | 20.04 (14.82; 29.50) | 0.66 (0.13; 1.18) | 3.42 (1.50; 6.58) | 3.37 (1.47; 6.49) | -0.28 (-1.33; 0.77) | 0.03 (0.02; 0.03) | 0.03 (0.02; 0.04) | 27.96 (27.52; 28.40) | 0.06 (0.04; 0.08) | 0.07 (0.05; 0.11) | 29.70 (29.09; 30.31) | 0.00 (0.00; 0.00) | 0.00 (0.00; 0.00) | 28.11 (26.81; 29.40) |
| **Solomon Islands** | 9.00 (7.38; 10.90) | 8.85 (7.25; 10.71) | -0.48 (-0.75; -0.20) | 20.08 (14.69; 29.21) | 19.97 (14.46; 29.02) | 0.29 (-0.23; 0.80) | 3.46 (1.46; 6.83) | 3.40 (1.43; 6.72) | -0.46 (-1.56; 0.63) | 0.08 (0.07; 0.10) | 0.09 (0.07; 0.12) | 8.94 (8.54; 9.34) | 0.18 (0.13; 0.26) | 0.21 (0.13; 0.31) | 12.55 (11.95; 13.15) | 0.00 (0.00; 0.00) | 0.00 (0.00; 0.00) | 9.02 (7.83; 10.20) |
| **Tokelau** | 8.40 (7.17; 9.74) | 8.38 (7.16; 9.72) | -0.42 (-0.63; -0.20) | 18.17 (14.18; 23.28) | 18.22 (14.17; 23.34) | 0.62 (0.26; 0.98) | 3.23 (1.44; 6.28) | 3.22 (1.44; 6.26) | -0.41 (-1.47; 0.64) | 0.00 (0.00; 0.00) | 0.00 (0.00; 0.00) | -30.68 (-30.98; -30.37) | 0.00 (0.00; 0.00) | 0.00 (0.00; 0.00) | -32.92 (-33.33; -32.51) | 0.00 (0.00; 0.00) | 0.00 (0.00; 0.00) | -30.56 (-31.51; -29.62) |
| **Tonga** | 8.90 (7.26; 10.77) | 8.77 (7.15; 10.62) | -0.34 (-0.62; -0.06) | 20.15 (14.58; 28.57) | 20.10 (14.47; 28.74) | 0.57 (0.07; 1.07) | 3.41 (1.44; 6.72) | 3.36 (1.42; 6.62) | -0.33 (-1.43; 0.76) | 0.01 (0.01; 0.01) | 0.01 (0.01; 0.02) | 9.59 (9.18; 10.00) | 0.03 (0.02; 0.04) | 0.03 (0.02; 0.05) | 10.98 (10.42; 11.55) | 0.00 (0.00; 0.00) | 0.00 (0.00; 0.00) | 9.66 (8.48; 10.85) |
| **Tuvalu** | 9.01 (7.42; 10.69) | 8.88 (7.31; 10.54) | -0.42 (-0.67; -0.16) | 20.02 (14.16; 29.29) | 19.96 (14.04; 29.00) | 0.49 (-0.04; 1.02) | 3.44 (1.51; 7.13) | 3.39 (1.49; 7.03) | -0.41 (-1.56; 0.75) | 0.00 (0.00; 0.00) | 0.00 (0.00; 0.00) | 7.82 (7.41; 8.23) | 0.00 (0.00; 0.00) | 0.00 (0.00; 0.00) | 9.95 (9.33; 10.57) | 0.00 (0.00; 0.00) | 0.00 (0.00; 0.00) | 7.79 (6.63; 8.96) |
| **Vanuatu** | 9.01 (7.27; 10.89) | 8.88 (7.17; 10.75) | -0.43 (-0.71; -0.14) | 20.00 (14.24; 29.26) | 20.00 (14.31; 28.97) | 0.78 (0.25; 1.31) | 3.44 (1.47; 6.76) | 3.39 (1.45; 6.67) | -0.42 (-1.50; 0.67) | 0.04 (0.03; 0.04) | 0.04 (0.03; 0.06) | 16.93 (16.54; 17.33) | 0.08 (0.06; 0.12) | 0.10 (0.07; 0.15) | 20.11 (19.50; 20.72) | 0.00 (0.00; 0.00) | 0.00 (0.00; 0.00) | 16.85 (15.66; 18.04) |
| **Southeast Asia** | 8.90 (7.38; 10.55) | 8.78 (7.30; 10.39) | -0.53 (-0.78; -0.28) | 20.37 (15.17; 29.25) | 20.48 (15.28; 29.39) | 1.25 (0.76; 1.75) | 3.41 (1.47; 6.76) | 3.36 (1.45; 6.67) | -0.52 (-1.62; 0.57) | 52.48 (43.54; 62.50) | 40.51 (31.88; 50.94) | -22.25 (-22.51; -22) | 122.52 (90.61; 178.33) | 97.51 (68.78; 142.01) | -20.07 (-20.54; -19.60) | 0.02 (0.01; 0.04) | 0.02 (0.01; 0.03) | -22.27 (-23.24; -21.30) |
| **Cambodia** | 8.88 (7.08; 10.68) | 8.75 (6.98; 10.52) | -0.55 (-0.83; -0.26) | 19.86 (14.63; 28.69) | 19.95 (14.64; 29.11) | 1.16 (0.65; 1.67) | 3.40 (1.42; 6.77) | 3.35 (1.40; 6.68) | -0.53 (-1.64; 0.58) | 1.57 (1.25; 1.89) | 1.28 (0.88; 1.72) | -17.85 (-18.24; -17.46) | 3.54 (2.60; 5.15) | 3.01 (1.95; 4.71) | -14.54 (-15.11; -13.98) | 0.00 (0.00; 0.00) | 0.00 (0.00; 0.00) | -17.83 (-18.84; -16.82) |
| **Indonesia** | 9.32 (7.66; 11.07) | 9.21 (7.57; 10.94) | -0.39 (-0.65; -0.13) | 21.01 (15.32; 30.55) | 20.91 (15.21; 30.59) | 0.24 (-0.28; 0.76) | 3.56 (1.53; 7.04) | 3.52 (1.51; 6.96) | -0.38 (-1.47; 0.71) | 21.32 (17.52; 25.45) | 16.02 (12.01; 20.97) | -24.45 (-24.74; -24.16) | 49.18 (35.47; 72.57) | 37.67 (25.68; 56.03) | -23.10 (-23.59; -22.61) | 0.01 (0.00; 0.02) | 0.01 (0.00; 0.01) | -24.50 (-25.49; -23.52) |
| **Lao People's Democratic Republic** | 8.88 (7.16; 10.67) | 8.73 (7.04; 10.51) | -0.57 (-0.85; -0.29) | 20.19 (14.48; 29.24) | 20.03 (14.40; 28.86) | 0.04 (-0.48; 0.55) | 3.39 (1.45; 6.95) | 3.34 (1.43; 6.84) | -0.56 (-1.70; 0.58) | 0.71 (0.57; 0.85) | 0.56 (0.40; 0.73) | -23 (-23.32; -22.68) | 1.60 (1.16; 2.32) | 1.32 (0.83; 1.98) | -19.26 (-19.76; -18.75) | 0.00 (0.00; 0.00) | 0.00 (0.00; 0.00) | -22.97 (-23.94; -22) |
| **Malaysia** | 8.37 (6.61; 10.08) | 8.29 (6.55; 9.98) | -0.39 (-0.68; -0.10) | 19.86 (14.38; 27.40) | 19.82 (14.37; 27.43) | 0.49 (0.03; 0.96) | 3.20 (1.35; 6.59) | 3.17 (1.33; 6.53) | -0.38 (-1.54; 0.77) | 2.17 (1.72; 2.62) | 1.71 (1.24; 2.30) | -20.49 (-20.84; -20.15) | 5.26 (3.77; 7.43) | 4.22 (2.75; 5.99) | -19.06 (-19.55; -18.58) | 0.00 (0.00; 0.00) | 0.00 (0.00; 0.00) | -20.48 (-21.50; -19.46) |
| **Maldives** | 8.53 (6.83; 10.35) | 8.45 (6.76; 10.26) | -0.50 (-0.79; -0.21) | 20.08 (14.50; 28.75) | 19.94 (14.33; 28.41) | -0.03 (-0.53; 0.47) | 3.28 (1.38; 6.27) | 3.24 (1.36; 6.21) | -0.49 (-1.55; 0.56) | 0.03 (0.02; 0.04) | 0.03 (0.02; 0.04) | -6.26 (-6.64; -5.88) | 0.07 (0.05; 0.10) | 0.07 (0.04; 0.10) | -4.81 (-5.38; -4.25) | 0.00 (0.00; 0.00) | 0.00 (0.00; 0.00) | -6.37 (-7.42; -5.32) |
| **Mauritius** | 7.27 (5.72; 8.81) | 7.19 (5.66; 8.71) | -0.34 (-0.64; -0.04) | 14.84 (10.04; 19.20) | 14.77 (10.00; 19.06) | 0.33 (-0.11; 0.76) | 2.79 (1.27; 5.99) | 2.75 (1.25; 5.92) | -0.34 (-1.53; 0.86) | 0.05 (0.04; 0.06) | 0.03 (0.02; 0.04) | -42.72 (-43.03; -42.41) | 0.10 (0.07; 0.13) | 0.06 (0.04; 0.09) | -41.59 (-42; -41.17) | 0.00 (0.00; 0.00) | 0.00 (0.00; 0.00) | -42.66 (-43.66; -41.66) |
| **Myanmar** | 7.49 (5.87; 9.53) | 7.38 (5.79; 9.40) | -0.52 (-0.87; -0.18) | 20.02 (14.34; 26.99) | 20.28 (14.54; 27.15) | 2.01 (1.56; 2.46) | 2.88 (1.23; 5.67) | 2.84 (1.21; 5.60) | -0.51 (-1.60; 0.58) | 4.00 (3.14; 5.11) | 3.06 (2.25; 4.11) | -23.35 (-23.72; -22.98) | 10.82 (7.72; 14.79) | 8.67 (5.72; 12.33) | -19.92 (-20.37; -19.46) | 0.00 (0.00; 0.00) | 0.00 (0.00; 0.00) | -23.35 (-24.34; -22.36) |
| **Philippines** | 8.77 (7.42; 10.26) | 8.66 (7.34; 10.14) | -0.35 (-0.58; -0.12) | 20.17 (15.33; 27.67) | 20.58 (15.64; 28.10) | 2.70 (2.27; 3.14) | 3.35 (1.46; 6.55) | 3.31 (1.44; 6.47) | -0.35 (-1.42; 0.73) | 10.16 (8.61; 11.91) | 9.20 (7.20; 11.43) | -9.02 (-9.30; -8.73) | 23.79 (17.96; 33.04) | 22.46 (15.78; 31.89) | -5.36 (-5.83; -4.89) | 0.00 (0.00; 0.01) | 0.00 (0.00; 0.01) | -8.97 (-10.03; -7.91) |
| **Seychelles** | 8.48 (6.79; 10.41) | 8.37 (6.71; 10.29) | -0.36 (-0.66; -0.06) | 20.01 (14.51; 28.71) | 20.01 (14.56; 28.80) | 0.76 (0.25; 1.26) | 3.24 (1.38; 6.55) | 3.20 (1.36; 6.47) | -0.36 (-1.48; 0.77) | 0.01 (0.01; 0.01) | 0.01 (0.01; 0.01) | 12.41 (11.97; 12.84) | 0.02 (0.01; 0.02) | 0.02 (0.01; 0.03) | 14.53 (13.93; 15.13) | 0.00 (0.00; 0.00) | 0.00 (0.00; 0.00) | 12.42 (11.20; 13.63) |
| **Sri Lanka** | 9.34 (7.54; 10.65) | 9.25 (7.48; 10.55) | -0.31 (-0.55; -0.08) | 19.58 (13.96; 29.86) | 19.20 (13.67; 29.32) | -1.35 (-1.92; -0.77) | 3.58 (1.52; 7.26) | 3.55 (1.50; 7.20) | -0.30 (-1.44; 0.83) | 1.61 (1.31; 1.84) | 0.98 (0.69; 1.30) | -38.27 (-38.55; -37.98) | 3.49 (2.44; 5.48) | 2.09 (1.24; 3.48) | -38.82 (-39.37; -38.27) | 0.00 (0.00; 0.00) | 0.00 (0.00; 0.00) | -38.20 (-39.11; -37.29) |
| **Thailand** | 8.97 (7.52; 10.38) | 8.89 (7.45; 10.30) | -0.33 (-0.56; -0.10) | 19.24 (13.77; 28.21) | 20.25 (14.52; 29.33) | 5.63 (5.09; 6.17) | 3.42 (1.44; 6.91) | 3.39 (1.43; 6.85) | -0.32 (-1.45; 0.80) | 2.90 (2.41; 3.38) | 1.59 (1.19; 2.03) | -43.60 (-43.86; -43.35) | 6.49 (4.53; 9.97) | 3.78 (2.43; 6.01) | -40.41 (-40.92; -39.90) | 0.00 (0.00; 0.00) | 0.00 (0.00; 0.00) | -43.60 (-44.51; -42.68) |
| **Timor-Leste** | 10.16 (7.96; 11.96) | 9.97 (7.82; 11.75) | -0.58 (-0.86; -0.30) | 19.18 (12.97; 29.99) | 19.19 (12.99; 29.82) | 0.87 (0.24; 1.50) | 3.88 (1.57; 8.09) | 3.81 (1.54; 7.94) | -0.56 (-1.75; 0.62) | 0.18 (0.14; 0.21) | 0.18 (0.12; 0.25) | 0.12 (-0.29; 0.54) | 0.34 (0.23; 0.54) | 0.36 (0.21; 0.60) | 3.92 (3.21; 4.63) | 0.00 (0.00; 0.00) | 0.00 (0.00; 0.00) | 0.28 (-0.91; 1.46) |
| **Viet Nam** | 8.85 (7.12; 10.66) | 8.76 (7.05; 10.56) | -0.39 (-0.67; -0.11) | 20.04 (14.37; 29.37) | 20.03 (14.37; 29.20) | 0.60 (0.07; 1.13) | 3.41 (1.49; 6.53) | 3.37 (1.48; 6.47) | -0.38 (-1.43; 0.66) | 7.69 (6.19; 9.30) | 5.81 (4.40; 7.53) | -23.75 (-24.05; -23.45) | 17.66 (12.59; 25.93) | 13.66 (9.03; 20.75) | -22.52 (-23.06; -21.99) | 0.00 (0.00; 0.01) | 0.00 (0.00; 0.00) | -23.77 (-24.77; -22.76) |
| **Sub-Saharan Africa** | 6.84 (5.61; 8.00) | 6.73 (5.53; 7.88) | -0.42 (-0.67; -0.18) | 16.55 (12.86; 20.75) | 16.52 (12.90; 20.76) | 0.85 (0.51; 1.19) | 2.61 (1.14; 5.08) | 2.57 (1.13; 5.00) | -0.43 (-1.49; 0.64) | 113.96 (93.73; 133.25) | 152.79 (117.44; 191.84) | 32.39 (32.02; 32.76) | 273.99 (213.73; 342.65) | 376.95 (277.48; 491.92) | 35.66 (35.21; 36.11) | 0.04 (0.02; 0.08) | 0.06 (0.03; 0.11) | 32.38 (31.12; 33.64) |
| **Central Sub-Saharan Africa** | 5.84 (4.36; 7.27) | 5.72 (4.26; 7.14) | -0.99 (-1.35; -0.64) | 13.58 (8.98; 18.15) | 13.43 (8.84; 18.10) | -0.13 (-0.61; 0.35) | 2.23 (0.98; 4.61) | 2.18 (0.96; 4.54) | -1.00 (-2.15; 0.15) | 12.10 (9.02; 15.07) | 14.93 (9.79; 20.35) | 22.43 (21.89; 22.96) | 28.09 (18.58; 37.44) | 35.25 (21.67; 53.40) | 24.21 (23.53; 24.88) | 0.00 (0.00; 0.01) | 0.01 (0.00; 0.01) | 22.39 (21.03; 23.76) |
| **Angola** | 5.63 (4.15; 7.21) | 5.53 (4.07; 7.06) | -0.79 (-1.17; -0.41) | 13.31 (8.79; 18.12) | 13.26 (8.83; 17.96) | 0.66 (0.17; 1.15) | 2.16 (0.92; 4.59) | 2.12 (0.91; 4.50) | -0.79 (-1.99; 0.41) | 3.08 (2.27; 3.94) | 4.34 (2.86; 5.96) | 38.46 (37.88; 39.03) | 7.25 (4.83; 9.87) | 10.43 (6.45; 15.20) | 41.12 (40.43; 41.81) | 0.00 (0.00; 0.00) | 0.00 (0.00; 0.00) | 38.54 (37.06; 40.03) |
| **Central African Republic** | 5.87 (4.36; 7.40) | 5.74 (4.28; 7.25) | -0.74 (-1.11; -0.38) | 13.55 (9.11; 18.46) | 13.37 (8.78; 18.06) | -0.09 (-0.58; 0.40) | 2.23 (1.00; 4.64) | 2.18 (0.98; 4.54) | -0.75 (-1.90; 0.40) | 0.47 (0.35; 0.59) | 0.42 (0.26; 0.61) | -11.47 (-11.95; -10.98) | 1.07 (0.73; 1.45) | 0.98 (0.57; 1.51) | -9.54 (-10.11; -8.97) | 0.00 (0.00; 0.00) | 0.00 (0.00; 0.00) | -11.22 (-12.38; -10.05) |
| **Congo** | 7.42 (5.82; 9.24) | 7.34 (5.75; 9.13) | -0.49 (-0.82; -0.17) | 19.23 (13.62; 25.57) | 19.17 (13.64; 25.53) | 0.36 (-0.08; 0.80) | 2.84 (1.21; 5.83) | 2.80 (1.20; 5.76) | -0.48 (-1.63; 0.66) | 0.49 (0.39; 0.62) | 0.46 (0.33; 0.66) | -5.27 (-5.71; -4.84) | 1.30 (0.92; 1.74) | 1.24 (0.80; 1.80) | -4.28 (-4.81; -3.75) | 0.00 (0.00; 0.00) | 0.00 (0.00; 0.00) | -5.43 (-6.62; -4.24) |
| **Democratic Republic of the Congo** | 5.85 (4.31; 7.41) | 5.75 (4.24; 7.29) | -0.59 (-0.96; -0.22) | 13.40 (8.70; 18.16) | 13.29 (8.60; 18.09) | 0.10 (-0.41; 0.60) | 2.23 (0.98; 4.64) | 2.20 (0.96; 4.56) | -0.59 (-1.74; 0.57) | 7.82 (5.76; 9.90) | 9.44 (5.47; 14.16) | 19.85 (19.17; 20.53) | 17.90 (11.58; 24.24) | 21.92 (12.17; 36.59) | 21.44 (20.61; 22.27) | 0.00 (0.00; 0.01) | 0.00 (0.00; 0.01) | 19.74 (18.36; 21.12) |
| **Equatorial Guinea** | 5.51 (3.95; 7.09) | 5.45 (3.91; 7.03) | -0.49 (-0.89; -0.09) | 13.35 (8.88; 18.07) | 13.41 (8.91; 18.18) | 1.09 (0.60; 1.58) | 2.11 (0.92; 4.58) | 2.08 (0.91; 4.54) | -0.50 (-1.73; 0.73) | 0.11 (0.08; 0.14) | 0.15 (0.10; 0.22) | 38.31 (37.69; 38.93) | 0.27 (0.18; 0.36) | 0.37 (0.22; 0.54) | 39 (38.29; 39.70) | 0.00 (0.00; 0.00) | 0.00 (0.00; 0.00) | 38.19 (36.73; 39.66) |
| **Gabon** | 5.52 (4.03; 7.06) | 5.46 (3.99; 6.98) | -0.52 (-0.90; -0.13) | 13.32 (8.91; 18.24) | 13.22 (8.86; 17.98) | -0.01 (-0.51; 0.48) | 2.11 (0.91; 4.41) | 2.08 (0.90; 4.35) | -0.51 (-1.68; 0.66) | 0.12 (0.09; 0.16) | 0.13 (0.08; 0.19) | 4.87 (4.35; 5.39) | 0.30 (0.20; 0.41) | 0.31 (0.18; 0.47) | 5.28 (4.67; 5.89) | 0.00 (0.00; 0.00) | 0.00 (0.00; 0.00) | 4.96 (3.70; 6.22) |
| **Eastern Sub-Saharan Africa** | 7.05 (5.80; 8.14) | 6.92 (5.73; 7.98) | -0.61 (-0.84; -0.38) | 16.82 (13.18; 21.31) | 17.09 (13.41; 21.57) | 2.61 (2.27; 2.95) | 2.69 (1.19; 5.24) | 2.64 (1.17; 5.13) | -0.62 (-1.68; 0.43) | 43.78 (36.05; 50.57) | 55.63 (42.54; 70.12) | 25.68 (25.33; 26.04) | 104.12 (81.67; 131.79) | 138.54 (99.90; 184.09) | 31.48 (31.01; 31.95) | 0.02 (0.01; 0.03) | 0.02 (0.01; 0.04) | 25.62 (24.39; 26.84) |
| **Burundi** | 6.05 (4.61; 7.58) | 5.94 (4.52; 7.43) | -0.64 (-0.99; -0.29) | 13.36 (8.96; 18.07) | 13.26 (8.91; 18.09) | 0.41 (-0.07; 0.90) | 2.31 (1.02; 5.07) | 2.27 (1.00; 4.96) | -0.64 (-1.88; 0.59) | 1.25 (0.95; 1.57) | 1.67 (1.12; 2.37) | 31.47 (30.90; 32.04) | 2.75 (1.85; 3.72) | 3.74 (2.23; 5.58) | 33.84 (33.12; 34.55) | 0.00 (0.00; 0.00) | 0.00 (0.00; 0.00) | 31.44 (29.98; 32.91) |
| **Comoros** | 7.73 (5.93; 9.69) | 7.61 (5.85; 9.56) | -0.55 (-0.89; -0.21) | 19.10 (13.57; 27.02) | 18.99 (13.62; 26.78) | 0.19 (-0.30; 0.69) | 2.95 (1.23; 5.90) | 2.91 (1.21; 5.81) | -0.53 (-1.65; 0.58) | 0.06 (0.05; 0.08) | 0.05 (0.03; 0.07) | -26 (-26.46; -25.54) | 0.16 (0.11; 0.23) | 0.12 (0.07; 0.20) | -23.98 (-24.55; -23.42) | 0.00 (0.00; 0.00) | 0.00 (0.00; 0.00) | -25.75 (-26.88; -24.63) |
| **Djibouti** | 5.90 (4.36; 7.43) | 5.80 (4.29; 7.30) | -0.68 (-1.05; -0.31) | 13.28 (8.95; 17.95) | 13.18 (8.77; 18.07) | 0.15 (-0.34; 0.64) | 2.26 (0.98; 4.72) | 2.22 (0.96; 4.63) | -0.68 (-1.85; 0.49) | 0.09 (0.06; 0.11) | 0.07 (0.05; 0.11) | -13.89 (-14.34; -13.44) | 0.19 (0.13; 0.26) | 0.17 (0.10; 0.26) | -12.16 (-12.70; -11.61) | 0.00 (0.00; 0.00) | 0.00 (0.00; 0.00) | -13.75 (-14.91; -12.59) |
| **Eritrea** | 5.98 (4.54; 7.55) | 5.87 (4.45; 7.42) | -0.68 (-1.04; -0.32) | 13.39 (9.07; 18.45) | 13.37 (9.04; 18.35) | 0.91 (0.41; 1.40) | 2.29 (1.00; 4.81) | 2.25 (0.98; 4.72) | -0.68 (-1.85; 0.49) | 0.53 (0.40; 0.67) | 0.50 (0.26; 0.84) | -6.32 (-7.00; -5.64) | 1.18 (0.80; 1.63) | 1.15 (0.58; 1.96) | -4.03 (-4.79; -3.28) | 0.00 (0.00; 0.00) | 0.00 (0.00; 0.00) | -6.38 (-7.68; -5.07) |
| **Ethiopia** | 8.21 (6.67; 9.87) | 8.04 (6.53; 9.68) | -0.81 (-1.08; -0.53) | 20.29 (14.82; 27.98) | 20.04 (14.70; 27.54) | -0.30 (-0.76; 0.16) | 3.13 (1.35; 6.11) | 3.06 (1.32; 5.99) | -0.79 (-1.86; 0.28) | 12.60 (10.22; 15.11) | 16.52 (11.68; 21.79) | 29.57 (29.10; 30.03) | 30.92 (22.60; 42.72) | 41.94 (27.24; 61.07) | 34.06 (33.41; 34.71) | 0.00 (0.00; 0.01) | 0.01 (0.00; 0.01) | 29.52 (28.15; 30.89) |
| **Kenya** | 7.42 (6.04; 8.88) | 7.34 (5.98; 8.78) | -0.47 (-0.74; -0.20) | 18.73 (14.33; 24.17) | 18.39 (14.05; 23.74) | -1.09 (-1.46; -0.72) | 2.84 (1.23; 5.45) | 2.81 (1.21; 5.39) | -0.46 (-1.51; 0.59) | 4.71 (3.83; 5.66) | 4.17 (2.89; 5.85) | -10.36 (-10.73; -9.98) | 12.14 (9.16; 15.95) | 10.70 (7.00; 15.31) | -10.94 (-11.40; -10.49) | 0.00 (0.00; 0.00) | 0.00 (0.00; 0.00) | -10.43 (-11.52; -9.34) |
| **Madagascar** | 6.42 (4.98; 7.97) | 6.33 (4.91; 7.87) | -0.50 (-0.83; -0.17) | 13.51 (9.27; 18.41) | 16.02 (10.64; 22.35) | 18.94 (18.39; 19.49) | 2.45 (1.08; 5.01) | 2.42 (1.07; 4.94) | -0.50 (-1.63; 0.63) | 2.64 (2.05; 3.28) | 3.06 (2.14; 4.12) | 15.61 (15.16; 16.06) | 5.56 (3.81; 7.58) | 7.79 (4.75; 11.49) | 38.73 (38.04; 39.42) | 0.00 (0.00; 0.00) | 0.00 (0.00; 0.00) | 15.68 (14.40; 16.95) |
| **Malawi** | 7.80 (6.10; 9.82) | 7.68 (6.01; 9.67) | -0.61 (-0.95; -0.28) | 19.13 (13.49; 26.96) | 19.05 (13.46; 26.58) | 0.32 (-0.17; 0.81) | 2.97 (1.25; 6.08) | 2.92 (1.23; 5.99) | -0.60 (-1.75; 0.55) | 2.16 (1.69; 2.73) | 2.26 (1.45; 3.24) | 5.09 (4.60; 5.57) | 5.37 (3.78; 7.74) | 5.72 (3.39; 8.74) | 6.92 (6.29; 7.54) | 0.00 (0.00; 0.00) | 0.00 (0.00; 0.00) | 5.08 (3.91; 6.26) |
| **Mozambique** | 6.00 (4.48; 7.62) | 5.89 (4.40; 7.48) | -0.81 (-1.18; -0.44) | 13.20 (8.99; 18.28) | 13.42 (9.18; 18.45) | 2.65 (2.15; 3.15) | 2.30 (1.01; 4.77) | 2.25 (0.99; 4.68) | -0.81 (-1.96; 0.34) | 3.05 (2.28; 3.88) | 3.68 (2.48; 5.27) | 19 (18.46; 19.53) | 6.70 (4.58; 9.27) | 8.40 (5.05; 12.27) | 23.71 (23.07; 24.35) | 0.00 (0.00; 0.00) | 0.00 (0.00; 0.00) | 19.12 (17.80; 20.44) |
| **Rwanda** | 5.96 (4.56; 7.53) | 5.86 (4.48; 7.40) | -0.64 (-0.99; -0.29) | 13.30 (8.95; 18.08) | 13.31 (9.00; 17.99) | 1.01 (0.52; 1.49) | 2.28 (1.01; 4.82) | 2.24 (0.99; 4.74) | -0.65 (-1.82; 0.53) | 1.03 (0.78; 1.30) | 1.27 (0.88; 1.71) | 21.81 (21.32; 22.30) | 2.29 (1.54; 3.11) | 2.90 (1.77; 4.22) | 24.94 (24.30; 25.59) | 0.00 (0.00; 0.00) | 0.00 (0.00; 0.00) | 21.89 (20.47; 23.31) |
| **Somalia** | 6.26 (4.86; 7.85) | 6.11 (4.75; 7.66) | -0.58 (-0.92; -0.25) | 13.49 (9.22; 18.31) | 13.37 (9.18; 18.17) | 0.59 (0.12; 1.07) | 2.39 (1.05; 4.99) | 2.33 (1.02; 4.88) | -0.58 (-1.75; 0.59) | 2.31 (1.80; 2.90) | 4.10 (2.58; 6.02) | 71.33 (70.53; 72.14) | 4.92 (3.40; 6.63) | 8.93 (5.15; 13.50) | 75.22 (74.30; 76.14) | 0.00 (0.00; 0.00) | 0.00 (0.00; 0.00) | 71.35 (69.59; 73.10) |
| **South Sudan** | 7.76 (6.00; 9.51) | 7.55 (5.84; 9.25) | -0.77 (-1.08; -0.45) | 19.11 (13.67; 27.07) | 19.11 (13.51; 27.10) | 1.15 (0.64; 1.65) | 2.95 (1.24; 5.87) | 2.87 (1.21; 5.71) | -0.74 (-1.85; 0.36) | 1.12 (0.87; 1.38) | 2.32 (1.64; 3.17) | 102.86 (102.14; 103.59) | 2.77 (1.97; 3.94) | 5.82 (3.81; 8.22) | 107.82 (106.94; 108.70) | 0.00 (0.00; 0.00) | 0.00 (0.00; 0.00) | 102.68 (100.89; 104.46) |
| **Uganda** | 7.53 (5.76; 9.44) | 7.38 (5.65; 9.26) | -0.74 (-1.09; -0.40) | 18.67 (13.42; 26.83) | 19.02 (13.73; 27.22) | 2.68 (2.16; 3.19) | 2.87 (1.21; 5.88) | 2.82 (1.19; 5.76) | -0.73 (-1.87; 0.42) | 5.24 (4.01; 6.55) | 7.41 (5.22; 10.09) | 38.73 (38.19; 39.26) | 12.83 (9.28; 18.37) | 19.14 (12.57; 28.21) | 46.09 (45.38; 46.80) | 0.00 (0.00; 0.00) | 0.00 (0.00; 0.01) | 38.66 (37.22; 40.10) |
| **United Republic of Tanzania** | 5.51 (4.04; 6.97) | 5.39 (3.96; 6.83) | -0.85 (-1.22; -0.47) | 12.60 (8.36; 17.35) | 14.02 (9.18; 19.61) | 12.05 (11.50; 12.60) | 2.10 (0.92; 4.42) | 2.06 (0.90; 4.34) | -0.83 (-2.01; 0.34) | 4.71 (3.45; 5.97) | 5.68 (3.76; 7.97) | 18.11 (17.57; 18.65) | 10.75 (7.20; 14.81) | 14.79 (8.95; 21.97) | 34.41 (33.71; 35.11) | 0.00 (0.00; 0.00) | 0.00 (0.00; 0.00) | 18.05 (16.74; 19.37) |
| **Zambia** | 7.72 (5.94; 9.74) | 7.61 (5.85; 9.61) | -0.55 (-0.90; -0.20) | 19.02 (13.39; 26.36) | 18.97 (13.38; 26.24) | 0.51 (0.03; 0.99) | 2.94 (1.23; 5.90) | 2.90 (1.21; 5.82) | -0.53 (-1.65; 0.59) | 2.24 (1.73; 2.83) | 2.84 (1.98; 4.04) | 25.51 (24.97; 26.05) | 5.51 (3.89; 7.60) | 7.13 (4.65; 10.51) | 28 (27.36; 28.64) | 0.00 (0.00; 0.00) | 0.00 (0.00; 0.00) | 25.27 (23.96; 26.58) |
| **Southern Sub-Saharan Africa** | 6.96 (5.48; 8.69) | 6.81 (5.35; 8.55) | -1.34 (-1.67; -1.01) | 18.24 (13.48; 23.38) | 18.05 (13.40; 23.23) | -0.29 (-0.67; 0.10) | 2.66 (1.12; 5.30) | 2.61 (1.09; 5.17) | -1.33 (-2.43; -0.23) | 5.78 (4.54; 7.23) | 5.04 (3.76; 6.52) | -12.15 (-12.51; -11.80) | 15.32 (11.31; 19.69) | 13.59 (9.45; 18.40) | -10.81 (-11.21; -10.40) | 0.00 (0.00; 0.00) | 0.00 (0.00; 0.00) | -12.13 (-13.18; -11.09) |
| **Botswana** | 6.71 (5.13; 8.43) | 6.62 (5.06; 8.32) | -0.50 (-0.85; -0.15) | 18.09 (12.63; 25.46) | 18.03 (12.63; 25.43) | 0.38 (-0.13; 0.88) | 2.56 (1.07; 4.94) | 2.53 (1.06; 4.87) | -0.49 (-1.55; 0.58) | 0.16 (0.12; 0.20) | 0.13 (0.09; 0.19) | -16.63 (-17.04; -16.21) | 0.44 (0.31; 0.62) | 0.37 (0.23; 0.57) | -14.95 (-15.48; -14.42) | 0.00 (0.00; 0.00) | 0.00 (0.00; 0.00) | -16.71 (-17.72; -15.69) |
| **Eswatini** | 7.64 (5.87; 9.30) | 7.53 (5.79; 9.18) | -0.48 (-0.80; -0.16) | 18.47 (12.86; 27.12) | 18.35 (12.78; 26.86) | 0.11 (-0.43; 0.66) | 2.92 (1.21; 5.80) | 2.88 (1.19; 5.71) | -0.47 (-1.57; 0.64) | 0.11 (0.08; 0.13) | 0.08 (0.06; 0.11) | -23.46 (-23.82; -23.09) | 0.27 (0.18; 0.39) | 0.21 (0.13; 0.31) | -22.01 (-22.54; -21.48) | 0.00 (0.00; 0.00) | 0.00 (0.00; 0.00) | -23.53 (-24.54; -22.52) |
| **Lesotho** | 5.63 (4.08; 7.30) | 5.56 (4.03; 7.21) | -0.43 (-0.83; -0.02) | 13.34 (8.84; 18.54) | 13.29 (8.82; 18.46) | 0.42 (-0.09; 0.93) | 2.16 (0.94; 4.62) | 2.13 (0.93; 4.57) | -0.43 (-1.63; 0.78) | 0.12 (0.09; 0.16) | 0.10 (0.06; 0.15) | -15.63 (-16.09; -15.18) | 0.29 (0.19; 0.40) | 0.24 (0.14; 0.36) | -15.02 (-15.56; -14.49) | 0.00 (0.00; 0.00) | 0.00 (0.00; 0.00) | -15.64 (-16.78; -14.49) |
| **Namibia** | 5.40 (3.88; 7.05) | 5.33 (3.83; 6.96) | -0.43 (-0.84; -0.02) | 13.22 (8.80; 18.32) | 13.20 (8.77; 18.34) | 0.68 (0.17; 1.19) | 2.08 (0.89; 4.55) | 2.05 (0.88; 4.49) | -0.43 (-1.67; 0.82) | 0.15 (0.11; 0.20) | 0.15 (0.10; 0.22) | -0.49 (-1.01; 0.02) | 0.38 (0.25; 0.52) | 0.38 (0.24; 0.56) | 0.86 (0.29; 1.42) | 0.00 (0.00; 0.00) | 0.00 (0.00; 0.00) | -0.50 (-1.79; 0.78) |
| **South Africa** | 7.40 (5.82; 9.30) | 7.33 (5.76; 9.21) | -0.30 (-0.64; 0.03) | 18.91 (14.06; 24.12) | 18.89 (14.07; 24.24) | 0.54 (0.16; 0.92) | 2.83 (1.20; 5.66) | 2.80 (1.19; 5.61) | -0.30 (-1.41; 0.81) | 3.87 (3.03; 4.87) | 3.10 (2.25; 4.06) | -18.89 (-19.26; -18.52) | 10.04 (7.43; 12.91) | 8.18 (5.61; 11.00) | -17.86 (-18.26; -17.46) | 0.00 (0.00; 0.00) | 0.00 (0.00; 0.00) | -18.86 (-19.88; -17.85) |
| **Zimbabwe** | 6.24 (4.68; 8.11) | 6.15 (4.61; 8.00) | -0.45 (-0.84; -0.07) | 17.79 (12.36; 23.35) | 17.50 (12.09; 23.06) | -0.79 (-1.23; -0.35) | 2.39 (0.96; 4.85) | 2.35 (0.95; 4.79) | -0.44 (-1.59; 0.71) | 1.37 (1.03; 1.78) | 1.47 (1.00; 2.06) | 7.03 (6.55; 7.51) | 3.91 (2.71; 5.12) | 4.21 (2.59; 5.97) | 7.52 (6.97; 8.07) | 0.00 (0.00; 0.00) | 0.00 (0.00; 0.00) | 7.07 (5.87; 8.27) |
| **Western Sub-Saharan Africa** | 6.92 (5.54; 8.34) | 6.82 (5.44; 8.25) | -0.26 (-0.55; 0.03) | 16.95 (12.93; 21.59) | 16.74 (12.78; 21.32) | -0.22 (-0.58; 0.14) | 2.64 (1.12; 5.13) | 2.60 (1.11; 5.07) | -0.24 (-1.31; 0.84) | 52.30 (41.82; 63.00) | 77.18 (59.08; 96.93) | 45.07 (44.65; 45.48) | 126.47 (96.99; 160.15) | 189.56 (135.63; 246.70) | 47.10 (46.60; 47.59) | 0.02 (0.01; 0.04) | 0.03 (0.01; 0.06) | 45.14 (43.81; 46.47) |
| **Benin** | 7.08 (5.38; 8.90) | 6.93 (5.26; 8.71) | -0.88 (-1.23; -0.53) | 18.24 (12.76; 24.93) | 18.34 (12.76; 25.27) | 1.46 (0.98; 1.95) | 2.70 (1.10; 5.43) | 2.64 (1.08; 5.32) | -0.84 (-1.97; 0.29) | 1.53 (1.17; 1.92) | 2.42 (1.67; 3.26) | 54.89 (54.32; 55.47) | 3.85 (2.71; 5.24) | 6.42 (4.11; 9.01) | 62.45 (61.74; 63.15) | 0.00 (0.00; 0.00) | 0.00 (0.00; 0.00) | 55.05 (53.46; 56.64) |
| **Burkina Faso** | 5.77 (4.39; 7.31) | 5.62 (4.28; 7.12) | -0.79 (-1.14; -0.43) | 12.76 (8.51; 17.85) | 11.10 (7.58; 15.22) | -11.32 (-11.79; -10.84) | 2.20 (0.99; 4.60) | 2.14 (0.96; 4.48) | -0.80 (-1.95; 0.36) | 2.13 (1.62; 2.69) | 3.94 (2.78; 5.22) | 78.90 (78.29; 79.51) | 4.66 (3.14; 6.44) | 7.77 (5.08; 11.06) | 61.90 (61.18; 62.61) | 0.00 (0.00; 0.00) | 0.00 (0.00; 0.00) | 79.04 (77.36; 80.72) |
| **Cabo Verde** | 7.01 (5.16; 9.00) | 6.93 (5.10; 8.89) | -0.66 (-1.05; -0.28) | 18.21 (12.68; 24.87) | 18.20 (12.65; 24.89) | 0.54 (0.07; 1.02) | 2.69 (1.14; 5.26) | 2.65 (1.12; 5.20) | -0.65 (-1.73; 0.43) | 0.03 (0.02; 0.04) | 0.02 (0.01; 0.03) | -45.29 (-45.71; -44.88) | 0.09 (0.06; 0.13) | 0.05 (0.03; 0.09) | -44.09 (-44.62; -43.56) | 0.00 (0.00; 0.00) | 0.00 (0.00; 0.00) | -45.18 (-46.16; -44.20) |
| **Cameroon** | 7.04 (5.41; 8.97) | 6.92 (5.32; 8.82) | -0.63 (-0.99; -0.27) | 18.16 (12.69; 25.04) | 18.19 (12.77; 24.96) | 0.97 (0.49; 1.45) | 2.69 (1.12; 5.59) | 2.65 (1.10; 5.49) | -0.61 (-1.78; 0.56) | 3.34 (2.57; 4.24) | 3.95 (2.59; 5.78) | 17.17 (16.61; 17.74) | 8.55 (5.99; 11.75) | 10.46 (6.56; 15.45) | 20.62 (19.99; 21.25) | 0.00 (0.00; 0.00) | 0.00 (0.00; 0.00) | 17.06 (15.74; 18.38) |
| **Chad** | 7.16 (5.40; 9.11) | 6.98 (5.27; 8.90) | -0.91 (-1.28; -0.55) | 18.32 (12.79; 24.93) | 18.35 (12.63; 25.52) | 1.23 (0.75; 1.72) | 2.73 (1.14; 5.52) | 2.66 (1.12; 5.38) | -0.89 (-2.02; 0.24) | 2.30 (1.74; 2.92) | 5.69 (4.04; 7.44) | 139.43 (138.66; 140.20) | 5.70 (4.04; 7.61) | 14.73 (9.69; 20.63) | 149.30 (148.30; 150.29) | 0.00 (0.00; 0.00) | 0.00 (0.00; 0.00) | 139.37 (137.36; 141.38) |
| **Côte d'Ivoire** | 7.00 (5.25; 8.81) | 6.86 (5.14; 8.63) | -0.81 (-1.17; -0.45) | 18.13 (12.51; 24.85) | 18.16 (12.66; 24.51) | 1.06 (0.59; 1.54) | 2.67 (1.09; 5.16) | 2.62 (1.07; 5.05) | -0.79 (-1.87; 0.28) | 2.87 (2.15; 3.61) | 3.45 (2.39; 4.68) | 17.59 (17.12; 18.06) | 7.31 (5.06; 9.92) | 9.22 (5.79; 13.14) | 22.89 (22.28; 23.49) | 0.00 (0.00; 0.00) | 0.00 (0.00; 0.00) | 17.61 (16.40; 18.83) |
| **Gambia** | 7.19 (5.50; 9.09) | 7.05 (5.40; 8.93) | -0.75 (-1.10; -0.40) | 15.31 (11.95; 19.06) | 15.33 (12.03; 19.18) | 1.15 (0.81; 1.48) | 2.75 (1.17; 5.50) | 2.70 (1.15; 5.39) | -0.73 (-1.84; 0.37) | 0.25 (0.19; 0.31) | 0.25 (0.17; 0.34) | -1.63 (-2.05; -1.21) | 0.53 (0.41; 0.65) | 0.54 (0.37; 0.73) | 1.74 (1.32; 2.17) | 0.00 (0.00; 0.00) | 0.00 (0.00; 0.00) | -1.55 (-2.68; -0.41) |
| **Ghana** | 7.03 (5.41; 8.88) | 6.93 (5.33; 8.75) | -0.54 (-0.89; -0.20) | 18.05 (12.83; 24.63) | 17.99 (12.84; 24.66) | 0.44 (-0.02; 0.91) | 2.68 (1.10; 5.43) | 2.64 (1.08; 5.34) | -0.53 (-1.67; 0.61) | 3.20 (2.46; 4.03) | 3.28 (2.09; 4.78) | 1.50 (0.99; 2.02) | 8.14 (5.81; 11.07) | 8.62 (5.29; 12.94) | 4.29 (3.71; 4.87) | 0.00 (0.00; 0.00) | 0.00 (0.00; 0.00) | 1.68 (0.52; 2.85) |
| **Guinea** | 7.13 (5.37; 8.97) | 6.98 (5.26; 8.79) | -0.88 (-1.23; -0.52) | 18.17 (12.78; 24.19) | 18.13 (12.76; 24.13) | 0.69 (0.24; 1.13) | 2.73 (1.17; 5.63) | 2.67 (1.14; 5.53) | -0.86 (-2.01; 0.30) | 1.53 (1.15; 1.91) | 2.17 (1.52; 2.81) | 39.64 (39.14; 40.14) | 3.84 (2.73; 5.09) | 5.66 (3.71; 7.96) | 44.59 (43.95; 45.22) | 0.00 (0.00; 0.00) | 0.00 (0.00; 0.00) | 39.57 (38.18; 40.96) |
| **Guinea-Bissau** | 7.18 (5.43; 9.04) | 7.04 (5.33; 8.87) | -0.78 (-1.13; -0.42) | 18.29 (12.87; 24.97) | 18.33 (12.70; 25.29) | 1.12 (0.64; 1.60) | 2.74 (1.16; 5.42) | 2.68 (1.13; 5.32) | -0.76 (-1.86; 0.34) | 0.23 (0.17; 0.29) | 0.28 (0.18; 0.39) | 19.11 (18.58; 19.64) | 0.57 (0.41; 0.78) | 0.73 (0.45; 1.11) | 24.93 (24.25; 25.61) | 0.00 (0.00; 0.00) | 0.00 (0.00; 0.00) | 19.31 (18.03; 20.60) |
| **Liberia** | 7.21 (5.46; 9.16) | 7.10 (5.37; 9.02) | -0.56 (-0.93; -0.20) | 18.25 (12.65; 25.05) | 18.24 (12.64; 25.32) | 0.74 (0.25; 1.23) | 2.75 (1.15; 5.67) | 2.71 (1.13; 5.58) | -0.55 (-1.71; 0.61) | 0.55 (0.41; 0.69) | 0.57 (0.37; 0.87) | 4.69 (4.14; 5.25) | 1.38 (0.96; 1.90) | 1.50 (0.88; 2.28) | 7.71 (7.09; 8.34) | 0.00 (0.00; 0.00) | 0.00 (0.00; 0.00) | 4.67 (3.46; 5.89) |
| **Mali** | 7.11 (5.37; 9.00) | 6.94 (5.24; 8.79) | -0.87 (-1.23; -0.51) | 18.10 (12.87; 24.54) | 18.22 (13.00; 24.75) | 1.71 (1.25; 2.17) | 2.71 (1.13; 5.51) | 2.65 (1.10; 5.39) | -0.85 (-1.99; 0.29) | 2.92 (2.22; 3.68) | 5.92 (4.15; 7.97) | 96.84 (96.15; 97.54) | 7.23 (5.18; 9.74) | 15.38 (10.13; 21.54) | 106.07 (105.22; 106.91) | 0.00 (0.00; 0.00) | 0.00 (0.00; 0.00) | 96.86 (95.07; 98.64) |
| **Mauritania** | 7.06 (5.28; 8.86) | 6.95 (5.20; 8.73) | -0.67 (-1.02; -0.31) | 18.11 (12.78; 24.94) | 18.17 (12.79; 24.76) | 1.10 (0.62; 1.57) | 2.70 (1.11; 5.37) | 2.66 (1.09; 5.28) | -0.65 (-1.76; 0.46) | 0.46 (0.34; 0.58) | 0.54 (0.37; 0.78) | 17.29 (16.77; 17.81) | 1.18 (0.83; 1.62) | 1.43 (0.93; 2.18) | 20.64 (20.01; 21.27) | 0.00 (0.00; 0.00) | 0.00 (0.00; 0.00) | 17.15 (15.88; 18.41) |
| **Niger** | 7.77 (5.94; 9.76) | 7.60 (5.81; 9.57) | -0.60 (-0.95; -0.25) | 15.71 (12.24; 20.30) | 15.74 (12.17; 20.19) | 1.37 (1.00; 1.73) | 2.97 (1.23; 6.04) | 2.90 (1.21; 5.92) | -0.58 (-1.72; 0.56) | 3.53 (2.70; 4.41) | 9.07 (6.59; 12.06) | 147.54 (146.74; 148.34) | 7.01 (5.49; 8.88) | 18.48 (13.27; 24.44) | 154.07 (153.27; 154.88) | 0.00 (0.00; 0.00) | 0.00 (0.00; 0.01) | 147.70 (145.54; 149.86) |
| **Nigeria** | 6.87 (5.67; 8.12) | 6.73 (5.56; 7.95) | -0.70 (-0.95; -0.45) | 16.83 (12.94; 20.90) | 16.74 (12.90; 20.76) | 0.40 (0.07; 0.73) | 2.61 (1.13; 5.05) | 2.56 (1.10; 4.95) | -0.69 (-1.75; 0.37) | 24.37 (20.10; 28.80) | 32.19 (23.13; 42.35) | 30.52 (30.08; 30.96) | 59.28 (45.58; 73.38) | 80.50 (54.12; 108.72) | 33.94 (33.42; 34.46) | 0.01 (0.00; 0.02) | 0.01 (0.01; 0.02) | 30.59 (29.37; 31.81) |
| **Sao Tome and Principe** | 6.27 (5.09; 7.42) | 6.21 (5.05; 7.35) | -0.35 (-0.61; -0.08) | 12.71 (8.47; 17.54) | 12.69 (8.48; 17.51) | 0.43 (-0.07; 0.94) | 2.40 (1.06; 4.77) | 2.38 (1.05; 4.72) | -0.34 (-1.43; 0.75) | 0.02 (0.01; 0.02) | 0.01 (0.01; 0.02) | -33.88 (-34.24; -33.52) | 0.03 (0.02; 0.05) | 0.02 (0.01; 0.03) | -33.41 (-33.91; -32.92) | 0.00 (0.00; 0.00) | 0.00 (0.00; 0.00) | -33.74 (-34.71; -32.77) |
| **Senegal** | 7.13 (5.43; 9.05) | 7.00 (5.33; 8.89) | -0.79 (-1.15; -0.43) | 18.24 (12.73; 25.19) | 16.87 (11.53; 23.86) | -6.51 (-7.00; -6.03) | 2.72 (1.13; 5.61) | 2.67 (1.11; 5.50) | -0.78 (-1.94; 0.38) | 1.58 (1.20; 2.00) | 1.81 (1.24; 2.43) | 13.68 (13.21; 14.15) | 4.01 (2.80; 5.54) | 4.44 (2.75; 6.69) | 9.58 (8.97; 10.18) | 0.00 (0.00; 0.00) | 0.00 (0.00; 0.00) | 13.66 (12.42; 14.89) |
| **Sierra Leone** | 6.55 (5.38; 7.75) | 6.43 (5.28; 7.61) | -0.56 (-0.82; -0.31) | 12.92 (8.36; 17.47) | 13.93 (8.85; 19.05) | 8.94 (8.41; 9.48) | 2.51 (1.11; 4.79) | 2.46 (1.09; 4.71) | -0.59 (-1.63; 0.45) | 0.82 (0.68; 0.97) | 0.98 (0.72; 1.23) | 17.19 (16.82; 17.55) | 1.62 (1.06; 2.18) | 2.14 (1.29; 3.15) | 29.82 (29.15; 30.48) | 0.00 (0.00; 0.00) | 0.00 (0.00; 0.00) | 17.20 (16; 18.41) |
| **Togo** | 5.58 (4.13; 7.00) | 5.49 (4.06; 6.88) | -0.68 (-1.04; -0.31) | 12.71 (8.27; 17.50) | 12.72 (8.30; 17.56) | 0.94 (0.42; 1.45) | 2.12 (0.93; 4.53) | 2.09 (0.92; 4.46) | -0.68 (-1.88; 0.51) | 0.65 (0.48; 0.82) | 0.63 (0.41; 0.87) | -4.26 (-4.73; -3.80) | 1.48 (0.97; 2.04) | 1.46 (0.90; 2.14) | -1.95 (-2.51; -1.38) | 0.00 (0.00; 0.00) | 0.00 (0.00; 0.00) | -4.21 (-5.41; -3.01) |

*Footnote*

Different colors reflect different geographic levels. Red: Global; Blue: Super-Regions; Green: Regions; Yellow: Country.

TPC, total percentage change.

**Appendix Table 3.** Prevalence, incidence and burden of untreated caries of permanent teeth in 2021, with projections to 2050, in 21 GBD regions and 204 countries.

| **CARIES OF PERMANENT TEETH** | | | | | | | | | | | | | | | | | | |
| --- | --- | --- | --- | --- | --- | --- | --- | --- | --- | --- | --- | --- | --- | --- | --- | --- | --- | --- |
| **Location** | **Age-standardized** | | | | | | | | | **All Ages (nº of cases)** | | | | | | | | |
|  | **Prevalence (%)** | | | **Incidence (%)** | | | **YLDs (per 100,000)** | | | **Prevalence (Millions)** | | | **Incidence (Millions)** | | | **YLDs (Millions)** | | |
|  | **2021** | **2050** | **2021-2050 TPC (%)** | **2021** | **2050** | **2021-2050 TPC (%)** | **2021** | **2050** | **2021-2050 TPC (%)** | **2021** | **2050** | **2021-2050 TPC (%)** | **2021** | **2050** | **2021-2050 TPC (%)** | **2021** | **2050** | **2021-2050 TPC (%)** |
| **Global** | 27.54 (23.98; 32.02) | 27.76 (24.25; 32.10) | 0.61 (0.41; 0.82) | 29.78 (26.31; 33.49) | 30.26 (26.71; 34.05) | 1.40 (1.23; 1.58) | 27.02 (12.10; 51.36) | 27.20 (12.20; 51.43) | 0.51 (-0.52; 1.53) | 2242.87 (1957.58; 2599.62) | 2804.53 (2445.18; 3176.28) | 23.77 (23.56; 23.99) | 2370.41 (2099.10; 2661.35) | 2746.92 (2404.40; 3110.24) | 14.91 (14.72; 15.10) | 2.20 (0.99; 4.17) | 2.73 (1.24; 5.10) | 22.80 (21.66; 23.94) |
| **Central Europe, Eastern Europe, and Central Asia** | 33.13 (28.65; 38.23) | 33.06 (28.57; 38.13) | -0.30 (-0.50; -0.09) | 32.89 (28.67; 37.00) | 32.98 (28.75; 37.20) | 0.08 (-0.10; 0.26) | 32.55 (14.39; 61.95) | 32.50 (14.30; 61.90) | -0.27 (-1.31; 0.76) | 148.68 (128.23; 169.29) | 139.96 (119.90; 160.52) | -5.17 (-5.38; -4.97) | 134.85 (118.79; 151.89) | 124.23 (108.22; 141.20) | -7.21 (-7.39; -7.03) | 0.15 (0.07; 0.28) | 0.14 (0.06; 0.26) | -5.69 (-6.71; -4.67) |
| **Central Asia** | 36.16 (30.99; 42.09) | 35.62 (30.56; 41.37) | -1.58 (-1.80; -1.37) | 31.94 (27.49; 36.71) | 31.76 (27.37; 36.61) | -0.69 (-0.89; -0.48) | 35.58 (15.67; 67.47) | 35.06 (15.39; 66.45) | -1.55 (-2.57; -0.53) | 34.92 (29.81; 40.87) | 44.64 (36.81; 52.90) | 26.93 (26.64; 27.22) | 30.60 (26.28; 35.36) | 37.98 (31.22; 45.33) | 23.34 (23.05; 23.63) | 0.03 (0.02; 0.07) | 0.04 (0.02; 0.08) | 26.23 (25.04; 27.42) |
| **Armenia** | 36.16 (30.85; 42.06) | 35.65 (30.41; 41.51) | -1.47 (-1.68; -1.25) | 31.74 (27.27; 36.95) | 31.91 (27.38; 37.20) | 0.34 (0.12; 0.56) | 35.59 (15.53; 67.50) | 35.10 (15.32; 66.59) | -1.44 (-2.46; -0.41) | 1.17 (0.99; 1.35) | 0.93 (0.70; 1.17) | -20.23 (-20.51; -19.94) | 0.94 (0.82; 1.11) | 0.71 (0.54; 0.90) | -24.42 (-24.70; -24.15) | 0.00 (0.00; 0.00) | 0.00 (0.00; 0.00) | -20.98 (-21.93; -20.04) |
| **Azerbaijan** | 35.52 (30.04; 41.35) | 35.05 (29.65; 40.78) | -1.45 (-1.67; -1.22) | 32.14 (27.26; 37.21) | 32.63 (27.66; 37.78) | 1.31 (1.09; 1.53) | 35.00 (15.59; 67.74) | 34.55 (15.38; 66.88) | -1.42 (-2.46; -0.37) | 4.02 (3.40; 4.68) | 4.37 (3.45; 5.33) | 7.92 (7.61; 8.24) | 3.48 (2.96; 4.07) | 3.58 (2.87; 4.34) | 2.49 (2.19; 2.79) | 0.00 (0.00; 0.01) | 0.00 (0.00; 0.01) | 6.99 (5.87; 8.11) |
| **Georgia** | 36.16 (30.40; 42.14) | 35.64 (29.98; 41.55) | -1.48 (-1.71; -1.25) | 32.03 (27.27; 37.29) | 31.34 (26.67; 36.55) | -2.13 (-2.35; -1.91) | 35.57 (15.63; 67.58) | 35.07 (15.42; 66.69) | -1.46 (-2.49; -0.44) | 1.38 (1.17; 1.59) | 1.26 (1.01; 1.53) | -8.43 (-8.69; -8.17) | 1.12 (0.96; 1.30) | 1.01 (0.80; 1.24) | -8.89 (-9.17; -8.61) | 0.00 (0.00; 0.00) | 0.00 (0.00; 0.00) | -8.79 (-9.78; -7.79) |
| **Kazakhstan** | 34.97 (29.73; 40.88) | 34.39 (29.21; 40.23) | -1.76 (-1.98; -1.54) | 32.38 (27.54; 37.45) | 31.57 (26.83; 36.45) | -2.59 (-2.80; -2.38) | 34.36 (15.03; 66.87) | 33.80 (14.79; 65.84) | -1.74 (-2.80; -0.68) | 6.70 (5.66; 7.80) | 8.21 (6.70; 9.92) | 21.92 (21.61; 22.23) | 6.06 (5.13; 6.99) | 7.29 (5.80; 8.71) | 19.53 (19.23; 19.84) | 0.01 (0.00; 0.01) | 0.01 (0.00; 0.01) | 21.30 (20.18; 22.41) |
| **Kyrgyzstan** | 37.62 (32.10; 43.65) | 37.06 (31.63; 42.97) | -1.58 (-1.80; -1.37) | 31.71 (27.31; 36.71) | 31.41 (27.06; 36.33) | -1.05 (-1.26; -0.84) | 37.09 (16.13; 69.44) | 36.54 (15.89; 68.48) | -1.56 (-2.57; -0.56) | 2.51 (2.13; 2.93) | 3.21 (2.48; 3.98) | 26.50 (26.14; 26.87) | 2.18 (1.87; 2.54) | 2.63 (2.06; 3.31) | 19.13 (18.78; 19.49) | 0.00 (0.00; 0.00) | 0.00 (0.00; 0.01) | 25.85 (24.64; 27.06) |
| **Mongolia** | 35.99 (30.27; 41.99) | 35.23 (29.63; 41.15) | -2.22 (-2.45; -1.99) | 32.10 (27.67; 37.06) | 32.36 (27.84; 37.49) | 0.61 (0.40; 0.82) | 35.38 (15.63; 69.20) | 34.64 (15.31; 67.79) | -2.20 (-3.26; -1.14) | 1.18 (0.98; 1.38) | 1.95 (1.61; 2.31) | 62.41 (62.07; 62.76) | 1.06 (0.90; 1.23) | 1.77 (1.47; 2.10) | 63.59 (63.25; 63.92) | 0.00 (0.00; 0.00) | 0.00 (0.00; 0.00) | 61.87 (60.47; 63.26) |
| **Tajikistan** | 37.96 (33.08; 43.70) | 37.20 (32.42; 42.78) | -2.05 (-2.25; -1.86) | 31.73 (27.17; 37.08) | 31.54 (27.05; 36.79) | -0.71 (-0.92; -0.49) | 37.41 (16.32; 70.36) | 36.67 (15.98; 68.95) | -2.03 (-3.04; -1.02) | 3.65 (3.14; 4.25) | 5.75 (4.56; 7.01) | 54.81 (54.42; 55.20) | 3.22 (2.73; 3.79) | 4.85 (3.89; 5.90) | 47.80 (47.44; 48.17) | 0.00 (0.00; 0.01) | 0.01 (0.00; 0.01) | 54.03 (52.69; 55.37) |
| **Turkmenistan** | 35.05 (29.55; 41.12) | 34.47 (29.03; 40.42) | -1.73 (-1.96; -1.50) | 32.24 (27.71; 36.58) | 32.66 (27.97; 37.08) | 1.09 (0.89; 1.29) | 34.51 (15.14; 66.52) | 33.95 (14.89; 65.42) | -1.72 (-2.76; -0.67) | 1.81 (1.52; 2.13) | 2.36 (1.90; 2.87) | 28.86 (28.53; 29.19) | 1.69 (1.44; 1.91) | 2.15 (1.76; 2.59) | 25.73 (25.43; 26.04) | 0.00 (0.00; 0.00) | 0.00 (0.00; 0.00) | 28.23 (26.97; 29.48) |
| **Uzbekistan** | 36.47 (31.05; 42.72) | 35.83 (30.54; 41.98) | -1.83 (-2.05; -1.60) | 31.69 (26.91; 36.68) | 31.62 (26.89; 36.65) | -0.37 (-0.59; -0.16) | 35.87 (15.82; 68.25) | 35.25 (15.53; 67.07) | -1.80 (-2.83; -0.78) | 12.51 (10.57; 14.72) | 16.59 (11.09; 22.46) | 32.14 (31.58; 32.69) | 10.85 (9.23; 12.65) | 14.00 (9.40; 18.80) | 28.67 (28.13; 29.20) | 0.01 (0.01; 0.02) | 0.02 (0.01; 0.03) | 31.35 (30.08; 32.61) |
| **Central Europe** | 35.60 (31.05; 40.32) | 35.10 (30.65; 39.93) | -1.51 (-1.69; -1.32) | 32.34 (28.38; 36.19) | 32.76 (28.71; 36.70) | 1.15 (0.97; 1.32) | 35.05 (15.67; 66.02) | 34.56 (15.53; 65.05) | -1.49 (-2.50; -0.49) | 44.31 (38.62; 49.62) | 34.83 (30.12; 39.05) | -19.64 (-19.80; -19.47) | 36.23 (32.32; 40.77) | 28.70 (25.31; 32.72) | -19.05 (-19.21; -18.88) | 0.04 (0.02; 0.08) | 0.03 (0.02; 0.07) | -20.15 (-21.11; -19.20) |
| **Albania** | 38.83 (33.23; 44.72) | 38.31 (32.77; 44.11) | -1.44 (-1.65; -1.24) | 31.55 (26.75; 36.17) | 32.22 (27.25; 37.03) | 1.89 (1.67; 2.10) | 38.29 (16.76; 71.52) | 37.77 (16.55; 70.63) | -1.43 (-2.44; -0.43) | 1.12 (0.96; 1.28) | 0.95 (0.75; 1.18) | -14.84 (-15.11; -14.56) | 0.84 (0.72; 0.97) | 0.71 (0.55; 0.89) | -14.85 (-15.14; -14.56) | 0.00 (0.00; 0.00) | 0.00 (0.00; 0.00) | -15.38 (-16.37; -14.39) |
| **Bosnia and Herzegovina** | 38.62 (33.30; 44.25) | 37.98 (32.74; 43.53) | -1.72 (-1.92; -1.52) | 31.74 (27.38; 36.43) | 32.49 (27.98; 37.28) | 2.13 (1.93; 2.34) | 38.00 (16.73; 70.69) | 37.38 (16.46; 69.52) | -1.71 (-2.70; -0.71) | 1.38 (1.19; 1.57) | 0.91 (0.68; 1.14) | -33.42 (-33.67; -33.17) | 1.03 (0.90; 1.18) | 0.69 (0.53; 0.87) | -32.21 (-32.46; -31.97) | 0.00 (0.00; 0.00) | 0.00 (0.00; 0.00) | -34.26 (-35.14; -33.37) |
| **Bulgaria** | 39.87 (34.54; 45.44) | 39.47 (34.19; 45.02) | -1.06 (-1.25; -0.87) | 33.94 (29.32; 38.71) | 34.31 (29.60; 39.14) | 0.89 (0.70; 1.09) | 39.21 (17.40; 74.10) | 38.83 (17.22; 73.39) | -1.06 (-2.07; -0.04) | 2.94 (2.52; 3.31) | 2.05 (1.69; 2.40) | -29.25 (-29.46; -29.04) | 2.27 (1.99; 2.60) | 1.63 (1.36; 1.93) | -27.25 (-27.46; -27.04) | 0.00 (0.00; 0.01) | 0.00 (0.00; 0.00) | -29.52 (-30.44; -28.60) |
| **Croatia** | 40.55 (34.54; 46.28) | 40.10 (34.15; 45.76) | -1.20 (-1.41; -1.00) | 30.99 (26.75; 35.58) | 30.40 (26.22; 34.88) | -1.88 (-2.08; -1.68) | 39.94 (17.78; 75.40) | 39.49 (17.59; 74.56) | -1.20 (-2.21; -0.18) | 1.84 (1.57; 2.08) | 1.24 (0.99; 1.51) | -32.01 (-32.24; -31.77) | 1.27 (1.11; 1.45) | 0.84 (0.66; 1.03) | -33.14 (-33.38; -32.90) | 0.00 (0.00; 0.00) | 0.00 (0.00; 0.00) | -32.69 (-33.58; -31.80) |
| **Czechia** | 37.49 (32.08; 43.25) | 37.21 (31.85; 42.92) | -0.86 (-1.07; -0.65) | 31.92 (27.63; 36.81) | 32.03 (27.71; 36.99) | 0.19 (-0.01; 0.40) | 36.87 (16.33; 69.93) | 36.59 (16.21; 69.43) | -0.86 (-1.88; 0.17) | 4.22 (3.58; 4.80) | 3.65 (3.03; 4.37) | -13.25 (-13.49; -13) | 3.28 (2.87; 3.79) | 2.91 (2.43; 3.43) | -11.21 (-11.44; -10.98) | 0.00 (0.00; 0.01) | 0.00 (0.00; 0.01) | -13.75 (-14.68; -12.81) |
| **Hungary** | 34.91 (29.66; 41.09) | 34.53 (29.34; 40.63) | -1.19 (-1.42; -0.96) | 32.28 (27.58; 36.82) | 33.33 (28.49; 38.04) | 2.90 (2.70; 3.11) | 34.41 (15.35; 66.05) | 34.04 (15.19; 65.36) | -1.18 (-2.21; -0.14) | 3.57 (3.00; 4.13) | 3.29 (2.66; 3.96) | -7.46 (-7.74; -7.18) | 2.99 (2.60; 3.40) | 2.89 (2.42; 3.45) | -3.37 (-3.62; -3.11) | 0.00 (0.00; 0.01) | 0.00 (0.00; 0.01) | -7.64 (-8.68; -6.61) |
| **Montenegro** | 39.72 (34.25; 45.56) | 39.26 (33.86; 45.07) | -1.24 (-1.44; -1.04) | 31.12 (26.76; 36.01) | 31.37 (26.99; 36.31) | 0.62 (0.40; 0.83) | 39.13 (16.94; 72.93) | 38.68 (16.77; 72.06) | -1.23 (-2.23; -0.22) | 0.26 (0.23; 0.30) | 0.22 (0.18; 0.26) | -17.02 (-17.26; -16.78) | 0.19 (0.17; 0.22) | 0.16 (0.13; 0.19) | -17.14 (-17.40; -16.88) | 0.00 (0.00; 0.00) | 0.00 (0.00; 0.00) | -17.57 (-18.51; -16.63) |
| **North Macedonia** | 39.38 (33.87; 45.33) | 38.93 (33.49; 44.81) | -1.25 (-1.46; -1.05) | 31.52 (26.99; 35.90) | 31.71 (27.11; 36.15) | 0.45 (0.24; 0.65) | 38.77 (17.11; 73.05) | 38.32 (16.91; 72.26) | -1.24 (-2.26; -0.23) | 0.95 (0.82; 1.09) | 0.74 (0.56; 0.91) | -21.90 (-22.19; -21.61) | 0.69 (0.59; 0.80) | 0.53 (0.41; 0.67) | -23.36 (-23.66; -23.06) | 0.00 (0.00; 0.00) | 0.00 (0.00; 0.00) | -22.71 (-23.65; -21.76) |
| **Poland** | 29.72 (26.12; 33.87) | 29.33 (25.76; 33.42) | -1.42 (-1.60; -1.24) | 33.48 (29.87; 36.91) | 33.66 (30.04; 37.12) | 0.33 (0.18; 0.48) | 29.26 (13.24; 56.49) | 28.87 (13.07; 55.71) | -1.41 (-2.45; -0.37) | 12.35 (10.90; 13.77) | 9.98 (8.44; 11.47) | -18.64 (-18.84; -18.44) | 12.30 (11.06; 13.54) | 9.68 (8.34; 11.06) | -20.67 (-20.85; -20.49) | 0.01 (0.01; 0.02) | 0.01 (0.00; 0.02) | -19.31 (-20.33; -18.28) |
| **Romania** | 41.32 (35.54; 46.92) | 40.85 (35.14; 46.38) | -1.21 (-1.41; -1.02) | 32.15 (28.00; 37.42) | 33.86 (29.39; 39.37) | 4.90 (4.69; 5.11) | 40.69 (18.26; 75.23) | 40.23 (18.07; 74.39) | -1.21 (-2.19; -0.23) | 8.44 (7.28; 9.46) | 6.39 (5.11; 7.69) | -23.52 (-23.77; -23.28) | 5.98 (5.24; 6.94) | 4.78 (3.85; 5.83) | -19.45 (-19.71; -19.20) | 0.01 (0.00; 0.02) | 0.01 (0.00; 0.01) | -23.90 (-24.84; -22.96) |
| **Serbia** | 37.48 (31.75; 43.41) | 36.98 (31.38; 42.78) | -1.44 (-1.66; -1.22) | 28.30 (24.38; 32.44) | 26.41 (22.68; 30.47) | -6.39 (-6.59; -6.19) | 36.93 (16.57; 69.89) | 36.43 (16.34; 68.99) | -1.43 (-2.44; -0.41) | 3.63 (3.12; 4.18) | 2.76 (2.23; 3.31) | -23.43 (-23.68; -23.19) | 2.50 (2.16; 2.87) | 1.69 (1.36; 2.04) | -31.58 (-31.83; -31.32) | 0.00 (0.00; 0.01) | 0.00 (0.00; 0.01) | -24.02 (-24.96; -23.08) |
| **Slovakia** | 37.34 (31.91; 43.28) | 37.07 (31.68; 42.99) | -0.84 (-1.05; -0.62) | 31.69 (27.27; 36.56) | 31.94 (27.50; 36.88) | 0.61 (0.40; 0.82) | 36.80 (16.29; 69.88) | 36.53 (16.18; 69.36) | -0.83 (-1.86; 0.19) | 2.19 (1.87; 2.52) | 1.92 (1.61; 2.27) | -12.02 (-12.25; -11.78) | 1.68 (1.46; 1.95) | 1.50 (1.27; 1.77) | -10.61 (-10.84; -10.38) | 0.00 (0.00; 0.00) | 0.00 (0.00; 0.00) | -12.60 (-13.62; -11.57) |
| **Slovenia** | 35.08 (29.61; 40.77) | 34.73 (29.31; 40.37) | -1.09 (-1.32; -0.87) | 34.22 (29.38; 39.12) | 35.72 (30.69; 40.81) | 3.97 (3.76; 4.17) | 34.58 (14.90; 67.41) | 34.24 (14.75; 66.79) | -1.09 (-2.16; -0.02) | 0.77 (0.65; 0.88) | 0.74 (0.63; 0.86) | -3.56 (-3.79; -3.34) | 0.69 (0.60; 0.78) | 0.71 (0.60; 0.82) | 2.74 (2.50; 2.97) | 0.00 (0.00; 0.00) | 0.00 (0.00; 0.00) | -3.99 (-5.03; -2.96) |
| **Eastern Europe** | 30.41 (26.11; 35.46) | 30.26 (25.98; 35.18) | -0.64 (-0.86; -0.42) | 33.78 (29.49; 38.09) | 34.21 (29.86; 38.46) | 1.04 (0.86; 1.22) | 29.82 (13.21; 57.55) | 29.67 (13.18; 57.28) | -0.63 (-1.68; 0.41) | 69.44 (59.34; 79.72) | 60.49 (50.87; 71.39) | -12.44 (-12.67; -12.21) | 68.01 (59.76; 76.36) | 57.55 (48.83; 66.50) | -14.92 (-15.12; -14.72) | 0.07 (0.03; 0.13) | 0.06 (0.03; 0.11) | -13.03 (-14.01; -12.06) |
| **Belarus** | 31.33 (26.24; 37.02) | 31.00 (25.93; 36.63) | -1.19 (-1.43; -0.95) | 31.50 (26.71; 36.31) | 31.71 (26.91; 36.53) | 0.47 (0.26; 0.69) | 30.80 (13.40; 58.09) | 30.48 (13.25; 57.49) | -1.18 (-2.20; -0.16) | 3.17 (2.64; 3.68) | 2.70 (2.16; 3.28) | -14.27 (-14.55; -14) | 2.82 (2.39; 3.27) | 2.40 (1.92; 2.92) | -14.78 (-15.06; -14.49) | 0.00 (0.00; 0.01) | 0.00 (0.00; 0.01) | -14.72 (-15.70; -13.73) |
| **Estonia** | 20.80 (16.45; 25.97) | 20.59 (16.28; 25.72) | -1.14 (-1.46; -0.82) | 27.38 (22.54; 33.02) | 27.56 (22.68; 33.21) | 0.43 (0.16; 0.70) | 20.55 (8.92; 39.81) | 20.34 (8.83; 39.39) | -1.14 (-2.20; -0.09) | 0.28 (0.22; 0.34) | 0.25 (0.19; 0.30) | -10.83 (-11.14; -10.52) | 0.33 (0.27; 0.39) | 0.29 (0.24; 0.36) | -10.99 (-11.27; -10.71) | 0.00 (0.00; 0.00) | 0.00 (0.00; 0.00) | -11.16 (-12.18; -10.13) |
| **Latvia** | 30.78 (25.76; 36.61) | 30.52 (25.55; 36.32) | -0.93 (-1.18; -0.68) | 31.76 (26.80; 36.88) | 32.00 (26.95; 37.20) | 0.53 (0.30; 0.75) | 30.28 (13.19; 58.87) | 30.03 (13.07; 58.38) | -0.93 (-1.99; 0.14) | 0.61 (0.51; 0.71) | 0.43 (0.34; 0.52) | -29.18 (-29.42; -28.94) | 0.55 (0.47; 0.65) | 0.38 (0.31; 0.46) | -29.73 (-29.96; -29.51) | 0.00 (0.00; 0.00) | 0.00 (0.00; 0.00) | -29.53 (-30.47; -28.59) |
| **Lithuania** | 34.67 (29.67; 40.31) | 34.31 (29.38; 39.88) | -1.13 (-1.34; -0.91) | 33.53 (28.57; 38.38) | 35.09 (29.86; 40.26) | 4.16 (3.95; 4.37) | 34.08 (15.11; 65.42) | 33.73 (14.95; 64.82) | -1.12 (-2.16; -0.08) | 1.03 (0.88; 1.18) | 0.70 (0.56; 0.84) | -31.15 (-31.38; -30.92) | 0.88 (0.76; 1.00) | 0.60 (0.49; 0.73) | -30.74 (-30.96; -30.52) | 0.00 (0.00; 0.00) | 0.00 (0.00; 0.00) | -31.61 (-32.50; -30.72) |
| **Republic of Moldova** | 33.13 (27.44; 39.40) | 32.80 (27.17; 39.01) | -1.11 (-1.37; -0.86) | 31.41 (26.44; 36.31) | 31.43 (26.45; 36.32) | -0.10 (-0.32; 0.12) | 32.64 (13.99; 62.11) | 32.31 (13.85; 61.48) | -1.11 (-2.15; -0.07) | 1.33 (1.10; 1.56) | 0.78 (0.54; 1.03) | -40.19 (-40.50; -39.88) | 1.11 (0.96; 1.29) | 0.60 (0.40; 0.81) | -44.64 (-44.93; -44.35) | 0.00 (0.00; 0.00) | 0.00 (0.00; 0.00) | -41.18 (-42.06; -40.30) |
| **Russian Federation** | 30.94 (26.71; 35.76) | 30.69 (26.50; 35.48) | -0.94 (-1.15; -0.74) | 33.96 (29.72; 38.23) | 34.45 (30.13; 38.74) | 1.20 (1.02; 1.38) | 30.32 (13.58; 58.61) | 30.07 (13.46; 58.14) | -0.93 (-1.98; 0.11) | 49.37 (42.64; 56.43) | 45.91 (37.98; 55.27) | -6.68 (-6.94; -6.41) | 47.83 (42.27; 53.79) | 43.69 (36.34; 50.76) | -8.36 (-8.59; -8.12) | 0.05 (0.02; 0.09) | 0.04 (0.02; 0.09) | -7.24 (-8.26; -6.23) |
| **Ukraine** | 28.27 (23.85; 33.75) | 28.18 (23.77; 33.66) | -0.52 (-0.77; -0.27) | 34.10 (29.31; 38.75) | 34.11 (29.33; 38.73) | -0.18 (-0.37; 0.02) | 27.72 (11.93; 52.85) | 27.64 (11.89; 52.67) | -0.52 (-1.56; 0.53) | 13.65 (11.32; 16.05) | 9.72 (7.25; 12.43) | -28.18 (-28.50; -27.86) | 14.49 (12.61; 16.42) | 9.59 (7.36; 12.01) | -33.09 (-33.37; -32.81) | 0.01 (0.01; 0.03) | 0.01 (0.00; 0.02) | -28.89 (-29.82; -27.96) |
| **High-income** | 25.01 (22.20; 28.65) | 25.02 (22.25; 28.73) | -0.19 (-0.38; -0.01) | 30.22 (26.80; 33.78) | 30.79 (27.29; 34.41) | 1.60 (1.44; 1.77) | 24.54 (10.96; 46.60) | 24.54 (11.00; 46.55) | -0.24 (-1.26; 0.79) | 299.05 (267.81; 334.61) | 311.60 (280.81; 347.55) | 3.71 (3.55; 3.86) | 309.62 (278.31; 343.63) | 311.48 (277.76; 348.51) | 0.30 (0.14; 0.46) | 0.29 (0.13; 0.55) | 0.30 (0.14; 0.57) | 3.14 (2.12; 4.17) |
| **Australasia** | 25.92 (21.53; 31.39) | 25.58 (21.26; 30.96) | -1.44 (-1.71; -1.17) | 29.33 (25.15; 33.97) | 29.91 (25.81; 34.51) | 1.68 (1.47; 1.89) | 25.38 (11.24; 48.41) | 25.06 (11.08; 47.84) | -1.43 (-2.45; -0.40) | 8.82 (7.36; 10.41) | 11.99 (9.94; 14.23) | 34.01 (33.71; 34.32) | 8.94 (7.73; 10.36) | 12.23 (10.50; 14.37) | 34.83 (34.56; 35.09) | 0.01 (0.00; 0.02) | 0.01 (0.01; 0.02) | 33.52 (32.29; 34.75) |
| **Australia** | 26.41 (21.84; 32.00) | 26.05 (21.52; 31.55) | -1.50 (-1.77; -1.23) | 28.79 (24.57; 33.52) | 29.53 (25.29; 34.25) | 2.26 (2.04; 2.48) | 25.86 (11.39; 49.35) | 25.51 (11.22; 48.72) | -1.48 (-2.51; -0.45) | 7.49 (6.23; 8.87) | 10.20 (8.39; 12.20) | 34.23 (33.92; 34.54) | 7.29 (6.29; 8.48) | 10.09 (8.62; 11.91) | 36.48 (36.20; 36.76) | 0.01 (0.00; 0.01) | 0.01 (0.00; 0.02) | 33.74 (32.52; 34.97) |
| **New Zealand** | 23.48 (19.33; 28.25) | 23.24 (19.15; 27.95) | -1.18 (-1.44; -0.91) | 31.97 (26.95; 37.20) | 31.79 (26.78; 36.97) | -0.76 (-0.99; -0.54) | 23.01 (10.12; 44.53) | 22.78 (10.02; 44.10) | -1.17 (-2.22; -0.12) | 1.33 (1.10; 1.57) | 1.79 (1.46; 2.14) | 32.78 (32.46; 33.10) | 1.65 (1.40; 1.91) | 2.14 (1.81; 2.53) | 27.57 (27.30; 27.85) | 0.00 (0.00; 0.00) | 0.00 (0.00; 0.00) | 32.26 (31.01; 33.50) |
| **High-income Asia Pacific** | 14.59 (12.66; 17.04) | 14.36 (12.48; 16.71) | -1.85 (-2.06; -1.64) | 28.45 (25.06; 31.64) | 28.56 (25.14; 31.90) | 0.09 (-0.08; 0.26) | 14.49 (6.49; 28.15) | 14.24 (6.38; 27.81) | -1.93 (-2.98; -0.88) | 25.62 (22.44; 29.25) | 21.30 (18.46; 24.23) | -16.73 (-16.92; -16.55) | 47.16 (42.06; 52.28) | 38.27 (33.54; 43.23) | -18.64 (-18.79; -18.48) | 0.03 (0.01; 0.05) | 0.02 (0.01; 0.04) | -17.54 (-18.50; -16.58) |
| **Brunei Darussalam** | 16.52 (13.49; 20.02) | 16.26 (13.27; 19.73) | -1.73 (-2.01; -1.45) | 32.25 (27.20; 37.51) | 32.12 (27.13; 37.33) | -0.61 (-0.83; -0.38) | 16.35 (7.18; 31.02) | 16.10 (7.06; 30.57) | -1.75 (-2.77; -0.73) | 0.08 (0.07; 0.10) | 0.07 (0.06; 0.09) | -11.26 (-11.60; -10.91) | 0.16 (0.13; 0.18) | 0.14 (0.10; 0.17) | -15.78 (-16.08; -15.49) | 0.00 (0.00; 0.00) | 0.00 (0.00; 0.00) | -12.48 (-13.46; -11.50) |
| **Japan** | 11.76 (10.21; 13.64) | 11.65 (10.11; 13.49) | -1.20 (-1.40; -0.99) | 24.61 (21.57; 27.75) | 24.75 (21.66; 27.88) | 0.27 (0.09; 0.45) | 11.70 (5.26; 22.92) | 11.58 (5.20; 22.70) | -1.21 (-2.28; -0.15) | 13.03 (11.39; 14.80) | 10.32 (8.85; 11.96) | -20.48 (-20.66; -20.29) | 26.16 (23.27; 29.39) | 20.77 (18.00; 23.91) | -20.30 (-20.48; -20.12) | 0.01 (0.01; 0.03) | 0.01 (0.00; 0.02) | -20.80 (-21.74; -19.87) |
| **Republic of Korea** | 20.63 (17.63; 24.17) | 20.38 (17.40; 23.87) | -1.38 (-1.60; -1.15) | 36.41 (31.92; 40.69) | 36.98 (32.37; 41.37) | 1.30 (1.13; 1.48) | 20.41 (8.93; 39.05) | 20.16 (8.82; 38.55) | -1.38 (-2.42; -0.35) | 11.53 (9.79; 13.41) | 9.70 (8.03; 11.52) | -15.96 (-16.19; -15.72) | 19.00 (16.68; 21.39) | 15.10 (12.72; 17.63) | -20.35 (-20.55; -20.16) | 0.01 (0.00; 0.02) | 0.01 (0.00; 0.02) | -17.30 (-18.28; -16.33) |
| **Singapore** | 16.44 (13.34; 19.90) | 16.20 (13.16; 19.58) | -1.69 (-1.97; -1.41) | 32.33 (27.69; 37.44) | 32.67 (27.98; 37.78) | 0.78 (0.57; 0.99) | 16.33 (7.07; 32.04) | 16.09 (6.95; 31.56) | -1.70 (-2.77; -0.63) | 0.98 (0.79; 1.18) | 1.21 (1.00; 1.45) | 23.06 (22.74; 23.37) | 1.84 (1.54; 2.14) | 2.26 (1.88; 2.64) | 22.15 (21.88; 22.43) | 0.00 (0.00; 0.00) | 0.00 (0.00; 0.00) | 22.14 (20.96; 23.33) |
| **High-income North America** | 19.28 (16.33; 22.89) | 19.12 (16.20; 22.71) | -0.98 (-1.21; -0.74) | 30.90 (26.81; 34.83) | 30.95 (26.77; 34.89) | -0.07 (-0.25; 0.12) | 18.85 (8.43; 36.61) | 18.70 (8.37; 36.33) | -0.95 (-2.00; 0.10) | 76.33 (65.61; 87.75) | 82.14 (69.12; 96.57) | 7.08 (6.83; 7.32) | 111.27 (97.36; 125.02) | 116.81 (101.39; 134.40) | 4.57 (4.36; 4.78) | 0.07 (0.03; 0.14) | 0.08 (0.04; 0.15) | 6.65 (5.56; 7.73) |
| **Canada** | 18.82 (15.58; 22.94) | 18.67 (15.46; 22.75) | -0.96 (-1.24; -0.69) | 27.33 (22.56; 32.03) | 27.40 (22.61; 32.13) | 0 (-0.24; 0.25) | 18.51 (8.12; 35.72) | 18.36 (8.06; 35.48) | -0.96 (-2.01; 0.09) | 7.67 (6.34; 9.16) | 9.87 (8.10; 11.86) | 26.99 (26.69; 27.30) | 9.75 (8.16; 11.26) | 12.52 (10.47; 14.67) | 26.66 (26.39; 26.93) | 0.01 (0.00; 0.01) | 0.01 (0.00; 0.02) | 26.66 (25.45; 27.87) |
| **Greenland** | 18.13 (14.62; 21.97) | 17.87 (14.42; 21.68) | -1.53 (-1.81; -1.24) | 26.97 (23.00; 32.12) | 26.98 (22.99; 32.14) | -0.16 (-0.40; 0.07) | 17.80 (7.73; 34.19) | 17.55 (7.63; 33.73) | -1.54 (-2.58; -0.49) | 0.01 (0.01; 0.01) | 0.01 (0.01; 0.01) | 3.69 (3.37; 4.00) | 0.02 (0.01; 0.02) | 0.02 (0.01; 0.02) | -0.52 (-0.80; -0.24) | 0.00 (0.00; 0.00) | 0.00 (0.00; 0.00) | 2.94 (1.87; 4.01) |
| **United States of America** | 19.32 (16.43; 22.80) | 19.18 (16.33; 22.63) | -0.89 (-1.12; -0.66) | 31.29 (27.30; 35.23) | 31.44 (27.42; 35.38) | 0.24 (0.06; 0.42) | 18.88 (8.42; 36.56) | 18.74 (8.36; 36.30) | -0.87 (-1.92; 0.18) | 68.66 (59.13; 78.95) | 72.26 (61.02; 84.51) | 4.83 (4.59; 5.07) | 101.51 (89.02; 114.05) | 104.28 (90.77; 120.87) | 2.43 (2.22; 2.64) | 0.07 (0.03; 0.13) | 0.07 (0.03; 0.13) | 4.37 (3.30; 5.45) |
| **Southern Latin America** | 41.97 (37.63; 46.39) | 41.44 (37.21; 45.71) | -1.39 (-1.54; -1.25) | 30.25 (26.67; 34.58) | 30.26 (26.77; 34.49) | -0.12 (-0.30; 0.06) | 41.10 (18.54; 78.13) | 40.58 (18.34; 77.22) | -1.38 (-2.40; -0.36) | 30.61 (27.57; 33.62) | 34.50 (30.31; 39.06) | 11.62 (11.43; 11.82) | 20.73 (18.30; 23.74) | 20.41 (17.43; 23.83) | -2.06 (-2.27; -1.84) | 0.03 (0.01; 0.06) | 0.03 (0.02; 0.06) | 10.73 (9.65; 11.80) |
| **Argentina** | 41.73 (35.95; 47.65) | 41.15 (35.50; 47.03) | -1.52 (-1.71; -1.32) | 30.43 (26.02; 35.64) | 30.51 (26.09; 35.85) | 0.10 (-0.13; 0.32) | 40.88 (18.12; 76.52) | 40.31 (17.88; 75.45) | -1.50 (-2.50; -0.50) | 20.24 (17.51; 23.00) | 22.40 (18.41; 26.88) | 9.68 (9.40; 9.96) | 14.08 (12.07; 16.54) | 13.69 (11.14; 16.77) | -3.32 (-3.60; -3.04) | 0.02 (0.01; 0.04) | 0.02 (0.01; 0.04) | 8.84 (7.75; 9.93) |
| **Chile** | 42.65 (40.98; 44.54) | 42.23 (40.59; 44.09) | -1.11 (-1.17; -1.05) | 29.83 (26.16; 33.09) | 29.57 (25.80; 32.82) | -1.00 (-1.16; -0.83) | 41.74 (19.67; 79.63) | 41.33 (19.47; 78.89) | -1.10 (-2.11; -0.09) | 8.86 (8.60; 9.14) | 10.64 (9.76; 11.71) | 18.58 (18.43; 18.73) | 5.62 (5.02; 6.19) | 5.80 (4.99; 6.72) | 2.48 (2.27; 2.69) | 0.01 (0.00; 0.02) | 0.01 (0.00; 0.02) | 17.52 (16.40; 18.65) |
| **Uruguay** | 40.71 (35.71; 46.54) | 40.13 (35.28; 45.86) | -1.52 (-1.71; -1.34) | 30.48 (26.10; 35.23) | 31.63 (27.11; 36.54) | 3.49 (3.27; 3.70) | 39.90 (17.89; 73.87) | 39.34 (17.64; 72.91) | -1.50 (-2.49; -0.52) | 1.51 (1.34; 1.72) | 1.46 (1.20; 1.73) | -3.45 (-3.70; -3.20) | 1.03 (0.88; 1.18) | 0.92 (0.75; 1.13) | -9.67 (-9.94; -9.40) | 0.00 (0.00; 0.00) | 0.00 (0.00; 0.00) | -4.04 (-5.06; -3.02) |
| **Western Europe** | 31.59 (28.23; 35.78) | 31.37 (28.10; 35.48) | -0.87 (-1.03; -0.70) | 30.50 (27.07; 33.94) | 31.53 (28.03; 35.10) | 3.05 (2.89; 3.21) | 30.99 (14.07; 59.26) | 30.77 (13.98; 58.70) | -0.86 (-1.88; 0.17) | 157.66 (141.79; 175.17) | 161.66 (145.39; 178.24) | 2.12 (1.97; 2.27) | 121.53 (108.94; 134.79) | 123.77 (110.84; 138.18) | 1.52 (1.36; 1.68) | 0.15 (0.07; 0.29) | 0.16 (0.07; 0.29) | 1.61 (0.60; 2.63) |
| **Andorra** | 33.50 (28.15; 38.97) | 33.35 (28.01; 38.78) | -0.60 (-0.83; -0.37) | 30.90 (26.35; 36.42) | 31.10 (26.54; 36.67) | 0.43 (0.20; 0.66) | 32.82 (14.64; 62.18) | 32.67 (14.58; 61.91) | -0.60 (-1.62; 0.42) | 0.03 (0.03; 0.04) | 0.03 (0.03; 0.04) | -14.13 (-14.37; -13.89) | 0.03 (0.02; 0.03) | 0.02 (0.02; 0.02) | -27.25 (-27.49; -27.02) | 0.00 (0.00; 0.00) | 0.00 (0.00; 0.00) | -15.69 (-16.64; -14.73) |
| **Austria** | 29.46 (25.21; 34.89) | 29.29 (25.09; 34.66) | -0.72 (-0.95; -0.49) | 30.29 (25.55; 35.24) | 30.65 (25.88; 35.62) | 0.94 (0.71; 1.17) | 28.94 (12.81; 55.91) | 28.77 (12.75; 55.64) | -0.72 (-1.77; 0.33) | 3.07 (2.61; 3.55) | 3.34 (2.83; 3.95) | 8.51 (8.26; 8.77) | 2.58 (2.20; 2.97) | 2.74 (2.30; 3.19) | 6.33 (6.09; 6.57) | 0.00 (0.00; 0.01) | 0.00 (0.00; 0.01) | 7.93 (6.83; 9.04) |
| **Belgium** | 26.68 (21.77; 32.46) | 26.33 (21.50; 32.03) | -1.43 (-1.71; -1.15) | 28.40 (23.34; 33.39) | 28.64 (23.56; 33.60) | 0.60 (0.35; 0.85) | 26.16 (11.57; 49.08) | 25.82 (11.42; 48.44) | -1.41 (-2.42; -0.41) | 3.47 (2.87; 4.08) | 3.87 (3.13; 4.70) | 11.03 (10.73; 11.34) | 3.05 (2.50; 3.51) | 3.40 (2.77; 4.05) | 11.28 (11; 11.57) | 0.00 (0.00; 0.01) | 0.00 (0.00; 0.01) | 10.67 (9.59; 11.76) |
| **Cyprus** | 34.65 (29.34; 40.43) | 34.21 (28.98; 39.88) | -1.39 (-1.62; -1.17) | 30.90 (26.30; 36.17) | 31.19 (26.57; 36.46) | 0.73 (0.50; 0.95) | 33.99 (15.22; 65.95) | 33.56 (15.02; 65.13) | -1.37 (-2.42; -0.32) | 0.54 (0.46; 0.62) | 0.64 (0.52; 0.76) | 15.61 (15.31; 15.91) | 0.41 (0.36; 0.48) | 0.43 (0.36; 0.52) | 4.54 (4.26; 4.83) | 0.00 (0.00; 0.00) | 0.00 (0.00; 0.00) | 14.59 (13.46; 15.72) |
| **Denmark** | 21.99 (18.07; 26.28) | 21.78 (17.89; 26.03) | -1.08 (-1.34; -0.82) | 24.98 (21.45; 29.02) | 25.51 (21.93; 29.57) | 1.81 (1.59; 2.02) | 21.68 (9.81; 43.75) | 21.48 (9.72; 43.34) | -1.08 (-2.18; 0.03) | 1.35 (1.09; 1.61) | 1.48 (1.18; 1.76) | 8.71 (8.42; 9.00) | 1.26 (1.09; 1.45) | 1.38 (1.18; 1.63) | 9.29 (9.05; 9.53) | 0.00 (0.00; 0.00) | 0.00 (0.00; 0.00) | 8.55 (7.35; 9.76) |
| **Finland** | 22.81 (18.77; 27.58) | 22.54 (18.55; 27.24) | -1.32 (-1.59; -1.05) | 22.48 (18.51; 27.50) | 22.26 (18.29; 27.15) | -1.19 (-1.47; -0.91) | 22.37 (9.67; 43.24) | 22.11 (9.55; 42.74) | -1.32 (-2.38; -0.27) | 1.43 (1.19; 1.69) | 1.39 (1.12; 1.67) | -3.26 (-3.54; -2.98) | 1.13 (0.94; 1.34) | 1.05 (0.85; 1.27) | -7.33 (-7.61; -7.06) | 0.00 (0.00; 0.00) | 0.00 (0.00; 0.00) | -3.61 (-4.66; -2.55) |
| **France** | 39.06 (33.72; 44.58) | 38.58 (33.33; 44.03) | -1.34 (-1.54; -1.15) | 33.66 (29.09; 38.62) | 35.26 (30.50; 40.49) | 4.40 (4.19; 4.60) | 38.18 (17.03; 73.09) | 37.71 (16.83; 72.30) | -1.33 (-2.36; -0.30) | 29.89 (26.44; 33.38) | 29.38 (24.94; 34.34) | -2.10 (-2.32; -1.88) | 21.25 (18.66; 24.14) | 20.79 (17.48; 24.51) | -2.46 (-2.69; -2.23) | 0.03 (0.01; 0.05) | 0.03 (0.01; 0.05) | -2.68 (-3.70; -1.66) |
| **Germany** | 28.27 (24.11; 33.30) | 28.06 (23.94; 33.06) | -0.87 (-1.10; -0.64) | 27.59 (24.06; 32.17) | 28.39 (24.72; 33.13) | 2.57 (2.36; 2.78) | 27.82 (12.59; 53.92) | 27.61 (12.50; 53.49) | -0.87 (-1.91; 0.18) | 26.11 (21.77; 30.52) | 25.77 (20.98; 30.80) | -1.52 (-1.79; -1.25) | 19.44 (16.85; 22.35) | 19.59 (16.52; 23.11) | 0.70 (0.45; 0.95) | 0.03 (0.01; 0.05) | 0.02 (0.01; 0.05) | -1.79 (-2.84; -0.74) |
| **Greece** | 37.62 (32.25; 43.33) | 37.08 (31.78; 42.69) | -1.53 (-1.73; -1.32) | 31.09 (26.28; 36.43) | 35.40 (29.84; 41.80) | 12.94 (12.69; 13.19) | 36.89 (16.39; 70.78) | 36.36 (16.16; 69.73) | -1.51 (-2.55; -0.48) | 4.55 (3.96; 5.12) | 3.56 (2.89; 4.26) | -21.27 (-21.51; -21.03) | 2.91 (2.51; 3.37) | 2.30 (1.83; 2.86) | -20.36 (-20.63; -20.10) | 0.00 (0.00; 0.01) | 0.00 (0.00; 0.01) | -21.85 (-22.82; -20.89) |
| **Iceland** | 34.24 (29.17; 39.98) | 34.00 (28.95; 39.72) | -0.84 (-1.07; -0.62) | 26.50 (23.13; 30.85) | 27.99 (24.39; 32.52) | 5.21 (5.00; 5.43) | 33.77 (15.37; 65.81) | 33.52 (15.25; 65.33) | -0.85 (-1.90; 0.21) | 0.13 (0.11; 0.15) | 0.18 (0.15; 0.21) | 36.57 (36.29; 36.85) | 0.09 (0.07; 0.10) | 0.12 (0.10; 0.14) | 38.53 (38.24; 38.82) | 0.00 (0.00; 0.00) | 0.00 (0.00; 0.00) | 36.09 (34.79; 37.39) |
| **Ireland** | 34.15 (28.68; 39.86) | 33.82 (28.41; 39.47) | -1.10 (-1.33; -0.87) | 31.18 (26.47; 36.48) | 32.00 (27.18; 37.49) | 2.35 (2.12; 2.58) | 33.44 (14.89; 62.46) | 33.13 (14.74; 61.86) | -1.08 (-2.08; -0.08) | 1.89 (1.60; 2.18) | 2.41 (1.99; 2.84) | 26.06 (25.76; 26.35) | 1.49 (1.28; 1.73) | 1.85 (1.53; 2.19) | 22.33 (22.05; 22.61) | 0.00 (0.00; 0.00) | 0.00 (0.00; 0.00) | 25.33 (24.17; 26.49) |
| **Israel** | 38.99 (33.91; 44.63) | 38.53 (33.55; 44.09) | -1.30 (-1.49; -1.11) | 33.88 (29.54; 38.75) | 35.61 (31.10; 40.75) | 4.69 (4.49; 4.88) | 38.24 (17.23; 73.70) | 37.79 (17.01; 72.87) | -1.29 (-2.33; -0.25) | 3.82 (3.32; 4.35) | 5.48 (4.53; 6.65) | 41.29 (40.95; 41.63) | 3.17 (2.77; 3.63) | 4.56 (3.62; 5.56) | 41.41 (41.04; 41.78) | 0.00 (0.00; 0.01) | 0.01 (0.00; 0.01) | 40.73 (39.46; 42) |
| **Italy** | 28.73 (24.37; 34.13) | 28.35 (24.05; 33.68) | -1.43 (-1.67; -1.19) | 30.60 (26.50; 34.85) | 31.69 (27.42; 36.15) | 3.20 (3.00; 3.40) | 28.18 (12.51; 53.58) | 27.82 (12.34; 52.87) | -1.42 (-2.44; -0.40) | 20.34 (17.21; 23.31) | 16.68 (14.04; 19.72) | -17.85 (-18.08; -17.62) | 16.37 (14.33; 18.61) | 12.70 (10.69; 14.75) | -22.03 (-22.24; -21.83) | 0.02 (0.01; 0.04) | 0.02 (0.01; 0.03) | -18.61 (-19.55; -17.67) |
| **Luxembourg** | 33.27 (28.13; 38.68) | 33.04 (27.94; 38.43) | -0.83 (-1.06; -0.61) | 31.01 (26.16; 36.54) | 31.48 (26.56; 37.03) | 1.26 (1.02; 1.50) | 32.66 (14.60; 61.77) | 32.43 (14.50; 61.37) | -0.83 (-1.85; 0.19) | 0.25 (0.21; 0.29) | 0.38 (0.31; 0.44) | 49.27 (48.96; 49.58) | 0.19 (0.17; 0.23) | 0.30 (0.25; 0.35) | 48.71 (48.40; 49.03) | 0.00 (0.00; 0.00) | 0.00 (0.00; 0.00) | 48.63 (47.30; 49.96) |
| **Malta** | 34.52 (29.00; 40.11) | 33.90 (28.46; 39.40) | -1.89 (-2.12; -1.67) | 30.93 (26.14; 35.64) | 31.61 (26.74; 36.42) | 1.89 (1.68; 2.11) | 33.88 (15.25; 63.26) | 33.28 (14.96; 62.11) | -1.88 (-2.87; -0.89) | 0.18 (0.15; 0.20) | 0.22 (0.18; 0.25) | 18.66 (18.39; 18.93) | 0.13 (0.11; 0.15) | 0.16 (0.13; 0.19) | 25.46 (25.16; 25.76) | 0.00 (0.00; 0.00) | 0.00 (0.00; 0.00) | 18.39 (17.28; 19.50) |
| **Monaco** | 31.27 (26.37; 36.89) | 31.04 (26.20; 36.65) | -0.86 (-1.10; -0.62) | 31.38 (26.56; 36.09) | 31.80 (26.92; 36.55) | 1.09 (0.88; 1.31) | 30.66 (13.44; 58.87) | 30.45 (13.34; 58.46) | -0.85 (-1.89; 0.20) | 0.01 (0.01; 0.02) | 0.01 (0.01; 0.01) | -24.75 (-24.99; -24.51) | 0.01 (0.01; 0.01) | 0.01 (0.01; 0.01) | -22.80 (-23.02; -22.59) | 0.00 (0.00; 0.00) | 0.00 (0.00; 0.00) | -25.08 (-26; -24.15) |
| **Netherlands** | 22.55 (18.63; 27.17) | 22.32 (18.46; 26.91) | -1.12 (-1.39; -0.85) | 25.30 (21.66; 29.20) | 25.66 (21.97; 29.59) | 1.13 (0.92; 1.34) | 22.29 (10.18; 44.28) | 22.07 (10.08; 43.83) | -1.11 (-2.19; -0.04) | 4.10 (3.38; 4.86) | 4.26 (3.43; 5.04) | 3.60 (3.32; 3.88) | 3.70 (3.20; 4.23) | 3.85 (3.22; 4.54) | 3.79 (3.55; 4.04) | 0.00 (0.00; 0.01) | 0.00 (0.00; 0.01) | 3.32 (2.22; 4.43) |
| **Norway** | 29.15 (25.05; 33.71) | 29.00 (24.92; 33.54) | -0.66 (-0.87; -0.46) | 33.32 (29.27; 37.38) | 33.26 (29.21; 37.34) | -0.37 (-0.54; -0.20) | 28.62 (12.91; 55.37) | 28.47 (12.84; 55.09) | -0.66 (-1.71; 0.38) | 1.81 (1.57; 2.03) | 2.13 (1.83; 2.44) | 16.93 (16.70; 17.16) | 1.75 (1.56; 1.96) | 1.92 (1.66; 2.21) | 9.24 (9.03; 9.46) | 0.00 (0.00; 0.00) | 0.00 (0.00; 0.00) | 16.34 (15.19; 17.49) |
| **Portugal** | 29.55 (24.74; 35.05) | 28.95 (24.19; 34.34) | -2.11 (-2.35; -1.86) | 30.59 (25.30; 35.71) | 32.43 (26.66; 37.95) | 5.53 (5.28; 5.78) | 28.95 (12.61; 54.97) | 28.37 (12.34; 53.94) | -2.08 (-3.11; -1.06) | 3.71 (3.11; 4.36) | 3.50 (2.83; 4.19) | -5.84 (-6.12; -5.56) | 3.01 (2.50; 3.48) | 2.93 (2.38; 3.49) | -2.85 (-3.12; -2.58) | 0.00 (0.00; 0.01) | 0.00 (0.00; 0.01) | -6.35 (-7.37; -5.32) |
| **San Marino** | 34.06 (28.98; 39.69) | 33.78 (28.70; 39.36) | -0.95 (-1.17; -0.73) | 31.08 (26.05; 36.54) | 31.60 (26.59; 37.14) | 1.42 (1.18; 1.66) | 33.38 (14.92; 64.52) | 33.11 (14.77; 63.88) | -0.91 (-1.95; 0.14) | 0.01 (0.01; 0.02) | 0.01 (0.01; 0.01) | -11.32 (-11.58; -11.06) | 0.01 (0.01; 0.01) | 0.01 (0.01; 0.01) | -17.44 (-17.71; -17.18) | 0.00 (0.00; 0.00) | 0.00 (0.00; 0.00) | -12.36 (-13.33; -11.38) |
| **Spain** | 35.68 (30.73; 40.90) | 35.15 (30.32; 40.30) | -1.56 (-1.76; -1.36) | 32.14 (27.15; 37.24) | 34.24 (28.88; 39.61) | 6.06 (5.84; 6.29) | 35.01 (15.58; 67.84) | 34.50 (15.34; 66.82) | -1.55 (-2.60; -0.50) | 19.36 (16.75; 21.73) | 21.40 (18.06; 24.63) | 9.46 (9.22; 9.69) | 13.66 (11.77; 15.86) | 15.74 (13.30; 18.46) | 13.85 (13.60; 14.11) | 0.02 (0.01; 0.04) | 0.02 (0.01; 0.04) | 8.78 (7.68; 9.89) |
| **Sweden** | 30.71 (25.67; 36.41) | 30.44 (25.46; 36.07) | -1.03 (-1.28; -0.79) | 29.43 (24.85; 34.21) | 30.56 (25.77; 35.53) | 3.50 (3.27; 3.73) | 30.18 (13.23; 57.35) | 29.91 (13.12; 56.83) | -1.03 (-2.06; 0.00) | 3.60 (3.04; 4.20) | 4.65 (3.90; 5.56) | 27.93 (27.63; 28.22) | 2.82 (2.42; 3.26) | 3.75 (3.16; 4.39) | 31.28 (31; 31.56) | 0.00 (0.00; 0.01) | 0.00 (0.00; 0.01) | 27.82 (26.67; 28.98) |
| **Switzerland** | 44.43 (39.77; 50.01) | 44.30 (39.66; 49.84) | -0.45 (-0.61; -0.29) | 30.93 (26.75; 35.18) | 31.19 (26.97; 35.47) | 0.65 (0.46; 0.84) | 43.50 (19.33; 82.73) | 43.37 (19.27; 82.51) | -0.45 (-1.48; 0.58) | 4.63 (4.22; 5.04) | 5.30 (4.66; 6.00) | 13.25 (13.05; 13.45) | 2.59 (2.25; 2.92) | 2.87 (2.44; 3.30) | 9.68 (9.45; 9.90) | 0.00 (0.00; 0.01) | 0.01 (0.00; 0.01) | 12.42 (11.31; 13.53) |
| **United Kingdom** | 30.30 (27.06; 34.15) | 30.00 (26.81; 33.83) | -1.11 (-1.27; -0.95) | 32.00 (28.73; 35.38) | 32.02 (28.75; 35.41) | -0.14 (-0.29; 0.01) | 29.68 (13.49; 56.83) | 29.40 (13.36; 56.27) | -1.10 (-2.12; -0.07) | 23.24 (21.00; 25.74) | 25.48 (22.78; 28.74) | 9.15 (8.98; 9.32) | 20.39 (18.47; 22.44) | 21.19 (18.73; 23.62) | 3.67 (3.50; 3.83) | 0.02 (0.01; 0.04) | 0.02 (0.01; 0.05) | 8.69 (7.61; 9.77) |
| **Latin America and Caribbean** | 31.88 (27.67; 36.77) | 31.34 (27.20; 36.12) | -1.83 (-2.03; -1.63) | 33.07 (28.97; 37.43) | 33.07 (28.97; 37.45) | -0.11 (-0.29; 0.07) | 31.20 (13.89; 59.62) | 30.68 (13.68; 58.56) | -1.80 (-2.83; -0.77) | 198.39 (172.48; 228.61) | 232.57 (200.46; 263.41) | 16.10 (15.88; 16.31) | 202.32 (177.75; 229.49) | 217.81 (186.14; 248.61) | 6.97 (6.77; 7.18) | 0.19 (0.09; 0.37) | 0.23 (0.10; 0.42) | 15.03 (13.93; 16.13) |
| **Andean Latin America** | 42.66 (37.13; 48.44) | 41.97 (36.55; 47.67) | -1.72 (-1.91; -1.54) | 30.43 (26.38; 35.42) | 31.40 (27.27; 36.43) | 2.95 (2.74; 3.16) | 41.91 (18.60; 79.06) | 41.23 (18.27; 77.84) | -1.71 (-2.72; -0.70) | 28.57 (24.77; 32.59) | 36.24 (31.03; 41.70) | 25.32 (25.08; 25.55) | 20.61 (17.85; 24.01) | 24.82 (21.01; 29.11) | 19.12 (18.86; 19.37) | 0.03 (0.01; 0.05) | 0.04 (0.02; 0.07) | 24.24 (23.06; 25.41) |
| **Bolivia (Plurinational State of)** | 43.10 (37.46; 49.19) | 42.20 (36.70; 48.14) | -2.15 (-2.34; -1.96) | 30.09 (25.73; 35.49) | 30.10 (25.72; 35.49) | -0.11 (-0.34; 0.12) | 42.27 (18.64; 79.79) | 41.40 (18.26; 78.20) | -2.13 (-3.15; -1.12) | 5.06 (4.39; 5.83) | 7.01 (5.73; 8.43) | 36.74 (36.41; 37.07) | 3.64 (3.10; 4.30) | 4.75 (3.83; 5.86) | 28.97 (28.62; 29.31) | 0.00 (0.00; 0.01) | 0.01 (0.00; 0.01) | 35.78 (34.57; 36.98) |
| **Ecuador** | 42.85 (36.96; 49.18) | 42.10 (36.32; 48.35) | -1.84 (-2.04; -1.64) | 30.46 (26.21; 34.99) | 33.56 (29.02; 38.78) | 9.68 (9.46; 9.89) | 42.06 (18.77; 79.37) | 41.33 (18.45; 77.98) | -1.83 (-2.84; -0.82) | 7.82 (6.73; 9.02) | 10.39 (8.34; 12.37) | 30.94 (30.62; 31.27) | 5.64 (4.85; 6.50) | 7.49 (5.95; 9.09) | 30.81 (30.47; 31.15) | 0.01 (0.00; 0.01) | 0.01 (0.00; 0.02) | 29.97 (28.71; 31.23) |
| **Peru** | 42.43 (36.83; 48.60) | 41.81 (36.25; 47.89) | -1.58 (-1.77; -1.38) | 30.52 (26.27; 35.73) | 30.70 (26.42; 35.81) | 0.43 (0.21; 0.65) | 41.72 (18.69; 78.82) | 41.11 (18.40; 77.74) | -1.56 (-2.57; -0.55) | 15.69 (13.55; 18.04) | 18.84 (15.72; 22.22) | 18.81 (18.55; 19.07) | 11.34 (9.74; 13.34) | 12.58 (10.37; 15.07) | 10.08 (9.81; 10.36) | 0.02 (0.01; 0.03) | 0.02 (0.01; 0.04) | 17.64 (16.49; 18.80) |
| **Caribbean** | 34.96 (31.39; 39.07) | 34.08 (30.80; 37.99) | -2.63 (-2.78; -2.48) | 32.78 (29.40; 36.35) | 32.55 (29.42; 35.87) | -0.82 (-0.96; -0.68) | 34.30 (15.46; 64.98) | 33.43 (15.16; 63.66) | -2.67 (-3.68; -1.66) | 17.23 (15.49; 19.17) | 17.94 (15.77; 20.31) | 3.73 (3.55; 3.91) | 15.72 (14.10; 17.42) | 15.74 (13.69; 17.99) | -0.08 (-0.27; 0.10) | 0.02 (0.01; 0.03) | 0.02 (0.01; 0.03) | 3.01 (2.01; 4.01) |
| **Antigua and Barbuda** | 34.82 (29.76; 40.45) | 34.30 (29.34; 39.87) | -1.62 (-1.84; -1.41) | 32.99 (28.16; 37.93) | 33.32 (28.44; 38.33) | 0.81 (0.60; 1.02) | 34.18 (14.95; 65.80) | 33.68 (14.73; 64.79) | -1.60 (-2.64; -0.55) | 0.03 (0.03; 0.04) | 0.03 (0.03; 0.04) | -1.26 (-1.54; -0.97) | 0.03 (0.03; 0.04) | 0.03 (0.02; 0.04) | -3.47 (-3.75; -3.19) | 0.00 (0.00; 0.00) | 0.00 (0.00; 0.00) | -2.10 (-3.21; -0.99) |
| **Bahamas** | 37.93 (32.58; 43.10) | 37.56 (32.26; 42.67) | -1.09 (-1.29; -0.90) | 32.47 (27.81; 38.02) | 32.56 (27.85; 38.10) | 0.13 (-0.10; 0.35) | 37.22 (16.92; 69.96) | 36.87 (16.75; 69.28) | -1.07 (-2.08; -0.07) | 0.16 (0.14; 0.18) | 0.18 (0.14; 0.21) | 9.54 (9.25; 9.83) | 0.13 (0.11; 0.15) | 0.14 (0.11; 0.17) | 2.69 (2.40; 2.98) | 0.00 (0.00; 0.00) | 0.00 (0.00; 0.00) | 8.60 (7.54; 9.67) |
| **Barbados** | 35.81 (30.96; 41.02) | 35.30 (30.50; 40.44) | -1.53 (-1.73; -1.33) | 33.01 (28.27; 38.85) | 33.29 (28.48; 39.20) | 0.68 (0.45; 0.91) | 35.19 (15.63; 67.16) | 34.71 (15.43; 66.19) | -1.50 (-2.52; -0.47) | 0.12 (0.10; 0.13) | 0.10 (0.08; 0.12) | -13.06 (-13.32; -12.80) | 0.10 (0.09; 0.12) | 0.09 (0.07; 0.11) | -14.71 (-14.99; -14.44) | 0.00 (0.00; 0.00) | 0.00 (0.00; 0.00) | -13.60 (-14.54; -12.66) |
| **Belize** | 31.24 (26.38; 36.46) | 30.59 (25.84; 35.73) | -2.16 (-2.38; -1.93) | 31.82 (28.01; 36.37) | 32.13 (28.31; 36.64) | 0.79 (0.61; 0.98) | 30.77 (14.04; 60.42) | 30.14 (13.75; 59.24) | -2.14 (-3.20; -1.09) | 0.14 (0.12; 0.16) | 0.21 (0.17; 0.26) | 52.95 (52.56; 53.34) | 0.15 (0.13; 0.17) | 0.21 (0.17; 0.25) | 37.09 (36.77; 37.42) | 0.00 (0.00; 0.00) | 0.00 (0.00; 0.00) | 51.96 (50.56; 53.37) |
| **Bermuda** | 33.29 (28.38; 38.74) | 32.88 (28.01; 38.28) | -1.39 (-1.61; -1.17) | 33.28 (28.26; 38.92) | 33.60 (28.52; 39.24) | 0.75 (0.53; 0.98) | 32.81 (14.31; 62.57) | 32.40 (14.14; 61.87) | -1.38 (-2.41; -0.34) | 0.02 (0.02; 0.03) | 0.02 (0.02; 0.02) | -21.46 (-21.68; -21.23) | 0.02 (0.02; 0.02) | 0.02 (0.01; 0.02) | -20.87 (-21.09; -20.65) | 0.00 (0.00; 0.00) | 0.00 (0.00; 0.00) | -22.30 (-23.24; -21.35) |
| **Cuba** | 35.86 (30.88; 41.45) | 35.37 (30.46; 40.87) | -1.48 (-1.69; -1.28) | 32.98 (28.09; 37.87) | 31.29 (26.66; 36.14) | -5.07 (-5.28; -4.87) | 35.26 (15.33; 68.02) | 34.78 (15.12; 67.15) | -1.47 (-2.52; -0.42) | 4.44 (3.83; 5.06) | 3.56 (2.88; 4.18) | -19.66 (-19.90; -19.42) | 3.71 (3.19; 4.28) | 2.82 (2.34; 3.46) | -23.48 (-23.72; -23.24) | 0.00 (0.00; 0.01) | 0.00 (0.00; 0.01) | -20.40 (-21.33; -19.47) |
| **Dominica** | 35.97 (30.61; 41.17) | 35.43 (30.13; 40.57) | -1.64 (-1.84; -1.43) | 32.67 (28.07; 37.30) | 32.84 (28.24; 37.46) | 0.35 (0.15; 0.55) | 35.33 (15.50; 69.17) | 34.80 (15.27; 68.10) | -1.63 (-2.70; -0.57) | 0.03 (0.02; 0.03) | 0.02 (0.02; 0.03) | -6.17 (-6.45; -5.89) | 0.02 (0.02; 0.03) | 0.02 (0.02; 0.02) | -12.20 (-12.47; -11.93) | 0.00 (0.00; 0.00) | 0.00 (0.00; 0.00) | -6.86 (-7.91; -5.81) |
| **Dominican Republic** | 35.37 (30.77; 40.79) | 34.40 (29.94; 39.66) | -2.78 (-2.98; -2.59) | 32.71 (28.11; 37.98) | 33.38 (28.66; 38.72) | 1.81 (1.59; 2.02) | 34.72 (15.18; 66.59) | 33.78 (14.77; 64.84) | -2.76 (-3.80; -1.73) | 4.00 (3.48; 4.64) | 4.69 (3.71; 5.66) | 16.81 (16.50; 17.12) | 3.68 (3.17; 4.28) | 4.29 (3.43; 5.27) | 16.03 (15.72; 16.35) | 0.00 (0.00; 0.01) | 0.00 (0.00; 0.01) | 15.95 (14.77; 17.13) |
| **Grenada** | 35.81 (31.02; 41.18) | 35.03 (30.33; 40.34) | -2.26 (-2.46; -2.06) | 33.05 (28.29; 37.84) | 33.37 (28.58; 38.15) | 0.76 (0.56; 0.97) | 35.13 (15.32; 67.41) | 34.37 (14.98; 65.96) | -2.25 (-3.29; -1.22) | 0.04 (0.03; 0.05) | 0.04 (0.03; 0.04) | -9.71 (-9.99; -9.43) | 0.04 (0.03; 0.04) | 0.03 (0.02; 0.04) | -12.92 (-13.20; -12.65) | 0.00 (0.00; 0.00) | 0.00 (0.00; 0.00) | -10.52 (-11.53; -9.50) |
| **Guyana** | 36.23 (31.10; 41.85) | 34.93 (29.97; 40.36) | -3.34 (-3.54; -3.13) | 32.62 (27.99; 37.44) | 33.25 (28.41; 38.24) | 1.55 (1.34; 1.76) | 35.35 (15.31; 67.22) | 34.08 (14.76; 64.86) | -3.32 (-4.34; -2.29) | 0.28 (0.24; 0.33) | 0.23 (0.17; 0.30) | -16.81 (-17.11; -16.51) | 0.26 (0.22; 0.29) | 0.21 (0.15; 0.27) | -17.06 (-17.38; -16.74) | 0.00 (0.00; 0.00) | 0.00 (0.00; 0.00) | -17.09 (-18.15; -16.02) |
| **Haiti** | 34.35 (29.49; 39.50) | 33.40 (28.70; 38.46) | -2.89 (-3.09; -2.68) | 31.19 (27.24; 35.58) | 31.20 (27.28; 35.64) | -0.10 (-0.29; 0.09) | 33.58 (15.52; 66.27) | 32.66 (15.10; 64.48) | -2.85 (-3.90; -1.79) | 4.30 (3.65; 5.02) | 5.76 (4.28; 7.39) | 31.80 (31.37; 32.23) | 4.22 (3.69; 4.84) | 5.24 (3.94; 6.70) | 22.52 (22.13; 22.91) | 0.00 (0.00; 0.01) | 0.01 (0.00; 0.01) | 31.08 (29.88; 32.27) |
| **Jamaica** | 36.34 (31.25; 41.65) | 35.83 (30.82; 41.02) | -1.53 (-1.73; -1.33) | 32.71 (27.99; 38.42) | 32.74 (28.01; 38.43) | -0.09 (-0.31; 0.14) | 35.73 (15.80; 67.51) | 35.24 (15.58; 66.56) | -1.51 (-2.52; -0.49) | 1.10 (0.95; 1.26) | 1.02 (0.82; 1.24) | -7.72 (-7.98; -7.45) | 0.96 (0.83; 1.12) | 0.81 (0.65; 1.00) | -15.23 (-15.49; -14.96) | 0.00 (0.00; 0.00) | 0.00 (0.00; 0.00) | -8.67 (-9.69; -7.64) |
| **Puerto Rico** | 34.29 (29.39; 39.74) | 33.96 (29.12; 39.39) | -1.08 (-1.29; -0.86) | 33.17 (28.19; 38.31) | 33.21 (28.22; 38.37) | -0.04 (-0.26; 0.18) | 33.69 (14.77; 64.31) | 33.38 (14.62; 63.72) | -1.06 (-2.09; -0.02) | 1.24 (1.07; 1.41) | 0.85 (0.68; 1.01) | -31.02 (-31.23; -30.82) | 1.10 (0.94; 1.27) | 0.70 (0.56; 0.84) | -35.52 (-35.74; -35.31) | 0.00 (0.00; 0.00) | 0.00 (0.00; 0.00) | -32.06 (-32.94; -31.18) |
| **Saint Kitts and Nevis** | 34.68 (29.26; 39.95) | 34.08 (28.76; 39.27) | -1.83 (-2.04; -1.61) | 33.22 (28.24; 38.06) | 33.48 (28.51; 38.31) | 0.58 (0.38; 0.79) | 34.04 (14.92; 64.28) | 33.46 (14.66; 63.14) | -1.82 (-2.83; -0.80) | 0.02 (0.02; 0.03) | 0.02 (0.02; 0.02) | -12.89 (-13.19; -12.58) | 0.02 (0.02; 0.02) | 0.02 (0.01; 0.02) | -15.56 (-15.87; -15.25) | 0.00 (0.00; 0.00) | 0.00 (0.00; 0.00) | -13.82 (-14.83; -12.82) |
| **Saint Lucia** | 35.76 (30.92; 41.13) | 35.08 (30.32; 40.35) | -1.99 (-2.19; -1.79) | 32.86 (28.48; 37.57) | 33.05 (28.59; 37.74) | 0.41 (0.21; 0.60) | 35.07 (15.47; 67.68) | 34.41 (15.18; 66.48) | -1.97 (-3.01; -0.93) | 0.07 (0.06; 0.08) | 0.07 (0.06; 0.08) | -4.11 (-4.36; -3.85) | 0.06 (0.05; 0.07) | 0.06 (0.05; 0.07) | -7.00 (-7.26; -6.73) | 0.00 (0.00; 0.00) | 0.00 (0.00; 0.00) | -5.08 (-6.14; -4.02) |
| **Saint Vincent and the Grenadines** | 41.02 (35.76; 46.16) | 40.14 (35.01; 45.15) | -2.21 (-2.39; -2.04) | 32.05 (27.82; 36.85) | 35.99 (31.21; 41.50) | 11.57 (11.36; 11.79) | 40.18 (18.04; 76.12) | 39.33 (17.65; 74.52) | -2.20 (-3.21; -1.19) | 0.05 (0.04; 0.06) | 0.04 (0.03; 0.05) | -12.94 (-13.18; -12.69) | 0.04 (0.03; 0.04) | 0.03 (0.03; 0.04) | -10.59 (-10.86; -10.32) | 0.00 (0.00; 0.00) | 0.00 (0.00; 0.00) | -13.60 (-14.58; -12.62) |
| **Suriname** | 27.16 (22.30; 32.47) | 26.60 (21.85; 31.81) | -2.21 (-2.47; -1.94) | 33.09 (27.94; 38.38) | 33.33 (28.13; 38.72) | 0.53 (0.31; 0.76) | 26.65 (11.65; 51.20) | 26.12 (11.41; 50.23) | -2.16 (-3.20; -1.13) | 0.16 (0.13; 0.19) | 0.18 (0.14; 0.23) | 10.98 (10.64; 11.31) | 0.20 (0.17; 0.23) | 0.22 (0.17; 0.27) | 10.03 (9.73; 10.34) | 0.00 (0.00; 0.00) | 0.00 (0.00; 0.00) | 10.44 (9.31; 11.58) |
| **Trinidad and Tobago** | 27.65 (23.37; 32.81) | 27.29 (23.06; 32.41) | -1.43 (-1.67; -1.19) | 31.57 (27.61; 36.29) | 32.22 (28.20; 36.98) | 1.84 (1.65; 2.04) | 27.18 (12.18; 53.66) | 26.83 (12.01; 52.95) | -1.43 (-2.50; -0.36) | 0.41 (0.34; 0.48) | 0.28 (0.21; 0.36) | -29.90 (-30.19; -29.62) | 0.42 (0.37; 0.49) | 0.26 (0.20; 0.33) | -37.74 (-37.99; -37.49) | 0.00 (0.00; 0.00) | 0.00 (0.00; 0.00) | -30.87 (-31.79; -29.95) |
| **United States Virgin Islands** | 33.77 (28.77; 39.26) | 33.37 (28.42; 38.82) | -1.30 (-1.52; -1.08) | 33.40 (28.52; 37.98) | 33.72 (28.79; 38.34) | 0.78 (0.58; 0.98) | 33.13 (14.62; 64.28) | 32.75 (14.43; 63.56) | -1.28 (-2.34; -0.23) | 0.03 (0.03; 0.04) | 0.02 (0.02; 0.03) | -26.49 (-26.71; -26.26) | 0.03 (0.02; 0.03) | 0.02 (0.02; 0.02) | -26.07 (-26.29; -25.85) | 0.00 (0.00; 0.00) | 0.00 (0.00; 0.00) | -27.11 (-28.01; -26.21) |
| **Central Latin America** | 29.07 (24.81; 34.08) | 28.45 (24.19; 33.31) | -2.29 (-2.51; -2.06) | 30.65 (26.66; 35.14) | 30.31 (26.29; 34.79) | -1.20 (-1.39; -1.00) | 28.50 (12.73; 54.11) | 27.89 (12.46; 52.72) | -2.27 (-3.28; -1.27) | 76.71 (65.44; 90.05) | 90.44 (76.13; 103.96) | 16.50 (16.25; 16.74) | 80.57 (70.35; 92.03) | 84.31 (71.76; 96.72) | 3.89 (3.68; 4.09) | 0.08 (0.03; 0.14) | 0.09 (0.04; 0.17) | 15.44 (14.31; 16.57) |
| **Colombia** | 31.80 (26.03; 38.04) | 31.28 (25.64; 37.41) | -1.74 (-2.00; -1.47) | 27.63 (23.28; 32.60) | 24.65 (20.34; 29.21) | -10.21 (-10.45; -9.98) | 31.28 (13.82; 58.85) | 30.77 (13.58; 57.94) | -1.73 (-2.74; -0.72) | 16.72 (13.74; 20.02) | 19.50 (15.91; 23.55) | 15.59 (15.28; 15.90) | 14.14 (12.01; 16.53) | 12.85 (10.42; 15.85) | -8.70 (-8.97; -8.43) | 0.02 (0.01; 0.03) | 0.02 (0.01; 0.04) | 14.44 (13.35; 15.54) |
| **Costa Rica** | 34.87 (29.64; 40.52) | 34.28 (29.17; 39.83) | -1.78 (-2.00; -1.57) | 31.98 (27.01; 37.47) | 32.40 (27.40; 38.05) | 1.08 (0.85; 1.32) | 34.22 (14.97; 63.95) | 33.65 (14.71; 62.85) | -1.76 (-2.76; -0.76) | 1.79 (1.52; 2.06) | 1.99 (1.61; 2.38) | 10.20 (9.90; 10.50) | 1.57 (1.34; 1.85) | 1.55 (1.28; 1.87) | -1.85 (-2.12; -1.58) | 0.00 (0.00; 0.00) | 0.00 (0.00; 0.00) | 8.90 (7.80; 10.00) |
| **El Salvador** | 35.94 (30.25; 41.61) | 35.09 (29.51; 40.63) | -2.44 (-2.66; -2.22) | 31.58 (26.85; 36.66) | 31.92 (27.13; 36.99) | 0.85 (0.63; 1.07) | 35.20 (15.52; 67.14) | 34.38 (15.18; 65.61) | -2.42 (-3.44; -1.39) | 2.33 (1.97; 2.71) | 2.08 (1.54; 2.71) | -10.91 (-11.24; -10.58) | 2.09 (1.78; 2.43) | 1.65 (1.22; 2.13) | -21.16 (-21.47; -20.85) | 0.00 (0.00; 0.00) | 0.00 (0.00; 0.00) | -11.97 (-12.99; -10.94) |
| **Guatemala** | 36.05 (30.19; 41.77) | 35.04 (29.35; 40.55) | -2.89 (-3.11; -2.67) | 31.65 (27.09; 37.31) | 31.64 (27.09; 37.18) | -0.22 (-0.44; 0.01) | 35.21 (15.44; 66.53) | 34.23 (15.00; 64.73) | -2.86 (-3.87; -1.84) | 5.54 (4.62; 6.51) | 7.37 (5.96; 8.84) | 30.84 (30.52; 31.16) | 5.23 (4.48; 6.20) | 6.17 (4.98; 7.46) | 16.37 (16.07; 16.66) | 0.01 (0.00; 0.01) | 0.01 (0.00; 0.01) | 29.64 (28.41; 30.86) |
| **Honduras** | 36.84 (31.26; 42.57) | 35.89 (30.45; 41.42) | -2.68 (-2.89; -2.47) | 31.58 (26.89; 36.92) | 31.71 (27.02; 37.10) | 0.22 (0.00; 0.45) | 36.10 (15.87; 68.44) | 35.18 (15.45; 66.71) | -2.64 (-3.66; -1.62) | 3.59 (3.00; 4.22) | 5.31 (4.36; 6.36) | 45.47 (45.13; 45.81) | 3.33 (2.82; 3.88) | 4.44 (3.55; 5.39) | 31.05 (30.71; 31.38) | 0.00 (0.00; 0.01) | 0.01 (0.00; 0.01) | 44.39 (43.06; 45.72) |
| **Mexico** | 23.26 (19.50; 27.58) | 22.74 (19.07; 26.95) | -2.33 (-2.57; -2.09) | 30.23 (26.12; 34.40) | 30.66 (26.53; 34.96) | 1.15 (0.95; 1.35) | 22.78 (10.14; 44.35) | 22.28 (9.91; 43.37) | -2.31 (-3.36; -1.26) | 31.61 (26.43; 37.57) | 37.93 (31.18; 44.80) | 18.40 (18.12; 18.69) | 40.93 (35.42; 46.38) | 44.67 (37.85; 51.94) | 7.91 (7.67; 8.14) | 0.03 (0.01; 0.06) | 0.04 (0.02; 0.07) | 17.38 (16.21; 18.55) |
| **Nicaragua** | 36.76 (31.51; 42.14) | 35.93 (30.82; 41.12) | -2.34 (-2.54; -2.14) | 31.62 (27.17; 36.62) | 31.80 (27.34; 36.88) | 0.38 (0.17; 0.60) | 36.00 (16.16; 68.31) | 35.19 (15.79; 66.77) | -2.32 (-3.33; -1.31) | 2.44 (2.06; 2.83) | 3.09 (2.36; 3.91) | 25.14 (24.73; 25.54) | 2.19 (1.88; 2.55) | 2.44 (1.90; 3.08) | 10.57 (10.22; 10.92) | 0.00 (0.00; 0.00) | 0.00 (0.00; 0.01) | 23.74 (22.58; 24.89) |
| **Panama** | 41.77 (36.08; 47.13) | 40.93 (35.35; 46.18) | -2.09 (-2.27; -1.90) | 32.74 (28.32; 38.13) | 35.15 (30.44; 40.91) | 6.88 (6.66; 7.10) | 40.99 (18.39; 78.07) | 40.17 (18.03; 76.51) | -2.07 (-3.09; -1.05) | 1.83 (1.58; 2.06) | 2.69 (2.26; 3.16) | 44.31 (44; 44.61) | 1.43 (1.24; 1.66) | 2.07 (1.69; 2.48) | 42.34 (42.01; 42.67) | 0.00 (0.00; 0.00) | 0.00 (0.00; 0.00) | 43.26 (42.01; 44.51) |
| **Venezuela (Bolivarian Republic of)** | 38.88 (33.50; 44.21) | 39.82 (34.34; 45.22) | 1.73 (1.54; 1.93) | 36.16 (31.57; 40.97) | 35.93 (31.38; 40.73) | -0.63 (-0.82; -0.45) | 38.07 (16.94; 73.26) | 38.98 (17.36; 75.02) | 1.72 (0.66; 2.77) | 10.88 (9.34; 12.30) | 10.49 (7.79; 13.24) | -4.47 (-4.78; -4.16) | 9.65 (8.44; 10.89) | 8.47 (6.31; 10.75) | -12.36 (-12.66; -12.06) | 0.01 (0.00; 0.02) | 0.01 (0.00; 0.02) | -5.47 (-6.51; -4.43) |
| **Tropical Latin America** | 31.31 (26.93; 36.28) | 30.55 (26.30; 35.38) | -2.52 (-2.73; -2.31) | 36.54 (31.90; 41.30) | 36.80 (32.14; 41.64) | 0.52 (0.34; 0.70) | 30.51 (13.45; 59.27) | 29.79 (13.12; 57.95) | -2.48 (-3.53; -1.43) | 75.88 (65.47; 87.92) | 87.96 (72.48; 104.06) | 15 (14.73; 15.28) | 85.41 (74.74; 96.55) | 92.94 (77.16; 110.84) | 8.24 (7.98; 8.50) | 0.07 (0.03; 0.14) | 0.08 (0.04; 0.16) | 13.84 (12.76; 14.91) |
| **Brazil** | 30.94 (26.57; 35.90) | 30.20 (25.99; 35.02) | -2.48 (-2.69; -2.27) | 36.80 (32.04; 41.66) | 37.07 (32.29; 41.99) | 0.53 (0.35; 0.72) | 30.15 (13.31; 58.68) | 29.44 (12.98; 57.28) | -2.44 (-3.49; -1.39) | 72.85 (62.67; 84.49) | 84.50 (69.13; 100.42) | 15.06 (14.78; 15.34) | 83.27 (72.80; 94.04) | 90.62 (74.87; 108.33) | 8.25 (7.98; 8.52) | 0.07 (0.03; 0.14) | 0.08 (0.04; 0.15) | 13.88 (12.81; 14.96) |
| **Paraguay** | 41.40 (35.67; 47.17) | 40.43 (34.87; 46.04) | -2.42 (-2.61; -2.23) | 28.90 (24.56; 34.32) | 28.99 (24.71; 34.44) | 0.18 (-0.06; 0.42) | 40.56 (17.49; 76.82) | 39.62 (17.08; 75.01) | -2.39 (-3.41; -1.37) | 3.02 (2.59; 3.46) | 3.45 (2.60; 4.32) | 13.68 (13.31; 14.05) | 2.15 (1.83; 2.55) | 2.32 (1.71; 2.98) | 7.78 (7.40; 8.17) | 0.00 (0.00; 0.01) | 0.00 (0.00; 0.01) | 12.67 (11.51; 13.83) |
| **North Africa and Middle East** | 33.92 (29.42; 39.16) | 33.38 (28.91; 38.55) | -1.72 (-1.92; -1.51) | 31.92 (27.85; 36.31) | 31.86 (27.70; 36.34) | -0.33 (-0.51; -0.14) | 33.22 (14.66; 63.70) | 32.66 (14.36; 62.54) | -1.79 (-2.83; -0.76) | 211.99 (182.57; 248.32) | 305.04 (262.57; 349.19) | 41.46 (41.21; 41.72) | 204.99 (177.92; 233.77) | 270.55 (230.26; 310.00) | 30.10 (29.86; 30.33) | 0.21 (0.09; 0.40) | 0.30 (0.13; 0.56) | 40.07 (38.82; 41.33) |
| **North Africa and Middle East** | 33.92 (29.42; 39.16) | 33.38 (28.91; 38.55) | -1.72 (-1.92; -1.51) | 31.92 (27.85; 36.31) | 31.86 (27.70; 36.34) | -0.33 (-0.51; -0.14) | 33.22 (14.66; 63.70) | 32.66 (14.36; 62.54) | -1.79 (-2.83; -0.76) | 211.99 (182.57; 248.32) | 305.04 (262.57; 349.19) | 41.46 (41.21; 41.72) | 204.99 (177.92; 233.77) | 270.55 (230.26; 310.00) | 30.10 (29.86; 30.33) | 0.21 (0.09; 0.40) | 0.30 (0.13; 0.56) | 40.07 (38.82; 41.33) |
| **Afghanistan** | 39.32 (34.15; 45.08) | 37.88 (32.95; 43.45) | -3.77 (-3.97; -3.58) | 31.13 (26.57; 36.28) | 31.60 (26.95; 36.80) | 1.34 (1.12; 1.57) | 38.10 (16.72; 71.02) | 36.71 (16.11; 68.51) | -3.76 (-4.75; -2.77) | 10.37 (8.69; 12.28) | 24.64 (15.98; 35.00) | 130.39 (129.43; 131.35) | 9.55 (7.94; 11.28) | 22.58 (14.52; 30.90) | 129 (128.10; 129.90) | 0.01 (0.00; 0.02) | 0.02 (0.01; 0.05) | 129.34 (127.45; 131.24) |
| **Algeria** | 36.02 (30.78; 42.68) | 35.01 (29.92; 41.46) | -2.87 (-3.10; -2.64) | 31.33 (26.09; 36.46) | 31.39 (26.04; 36.51) | 0.05 (-0.18; 0.29) | 35.34 (15.34; 68.33) | 34.35 (14.89; 66.44) | -2.85 (-3.90; -1.81) | 15.81 (13.43; 18.85) | 20.99 (16.13; 25.75) | 30.38 (30; 30.76) | 13.91 (11.52; 16.28) | 17.01 (13.21; 21.23) | 20.31 (19.95; 20.67) | 0.02 (0.01; 0.03) | 0.02 (0.01; 0.04) | 29.01 (27.80; 30.22) |
| **Bahrain** | 34.22 (28.79; 40.28) | 33.48 (28.11; 39.34) | -2.22 (-2.46; -1.99) | 31.82 (26.59; 36.90) | 31.90 (26.67; 37.10) | 0.08 (-0.15; 0.32) | 33.52 (14.49; 64.65) | 32.77 (14.12; 63.06) | -2.29 (-3.34; -1.25) | 0.57 (0.48; 0.68) | 0.84 (0.70; 0.98) | 44.10 (43.79; 44.41) | 0.53 (0.45; 0.62) | 0.68 (0.57; 0.81) | 26.78 (26.49; 27.07) | 0.00 (0.00; 0.00) | 0.00 (0.00; 0.00) | 41.46 (40.15; 42.77) |
| **Egypt** | 30.75 (25.62; 37.03) | 29.70 (24.77; 35.81) | -3.40 (-3.66; -3.15) | 31.73 (27.03; 36.37) | 32.42 (27.71; 37.22) | 1.92 (1.71; 2.12) | 30.20 (13.21; 57.13) | 29.17 (12.76; 55.13) | -3.40 (-4.41; -2.39) | 31.02 (25.41; 38.04) | 49.16 (38.81; 61.03) | 55.31 (54.89; 55.73) | 33.84 (28.38; 39.23) | 53.50 (43.38; 63.36) | 54.78 (54.44; 55.12) | 0.03 (0.01; 0.06) | 0.05 (0.02; 0.09) | 54.54 (53.14; 55.94) |
| **Iran (Islamic Republic of)** | 31.18 (27.49; 35.34) | 30.46 (26.84; 34.54) | -2.37 (-2.55; -2.20) | 34.57 (30.96; 38.33) | 33.43 (29.92; 37.02) | -3.30 (-3.45; -3.15) | 30.50 (13.70; 58.85) | 29.80 (13.38; 57.48) | -2.37 (-3.40; -1.33) | 28.07 (24.28; 32.19) | 34.72 (29.84; 40.03) | 22.37 (22.13; 22.61) | 30.52 (27.27; 33.92) | 31.43 (26.89; 36.42) | 2.46 (2.24; 2.68) | 0.03 (0.01; 0.05) | 0.03 (0.02; 0.07) | 20.59 (19.42; 21.76) |
| **Iraq** | 35.91 (30.30; 42.01) | 34.98 (29.50; 40.85) | -2.65 (-2.88; -2.42) | 31.60 (26.88; 36.77) | 31.71 (26.92; 36.93) | 0.20 (-0.02; 0.42) | 35.01 (15.43; 65.49) | 34.10 (15.03; 63.72) | -2.65 (-3.65; -1.66) | 14.45 (12.03; 17.12) | 22.73 (15.72; 30.36) | 54.79 (54.20; 55.38) | 13.60 (11.46; 15.85) | 19.74 (13.93; 26.15) | 43.06 (42.52; 43.60) | 0.01 (0.01; 0.03) | 0.02 (0.01; 0.04) | 53.29 (51.84; 54.74) |
| **Jordan** | 36.38 (30.71; 42.53) | 35.81 (30.23; 41.83) | -1.66 (-1.89; -1.43) | 31.04 (25.99; 36.64) | 30.39 (25.44; 35.72) | -2.16 (-2.39; -1.92) | 35.66 (15.72; 67.81) | 35.10 (15.41; 66.66) | -1.69 (-2.71; -0.67) | 4.52 (3.79; 5.36) | 8.82 (7.31; 10.33) | 89.72 (89.34; 90.10) | 4.06 (3.40; 4.83) | 7.04 (5.83; 8.52) | 68.94 (68.55; 69.32) | 0.00 (0.00; 0.01) | 0.01 (0.00; 0.02) | 87.66 (86.08; 89.25) |
| **Kuwait** | 33.61 (27.75; 39.86) | 33.25 (27.48; 39.41) | -1.20 (-1.45; -0.95) | 31.80 (26.50; 37.12) | 31.83 (26.53; 37.15) | -0.06 (-0.30; 0.17) | 32.99 (14.26; 63.56) | 32.62 (14.09; 62.87) | -1.21 (-2.26; -0.16) | 1.74 (1.39; 2.11) | 2.69 (2.19; 3.20) | 50.71 (50.34; 51.08) | 1.61 (1.34; 1.88) | 2.15 (1.74; 2.61) | 30.85 (30.50; 31.20) | 0.00 (0.00; 0.00) | 0.00 (0.00; 0.00) | 47.79 (46.44; 49.13) |
| **Lebanon** | 35.90 (30.22; 42.02) | 35.38 (29.71; 41.45) | -1.61 (-1.84; -1.38) | 31.34 (26.85; 36.01) | 31.45 (26.95; 36.07) | 0.20 (0.00; 0.41) | 35.07 (15.47; 65.94) | 34.57 (15.27; 64.99) | -1.58 (-2.59; -0.57) | 2.09 (1.76; 2.45) | 2.32 (1.82; 2.85) | 10.12 (9.79; 10.45) | 1.80 (1.53; 2.08) | 1.74 (1.36; 2.24) | -3.13 (-3.46; -2.80) | 0.00 (0.00; 0.00) | 0.00 (0.00; 0.00) | 8.68 (7.53; 9.82) |
| **Libya** | 36.80 (30.87; 42.94) | 36.20 (30.41; 42.24) | -1.74 (-1.97; -1.51) | 31.65 (26.57; 36.90) | 31.32 (26.30; 36.48) | -1.17 (-1.40; -0.94) | 35.96 (15.91; 67.16) | 35.38 (15.65; 66.12) | -1.75 (-2.75; -0.75) | 2.72 (2.25; 3.19) | 2.86 (2.26; 3.53) | 4.03 (3.71; 4.35) | 2.34 (1.96; 2.73) | 2.04 (1.56; 2.57) | -12.88 (-13.19; -12.57) | 0.00 (0.00; 0.01) | 0.00 (0.00; 0.01) | 2.25 (1.13; 3.36) |
| **Morocco** | 19.48 (16.18; 23.31) | 18.84 (15.64; 22.50) | -3.31 (-3.57; -3.06) | 32.60 (28.31; 36.91) | 33.28 (29.02; 37.74) | 1.84 (1.65; 2.03) | 19.15 (8.54; 37.40) | 18.52 (8.24; 36.16) | -3.33 (-4.38; -2.28) | 7.41 (6.13; 8.89) | 7.64 (6.08; 9.64) | 2.40 (2.08; 2.72) | 12.41 (10.78; 14.03) | 12.43 (10.02; 15.02) | -0.41 (-0.68; -0.15) | 0.01 (0.00; 0.01) | 0.01 (0.00; 0.01) | 1.50 (0.41; 2.59) |
| **Oman** | 36.78 (31.30; 42.69) | 35.97 (30.55; 41.72) | -2.32 (-2.53; -2.10) | 31.27 (26.25; 36.18) | 31.08 (26.16; 36.00) | -0.72 (-0.94; -0.49) | 36.11 (16.39; 68.52) | 35.29 (16.04; 67.06) | -2.38 (-3.39; -1.37) | 1.79 (1.49; 2.14) | 2.87 (2.39; 3.34) | 56.49 (56.16; 56.82) | 1.58 (1.33; 1.89) | 2.19 (1.83; 2.59) | 36.83 (36.53; 37.12) | 0.00 (0.00; 0.00) | 0.00 (0.00; 0.01) | 54.12 (52.75; 55.50) |
| **Palestine** | 31.98 (26.77; 38.32) | 31.29 (26.21; 37.50) | -2.23 (-2.48; -1.97) | 31.61 (27.04; 36.26) | 31.97 (27.36; 36.63) | 0.95 (0.74; 1.15) | 31.32 (13.95; 61.03) | 30.65 (13.65; 59.73) | -2.22 (-3.27; -1.17) | 1.56 (1.28; 1.90) | 2.35 (1.89; 2.86) | 48.22 (47.85; 48.60) | 1.69 (1.42; 1.96) | 2.33 (1.89; 2.76) | 35.45 (35.14; 35.76) | 0.00 (0.00; 0.00) | 0.00 (0.00; 0.00) | 46.73 (45.38; 48.08) |
| **Qatar** | 32.82 (27.46; 39.20) | 32.35 (27.05; 38.62) | -1.52 (-1.77; -1.27) | 32.27 (27.27; 36.77) | 32.38 (27.49; 36.89) | 0.20 (-0.01; 0.40) | 32.17 (13.85; 62.70) | 31.68 (13.70; 61.75) | -1.61 (-2.67; -0.54) | 1.11 (0.89; 1.37) | 2.60 (2.15; 3.08) | 125.08 (124.62; 125.53) | 1.10 (0.91; 1.28) | 2.27 (1.94; 2.62) | 100.55 (100.20; 100.90) | 0.00 (0.00; 0.00) | 0.00 (0.00; 0.00) | 120.97 (119.14; 122.79) |
| **Saudi Arabia** | 39.28 (33.84; 44.92) | 38.65 (33.26; 44.22) | -1.69 (-1.89; -1.49) | 35.86 (30.97; 40.79) | 35.95 (30.98; 40.84) | 0.11 (-0.08; 0.30) | 38.37 (16.80; 73.05) | 37.73 (16.54; 71.82) | -1.72 (-2.75; -0.69) | 15.93 (13.40; 18.67) | 20.95 (17.52; 24.43) | 29.08 (28.78; 29.38) | 14.71 (12.47; 16.93) | 16.26 (12.95; 19.36) | 9.25 (8.95; 9.55) | 0.02 (0.01; 0.03) | 0.02 (0.01; 0.04) | 26.39 (25.15; 27.63) |
| **Sudan** | 38.27 (32.83; 44.50) | 37.14 (31.85; 43.15) | -3.02 (-3.24; -2.81) | 31.22 (26.43; 35.94) | 31.29 (26.47; 35.99) | 0.08 (-0.14; 0.29) | 37.47 (16.47; 70.92) | 36.37 (15.97; 68.85) | -3.00 (-4.01; -1.99) | 15.34 (12.92; 18.30) | 26.13 (20.52; 32.42) | 65.82 (65.37; 66.26) | 13.93 (11.68; 16.18) | 21.65 (17.07; 26.67) | 51.49 (51.09; 51.90) | 0.02 (0.01; 0.03) | 0.03 (0.01; 0.05) | 64.49 (63.10; 65.88) |
| **Syrian Arab Republic** | 37.57 (31.92; 43.44) | 37.00 (31.53; 42.69) | -1.63 (-1.85; -1.42) | 30.87 (26.25; 36.05) | 30.91 (26.38; 35.98) | -0.02 (-0.24; 0.20) | 36.71 (16.44; 69.77) | 36.14 (16.13; 68.70) | -1.64 (-2.66; -0.62) | 5.48 (4.59; 6.39) | 6.66 (5.13; 8.57) | 20.06 (19.67; 20.45) | 4.44 (3.77; 5.20) | 5.06 (3.88; 6.30) | 12.56 (12.22; 12.90) | 0.01 (0.00; 0.01) | 0.01 (0.00; 0.01) | 19.15 (17.99; 20.30) |
| **Tunisia** | 36.19 (30.41; 42.23) | 35.32 (29.72; 41.13) | -2.47 (-2.70; -2.24) | 31.27 (26.96; 35.63) | 30.77 (26.56; 35.02) | -1.68 (-1.87; -1.48) | 35.45 (15.39; 66.37) | 34.61 (15.01; 64.85) | -2.44 (-3.44; -1.43) | 4.51 (3.81; 5.24) | 4.70 (3.85; 5.55) | 3.10 (2.83; 3.36) | 3.73 (3.22; 4.24) | 3.47 (2.85; 4.12) | -7.45 (-7.69; -7.21) | 0.00 (0.00; 0.01) | 0.00 (0.00; 0.01) | 1.72 (0.68; 2.76) |
| **Türkiye** | 36.69 (31.10; 42.63) | 35.90 (30.45; 41.79) | -2.16 (-2.38; -1.94) | 31.29 (26.24; 36.02) | 31.52 (26.50; 36.36) | 0.55 (0.33; 0.77) | 35.99 (15.69; 68.14) | 35.22 (15.35; 66.69) | -2.15 (-3.17; -1.13) | 32.69 (27.67; 37.84) | 33.98 (28.40; 39.42) | 3.47 (3.23; 3.71) | 26.85 (22.64; 30.84) | 25.13 (20.78; 29.63) | -6.57 (-6.81; -6.33) | 0.03 (0.01; 0.06) | 0.03 (0.02; 0.06) | 2.22 (1.22; 3.23) |
| **United Arab Emirates** | 33.29 (27.55; 39.53) | 32.93 (27.30; 39.11) | -1.19 (-1.45; -0.94) | 31.43 (26.62; 36.58) | 31.34 (26.58; 36.33) | -0.44 (-0.66; -0.22) | 32.66 (14.56; 62.66) | 32.26 (14.30; 61.72) | -1.30 (-2.33; -0.27) | 3.73 (2.85; 4.56) | 7.44 (6.00; 9.02) | 91.85 (91.37; 92.33) | 3.20 (2.64; 3.87) | 5.98 (4.84; 7.27) | 80.51 (80.07; 80.95) | 0.00 (0.00; 0.01) | 0.01 (0.00; 0.01) | 88.76 (87.12; 90.40) |
| **Yemen** | 35.59 (30.07; 41.97) | 34.38 (29.11; 40.50) | -3.56 (-3.79; -3.33) | 27.26 (22.39; 32.44) | 27.12 (22.25; 32.21) | -0.67 (-0.93; -0.41) | 34.73 (15.39; 64.51) | 33.55 (14.87; 62.38) | -3.54 (-4.53; -2.56) | 10.88 (9.06; 12.91) | 19.66 (15.51; 24.53) | 75.47 (75.01; 75.94) | 9.40 (7.45; 11.45) | 15.59 (11.86; 20.13) | 61.44 (60.94; 61.94) | 0.01 (0.00; 0.02) | 0.02 (0.01; 0.04) | 74.35 (72.86; 75.83) |
| **South Asia** | 30.71 (26.74; 35.65) | 29.56 (25.77; 34.24) | -3.76 (-3.97; -3.56) | 31.40 (27.64; 35.25) | 31.25 (27.64; 35.07) | -0.61 (-0.78; -0.44) | 29.90 (13.39; 56.12) | 28.78 (12.93; 53.78) | -3.74 (-4.73; -2.75) | 574.08 (497.72; 671.06) | 716.24 (602.56; 837.70) | 23.31 (23.04; 23.58) | 617.01 (540.95; 693.31) | 661.93 (558.58; 789.31) | 6.30 (6.06; 6.55) | 0.56 (0.25; 1.06) | 0.69 (0.31; 1.29) | 21.84 (20.70; 22.97) |
| **South Asia** | 30.71 (26.74; 35.65) | 29.56 (25.77; 34.24) | -3.76 (-3.97; -3.56) | 31.40 (27.64; 35.25) | 31.25 (27.64; 35.07) | -0.61 (-0.78; -0.44) | 29.90 (13.39; 56.12) | 28.78 (12.93; 53.78) | -3.74 (-4.73; -2.75) | 574.08 (497.72; 671.06) | 716.24 (602.56; 837.70) | 23.31 (23.04; 23.58) | 617.01 (540.95; 693.31) | 661.93 (558.58; 789.31) | 6.30 (6.06; 6.55) | 0.56 (0.25; 1.06) | 0.69 (0.31; 1.29) | 21.84 (20.70; 22.97) |
| **Bangladesh** | 30.97 (25.88; 37.24) | 29.85 (24.98; 35.90) | -3.63 (-3.88; -3.37) | 27.57 (22.94; 32.11) | 27.81 (23.23; 32.38) | 0.62 (0.38; 0.85) | 30.29 (13.26; 57.52) | 29.20 (12.79; 55.43) | -3.61 (-4.63; -2.60) | 51.69 (43.01; 62.37) | 62.17 (48.31; 77.49) | 18.86 (18.49; 19.24) | 47.82 (39.80; 56.02) | 49.62 (38.26; 62.58) | 2.68 (2.34; 3.02) | 0.05 (0.02; 0.10) | 0.06 (0.03; 0.11) | 17.44 (16.29; 18.59) |
| **Bhutan** | 29.38 (23.72; 35.19) | 28.17 (22.70; 33.74) | -4.21 (-4.49; -3.94) | 27.48 (22.84; 32.61) | 27.88 (23.24; 33.02) | 1.20 (0.95; 1.45) | 28.79 (12.68; 55.27) | 27.60 (12.13; 52.94) | -4.21 (-5.24; -3.19) | 0.23 (0.19; 0.28) | 0.29 (0.23; 0.35) | 21.48 (21.13; 21.82) | 0.23 (0.19; 0.27) | 0.24 (0.19; 0.30) | 4.27 (3.97; 4.57) | 0.00 (0.00; 0.00) | 0.00 (0.00; 0.00) | 20.17 (18.99; 21.35) |
| **India** | 31.41 (27.60; 36.24) | 30.45 (26.85; 35.02) | -3.10 (-3.29; -2.91) | 32.01 (28.38; 35.84) | 31.44 (27.96; 35.20) | -1.90 (-2.06; -1.73) | 30.56 (13.69; 57.41) | 29.63 (13.31; 55.47) | -3.07 (-4.06; -2.07) | 457.44 (400.19; 529.12) | 563.20 (465.71; 660.71) | 21.75 (21.47; 22.04) | 484.91 (430.90; 541.89) | 503.69 (421.48; 606.43) | 3.07 (2.81; 3.33) | 0.45 (0.20; 0.84) | 0.54 (0.24; 1.02) | 20.23 (19.09; 21.37) |
| **Nepal** | 28.57 (23.54; 34.57) | 27.40 (22.64; 33.16) | -4.18 (-4.44; -3.91) | 26.64 (22.09; 31.21) | 25.53 (21.22; 29.78) | -4.24 (-4.47; -4.01) | 27.83 (12.23; 52.11) | 26.69 (11.72; 50.07) | -4.15 (-5.14; -3.16) | 8.82 (7.25; 10.77) | 10.41 (8.18; 13.11) | 16.93 (16.56; 17.29) | 8.81 (7.29; 10.42) | 8.70 (6.83; 10.91) | -1.76 (-2.07; -1.45) | 0.01 (0.00; 0.02) | 0.01 (0.00; 0.02) | 15.57 (14.36; 16.78) |
| **Pakistan** | 25.99 (21.89; 31.14) | 24.91 (20.98; 29.80) | -4.21 (-4.45; -3.96) | 31.15 (26.63; 35.64) | 32.34 (27.47; 37.57) | 3.48 (3.26; 3.70) | 25.35 (11.17; 47.86) | 24.30 (10.69; 45.92) | -4.20 (-5.20; -3.20) | 55.89 (46.62; 68.40) | 80.17 (63.16; 97.79) | 41.05 (40.68; 41.42) | 75.24 (63.78; 87.02) | 99.68 (79.59; 120.30) | 30.19 (29.87; 30.51) | 0.05 (0.02; 0.10) | 0.08 (0.03; 0.15) | 39.76 (38.48; 41.04) |
| **Southeast Asia, East Asia, and Oceania** | 22.70 (19.30; 27.26) | 23.16 (19.76; 27.62) | 1.88 (1.64; 2.13) | 27.08 (23.53; 30.83) | 28.39 (24.72; 32.26) | 4.53 (4.34; 4.73) | 22.43 (9.92; 43.18) | 22.88 (10.10; 44.02) | 1.85 (0.79; 2.91) | 525.63 (446.69; 623.66) | 522.50 (441.92; 603.12) | -1.03 (-1.26; -0.80) | 579.32 (511.23; 658.98) | 527.27 (458.64; 603.76) | -9.13 (-9.32; -8.94) | 0.52 (0.23; 1.01) | 0.51 (0.23; 0.99) | -2.08 (-3.15; -1.00) |
| **East Asia** | 19.41 (16.36; 23.72) | 18.81 (15.86; 23.02) | -3.12 (-3.38; -2.85) | 23.40 (20.11; 27.25) | 23.45 (20.12; 27.36) | -0.04 (-0.26; 0.18) | 19.21 (8.53; 36.69) | 18.62 (8.24; 35.58) | -3.10 (-4.12; -2.08) | 303.80 (257.88; 364.72) | 255.70 (209.92; 298.33) | -15.84 (-16.08; -15.60) | 323.69 (282.15; 374.43) | 241.47 (203.91; 287.19) | -25.13 (-25.35; -24.91) | 0.30 (0.14; 0.58) | 0.25 (0.11; 0.49) | -16.84 (-17.86; -15.83) |
| **China** | 19.63 (16.57; 23.95) | 19.03 (16.05; 23.24) | -3.13 (-3.39; -2.87) | 23.76 (20.40; 27.68) | 23.82 (20.43; 27.77) | 0.03 (-0.19; 0.25) | 19.43 (8.64; 37.07) | 18.84 (8.34; 35.91) | -3.12 (-4.13; -2.10) | 296.62 (252.25; 355.04) | 249.59 (204.81; 291.55) | -15.87 (-16.11; -15.63) | 316.75 (276.81; 366.31) | 236.18 (198.99; 281.59) | -25.18 (-25.40; -24.96) | 0.29 (0.13; 0.57) | 0.24 (0.11; 0.48) | -16.87 (-17.89; -15.86) |
| **Democratic People's Republic of Korea** | 13.77 (10.69; 17.71) | 13.59 (10.57; 17.48) | -1.52 (-1.88; -1.16) | 14.29 (10.98; 17.84) | 14.16 (10.89; 17.68) | -1.13 (-1.47; -0.79) | 13.63 (5.87; 26.86) | 13.45 (5.79; 26.50) | -1.53 (-2.61; -0.45) | 3.94 (3.08; 5.00) | 3.49 (2.62; 4.42) | -11.21 (-11.57; -10.85) | 3.90 (3.05; 4.78) | 3.10 (2.37; 3.97) | -19.95 (-20.29; -19.62) | 0.00 (0.00; 0.01) | 0.00 (0.00; 0.01) | -11.97 (-12.98; -10.96) |
| **Taiwan (Province of China)** | 12.68 (9.75; 16.61) | 12.46 (9.57; 16.33) | -1.86 (-2.24; -1.48) | 13.33 (10.26; 16.27) | 13.24 (10.17; 16.16) | -0.86 (-1.18; -0.54) | 12.54 (5.28; 23.99) | 12.32 (5.19; 23.58) | -1.87 (-2.92; -0.83) | 3.25 (2.54; 4.11) | 2.63 (2.04; 3.26) | -18.84 (-19.16; -18.52) | 3.04 (2.43; 3.63) | 2.19 (1.75; 2.69) | -27.21 (-27.49; -26.94) | 0.00 (0.00; 0.01) | 0.00 (0.00; 0.00) | -19.85 (-20.83; -18.87) |
| **Oceania** | 39.79 (33.92; 46.82) | 38.74 (33.10; 45.63) | -2.76 (-2.99; -2.54) | 27.64 (22.85; 32.57) | 27.94 (23.01; 33.11) | 0.96 (0.70; 1.21) | 39.08 (17.08; 72.46) | 38.05 (16.63; 70.52) | -2.75 (-3.74; -1.76) | 5.21 (4.43; 6.20) | 10.19 (8.33; 12.36) | 90.68 (90.26; 91.11) | 3.91 (3.22; 4.64) | 7.61 (6.01; 9.21) | 89.77 (89.32; 90.22) | 0.01 (0.00; 0.01) | 0.01 (0.00; 0.02) | 90.02 (88.43; 91.60) |
| **American Samoa** | 38.00 (31.91; 44.49) | 37.39 (31.39; 43.82) | -1.69 (-1.92; -1.45) | 28.30 (23.88; 32.87) | 28.05 (23.66; 32.56) | -0.99 (-1.21; -0.77) | 37.23 (16.33; 69.91) | 36.64 (16.07; 68.78) | -1.68 (-2.69; -0.67) | 0.02 (0.02; 0.02) | 0.02 (0.02; 0.03) | 22.92 (22.61; 23.23) | 0.01 (0.01; 0.02) | 0.02 (0.01; 0.02) | 17.60 (17.33; 17.87) | 0.00 (0.00; 0.00) | 0.00 (0.00; 0.00) | 22.34 (21.14; 23.53) |
| **Cook Islands** | 36.69 (30.80; 43.22) | 36.03 (30.25; 42.40) | -1.89 (-2.13; -1.66) | 28.47 (23.61; 33.37) | 28.58 (23.72; 33.50) | 0.23 (-0.01; 0.47) | 36.06 (15.75; 69.84) | 35.41 (15.48; 68.59) | -1.88 (-2.93; -0.83) | 0.01 (0.01; 0.01) | 0.01 (0.01; 0.01) | 0.04 (-0.23; 0.32) | 0.00 (0.00; 0.01) | 0.00 (0.00; 0.01) | -3.94 (-4.22; -3.66) | 0.00 (0.00; 0.00) | 0.00 (0.00; 0.00) | -0.61 (-1.62; 0.39) |
| **Fiji** | 38.71 (32.98; 45.41) | 37.94 (32.35; 44.54) | -2.08 (-2.31; -1.86) | 28.23 (23.59; 32.89) | 28.60 (23.85; 33.33) | 1.14 (0.91; 1.38) | 37.98 (16.70; 69.66) | 37.23 (16.38; 68.29) | -2.08 (-3.05; -1.10) | 0.36 (0.30; 0.42) | 0.36 (0.29; 0.44) | 1.19 (0.90; 1.48) | 0.27 (0.22; 0.31) | 0.26 (0.20; 0.32) | -3.35 (-3.65; -3.04) | 0.00 (0.00; 0.00) | 0.00 (0.00; 0.00) | 0.49 (-0.57; 1.55) |
| **Guam** | 35.55 (29.01; 41.79) | 35.30 (28.83; 41.49) | -0.85 (-1.10; -0.60) | 28.42 (23.81; 34.10) | 28.45 (23.85; 34.16) | -0.05 (-0.30; 0.21) | 35.07 (15.06; 64.36) | 34.82 (14.94; 63.89) | -0.85 (-1.84; 0.14) | 0.06 (0.05; 0.07) | 0.06 (0.04; 0.07) | -4.50 (-4.78; -4.23) | 0.04 (0.04; 0.05) | 0.04 (0.03; 0.05) | -10.07 (-10.35; -9.79) | 0.00 (0.00; 0.00) | 0.00 (0.00; 0.00) | -5.44 (-6.41; -4.46) |
| **Kiribati** | 41.19 (35.69; 48.06) | 40.37 (34.92; 47.13) | -2.10 (-2.31; -1.89) | 27.72 (23.22; 32.08) | 27.70 (23.24; 32.13) | -0.21 (-0.43; 0.02) | 40.40 (17.75; 75.74) | 39.60 (17.40; 74.19) | -2.07 (-3.08; -1.07) | 0.05 (0.04; 0.06) | 0.06 (0.05; 0.07) | 23.02 (22.71; 23.33) | 0.03 (0.03; 0.04) | 0.04 (0.03; 0.05) | 15.07 (14.74; 15.40) | 0.00 (0.00; 0.00) | 0.00 (0.00; 0.00) | 22.27 (21.08; 23.46) |
| **Marshall Islands** | 40.13 (34.31; 47.29) | 39.25 (33.54; 46.31) | -2.24 (-2.47; -2.01) | 27.84 (23.00; 32.86) | 27.76 (22.91; 32.85) | -0.42 (-0.67; -0.17) | 39.32 (16.83; 73.73) | 38.46 (16.45; 72.05) | -2.23 (-3.24; -1.22) | 0.02 (0.02; 0.03) | 0.03 (0.02; 0.04) | 31.17 (30.82; 31.52) | 0.02 (0.01; 0.02) | 0.02 (0.02; 0.03) | 23.14 (22.75; 23.53) | 0.00 (0.00; 0.00) | 0.00 (0.00; 0.00) | 30.31 (29.07; 31.55) |
| **Micronesia (Federated States of)** | 40.13 (34.84; 47.08) | 39.37 (34.15; 46.17) | -2.02 (-2.24; -1.81) | 27.62 (22.86; 32.84) | 27.67 (22.92; 32.85) | 0.04 (-0.22; 0.29) | 39.37 (17.21; 74.79) | 38.62 (16.86; 73.38) | -2.02 (-3.04; -0.99) | 0.04 (0.04; 0.05) | 0.04 (0.03; 0.05) | 3.09 (2.75; 3.43) | 0.03 (0.02; 0.04) | 0.03 (0.02; 0.04) | -4.00 (-4.36; -3.64) | 0.00 (0.00; 0.00) | 0.00 (0.00; 0.00) | 2.33 (1.27; 3.38) |
| **Nauru** | 38.09 (31.82; 44.82) | 37.30 (31.17; 43.92) | -2.13 (-2.37; -1.89) | 27.87 (23.43; 32.79) | 27.89 (23.42; 32.83) | -0.06 (-0.30; 0.18) | 37.41 (16.27; 68.96) | 36.64 (15.96; 67.54) | -2.12 (-3.10; -1.13) | 0.00 (0.00; 0.00) | 0.01 (0.00; 0.01) | 53.93 (53.51; 54.34) | 0.00 (0.00; 0.00) | 0.00 (0.00; 0.01) | 47.21 (46.79; 47.64) | 0.00 (0.00; 0.00) | 0.00 (0.00; 0.00) | 53.26 (51.92; 54.59) |
| **Niue** | 38.18 (32.96; 44.67) | 37.44 (32.32; 43.74) | -2.01 (-2.22; -1.79) | 28.21 (23.48; 32.50) | 28.48 (23.66; 32.83) | 0.79 (0.56; 1.01) | 37.49 (16.58; 69.61) | 36.76 (16.27; 68.26) | -1.99 (-2.98; -1.00) | 0.00 (0.00; 0.00) | 0.00 (0.00; 0.00) | 0.83 (0.54; 1.12) | 0.00 (0.00; 0.00) | 0.00 (0.00; 0.00) | -1.17 (-1.47; -0.88) | 0.00 (0.00; 0.00) | 0.00 (0.00; 0.00) | 0.46 (-0.60; 1.53) |
| **Northern Mariana Islands** | 36.58 (30.65; 43.27) | 36.22 (30.37; 42.84) | -1.08 (-1.32; -0.84) | 28.23 (23.59; 33.06) | 28.10 (23.47; 32.91) | -0.57 (-0.81; -0.34) | 36.01 (15.41; 68.27) | 35.65 (15.25; 67.67) | -1.09 (-2.12; -0.05) | 0.02 (0.02; 0.02) | 0.02 (0.02; 0.02) | 3.68 (3.40; 3.95) | 0.01 (0.01; 0.02) | 0.01 (0.01; 0.02) | -2.97 (-3.22; -2.72) | 0.00 (0.00; 0.00) | 0.00 (0.00; 0.00) | 2.76 (1.70; 3.81) |
| **Palau** | 37.88 (31.07; 44.40) | 37.51 (30.89; 43.96) | -1.17 (-1.41; -0.92) | 28.11 (23.83; 33.54) | 28.28 (23.98; 33.66) | 0.48 (0.23; 0.72) | 37.21 (16.32; 70.23) | 36.84 (16.14; 69.46) | -1.17 (-2.19; -0.16) | 0.01 (0.01; 0.01) | 0.01 (0.01; 0.01) | -14.58 (-14.84; -14.32) | 0.01 (0.00; 0.01) | 0.00 (0.00; 0.01) | -17.92 (-18.18; -17.67) | 0.00 (0.00; 0.00) | 0.00 (0.00; 0.00) | -15.40 (-16.37; -14.43) |
| **Papua New Guinea** | 39.93 (34.10; 47.08) | 38.74 (33.13; 45.72) | -3.09 (-3.32; -2.86) | 27.56 (22.62; 32.76) | 27.93 (22.94; 33.10) | 1.19 (0.93; 1.45) | 39.20 (17.17; 72.47) | 38.04 (16.66; 70.37) | -3.07 (-4.06; -2.09) | 3.89 (3.29; 4.66) | 8.31 (6.74; 10.20) | 108.13 (107.64; 108.61) | 2.93 (2.39; 3.51) | 6.25 (4.86; 7.71) | 107.80 (107.28; 108.33) | 0.00 (0.00; 0.01) | 0.01 (0.00; 0.02) | 107.37 (105.64; 109.10) |
| **Samoa** | 39.65 (34.21; 46.07) | 38.99 (33.64; 45.31) | -1.80 (-2.01; -1.59) | 27.85 (23.49; 32.98) | 28.22 (23.79; 33.42) | 1.19 (0.94; 1.43) | 38.97 (16.90; 74.54) | 38.32 (16.61; 73.30) | -1.79 (-2.83; -0.75) | 0.08 (0.07; 0.09) | 0.12 (0.10; 0.15) | 51.98 (51.60; 52.36) | 0.06 (0.05; 0.07) | 0.09 (0.07; 0.11) | 52.26 (51.85; 52.66) | 0.00 (0.00; 0.00) | 0.00 (0.00; 0.00) | 51.77 (50.39; 53.16) |
| **Solomon Islands** | 40.94 (35.15; 47.92) | 39.79 (34.15; 46.50) | -2.94 (-3.16; -2.72) | 27.42 (23.04; 32.70) | 27.48 (23.00; 32.78) | 0.09 (-0.17; 0.34) | 40.22 (17.43; 74.92) | 39.09 (16.94; 72.64) | -2.93 (-3.93; -1.94) | 0.26 (0.22; 0.31) | 0.40 (0.32; 0.52) | 51.67 (51.23; 52.12) | 0.19 (0.16; 0.23) | 0.29 (0.22; 0.36) | 47.32 (46.90; 47.75) | 0.00 (0.00; 0.00) | 0.00 (0.00; 0.00) | 51.18 (49.82; 52.54) |
| **Tokelau** | 38.98 (33.40; 45.56) | 38.00 (32.59; 44.42) | -2.55 (-2.77; -2.34) | 27.89 (23.30; 32.49) | 28.20 (23.55; 32.80) | 0.91 (0.67; 1.14) | 38.28 (16.68; 72.66) | 37.33 (16.27; 70.85) | -2.53 (-3.55; -1.51) | 0.00 (0.00; 0.00) | 0.00 (0.00; 0.00) | 11.63 (11.35; 11.90) | 0.00 (0.00; 0.00) | 0.00 (0.00; 0.00) | 3.99 (3.70; 4.28) | 0.00 (0.00; 0.00) | 0.00 (0.00; 0.00) | 10.95 (9.83; 12.07) |
| **Tonga** | 39.29 (33.97; 45.82) | 38.49 (33.32; 44.93) | -2.12 (-2.33; -1.91) | 27.82 (23.43; 32.97) | 27.97 (23.70; 33.12) | 0.42 (0.18; 0.66) | 38.68 (16.93; 73.83) | 37.90 (16.59; 72.49) | -2.10 (-3.13; -1.07) | 0.04 (0.03; 0.05) | 0.05 (0.04; 0.06) | 34.56 (34.21; 34.90) | 0.03 (0.02; 0.03) | 0.04 (0.03; 0.05) | 33.35 (32.98; 33.71) | 0.00 (0.00; 0.00) | 0.00 (0.00; 0.00) | 34.23 (33.01; 35.45) |
| **Tuvalu** | 39.88 (33.99; 46.66) | 38.86 (33.09; 45.44) | -2.61 (-2.84; -2.39) | 27.61 (23.05; 32.80) | 27.64 (23.00; 32.76) | -0.03 (-0.28; 0.22) | 39.24 (17.31; 73.20) | 38.23 (16.85; 71.29) | -2.60 (-3.60; -1.61) | 0.00 (0.00; 0.01) | 0.01 (0.01; 0.01) | 35.80 (35.47; 36.13) | 0.00 (0.00; 0.00) | 0.00 (0.00; 0.01) | 32.86 (32.52; 33.21) | 0.00 (0.00; 0.00) | 0.00 (0.00; 0.00) | 35.43 (34.15; 36.70) |
| **Vanuatu** | 40.67 (34.93; 47.44) | 39.64 (34.10; 46.29) | -2.63 (-2.85; -2.42) | 27.83 (23.08; 32.38) | 27.97 (23.22; 32.56) | 0.36 (0.13; 0.60) | 39.98 (17.43; 74.87) | 38.97 (16.97; 73.00) | -2.62 (-3.63; -1.62) | 0.12 (0.10; 0.14) | 0.20 (0.17; 0.24) | 65.14 (64.79; 65.49) | 0.09 (0.07; 0.10) | 0.14 (0.12; 0.17) | 59.37 (59.01; 59.72) | 0.00 (0.00; 0.00) | 0.00 (0.00; 0.00) | 64.53 (63.11; 65.96) |
| **Southeast Asia** | 29.77 (25.48; 34.65) | 29.07 (24.91; 33.70) | -2.41 (-2.63; -2.20) | 34.78 (30.58; 39.15) | 35.27 (31.06; 39.71) | 1.18 (1.01; 1.36) | 29.30 (12.82; 57.09) | 28.61 (12.57; 55.60) | -2.41 (-3.46; -1.36) | 216.62 (184.94; 253.36) | 256.60 (223.52; 293.54) | 17.29 (17.06; 17.52) | 251.72 (222.05; 283.44) | 278.19 (242.56; 316.29) | 9.65 (9.46; 9.85) | 0.21 (0.09; 0.42) | 0.25 (0.11; 0.48) | 16.17 (15.01; 17.32) |
| **Cambodia** | 36.94 (31.24; 42.45) | 35.61 (30.11; 40.95) | -3.64 (-3.86; -3.43) | 37.69 (32.67; 42.77) | 37.51 (32.58; 42.61) | -0.60 (-0.78; -0.41) | 36.27 (16.18; 69.94) | 34.98 (15.61; 67.48) | -3.60 (-4.63; -2.58) | 6.21 (5.22; 7.28) | 8.32 (6.72; 10.23) | 33.21 (32.86; 33.57) | 6.58 (5.67; 7.49) | 8.42 (6.68; 10.35) | 27.28 (26.93; 27.63) | 0.01 (0.00; 0.01) | 0.01 (0.00; 0.02) | 32.26 (31; 33.52) |
| **Indonesia** | 30.81 (26.50; 35.61) | 29.98 (25.79; 34.67) | -2.76 (-2.97; -2.56) | 36.83 (32.32; 41.42) | 36.39 (31.99; 40.98) | -1.30 (-1.47; -1.13) | 30.30 (13.28; 59.19) | 29.49 (12.92; 57.67) | -2.74 (-3.80; -1.68) | 89.85 (76.78; 104.87) | 106.85 (89.81; 123.50) | 17.59 (17.34; 17.84) | 107.77 (94.43; 121.31) | 115.60 (99.01; 134.04) | 6.43 (6.21; 6.64) | 0.09 (0.04; 0.17) | 0.10 (0.05; 0.20) | 16.31 (15.15; 17.47) |
| **Lao People's Democratic Republic** | 29.96 (24.37; 35.77) | 28.81 (23.43; 34.40) | -3.92 (-4.19; -3.66) | 33.59 (28.61; 38.14) | 33.24 (28.48; 37.61) | -1.19 (-1.39; -0.99) | 29.55 (12.71; 56.71) | 28.42 (12.21; 54.48) | -3.89 (-4.93; -2.86) | 2.17 (1.74; 2.66) | 2.96 (2.34; 3.62) | 33.64 (33.26; 34.01) | 2.57 (2.18; 2.92) | 3.27 (2.64; 3.84) | 25.14 (24.85; 25.43) | 0.00 (0.00; 0.00) | 0.00 (0.00; 0.01) | 32.65 (31.36; 33.94) |
| **Malaysia** | 24.44 (19.91; 30.20) | 23.76 (19.38; 29.37) | -2.84 (-3.13; -2.55) | 25.38 (20.53; 30.05) | 25.30 (20.48; 29.96) | -0.51 (-0.77; -0.25) | 24.09 (10.47; 46.39) | 23.44 (10.18; 45.11) | -2.82 (-3.86; -1.78) | 8.21 (6.64; 10.20) | 10.09 (7.86; 12.62) | 21.86 (21.48; 22.25) | 8.64 (7.02; 10.18) | 9.57 (7.54; 11.71) | 10.21 (9.88; 10.54) | 0.01 (0.00; 0.02) | 0.01 (0.00; 0.02) | 20.90 (19.75; 22.05) |
| **Maldives** | 30.88 (25.44; 36.49) | 29.84 (24.64; 35.26) | -3.45 (-3.70; -3.20) | 33.90 (29.17; 38.65) | 33.88 (29.19; 38.59) | -0.21 (-0.40; -0.01) | 30.46 (13.33; 59.10) | 29.41 (12.89; 57.04) | -3.50 (-4.54; -2.45) | 0.17 (0.14; 0.21) | 0.27 (0.23; 0.33) | 52.36 (52; 52.72) | 0.20 (0.17; 0.23) | 0.27 (0.22; 0.31) | 33.71 (33.41; 34.01) | 0.00 (0.00; 0.00) | 0.00 (0.00; 0.00) | 50.27 (48.96; 51.58) |
| **Mauritius** | 30.19 (25.00; 35.94) | 29.50 (24.43; 35.09) | -2.38 (-2.63; -2.13) | 33.70 (28.77; 38.59) | 34.06 (29.17; 39.03) | 0.85 (0.65; 1.06) | 29.58 (12.88; 55.60) | 28.90 (12.58; 54.36) | -2.36 (-3.37; -1.35) | 0.43 (0.36; 0.50) | 0.37 (0.29; 0.45) | -14.18 (-14.45; -13.90) | 0.44 (0.38; 0.50) | 0.35 (0.28; 0.42) | -20.37 (-20.62; -20.11) | 0.00 (0.00; 0.00) | 0.00 (0.00; 0.00) | -15.24 (-16.19; -14.29) |
| **Myanmar** | 23.85 (19.26; 28.92) | 23.10 (18.67; 28.04) | -3.24 (-3.52; -2.96) | 32.61 (27.82; 37.38) | 33.21 (28.40; 38.16) | 1.62 (1.41; 1.82) | 23.51 (10.17; 46.22) | 22.78 (9.84; 44.83) | -3.20 (-4.27; -2.13) | 13.72 (11.01; 16.71) | 15.78 (12.60; 19.51) | 13.78 (13.44; 14.12) | 18.98 (16.20; 21.70) | 21.39 (17.38; 25.76) | 11.54 (11.26; 11.83) | 0.01 (0.01; 0.03) | 0.02 (0.01; 0.03) | 13 (11.86; 14.14) |
| **Philippines** | 31.95 (28.30; 36.13) | 31.22 (27.65; 35.29) | -2.36 (-2.53; -2.19) | 35.10 (31.65; 38.53) | 35.57 (32.08; 39.07) | 1.11 (0.97; 1.25) | 31.36 (14.09; 59.83) | 30.65 (13.76; 58.47) | -2.34 (-3.36; -1.32) | 35.64 (31.35; 40.66) | 51.34 (43.05; 59.57) | 41.88 (41.60; 42.17) | 41.41 (37.25; 45.53) | 54.26 (46.34; 62.83) | 29.31 (29.07; 29.55) | 0.04 (0.02; 0.07) | 0.05 (0.02; 0.10) | 40.76 (39.43; 42.09) |
| **Seychelles** | 29.95 (25.14; 35.58) | 29.21 (24.52; 34.67) | -2.50 (-2.74; -2.26) | 33.82 (29.42; 38.27) | 34.14 (29.62; 38.60) | 0.74 (0.56; 0.93) | 29.43 (12.89; 56.26) | 28.71 (12.59; 54.90) | -2.50 (-3.53; -1.47) | 0.03 (0.03; 0.04) | 0.04 (0.03; 0.05) | 28.52 (28.17; 28.88) | 0.04 (0.03; 0.04) | 0.05 (0.04; 0.06) | 27.67 (27.36; 27.98) | 0.00 (0.00; 0.00) | 0.00 (0.00; 0.00) | 27.59 (26.39; 28.79) |
| **Sri Lanka** | 30.82 (25.77; 36.56) | 30.24 (25.29; 35.87) | -2.00 (-2.24; -1.75) | 33.23 (28.22; 38.48) | 33.69 (28.61; 38.99) | 1.17 (0.95; 1.39) | 30.22 (13.34; 58.11) | 29.66 (13.08; 57.08) | -1.98 (-3.02; -0.94) | 7.26 (6.11; 8.55) | 6.96 (5.36; 8.60) | -4.67 (-4.98; -4.36) | 7.50 (6.41; 8.68) | 6.65 (5.29; 8.28) | -11.62 (-11.90; -11.33) | 0.01 (0.00; 0.01) | 0.01 (0.00; 0.01) | -5.93 (-6.98; -4.89) |
| **Thailand** | 30.73 (25.50; 36.68) | 30.06 (24.92; 35.89) | -2.27 (-2.53; -2.02) | 33.76 (29.05; 39.16) | 34.29 (29.53; 39.66) | 1.33 (1.11; 1.54) | 30.27 (13.17; 59.77) | 29.61 (12.88; 58.45) | -2.25 (-3.33; -1.18) | 23.08 (19.12; 27.18) | 19.44 (15.07; 24.09) | -15.77 (-16.08; -15.47) | 22.61 (19.57; 26.10) | 17.62 (14.21; 21.49) | -21.68 (-21.94; -21.42) | 0.02 (0.01; 0.05) | 0.02 (0.01; 0.04) | -17.11 (-18.08; -16.15) |
| **Timor-Leste** | 37.28 (31.76; 43.13) | 35.95 (30.61; 41.55) | -3.54 (-3.75; -3.32) | 32.66 (28.42; 37.18) | 32.01 (27.89; 36.41) | -2.04 (-2.22; -1.85) | 36.61 (15.90; 69.31) | 35.32 (15.34; 66.88) | -3.51 (-4.52; -2.50) | 0.48 (0.40; 0.56) | 0.74 (0.59; 0.91) | 52.03 (51.64; 52.42) | 0.46 (0.40; 0.53) | 0.67 (0.53; 0.80) | 43.20 (42.86; 43.54) | 0.00 (0.00; 0.00) | 0.00 (0.00; 0.00) | 51.29 (49.92; 52.66) |
| **Viet Nam** | 27.86 (22.55; 33.33) | 27.12 (21.97; 32.43) | -2.73 (-3.00; -2.46) | 33.40 (28.35; 38.69) | 36.69 (31.04; 42.43) | 9.15 (8.92; 9.38) | 27.54 (12.08; 53.42) | 26.80 (11.76; 52.04) | -2.71 (-3.76; -1.67) | 29.06 (23.19; 34.81) | 33.10 (26.79; 39.37) | 12.88 (12.57; 13.18) | 34.18 (29.04; 39.76) | 39.72 (32.68; 47.11) | 15.01 (14.74; 15.28) | 0.03 (0.01; 0.06) | 0.03 (0.01; 0.06) | 11.98 (10.88; 13.07) |
| **Sub-Saharan Africa** | 28.47 (24.77; 32.88) | 27.48 (23.88; 31.75) | -3.54 (-3.74; -3.34) | 28.24 (24.91; 31.89) | 28.73 (25.27; 32.64) | 1.49 (1.31; 1.67) | 27.87 (12.55; 52.52) | 26.92 (11.99; 50.45) | -3.49 (-4.48; -2.50) | 285.06 (242.97; 334.30) | 576.63 (490.26; 673.19) | 96.76 (96.41; 97.11) | 322.30 (278.67; 367.27) | 633.65 (547.19; 729.44) | 91.12 (90.82; 91.43) | 0.28 (0.12; 0.54) | 0.57 (0.25; 1.07) | 96.05 (94.46; 97.65) |
| **Central Sub-Saharan Africa** | 32.08 (26.43; 38.16) | 30.83 (25.48; 36.70) | -3.93 (-4.18; -3.68) | 28.04 (23.46; 32.87) | 27.81 (23.32; 32.60) | -1.02 (-1.25; -0.78) | 31.31 (13.62; 58.83) | 30.09 (13.09; 56.61) | -3.91 (-4.91; -2.91) | 37.67 (30.74; 45.91) | 76.85 (57.94; 97.86) | 98.45 (97.86; 99.04) | 38.14 (31.33; 45.57) | 72.91 (55.45; 92.51) | 85.84 (85.30; 86.37) | 0.04 (0.02; 0.07) | 0.08 (0.03; 0.14) | 97.60 (95.92; 99.28) |
| **Angola** | 29.95 (24.03; 36.14) | 28.58 (22.98; 34.49) | -4.64 (-4.92; -4.37) | 28.39 (23.43; 33.24) | 28.34 (23.38; 33.22) | -0.40 (-0.65; -0.16) | 29.24 (12.60; 55.17) | 27.92 (12.03; 52.69) | -4.61 (-5.61; -3.60) | 7.99 (6.32; 9.82) | 18.84 (14.26; 23.77) | 127.27 (126.64; 127.91) | 8.85 (7.20; 10.74) | 20.18 (16.21; 25.09) | 119.53 (118.99; 120.06) | 0.01 (0.00; 0.02) | 0.02 (0.01; 0.04) | 126.60 (124.71; 128.48) |
| **Central African Republic** | 33.19 (28.19; 39.25) | 31.78 (27.02; 37.63) | -4.33 (-4.56; -4.10) | 27.73 (23.07; 32.60) | 26.76 (22.26; 31.48) | -3.61 (-3.85; -3.38) | 32.27 (14.04; 60.84) | 30.91 (13.43; 58.43) | -4.31 (-5.31; -3.30) | 1.58 (1.31; 1.92) | 2.26 (1.63; 3.05) | 41.06 (40.54; 41.57) | 1.52 (1.24; 1.82) | 2.03 (1.40; 2.76) | 31.98 (31.47; 32.50) | 0.00 (0.00; 0.00) | 0.00 (0.00; 0.00) | 40.72 (39.32; 42.11) |
| **Congo** | 30.18 (24.42; 35.98) | 29.36 (23.77; 34.98) | -2.86 (-3.12; -2.59) | 28.13 (23.35; 33.16) | 27.76 (23.06; 32.78) | -1.49 (-1.74; -1.25) | 29.49 (12.94; 54.95) | 28.69 (12.58; 53.56) | -2.84 (-3.84; -1.85) | 1.53 (1.22; 1.88) | 2.44 (1.87; 3.01) | 55.44 (55; 55.88) | 1.57 (1.29; 1.87) | 2.27 (1.75; 2.84) | 41.37 (40.96; 41.79) | 0.00 (0.00; 0.00) | 0.00 (0.00; 0.00) | 54.44 (52.99; 55.89) |
| **Democratic Republic of the Congo** | 33.03 (27.23; 39.04) | 31.92 (26.33; 37.77) | -3.41 (-3.65; -3.16) | 27.93 (23.45; 32.71) | 27.62 (23.11; 32.39) | -1.28 (-1.51; -1.04) | 32.23 (14.20; 61.12) | 31.15 (13.72; 59.12) | -3.40 (-4.41; -2.38) | 25.69 (21.12; 31.25) | 51.58 (35.88; 69.40) | 95.60 (94.88; 96.33) | 25.20 (20.74; 29.99) | 46.63 (31.54; 63.39) | 80.18 (79.48; 80.88) | 0.03 (0.01; 0.05) | 0.05 (0.02; 0.10) | 94.67 (92.91; 96.42) |
| **Equatorial Guinea** | 27.75 (22.71; 33.32) | 27.04 (22.11; 32.48) | -2.70 (-2.97; -2.43) | 28.19 (23.71; 32.67) | 28.87 (24.23; 33.42) | 2.12 (1.89; 2.34) | 27.09 (11.88; 51.44) | 26.41 (11.58; 50.11) | -2.66 (-3.68; -1.64) | 0.39 (0.31; 0.48) | 0.91 (0.72; 1.11) | 126.13 (125.59; 126.67) | 0.46 (0.38; 0.54) | 0.97 (0.78; 1.17) | 104.22 (103.76; 104.68) | 0.00 (0.00; 0.00) | 0.00 (0.00; 0.00) | 124.57 (122.67; 126.46) |
| **Gabon** | 28.31 (23.65; 34.07) | 27.43 (22.92; 32.98) | -3.16 (-3.42; -2.91) | 28.18 (23.70; 33.32) | 27.51 (23.20; 32.45) | -2.48 (-2.72; -2.25) | 27.68 (12.13; 52.15) | 26.83 (11.73; 50.46) | -3.14 (-4.15; -2.14) | 0.49 (0.40; 0.59) | 0.83 (0.67; 1.05) | 66.81 (66.36; 67.26) | 0.53 (0.44; 0.63) | 0.82 (0.66; 1.02) | 52.47 (52.07; 52.87) | 0.00 (0.00; 0.00) | 0.00 (0.00; 0.00) | 66.09 (64.66; 67.52) |
| **Eastern Sub-Saharan Africa** | 31.66 (27.83; 36.02) | 30.33 (26.64; 34.48) | -4.20 (-4.38; -4.02) | 28.72 (25.37; 32.34) | 29.56 (26.04; 33.55) | 2.67 (2.49; 2.85) | 30.99 (14.01; 58.31) | 29.70 (13.39; 55.61) | -4.17 (-5.16; -3.19) | 119.15 (102.70; 137.89) | 239.47 (207.21; 276.32) | 95.31 (94.99; 95.63) | 125.25 (109.27; 142.52) | 243.78 (205.36; 279.60) | 88.99 (88.67; 89.31) | 0.12 (0.05; 0.22) | 0.24 (0.10; 0.45) | 94.42 (92.80; 96.03) |
| **Burundi** | 37.71 (31.44; 43.91) | 36.28 (30.31; 42.19) | -3.89 (-4.12; -3.66) | 28.84 (23.93; 34.60) | 28.98 (24.11; 34.72) | 0.29 (0.03; 0.55) | 36.95 (16.23; 69.36) | 35.56 (15.59; 66.71) | -3.88 (-4.87; -2.88) | 4.25 (3.47; 5.11) | 8.90 (6.90; 10.99) | 103.57 (103.05; 104.10) | 3.74 (3.04; 4.55) | 7.59 (5.72; 9.68) | 96.97 (96.40; 97.54) | 0.00 (0.00; 0.01) | 0.01 (0.00; 0.02) | 102.75 (101.02; 104.49) |
| **Comoros** | 29.15 (24.82; 34.53) | 28.22 (24.02; 33.44) | -3.22 (-3.45; -2.99) | 26.50 (23.00; 30.58) | 26.09 (22.62; 30.26) | -1.69 (-1.90; -1.49) | 28.78 (12.94; 57.50) | 27.87 (12.51; 55.69) | -3.21 (-4.29; -2.13) | 0.22 (0.18; 0.26) | 0.27 (0.20; 0.35) | 21.38 (20.96; 21.79) | 0.21 (0.18; 0.25) | 0.23 (0.17; 0.31) | 7.10 (6.71; 7.49) | 0.00 (0.00; 0.00) | 0.00 (0.00; 0.00) | 20.80 (19.56; 22.04) |
| **Djibouti** | 28.03 (23.75; 33.37) | 26.94 (22.84; 32.06) | -3.89 (-4.13; -3.65) | 26.42 (22.72; 31.21) | 26.17 (22.43; 30.84) | -1.12 (-1.35; -0.90) | 27.68 (12.62; 54.36) | 26.61 (12.16; 52.21) | -3.89 (-4.93; -2.84) | 0.35 (0.30; 0.43) | 0.59 (0.46; 0.72) | 61.79 (61.37; 62.21) | 0.36 (0.31; 0.42) | 0.52 (0.41; 0.64) | 43.37 (42.99; 43.75) | 0.00 (0.00; 0.00) | 0.00 (0.00; 0.00) | 61.01 (59.55; 62.47) |
| **Eritrea** | 41.49 (35.79; 47.68) | 39.94 (34.45; 45.94) | -3.82 (-4.02; -3.62) | 28.42 (23.95; 33.54) | 27.64 (23.31; 32.66) | -2.85 (-3.09; -2.62) | 40.59 (17.76; 75.78) | 39.08 (17.07; 72.92) | -3.80 (-4.79; -2.81) | 2.52 (2.13; 2.98) | 3.84 (2.47; 5.42) | 50.26 (49.59; 50.92) | 1.91 (1.59; 2.26) | 2.70 (1.62; 4.00) | 39.38 (38.66; 40.11) | 0.00 (0.00; 0.00) | 0.00 (0.00; 0.01) | 49.23 (47.74; 50.72) |
| **Ethiopia** | 28.17 (24.91; 32.13) | 26.74 (23.69; 30.48) | -5.08 (-5.25; -4.90) | 31.06 (28.13; 34.30) | 30.94 (28.02; 34.02) | -0.57 (-0.71; -0.43) | 27.59 (12.53; 53.09) | 26.19 (11.87; 50.44) | -5.06 (-6.07; -4.04) | 27.34 (23.98; 31.49) | 56.97 (46.37; 69.56) | 102.80 (102.34; 103.27) | 35.84 (32.05; 39.94) | 68.67 (55.66; 81.94) | 86.86 (86.46; 87.26) | 0.03 (0.01; 0.05) | 0.06 (0.03; 0.11) | 101.77 (99.98; 103.56) |
| **Kenya** | 25.55 (22.32; 29.26) | 24.68 (21.55; 28.25) | -3.42 (-3.60; -3.23) | 29.71 (26.24; 33.10) | 29.38 (25.98; 32.69) | -1.27 (-1.43; -1.11) | 25.03 (11.26; 47.77) | 24.19 (10.88; 46.15) | -3.39 (-4.40; -2.38) | 11.99 (10.32; 14.05) | 19.32 (16.28; 22.84) | 57.24 (56.93; 57.56) | 15.87 (13.79; 17.73) | 22.43 (18.63; 26.46) | 38.16 (37.88; 38.44) | 0.01 (0.01; 0.02) | 0.02 (0.01; 0.04) | 56.12 (54.74; 57.49) |
| **Madagascar** | 45.95 (40.30; 51.55) | 44.63 (39.15; 50.07) | -2.95 (-3.12; -2.78) | 31.31 (26.76; 36.33) | 35.45 (30.19; 41.58) | 12.58 (12.35; 12.82) | 45.00 (19.93; 83.93) | 43.71 (19.35; 81.50) | -2.93 (-3.92; -1.94) | 11.55 (9.93; 13.36) | 22.28 (18.65; 25.87) | 87.74 (87.39; 88.09) | 8.98 (7.61; 10.56) | 18.25 (14.91; 22.18) | 96.87 (96.43; 97.30) | 0.01 (0.01; 0.02) | 0.02 (0.01; 0.04) | 86.80 (85.30; 88.31) |
| **Malawi** | 36.86 (31.78; 42.94) | 35.56 (30.62; 41.38) | -3.59 (-3.80; -3.38) | 28.78 (23.96; 34.50) | 28.76 (23.98; 34.36) | -0.24 (-0.49; 0.02) | 36.09 (15.87; 67.89) | 34.82 (15.31; 65.49) | -3.57 (-4.57; -2.56) | 6.32 (5.26; 7.60) | 11.96 (9.71; 14.47) | 84.59 (84.17; 85) | 5.72 (4.65; 6.96) | 9.90 (7.91; 12.26) | 68.87 (68.44; 69.30) | 0.01 (0.00; 0.01) | 0.01 (0.01; 0.02) | 83.47 (81.96; 84.98) |
| **Mozambique** | 37.45 (31.90; 43.76) | 35.72 (30.38; 41.80) | -4.66 (-4.88; -4.44) | 28.61 (24.00; 33.65) | 33.61 (28.45; 39.58) | 16.78 (16.53; 17.04) | 36.38 (15.74; 66.24) | 34.71 (15.00; 63.19) | -4.62 (-5.58; -3.66) | 9.74 (8.01; 11.68) | 20.49 (16.17; 24.73) | 104.18 (103.70; 104.65) | 8.68 (7.09; 10.51) | 20.64 (16.68; 24.90) | 129.68 (129.17; 130.18) | 0.01 (0.00; 0.02) | 0.02 (0.01; 0.04) | 103.44 (101.79; 105.10) |
| **Rwanda** | 35.67 (30.00; 41.64) | 34.29 (28.81; 39.98) | -3.90 (-4.13; -3.68) | 28.74 (23.92; 33.72) | 29.48 (24.53; 34.43) | 2.29 (2.05; 2.53) | 34.99 (15.12; 66.37) | 33.64 (14.52; 63.94) | -3.86 (-4.88; -2.84) | 4.36 (3.60; 5.26) | 9.06 (7.22; 10.99) | 101.45 (100.97; 101.93) | 3.90 (3.20; 4.66) | 7.83 (6.11; 9.65) | 94.45 (93.95; 94.95) | 0.00 (0.00; 0.01) | 0.01 (0.00; 0.02) | 100.47 (98.80; 102.14) |
| **Somalia** | 39.99 (34.48; 46.22) | 38.68 (33.44; 44.68) | -3.39 (-3.59; -3.18) | 28.26 (23.55; 33.85) | 27.86 (23.12; 33.35) | -1.56 (-1.82; -1.31) | 39.06 (16.73; 74.13) | 37.78 (16.16; 71.73) | -3.36 (-4.38; -2.34) | 6.99 (5.83; 8.31) | 15.66 (10.28; 20.96) | 117 (116.20; 117.81) | 5.85 (4.76; 7.06) | 12.89 (8.56; 17.83) | 113.13 (112.30; 113.96) | 0.01 (0.00; 0.01) | 0.02 (0.01; 0.03) | 116.58 (114.60; 118.56) |
| **South Sudan** | 36.21 (30.66; 42.37) | 34.60 (29.31; 40.41) | -4.57 (-4.80; -4.35) | 29.04 (24.35; 33.87) | 29.19 (24.53; 34.07) | 0.34 (0.11; 0.57) | 35.30 (15.43; 66.25) | 33.74 (14.70; 63.48) | -4.54 (-5.54; -3.55) | 2.98 (2.46; 3.59) | 7.00 (5.27; 9.06) | 128.76 (128.09; 129.43) | 2.72 (2.24; 3.23) | 6.56 (5.05; 8.33) | 134.22 (133.59; 134.85) | 0.00 (0.00; 0.01) | 0.01 (0.00; 0.01) | 128.32 (126.41; 130.22) |
| **Uganda** | 24.01 (19.21; 29.47) | 22.92 (18.33; 28.18) | -4.56 (-4.85; -4.26) | 28.91 (23.81; 34.46) | 28.38 (23.49; 33.73) | -1.98 (-2.23; -1.72) | 23.56 (10.33; 45.86) | 22.50 (9.86; 43.88) | -4.52 (-5.56; -3.48) | 8.82 (7.08; 11.10) | 20.35 (15.67; 25.85) | 122.51 (121.90; 123.13) | 12.08 (9.77; 14.80) | 26.55 (20.73; 32.66) | 111.77 (111.24; 112.30) | 0.01 (0.00; 0.02) | 0.02 (0.01; 0.04) | 121.69 (119.84; 123.54) |
| **United Republic of Tanzania** | 32.32 (26.38; 38.33) | 31.08 (25.42; 36.94) | -3.88 (-4.14; -3.62) | 23.10 (18.92; 28.28) | 25.74 (20.70; 32.42) | 10.68 (10.36; 11.01) | 31.69 (13.90; 58.02) | 30.48 (13.35; 55.81) | -3.86 (-4.82; -2.89) | 16.76 (13.51; 20.48) | 32.66 (25.87; 40.01) | 88.53 (88.06; 88.99) | 13.62 (10.86; 17.13) | 28.12 (21.34; 36.15) | 98.40 (97.81; 98.98) | 0.02 (0.01; 0.03) | 0.03 (0.01; 0.06) | 87.72 (86.24; 89.20) |
| **Zambia** | 27.03 (22.70; 32.37) | 26.15 (21.96; 31.37) | -3.31 (-3.56; -3.06) | 26.61 (23.13; 30.97) | 27.15 (23.60; 31.46) | 1.74 (1.54; 1.95) | 26.51 (12.08; 52.64) | 25.64 (11.68; 50.95) | -3.31 (-4.37; -2.24) | 4.85 (4.06; 5.89) | 9.93 (7.92; 12.30) | 98.74 (98.25; 99.23) | 5.65 (4.85; 6.68) | 10.70 (8.37; 13.21) | 83.59 (83.12; 84.05) | 0.00 (0.00; 0.01) | 0.01 (0.00; 0.02) | 97.87 (96.21; 99.52) |
| **Southern Sub-Saharan Africa** | 25.10 (21.33; 29.97) | 24.58 (20.80; 29.44) | -2.19 (-2.43; -1.94) | 27.54 (23.94; 31.62) | 27.20 (23.70; 31.22) | -1.40 (-1.60; -1.21) | 24.43 (10.90; 46.77) | 23.94 (10.67; 45.88) | -2.15 (-3.18; -1.12) | 20.27 (17.09; 24.54) | 25.90 (21.21; 31.94) | 26.88 (26.54; 27.23) | 23.21 (20.18; 26.69) | 27.09 (22.72; 32.23) | 16.19 (15.93; 16.46) | 0.02 (0.01; 0.04) | 0.03 (0.01; 0.05) | 26.08 (24.91; 27.25) |
| **Botswana** | 25.92 (20.95; 30.88) | 25.23 (20.39; 30.08) | -2.78 (-3.05; -2.52) | 24.28 (20.51; 29.45) | 24.41 (20.64; 29.62) | 0.28 (0.02; 0.54) | 25.30 (11.06; 47.77) | 24.63 (10.74; 46.54) | -2.78 (-3.79; -1.76) | 0.64 (0.51; 0.77) | 0.85 (0.65; 1.05) | 31.17 (30.77; 31.57) | 0.62 (0.52; 0.76) | 0.76 (0.60; 0.94) | 20.59 (20.24; 20.94) | 0.00 (0.00; 0.00) | 0.00 (0.00; 0.00) | 30.05 (28.78; 31.32) |
| **Eswatini** | 26.92 (22.14; 32.51) | 26.28 (21.64; 31.70) | -2.48 (-2.75; -2.22) | 24.36 (20.38; 29.07) | 24.36 (20.36; 29.06) | -0.25 (-0.50; 0.01) | 26.19 (11.49; 49.82) | 25.57 (11.19; 48.62) | -2.48 (-3.50; -1.46) | 0.30 (0.24; 0.36) | 0.37 (0.29; 0.46) | 22.32 (21.96; 22.68) | 0.30 (0.25; 0.36) | 0.34 (0.26; 0.42) | 12.50 (12.14; 12.85) | 0.00 (0.00; 0.00) | 0.00 (0.00; 0.00) | 21.38 (20.18; 22.58) |
| **Lesotho** | 28.56 (23.15; 34.80) | 27.91 (22.62; 33.98) | -2.40 (-2.69; -2.12) | 24.29 (20.20; 28.92) | 24.58 (20.49; 29.26) | 0.94 (0.68; 1.19) | 27.73 (12.01; 52.99) | 27.10 (11.73; 51.82) | -2.40 (-3.43; -1.37) | 0.52 (0.42; 0.64) | 0.67 (0.51; 0.84) | 27.73 (27.33; 28.14) | 0.49 (0.40; 0.58) | 0.58 (0.46; 0.73) | 19.19 (18.85; 19.54) | 0.00 (0.00; 0.00) | 0.00 (0.00; 0.00) | 26.87 (25.67; 28.08) |
| **Namibia** | 30.38 (25.26; 36.49) | 29.68 (24.69; 35.63) | -2.47 (-2.73; -2.21) | 24.77 (20.52; 29.62) | 26.55 (21.99; 31.67) | 6.75 (6.48; 7.01) | 29.71 (12.93; 56.72) | 29.02 (12.61; 55.44) | -2.46 (-3.49; -1.43) | 0.71 (0.58; 0.87) | 1.08 (0.83; 1.35) | 49.30 (48.88; 49.73) | 0.63 (0.52; 0.76) | 0.95 (0.73; 1.19) | 47.12 (46.69; 47.55) | 0.00 (0.00; 0.00) | 0.00 (0.00; 0.00) | 48.47 (47.05; 49.90) |
| **South Africa** | 25.05 (21.45; 29.68) | 24.60 (21.05; 29.15) | -1.90 (-2.13; -1.67) | 29.03 (25.46; 32.89) | 28.98 (25.44; 32.79) | -0.39 (-0.57; -0.21) | 24.35 (10.92; 46.56) | 23.92 (10.73; 45.73) | -1.89 (-2.92; -0.87) | 14.78 (12.55; 17.72) | 17.80 (14.37; 21.96) | 19.83 (19.49; 20.17) | 17.44 (15.31; 19.77) | 18.99 (15.53; 22.45) | 8.68 (8.42; 8.95) | 0.01 (0.01; 0.03) | 0.02 (0.01; 0.03) | 18.90 (17.74; 20.06) |
| **Zimbabwe** | 23.51 (18.78; 29.38) | 22.94 (18.31; 28.70) | -2.57 (-2.88; -2.25) | 23.75 (19.83; 28.43) | 23.48 (19.62; 28.10) | -1.36 (-1.62; -1.11) | 23.01 (10.07; 44.65) | 22.46 (9.82; 43.68) | -2.55 (-3.60; -1.50) | 3.32 (2.62; 4.27) | 5.14 (3.77; 6.78) | 52.62 (52.09; 53.14) | 3.73 (3.09; 4.55) | 5.48 (4.26; 6.98) | 44.78 (44.35; 45.21) | 0.00 (0.00; 0.01) | 0.01 (0.00; 0.01) | 52.08 (50.64; 53.52) |
| **Western Sub-Saharan Africa** | 25.35 (22.00; 29.43) | 24.50 (21.22; 28.45) | -3.44 (-3.64; -3.23) | 28.03 (24.76; 31.35) | 28.44 (25.07; 31.98) | 1.23 (1.06; 1.40) | 24.87 (11.18; 46.77) | 24.06 (10.62; 45.11) | -3.38 (-4.38; -2.39) | 107.97 (92.38; 127.11) | 234.41 (198.63; 276.65) | 110.58 (110.19; 110.98) | 135.69 (117.39; 154.50) | 289.88 (247.27; 334.68) | 107.05 (106.70; 107.39) | 0.11 (0.05; 0.20) | 0.23 (0.10; 0.43) | 110.02 (108.37; 111.67) |
| **Benin** | 32.22 (26.46; 38.28) | 30.61 (25.15; 36.36) | -5.05 (-5.30; -4.79) | 27.51 (23.29; 32.28) | 27.79 (23.56; 32.72) | 0.77 (0.54; 1.00) | 31.57 (13.84; 60.00) | 30.00 (13.13; 56.98) | -5.01 (-6.02; -4.00) | 3.65 (2.93; 4.44) | 8.61 (6.77; 10.72) | 128.90 (128.32; 129.48) | 3.58 (2.92; 4.29) | 8.47 (6.57; 10.38) | 129.60 (129.03; 130.16) | 0.00 (0.00; 0.01) | 0.01 (0.00; 0.02) | 128.38 (126.47; 130.29) |
| **Burkina Faso** | 31.32 (25.96; 37.18) | 29.80 (24.73; 35.45) | -4.92 (-5.17; -4.67) | 27.77 (23.47; 33.31) | 31.03 (25.99; 37.17) | 11.12 (10.85; 11.39) | 30.77 (13.55; 57.99) | 29.28 (12.87; 55.26) | -4.88 (-5.88; -3.88) | 5.91 (4.78; 7.21) | 14.27 (11.45; 17.55) | 134.05 (133.51; 134.60) | 5.97 (4.87; 7.26) | 16.70 (13.37; 20.37) | 169.50 (168.91; 170.10) | 0.01 (0.00; 0.01) | 0.01 (0.01; 0.03) | 133.89 (132; 135.78) |
| **Cabo Verde** | 30.46 (25.31; 36.55) | 29.32 (24.37; 35.20) | -3.80 (-4.05; -3.54) | 27.70 (23.45; 32.25) | 28.18 (23.88; 32.74) | 1.46 (1.23; 1.68) | 29.96 (13.14; 57.37) | 28.84 (12.62; 55.32) | -3.78 (-4.81; -2.76) | 0.18 (0.15; 0.22) | 0.20 (0.16; 0.25) | 13.14 (12.78; 13.49) | 0.17 (0.14; 0.20) | 0.17 (0.13; 0.21) | 0.84 (0.51; 1.16) | 0.00 (0.00; 0.00) | 0.00 (0.00; 0.00) | 12.04 (10.90; 13.17) |
| **Cameroon** | 31.89 (25.85; 37.61) | 30.74 (24.94; 36.28) | -3.65 (-3.91; -3.40) | 27.85 (23.59; 32.35) | 27.79 (23.52; 32.30) | -0.42 (-0.64; -0.20) | 31.28 (13.54; 58.56) | 30.16 (13.07; 56.41) | -3.63 (-4.63; -2.63) | 8.86 (6.96; 10.68) | 17.51 (13.17; 22.96) | 92.19 (91.59; 92.80) | 8.78 (7.21; 10.37) | 16.41 (12.48; 20.37) | 81.64 (81.13; 82.15) | 0.01 (0.00; 0.02) | 0.02 (0.01; 0.03) | 91.24 (89.61; 92.86) |
| **Chad** | 32.49 (26.94; 38.23) | 30.85 (25.63; 36.34) | -5.13 (-5.37; -4.89) | 27.73 (23.42; 32.59) | 29.12 (24.56; 34.14) | 4.68 (4.44; 4.92) | 31.81 (14.27; 61.16) | 30.21 (13.51; 58.08) | -5.11 (-6.13; -4.10) | 4.45 (3.61; 5.42) | 13.81 (11.10; 17.51) | 198.48 (197.74; 199.21) | 4.47 (3.67; 5.40) | 15.08 (11.46; 18.59) | 223.33 (222.53; 224.13) | 0.00 (0.00; 0.01) | 0.01 (0.01; 0.03) | 198.24 (195.86; 200.62) |
| **Côte d'Ivoire** | 31.10 (25.62; 36.75) | 29.68 (24.43; 35.13) | -4.61 (-4.86; -4.37) | 27.52 (22.96; 31.77) | 27.54 (22.97; 31.78) | -0.16 (-0.38; 0.07) | 30.47 (13.29; 57.20) | 29.08 (12.65; 54.57) | -4.61 (-5.61; -3.62) | 7.62 (6.09; 9.27) | 14.50 (11.46; 18.35) | 84.82 (84.32; 85.32) | 7.57 (6.21; 8.90) | 14.03 (10.79; 17.07) | 80.01 (79.56; 80.46) | 0.01 (0.00; 0.01) | 0.01 (0.01; 0.03) | 83.94 (82.37; 85.52) |
| **Gambia** | 32.35 (26.32; 38.25) | 30.97 (25.23; 36.49) | -4.32 (-4.58; -4.07) | 27.68 (23.14; 32.62) | 27.44 (23.00; 32.42) | -1.04 (-1.28; -0.80) | 31.65 (13.87; 59.92) | 30.30 (13.24; 57.32) | -4.30 (-5.31; -3.30) | 0.68 (0.55; 0.82) | 1.18 (0.93; 1.49) | 68.70 (68.24; 69.16) | 0.67 (0.55; 0.80) | 1.08 (0.84; 1.34) | 58.18 (57.76; 58.60) | 0.00 (0.00; 0.00) | 0.00 (0.00; 0.00) | 67.94 (66.48; 69.40) |
| **Ghana** | 26.91 (22.06; 32.43) | 26.00 (21.33; 31.38) | -3.42 (-3.68; -3.15) | 27.58 (23.08; 32.52) | 27.76 (23.28; 32.68) | 0.39 (0.15; 0.63) | 26.38 (11.58; 51.31) | 25.51 (11.19; 49.56) | -3.36 (-4.41; -2.32) | 8.57 (6.87; 10.46) | 14.66 (11.42; 18.71) | 67 (66.52; 67.49) | 9.61 (7.98; 11.49) | 15.69 (12.23; 19.87) | 59.50 (59.05; 59.95) | 0.01 (0.00; 0.02) | 0.01 (0.01; 0.03) | 66.39 (64.85; 67.93) |
| **Guinea** | 32.54 (27.18; 38.78) | 30.89 (25.84; 36.86) | -5.11 (-5.35; -4.86) | 27.65 (23.17; 32.92) | 27.42 (23.00; 32.80) | -1.03 (-1.28; -0.78) | 31.89 (13.79; 59.69) | 30.29 (13.10; 56.80) | -5.06 (-6.05; -4.06) | 3.68 (3.02; 4.49) | 7.89 (6.26; 9.72) | 108.47 (107.96; 108.99) | 3.57 (2.91; 4.33) | 7.55 (6.11; 9.42) | 105.30 (104.79; 105.81) | 0.00 (0.00; 0.01) | 0.01 (0.00; 0.01) | 107.93 (106.24; 109.63) |
| **Guinea-Bissau** | 39.35 (34.16; 45.73) | 37.74 (32.77; 43.96) | -4.16 (-4.37; -3.96) | 26.96 (22.75; 31.60) | 27.65 (23.40; 32.59) | 2.29 (2.05; 2.52) | 38.50 (17.02; 74.89) | 36.94 (16.33; 71.91) | -4.13 (-5.17; -3.09) | 0.70 (0.59; 0.83) | 1.42 (1.11; 1.74) | 97.89 (97.40; 98.37) | 0.55 (0.46; 0.66) | 1.11 (0.88; 1.39) | 94.89 (94.37; 95.40) | 0.00 (0.00; 0.00) | 0.00 (0.00; 0.00) | 97.19 (95.55; 98.83) |
| **Liberia** | 33.36 (27.87; 39.32) | 32.32 (26.98; 37.99) | -3.22 (-3.46; -2.98) | 27.57 (23.10; 32.20) | 27.99 (23.48; 32.76) | 1.27 (1.04; 1.51) | 32.40 (14.16; 61.30) | 31.39 (13.74; 59.46) | -3.22 (-4.23; -2.20) | 1.64 (1.32; 1.97) | 3.06 (2.35; 3.83) | 81.22 (80.71; 81.72) | 1.53 (1.26; 1.82) | 2.68 (2.05; 3.35) | 70.55 (70.09; 71.02) | 0.00 (0.00; 0.00) | 0.00 (0.00; 0.01) | 80.07 (78.43; 81.71) |
| **Mali** | 32.45 (27.01; 38.62) | 30.85 (25.73; 36.64) | -5.00 (-5.24; -4.75) | 27.56 (22.96; 32.91) | 28.30 (23.67; 33.73) | 2.40 (2.14; 2.66) | 31.75 (13.84; 59.69) | 30.19 (13.19; 56.88) | -4.97 (-5.96; -3.97) | 6.29 (5.17; 7.70) | 16.27 (12.50; 20.71) | 150.23 (149.55; 150.91) | 6.20 (5.04; 7.45) | 16.84 (13.01; 21.21) | 162.22 (161.53; 162.90) | 0.01 (0.00; 0.01) | 0.02 (0.01; 0.03) | 149.97 (147.80; 152.14) |
| **Mauritania** | 31.51 (26.08; 37.61) | 30.31 (25.10; 36.15) | -3.86 (-4.11; -3.61) | 27.76 (23.42; 32.49) | 27.96 (23.48; 32.62) | 0.50 (0.27; 0.73) | 30.98 (13.50; 59.69) | 29.80 (12.97; 57.36) | -3.83 (-4.87; -2.80) | 1.21 (0.98; 1.47) | 2.35 (1.79; 2.97) | 88.24 (87.70; 88.77) | 1.20 (0.99; 1.41) | 2.24 (1.81; 2.76) | 81.67 (81.23; 82.11) | 0.00 (0.00; 0.00) | 0.00 (0.00; 0.00) | 87.60 (86; 89.20) |
| **Niger** | 35.39 (30.11; 41.04) | 34.07 (28.95; 39.55) | -3.85 (-4.07; -3.64) | 27.32 (22.87; 32.46) | 31.61 (26.26; 37.79) | 14.93 (14.66; 15.21) | 34.72 (15.38; 64.81) | 33.43 (14.79; 62.41) | -3.81 (-4.80; -2.83) | 6.83 (5.63; 8.20) | 21.05 (16.53; 26.60) | 194.69 (193.94; 195.43) | 6.26 (5.06; 7.63) | 23.58 (18.31; 30.01) | 257.40 (256.48; 258.32) | 0.01 (0.00; 0.01) | 0.02 (0.01; 0.04) | 194.67 (192.40; 196.95) |
| **Nigeria** | 19.27 (16.70; 22.43) | 18.51 (16.03; 21.54) | -4.04 (-4.24; -3.83) | 28.64 (25.65; 31.67) | 28.09 (25.23; 30.97) | -2.11 (-2.25; -1.96) | 18.93 (8.48; 36.94) | 18.18 (8.12; 35.48) | -4.01 (-5.05; -2.96) | 39.64 (34.31; 46.40) | 82.88 (68.06; 100.41) | 102.80 (102.37; 103.24) | 65.99 (58.14; 73.87) | 131.72 (108.67; 156.41) | 93.62 (93.24; 94.01) | 0.04 (0.02; 0.08) | 0.08 (0.04; 0.15) | 102.14 (100.53; 103.76) |
| **Sao Tome and Principe** | 31.86 (26.13; 37.86) | 30.88 (25.34; 36.69) | -3.14 (-3.39; -2.88) | 27.77 (23.55; 32.37) | 27.83 (23.58; 32.48) | 0 (-0.23; 0.22) | 31.33 (13.51; 59.47) | 30.37 (13.09; 57.72) | -3.12 (-4.15; -2.10) | 0.07 (0.05; 0.08) | 0.08 (0.06; 0.10) | 19.41 (19.01; 19.80) | 0.06 (0.05; 0.07) | 0.07 (0.05; 0.09) | 7.05 (6.71; 7.39) | 0.00 (0.00; 0.00) | 0.00 (0.00; 0.00) | 18.49 (17.28; 19.71) |
| **Senegal** | 24.45 (20.52; 29.02) | 23.51 (19.70; 27.91) | -3.88 (-4.12; -3.63) | 26.66 (22.96; 31.27) | 25.34 (21.93; 29.51) | -4.99 (-5.20; -4.78) | 24.04 (10.98; 46.65) | 23.12 (10.54; 44.79) | -3.87 (-4.90; -2.85) | 3.56 (2.95; 4.28) | 6.59 (5.22; 8.12) | 80.25 (79.80; 80.70) | 4.54 (3.93; 5.32) | 7.30 (5.93; 8.82) | 57.08 (56.71; 57.44) | 0.00 (0.00; 0.01) | 0.01 (0.00; 0.01) | 79.68 (78.14; 81.22) |
| **Sierra Leone** | 30.33 (25.98; 35.21) | 29.14 (25.02; 33.77) | -3.97 (-4.18; -3.76) | 27.10 (23.63; 30.97) | 32.36 (27.74; 37.28) | 18.54 (18.32; 18.76) | 29.78 (13.68; 57.52) | 28.62 (13.15; 55.38) | -3.97 (-4.99; -2.94) | 2.42 (2.07; 2.86) | 4.62 (3.73; 5.51) | 85.50 (85.10; 85.91) | 2.60 (2.26; 3.00) | 5.42 (4.37; 6.66) | 102.50 (102.04; 102.96) | 0.00 (0.00; 0.00) | 0.00 (0.00; 0.01) | 84.81 (83.24; 86.38) |
| **Togo** | 25.79 (21.49; 30.62) | 24.76 (20.71; 29.45) | -3.98 (-4.22; -3.73) | 26.76 (23.49; 30.86) | 26.70 (23.33; 30.74) | -0.46 (-0.66; -0.27) | 25.38 (11.59; 48.64) | 24.38 (11.14; 46.83) | -3.94 (-4.95; -2.93) | 2.00 (1.70; 2.41) | 3.45 (2.68; 4.31) | 68.73 (68.27; 69.19) | 2.38 (2.08; 2.77) | 3.72 (2.93; 4.55) | 52.53 (52.14; 52.93) | 0.00 (0.00; 0.00) | 0.00 (0.00; 0.01) | 68.21 (66.76; 69.66) |

*Footnote*

Different colors reflect different geographic levels. Red: Global; Blue: Super-Regions; Green: Regions; Yellow: Country.

TPC, total percentage change.

**Appendix Table 4.** Prevalence, incidence and burden of “other oral conditions” in 2021, with projections to 2050, in 21 GBD regions and 204 countries.

| **OTHER ORAL CONDITIONS** | | | | | | | | | | | | |
| --- | --- | --- | --- | --- | --- | --- | --- | --- | --- | --- | --- | --- |
| **Location** | **Age-standardized** | | | | | | **All Ages (nº of cases)** | | | | | |
|  | **Prevalence (%)** | | | **YLDs (per 100,000)** | | | **Prevalence (Millions)** | | | **YLDs (Millions)** | | |
|  | **2021** | **2050** | **2021-2050 TPC (%)** | **2021** | **2050** | **2021-2050 TPC (%)** | **2021** | **2050** | **2021-2050 TPC (%)** | **2021** | **2050** | **2021-2050 TPC (%)** |
| **Global** | 1.86 (1.78; 1.93) | 1.85 (1.78; 1.93) | -0.30 (-0.36; -0.24) | 53.60 (33.04; 80.21) | 53.44 (32.95; 79.94) | -0.42 (-1.04; 0.20) | 150.88 (144.57; 157.06) | 186.25 (175.81; 197.59) | 22.28 (22.19; 22.36) | 4.35 (2.68; 6.51) | 5.32 (3.30; 7.91) | 21.26 (20.58; 21.93) |
| **Central Europe, Eastern Europe, and Central Asia** | 1.86 (1.79; 1.94) | 1.86 (1.78; 1.94) | -0.31 (-0.37; -0.25) | 53.71 (33.01; 80.35) | 53.62 (32.95; 80.33) | -0.28 (-0.91; 0.34) | 8.44 (8.09; 8.79) | 8.05 (7.51; 8.67) | -4.02 (-4.12; -3.92) | 0.24 (0.15; 0.36) | 0.23 (0.14; 0.34) | -4.63 (-5.23; -4.04) |
| **Central Asia** | 1.86 (1.78; 1.93) | 1.85 (1.77; 1.92) | -0.32 (-0.38; -0.26) | 53.65 (32.79; 80.50) | 53.52 (32.71; 80.33) | -0.29 (-0.92; 0.33) | 1.79 (1.72; 1.87) | 2.33 (2.05; 2.60) | 29.07 (28.89; 29.26) | 0.05 (0.03; 0.08) | 0.07 (0.04; 0.10) | 28.20 (27.47; 28.93) |
| **Armenia** | 1.86 (1.78; 1.93) | 1.85 (1.77; 1.92) | -0.45 (-0.51; -0.39) | 53.75 (33.07; 80.88) | 53.59 (32.98; 80.67) | -0.42 (-1.05; 0.21) | 0.06 (0.06; 0.06) | 0.05 (0.04; 0.06) | -17.83 (-18.04; -17.62) | 0.00 (0.00; 0.00) | 0.00 (0.00; 0.00) | -18.81 (-19.41; -18.21) |
| **Azerbaijan** | 1.85 (1.77; 1.92) | 1.85 (1.77; 1.92) | -0.38 (-0.44; -0.33) | 53.70 (32.61; 80.21) | 53.60 (32.53; 80.00) | -0.36 (-0.98; 0.27) | 0.21 (0.20; 0.22) | 0.23 (0.20; 0.27) | 11.41 (11.19; 11.64) | 0.01 (0.00; 0.01) | 0.01 (0.00; 0.01) | 10.25 (9.58; 10.93) |
| **Georgia** | 1.85 (1.78; 1.93) | 1.85 (1.77; 1.93) | -0.25 (-0.31; -0.19) | 53.58 (32.83; 79.43) | 53.54 (32.80; 79.39) | -0.24 (-0.85; 0.38) | 0.07 (0.07; 0.07) | 0.07 (0.06; 0.08) | -7.37 (-7.56; -7.19) | 0.00 (0.00; 0.00) | 0.00 (0.00; 0.00) | -7.76 (-8.35; -7.16) |
| **Kazakhstan** | 1.86 (1.78; 1.93) | 1.85 (1.77; 1.92) | -0.35 (-0.41; -0.29) | 53.66 (32.90; 79.71) | 53.49 (32.81; 79.49) | -0.33 (-0.95; 0.28) | 0.36 (0.34; 0.37) | 0.45 (0.39; 0.51) | 23.98 (23.78; 24.18) | 0.01 (0.01; 0.02) | 0.01 (0.01; 0.02) | 23.25 (22.54; 23.96) |
| **Kyrgyzstan** | 1.86 (1.78; 1.93) | 1.85 (1.77; 1.93) | -0.27 (-0.33; -0.21) | 53.76 (33.25; 81.17) | 53.68 (33.20; 81.04) | -0.25 (-0.88; 0.38) | 0.12 (0.12; 0.13) | 0.16 (0.13; 0.19) | 28.10 (27.81; 28.39) | 0.00 (0.00; 0.01) | 0.00 (0.00; 0.01) | 27.29 (26.52; 28.05) |
| **Mongolia** | 1.86 (1.78; 1.93) | 1.86 (1.78; 1.93) | -0.20 (-0.26; -0.14) | 53.68 (32.80; 79.51) | 53.63 (32.77; 79.47) | -0.20 (-0.81; 0.42) | 0.06 (0.06; 0.06) | 0.10 (0.09; 0.11) | 64.53 (64.36; 64.70) | 0.00 (0.00; 0.00) | 0.00 (0.00; 0.00) | 63.75 (62.89; 64.61) |
| **Tajikistan** | 1.85 (1.77; 1.92) | 1.85 (1.77; 1.92) | -0.23 (-0.29; -0.17) | 53.59 (32.74; 80.77) | 53.50 (32.69; 80.61) | -0.22 (-0.85; 0.41) | 0.18 (0.17; 0.19) | 0.29 (0.24; 0.34) | 56.91 (56.61; 57.21) | 0.01 (0.00; 0.01) | 0.01 (0.01; 0.01) | 55.86 (55.02; 56.70) |
| **Turkmenistan** | 1.85 (1.77; 1.92) | 1.85 (1.77; 1.92) | -0.14 (-0.20; -0.08) | 53.62 (32.93; 79.92) | 53.59 (32.92; 79.88) | -0.14 (-0.76; 0.48) | 0.10 (0.09; 0.10) | 0.13 (0.11; 0.15) | 31.73 (31.51; 31.95) | 0.00 (0.00; 0.00) | 0.00 (0.00; 0.01) | 30.87 (30.09; 31.64) |
| **Uzbekistan** | 1.85 (1.77; 1.93) | 1.85 (1.77; 1.92) | -0.31 (-0.37; -0.26) | 53.62 (32.77; 80.55) | 53.48 (32.69; 80.35) | -0.29 (-0.92; 0.34) | 0.64 (0.61; 0.66) | 0.86 (0.60; 1.10) | 34.54 (34.07; 35.01) | 0.02 (0.01; 0.03) | 0.02 (0.01; 0.04) | 33.54 (32.68; 34.41) |
| **Central Europe** | 1.86 (1.78; 1.93) | 1.86 (1.78; 1.93) | -0.16 (-0.22; -0.10) | 53.66 (32.98; 80.45) | 53.65 (32.99; 80.42) | -0.16 (-0.78; 0.47) | 2.38 (2.28; 2.48) | 1.95 (1.82; 2.07) | -16.36 (-16.44; -16.28) | 0.07 (0.04; 0.10) | 0.06 (0.03; 0.08) | -17.02 (-17.60; -16.44) |
| **Albania** | 1.85 (1.77; 1.92) | 1.85 (1.77; 1.92) | -0.24 (-0.30; -0.18) | 53.57 (32.82; 80.08) | 53.50 (32.75; 79.99) | -0.23 (-0.86; 0.39) | 0.05 (0.05; 0.06) | 0.05 (0.04; 0.06) | -11.09 (-11.31; -10.87) | 0.00 (0.00; 0.00) | 0.00 (0.00; 0.00) | -11.83 (-12.43; -11.22) |
| **Bosnia and Herzegovina** | 1.85 (1.77; 1.92) | 1.85 (1.77; 1.92) | -0.22 (-0.28; -0.16) | 53.44 (32.60; 79.65) | 53.41 (32.59; 79.61) | -0.21 (-0.83; 0.41) | 0.07 (0.07; 0.07) | 0.05 (0.04; 0.06) | -29.35 (-29.53; -29.17) | 0.00 (0.00; 0.00) | 0.00 (0.00; 0.00) | -30.34 (-30.87; -29.80) |
| **Bulgaria** | 1.85 (1.77; 1.92) | 1.85 (1.77; 1.92) | -0.10 (-0.15; -0.04) | 53.52 (32.72; 79.97) | 53.53 (32.73; 80.01) | -0.09 (-0.72; 0.53) | 0.14 (0.14; 0.15) | 0.10 (0.09; 0.11) | -27.75 (-27.89; -27.61) | 0.00 (0.00; 0.01) | 0.00 (0.00; 0.00) | -28.09 (-28.63; -27.55) |
| **Croatia** | 1.85 (1.77; 1.92) | 1.85 (1.77; 1.92) | -0.17 (-0.23; -0.11) | 53.62 (33.14; 80.09) | 53.59 (33.12; 80.03) | -0.16 (-0.78; 0.46) | 0.09 (0.08; 0.09) | 0.06 (0.05; 0.07) | -29.03 (-29.21; -28.85) | 0.00 (0.00; 0.00) | 0.00 (0.00; 0.00) | -29.84 (-30.40; -29.28) |
| **Czechia** | 1.85 (1.77; 1.92) | 1.85 (1.77; 1.92) | -0.09 (-0.15; -0.03) | 53.49 (32.91; 79.45) | 53.51 (32.92; 79.47) | -0.09 (-0.70; 0.53) | 0.22 (0.21; 0.23) | 0.19 (0.17; 0.21) | -10.88 (-11.02; -10.75) | 0.01 (0.00; 0.01) | 0.01 (0.00; 0.01) | -11.54 (-12.10; -10.99) |
| **Hungary** | 1.85 (1.77; 1.93) | 1.85 (1.77; 1.92) | -0.21 (-0.27; -0.15) | 53.63 (32.99; 80.11) | 53.57 (32.96; 80.01) | -0.20 (-0.82; 0.42) | 0.20 (0.19; 0.21) | 0.19 (0.16; 0.21) | -5.73 (-5.91; -5.55) | 0.01 (0.00; 0.01) | 0.01 (0.00; 0.01) | -6.02 (-6.65; -5.39) |
| **Montenegro** | 1.85 (1.77; 1.92) | 1.85 (1.77; 1.92) | -0.21 (-0.27; -0.15) | 53.58 (32.62; 80.21) | 53.53 (32.59; 80.17) | -0.20 (-0.83; 0.43) | 0.01 (0.01; 0.01) | 0.01 (0.01; 0.01) | -14.19 (-14.36; -14.01) | 0.00 (0.00; 0.00) | 0.00 (0.00; 0.00) | -14.82 (-15.42; -14.22) |
| **North Macedonia** | 1.85 (1.77; 1.92) | 1.85 (1.77; 1.92) | -0.11 (-0.17; -0.05) | 53.45 (32.91; 79.14) | 53.48 (32.93; 79.17) | -0.10 (-0.72; 0.51) | 0.05 (0.04; 0.05) | 0.04 (0.03; 0.05) | -17.51 (-17.75; -17.26) | 0.00 (0.00; 0.00) | 0.00 (0.00; 0.00) | -18.51 (-19.12; -17.90) |
| **Poland** | 1.86 (1.79; 1.95) | 1.86 (1.79; 1.95) | -0.16 (-0.22; -0.10) | 53.82 (33.31; 80.35) | 53.82 (33.31; 80.37) | -0.16 (-0.78; 0.46) | 0.79 (0.76; 0.83) | 0.67 (0.59; 0.74) | -15.09 (-15.24; -14.95) | 0.02 (0.01; 0.03) | 0.02 (0.01; 0.03) | -15.91 (-16.49; -15.33) |
| **Romania** | 1.85 (1.77; 1.92) | 1.85 (1.77; 1.92) | -0.11 (-0.17; -0.06) | 53.68 (33.11; 80.72) | 53.69 (33.12; 80.73) | -0.11 (-0.74; 0.51) | 0.39 (0.37; 0.40) | 0.30 (0.26; 0.35) | -21.48 (-21.67; -21.29) | 0.01 (0.01; 0.02) | 0.01 (0.01; 0.01) | -21.97 (-22.54; -21.40) |
| **Serbia** | 1.85 (1.77; 1.92) | 1.85 (1.77; 1.92) | -0.18 (-0.24; -0.12) | 53.52 (32.64; 80.05) | 53.52 (32.64; 80.10) | -0.17 (-0.80; 0.45) | 0.18 (0.18; 0.19) | 0.15 (0.13; 0.17) | -19.15 (-19.32; -18.97) | 0.01 (0.00; 0.01) | 0.00 (0.00; 0.01) | -19.83 (-20.41; -19.26) |
| **Slovakia** | 1.85 (1.77; 1.92) | 1.85 (1.77; 1.92) | -0.14 (-0.20; -0.08) | 53.60 (33.05; 79.28) | 53.58 (33.03; 79.24) | -0.14 (-0.75; 0.47) | 0.11 (0.11; 0.12) | 0.10 (0.09; 0.11) | -9.04 (-9.18; -8.90) | 0.00 (0.00; 0.00) | 0.00 (0.00; 0.00) | -9.79 (-10.39; -9.20) |
| **Slovenia** | 1.85 (1.77; 1.92) | 1.85 (1.77; 1.92) | -0.06 (-0.12; 0.00) | 53.50 (33.04; 80.78) | 53.54 (33.05; 80.81) | -0.06 (-0.69; 0.57) | 0.04 (0.04; 0.04) | 0.04 (0.04; 0.05) | -1.09 (-1.23; -0.95) | 0.00 (0.00; 0.00) | 0.00 (0.00; 0.00) | -1.62 (-2.22; -1.01) |
| **Eastern Europe** | 1.87 (1.79; 1.95) | 1.87 (1.79; 1.95) | -0.27 (-0.33; -0.21) | 53.79 (33.23; 80.15) | 53.76 (33.22; 80.17) | -0.26 (-0.88; 0.36) | 4.27 (4.09; 4.47) | 3.77 (3.38; 4.20) | -11.34 (-11.49; -11.19) | 0.12 (0.08; 0.18) | 0.11 (0.06; 0.16) | -12.01 (-12.59; -11.43) |
| **Belarus** | 1.86 (1.78; 1.93) | 1.86 (1.78; 1.93) | -0.28 (-0.33; -0.22) | 53.68 (32.97; 79.18) | 53.65 (32.96; 79.15) | -0.26 (-0.87; 0.35) | 0.19 (0.18; 0.20) | 0.17 (0.15; 0.19) | -11.87 (-12.05; -11.68) | 0.01 (0.00; 0.01) | 0.00 (0.00; 0.01) | -12.42 (-12.99; -11.86) |
| **Estonia** | 1.85 (1.77; 1.93) | 1.85 (1.77; 1.92) | -0.23 (-0.29; -0.18) | 53.61 (32.98; 80.16) | 53.55 (32.96; 80.05) | -0.22 (-0.84; 0.40) | 0.03 (0.03; 0.03) | 0.02 (0.02; 0.03) | -7.62 (-7.76; -7.47) | 0.00 (0.00; 0.00) | 0.00 (0.00; 0.00) | -8.10 (-8.69; -7.51) |
| **Latvia** | 1.86 (1.78; 1.93) | 1.85 (1.78; 1.93) | -0.27 (-0.33; -0.21) | 53.64 (32.87; 80.14) | 53.59 (32.83; 80.06) | -0.26 (-0.88; 0.36) | 0.04 (0.04; 0.04) | 0.03 (0.02; 0.03) | -27.21 (-27.35; -27.06) | 0.00 (0.00; 0.00) | 0.00 (0.00; 0.00) | -27.66 (-28.20; -27.12) |
| **Lithuania** | 1.86 (1.78; 1.93) | 1.86 (1.78; 1.93) | -0.24 (-0.30; -0.18) | 53.57 (32.93; 80.64) | 53.54 (32.90; 80.58) | -0.23 (-0.86; 0.40) | 0.06 (0.05; 0.06) | 0.04 (0.03; 0.04) | -29.49 (-29.64; -29.35) | 0.00 (0.00; 0.00) | 0.00 (0.00; 0.00) | -30.05 (-30.61; -29.49) |
| **Republic of Moldova** | 1.86 (1.78; 1.93) | 1.86 (1.78; 1.93) | -0.19 (-0.25; -0.14) | 53.62 (33.08; 80.23) | 53.61 (33.08; 80.22) | -0.18 (-0.80; 0.44) | 0.08 (0.07; 0.08) | 0.05 (0.03; 0.06) | -35.27 (-35.52; -35.02) | 0.00 (0.00; 0.00) | 0.00 (0.00; 0.00) | -36.42 (-36.95; -35.88) |
| **Russian Federation** | 1.87 (1.79; 1.95) | 1.87 (1.79; 1.95) | -0.29 (-0.35; -0.23) | 53.80 (33.25; 80.35) | 53.76 (33.21; 80.30) | -0.27 (-0.89; 0.34) | 2.98 (2.85; 3.12) | 2.79 (2.44; 3.18) | -5.81 (-6.00; -5.63) | 0.08 (0.05; 0.13) | 0.08 (0.05; 0.12) | -6.44 (-7.05; -5.84) |
| **Ukraine** | 1.87 (1.79; 1.95) | 1.87 (1.79; 1.95) | -0.25 (-0.31; -0.18) | 53.79 (32.85; 79.68) | 53.79 (32.83; 79.74) | -0.23 (-0.85; 0.38) | 0.91 (0.87; 0.95) | 0.66 (0.53; 0.80) | -25.85 (-26.09; -25.60) | 0.03 (0.02; 0.04) | 0.02 (0.01; 0.03) | -26.68 (-27.27; -26.09) |
| **High-income** | 1.85 (1.78; 1.93) | 1.85 (1.78; 1.93) | -0.21 (-0.26; -0.15) | 53.43 (32.90; 79.65) | 53.38 (32.89; 79.55) | -0.24 (-0.86; 0.38) | 22.14 (21.29; 22.98) | 22.83 (21.75; 23.96) | 2.76 (2.69; 2.83) | 0.63 (0.39; 0.94) | 0.65 (0.40; 0.97) | 2.23 (1.60; 2.86) |
| **Australasia** | 1.85 (1.77; 1.93) | 1.85 (1.77; 1.93) | -0.25 (-0.31; -0.19) | 53.34 (32.85; 79.46) | 53.28 (32.81; 79.38) | -0.25 (-0.86; 0.37) | 0.62 (0.59; 0.65) | 0.84 (0.79; 0.90) | 34.49 (34.39; 34.60) | 0.02 (0.01; 0.03) | 0.02 (0.01; 0.04) | 34.03 (33.30; 34.76) |
| **Australia** | 1.85 (1.77; 1.93) | 1.85 (1.76; 1.93) | -0.27 (-0.33; -0.20) | 53.31 (32.88; 79.27) | 53.24 (32.84; 79.12) | -0.26 (-0.87; 0.36) | 0.52 (0.49; 0.54) | 0.70 (0.65; 0.76) | 34.68 (34.55; 34.81) | 0.01 (0.01; 0.02) | 0.02 (0.01; 0.03) | 34.23 (33.49; 34.96) |
| **New Zealand** | 1.86 (1.78; 1.94) | 1.86 (1.78; 1.94) | -0.20 (-0.26; -0.14) | 53.48 (32.77; 79.98) | 53.44 (32.75; 79.91) | -0.19 (-0.81; 0.43) | 0.10 (0.10; 0.11) | 0.14 (0.13; 0.15) | 33.57 (33.43; 33.72) | 0.00 (0.00; 0.00) | 0.00 (0.00; 0.01) | 33.07 (32.34; 33.79) |
| **High-income Asia Pacific** | 1.85 (1.78; 1.93) | 1.85 (1.78; 1.93) | -0.09 (-0.14; -0.03) | 53.81 (32.86; 80.77) | 53.86 (32.84; 80.85) | -0.09 (-0.72; 0.54) | 3.85 (3.70; 4.00) | 3.32 (3.11; 3.55) | -13.61 (-13.70; -13.52) | 0.11 (0.07; 0.17) | 0.09 (0.06; 0.14) | -14.31 (-14.91; -13.71) |
| **Brunei Darussalam** | 1.84 (1.76; 1.92) | 1.84 (1.76; 1.92) | 0.01 (-0.05; 0.08) | 53.20 (32.59; 80.36) | 53.24 (32.64; 80.44) | 0.01 (-0.62; 0.65) | 0.01 (0.01; 0.01) | 0.01 (0.01; 0.01) | 5.52 (5.30; 5.74) | 0.00 (0.00; 0.00) | 0.00 (0.00; 0.00) | 3.60 (2.94; 4.26) |
| **Japan** | 1.86 (1.78; 1.94) | 1.86 (1.78; 1.94) | -0.13 (-0.19; -0.07) | 53.98 (32.92; 81.16) | 54.00 (32.94; 81.20) | -0.13 (-0.76; 0.50) | 2.63 (2.52; 2.74) | 2.18 (1.99; 2.36) | -16.70 (-16.81; -16.58) | 0.08 (0.05; 0.11) | 0.06 (0.04; 0.09) | -17.11 (-17.71; -16.52) |
| **Republic of Korea** | 1.84 (1.76; 1.92) | 1.85 (1.76; 1.93) | -0.03 (-0.09; 0.03) | 53.45 (32.94; 79.90) | 53.58 (33.04; 80.13) | -0.03 (-0.65; 0.59) | 1.09 (1.04; 1.14) | 0.97 (0.90; 1.05) | -11.01 (-11.11; -10.91) | 0.03 (0.02; 0.05) | 0.03 (0.02; 0.04) | -12.38 (-12.97; -11.79) |
| **Singapore** | 1.84 (1.76; 1.92) | 1.85 (1.76; 1.93) | 0.01 (-0.05; 0.08) | 53.70 (32.84; 80.08) | 53.78 (32.88; 80.21) | 0.01 (-0.61; 0.64) | 0.12 (0.11; 0.13) | 0.16 (0.14; 0.17) | 28.17 (28.02; 28.32) | 0.00 (0.00; 0.01) | 0.00 (0.00; 0.01) | 27.01 (26.31; 27.71) |
| **High-income North America** | 1.85 (1.78; 1.93) | 1.85 (1.78; 1.93) | -0.22 (-0.28; -0.16) | 53.20 (33.05; 79.58) | 53.16 (33.01; 79.51) | -0.19 (-0.81; 0.42) | 7.45 (7.17; 7.75) | 8.13 (7.60; 8.72) | 8.58 (8.47; 8.69) | 0.21 (0.13; 0.32) | 0.23 (0.14; 0.35) | 8.15 (7.49; 8.81) |
| **Canada** | 1.85 (1.77; 1.92) | 1.85 (1.77; 1.92) | -0.23 (-0.29; -0.17) | 53.43 (32.90; 80.19) | 53.39 (32.88; 80.10) | -0.22 (-0.85; 0.40) | 0.76 (0.73; 0.79) | 0.98 (0.91; 1.05) | 27.07 (26.95; 27.19) | 0.02 (0.01; 0.03) | 0.03 (0.02; 0.04) | 26.73 (26.03; 27.42) |
| **Greenland** | 1.84 (1.76; 1.91) | 1.84 (1.77; 1.92) | 0.18 (0.13; 0.24) | 52.99 (32.69; 80.10) | 53.17 (32.81; 80.31) | 0.17 (-0.46; 0.81) | 0.00 (0.00; 0.00) | 0.00 (0.00; 0.00) | 5.62 (5.47; 5.78) | 0.00 (0.00; 0.00) | 0.00 (0.00; 0.00) | 4.83 (4.18; 5.47) |
| **United States of America** | 1.86 (1.78; 1.93) | 1.85 (1.78; 1.93) | -0.21 (-0.27; -0.15) | 53.17 (33.05; 79.52) | 53.13 (33.01; 79.45) | -0.20 (-0.82; 0.42) | 6.69 (6.42; 6.97) | 7.15 (6.61; 7.75) | 6.46 (6.34; 6.58) | 0.19 (0.12; 0.28) | 0.20 (0.12; 0.31) | 6.00 (5.35; 6.66) |
| **Southern Latin America** | 1.85 (1.77; 1.93) | 1.85 (1.77; 1.93) | -0.33 (-0.40; -0.27) | 53.40 (32.78; 79.25) | 53.35 (32.74; 79.15) | -0.33 (-0.94; 0.29) | 1.33 (1.26; 1.38) | 1.49 (1.33; 1.65) | 11.18 (11.02; 11.34) | 0.04 (0.02; 0.06) | 0.04 (0.03; 0.06) | 10.20 (9.51; 10.88) |
| **Argentina** | 1.85 (1.77; 1.93) | 1.85 (1.77; 1.93) | -0.35 (-0.41; -0.28) | 53.48 (32.80; 79.35) | 53.43 (32.76; 79.30) | -0.33 (-0.95; 0.28) | 0.88 (0.84; 0.92) | 0.98 (0.84; 1.14) | 9.79 (9.57; 10.02) | 0.03 (0.02; 0.04) | 0.03 (0.02; 0.04) | 8.82 (8.12; 9.52) |
| **Chile** | 1.85 (1.77; 1.93) | 1.85 (1.77; 1.93) | -0.27 (-0.34; -0.21) | 53.21 (32.67; 79.13) | 53.16 (32.65; 79.07) | -0.26 (-0.88; 0.36) | 0.37 (0.36; 0.39) | 0.44 (0.40; 0.49) | 17 (16.85; 17.16) | 0.01 (0.01; 0.02) | 0.01 (0.01; 0.02) | 15.95 (15.28; 16.62) |
| **Uruguay** | 1.85 (1.77; 1.93) | 1.85 (1.77; 1.93) | -0.39 (-0.45; -0.32) | 53.45 (32.99; 79.67) | 53.36 (32.95; 79.50) | -0.37 (-0.99; 0.24) | 0.07 (0.06; 0.07) | 0.06 (0.06; 0.07) | -3.23 (-3.42; -3.03) | 0.00 (0.00; 0.00) | 0.00 (0.00; 0.00) | -3.87 (-4.48; -3.27) |
| **Western Europe** | 1.85 (1.77; 1.93) | 1.85 (1.77; 1.93) | -0.21 (-0.27; -0.15) | 53.46 (32.87; 79.34) | 53.42 (32.85; 79.28) | -0.20 (-0.82; 0.41) | 8.89 (8.54; 9.27) | 9.05 (8.57; 9.53) | 1.44 (1.36; 1.51) | 0.25 (0.16; 0.38) | 0.26 (0.16; 0.38) | 1.01 (0.39; 1.62) |
| **Andorra** | 1.84 (1.76; 1.92) | 1.85 (1.76; 1.93) | 0.06 (0.00; 0.12) | 53.26 (32.52; 78.51) | 53.38 (32.60; 78.68) | 0.05 (-0.56; 0.66) | 0.00 (0.00; 0.00) | 0.00 (0.00; 0.00) | -17.08 (-17.23; -16.94) | 0.00 (0.00; 0.00) | 0.00 (0.00; 0.00) | -18.47 (-19.05; -17.88) |
| **Austria** | 1.85 (1.76; 1.93) | 1.85 (1.76; 1.93) | -0.16 (-0.23; -0.10) | 53.40 (32.66; 79.91) | 53.38 (32.64; 79.89) | -0.16 (-0.78; 0.47) | 0.18 (0.18; 0.19) | 0.20 (0.18; 0.22) | 7.68 (7.54; 7.82) | 0.01 (0.00; 0.01) | 0.01 (0.00; 0.01) | 7.15 (6.52; 7.78) |
| **Belgium** | 1.85 (1.76; 1.93) | 1.85 (1.76; 1.93) | -0.16 (-0.22; -0.10) | 53.33 (32.64; 79.23) | 53.32 (32.63; 79.21) | -0.15 (-0.77; 0.47) | 0.23 (0.22; 0.24) | 0.26 (0.23; 0.29) | 11.78 (11.63; 11.94) | 0.01 (0.00; 0.01) | 0.01 (0.00; 0.01) | 11.46 (10.82; 12.10) |
| **Cyprus** | 1.85 (1.77; 1.93) | 1.84 (1.76; 1.92) | -0.43 (-0.49; -0.37) | 53.54 (33.03; 80.26) | 53.36 (32.91; 79.96) | -0.42 (-1.04; 0.20) | 0.03 (0.03; 0.03) | 0.03 (0.03; 0.04) | 13.21 (13.01; 13.41) | 0.00 (0.00; 0.00) | 0.00 (0.00; 0.00) | 12.20 (11.55; 12.86) |
| **Denmark** | 1.85 (1.76; 1.93) | 1.85 (1.76; 1.93) | -0.12 (-0.19; -0.06) | 53.36 (32.79; 79.24) | 53.34 (32.78; 79.21) | -0.12 (-0.73; 0.50) | 0.12 (0.11; 0.12) | 0.13 (0.12; 0.14) | 9.79 (9.65; 9.93) | 0.00 (0.00; 0.01) | 0.00 (0.00; 0.01) | 9.61 (8.96; 10.27) |
| **Finland** | 1.84 (1.76; 1.93) | 1.85 (1.76; 1.93) | -0.07 (-0.13; -0.01) | 53.26 (32.77; 79.20) | 53.29 (32.78; 79.25) | -0.07 (-0.69; 0.55) | 0.11 (0.11; 0.12) | 0.11 (0.10; 0.12) | -2.41 (-2.56; -2.26) | 0.00 (0.00; 0.00) | 0.00 (0.00; 0.00) | -2.74 (-3.35; -2.14) |
| **France** | 1.85 (1.77; 1.93) | 1.85 (1.76; 1.93) | -0.29 (-0.35; -0.23) | 53.43 (32.85; 78.44) | 53.34 (32.80; 78.32) | -0.28 (-0.88; 0.33) | 1.33 (1.27; 1.38) | 1.28 (1.14; 1.43) | -3.35 (-3.50; -3.19) | 0.04 (0.02; 0.06) | 0.04 (0.02; 0.05) | -3.83 (-4.43; -3.24) |
| **Germany** | 1.84 (1.76; 1.92) | 1.84 (1.76; 1.92) | -0.03 (-0.09; 0.03) | 53.16 (32.67; 78.78) | 53.21 (32.71; 78.80) | -0.03 (-0.64; 0.59) | 1.75 (1.67; 1.82) | 1.73 (1.55; 1.89) | -1.33 (-1.48; -1.18) | 0.05 (0.03; 0.07) | 0.05 (0.03; 0.07) | -1.59 (-2.21; -0.98) |
| **Greece** | 1.85 (1.77; 1.93) | 1.85 (1.76; 1.93) | -0.28 (-0.34; -0.21) | 53.44 (33.08; 79.66) | 53.35 (33.03; 79.50) | -0.27 (-0.88; 0.35) | 0.21 (0.20; 0.22) | 0.16 (0.14; 0.18) | -22.18 (-22.36; -21.99) | 0.01 (0.00; 0.01) | 0.00 (0.00; 0.01) | -22.71 (-23.26; -22.15) |
| **Iceland** | 1.84 (1.76; 1.92) | 1.84 (1.76; 1.92) | -0.02 (-0.08; 0.05) | 53.41 (32.75; 79.91) | 53.41 (32.75; 79.91) | -0.02 (-0.64; 0.61) | 0.01 (0.01; 0.01) | 0.01 (0.01; 0.01) | 37.39 (37.22; 37.56) | 0.00 (0.00; 0.00) | 0.00 (0.00; 0.00) | 36.94 (36.18; 37.71) |
| **Ireland** | 1.85 (1.77; 1.93) | 1.85 (1.77; 1.93) | -0.29 (-0.35; -0.22) | 53.42 (33.02; 79.73) | 53.35 (32.98; 79.63) | -0.28 (-0.89; 0.34) | 0.10 (0.09; 0.10) | 0.12 (0.11; 0.14) | 24.86 (24.69; 25.02) | 0.00 (0.00; 0.00) | 0.00 (0.00; 0.01) | 24.18 (23.47; 24.89) |
| **Israel** | 1.85 (1.77; 1.93) | 1.85 (1.76; 1.93) | -0.25 (-0.32; -0.19) | 53.55 (33.09; 80.65) | 53.47 (33.03; 80.50) | -0.24 (-0.87; 0.38) | 0.18 (0.17; 0.19) | 0.25 (0.22; 0.29) | 39.36 (39.11; 39.62) | 0.01 (0.00; 0.01) | 0.01 (0.00; 0.01) | 38.81 (38.05; 39.56) |
| **Italy** | 1.86 (1.78; 1.94) | 1.86 (1.78; 1.94) | -0.27 (-0.33; -0.21) | 53.73 (32.80; 80.19) | 53.67 (32.78; 80.11) | -0.26 (-0.88; 0.37) | 1.24 (1.19; 1.30) | 1.01 (0.92; 1.11) | -18.36 (-18.49; -18.24) | 0.04 (0.02; 0.05) | 0.03 (0.02; 0.04) | -19.05 (-19.62; -18.49) |
[truncated: 147,324 more chars]
